# Alcohol‐Directed Carboamination of Conjugated Enynes

**DOI:** 10.1002/anie.6963888

**Published:** 2026-05-02

**Authors:** Helena Solé‐Àvila, Duncan K. Brownsey, Hugo Senelle, Jerome Waser

**Affiliations:** ^1^ Laboratory of Catalysis and Organic Synthesis Institute of Chemistry and Chemical Engineering, École Polytechnique Fédérale de Lausanne Lausanne Switzerland; ^2^ National Centre for Competence in Research‐Catalysis (NCCR‐Catalysis) Switzerland

**Keywords:** allene, carboamination, enyne, multicomponent, Pd‐catalysis

## Abstract

We report a Pd‐catalyzed carboamination of conjugated enynes for the direct synthesis of functionalized allenic amines. The reaction proceeds through a selective 1,4‐coupling of anilines and aryl triflates with enynes, utilizing a free alcohol as a native directing group. The obtained allenes undergo versatile transformations, including cyclization to dihydropyrans, hydroamination, and tandem carboamination sequences. Notably, the use of primary anilines enables a second carboamination to generate multifunctionalized 3‐pyrroline heterocycles through dual C‐N bond formation with two distinct aryl triflates. The transformation represents a novel approach to allene synthesis and heterocycle construction through sequential carboamination events.

## Graphical abstract

The first general three‐component intermolecular Pd‐catalyzed carboamination of conjugated enynes enables the coupling of anilines and aryl triflates to produce functionalized allenic amines, utilizing a native alcohol directing group to control regioselectivity. The allenic products undergo diverse transformations, including cyclization to dihydropyrans and sequential carboamination reactions. Notably, primary anilines enable a second carboamination with different aryl triflates, generating multifunctionalized 3‐pyrrolines through dual C‐N bond formation.

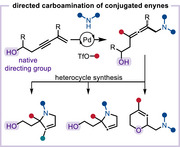

## 1 Introduction

**SCHEME 1:**
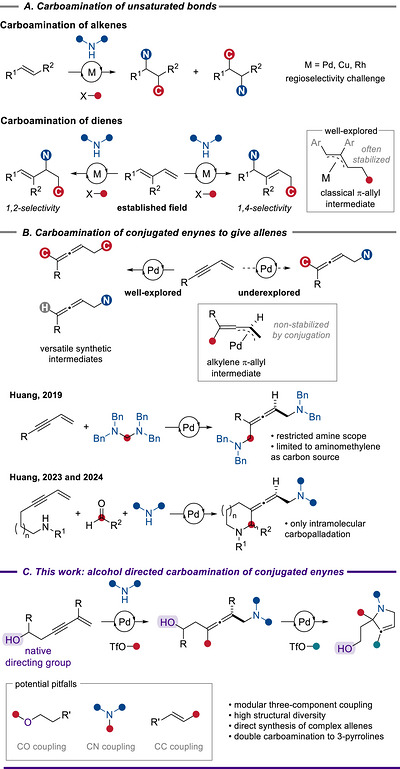
(A) Carboamination of unsaturated bonds; (B) current methods for the synthesis of aminated allenes; (C) our work.

Transition‐metal‐catalyzed carboamination reactions of π bonds, which introduce a carbon and a nitrogen substituent in a single step, represent powerful transformations for the rapid assembly of nitrogen‐containing building blocks [1, 2], which are essential for the synthesis of pharmaceuticals, agrochemicals, and natural products (Scheme 1A) [3, 4]. Among transition metals, palladium is particularly advantageous for difunctionalization reactions due to its versatile reactivity, functional group tolerance, and well‐established organometallic chemistry [5]. Early carboamination methodologies largely relied on intramolecular strategies in which the nucleophilic nitrogen source is covalently tethered to the alkene or alkyne substrate [6, 7, 8, 9]. Three‐component intermolecular carboaminations offer greater structural diversity [10], but face challenges with selectivity, competing two‐component pathways, and stability of organometallic intermediates. Among strategies to control selectivity in intermolecular carboaminations, the use of conjugated π systems has emerged as a particularly effective approach. Conjugated dienes and enynes can lead to the formation of stabilized intermediates with high regioselectivity, making them versatile building blocks in synthesis [11, 12]. Diene carboamination in particular is well‐established under both thermal [13, 14, 15, 16, 17] and photoinduced [18, 19, 20, 21] conditions, with protocols enabling both 1,2‐ and 1,4‐selectivity (Scheme 1A). These transformations typically proceed through stable π‐allyl intermediates, often in conjugation with neighboring aromatic groups. In contrast, enyne carboamination remains underexplored despite the straightforward access to 1,3‐enynes through cross‐coupling methodologies [22]. The key difference lies in the reactive intermediate: enynes form alkylene‐π‐allyl species lacking additional stabilization (Scheme 1B). While enyne dicarbofunctionalization [23, 24, 25] and hydroamination [26, 27] have been investigated, carboamination methods remain limited to two examples only [28, 29]. The carboamination of conjugated enynes by Huang and coworkers reported the synthesis of allenic 1,5‐diamines from symmetrical diamine precursors, though the scope is restricted to aminomethylene insertion as the carbon source [30]. More recently, the same group described a three‐component intramolecular carbopalladation of aminated enynes with aldehydes and amines to afford cyclic allenic amines, yet this approach is limited to cyclic structures [31, 32]. Ma and coworkers reported an alternative 1,2‐carboamination strategy starting from 2‐alkynyl‐1,4‐diol dicarbonates instead of enynes as starting materials, but only dibenzylamine could be used as a nucleophile [33]. These constraints highlight the challenge of developing general carboamination protocols for multicomponent allene synthesis, which are particularly attractive given their presence in natural products [34, 35, 36] and utility as versatile synthetic intermediates [37, 38, 39, 40, 41, 42].

To overcome the challenges associated with carboamination, several strategies have been explored. Our group has previously developed an in situ tethering strategy, which combines the selectivity advantages of intramolecular reactions with the ability to access linear products after tether removal [43, 44, 45]. Alternatively, directing group approaches using strongly coordinating auxiliaries can be used, but this strategy requires installation and removal steps [46, 47]. Leveraging native functional groups instead offers a more streamlined and step‐economical solution. Free alcohols are particularly attractive due to their ubiquity in both synthetic and biomass‐derived compounds, low cost, and versatility in subsequent derivatization. Alcohol‐directed C–H activation was successful in several Pd‐catalyzed transformations [48, 49, 50, 51]. However, using alcohols as directing groups in Pd‐catalyzed difunctionalization reactions of π bonds involving nucleophilic partners remains challenging [52, 53] due to competing pathways such as β‐hydride elimination, double‐bond isomerization, cyclization, or O‐arylation [54, 55, 56, 57].

Herein, we report a Pd‐catalyzed, free alcohol‐directed intermolecular carboamination of conjugated enynes, enabling the direct synthesis of highly functionalized allenes (Scheme 1C). In this transformation, the native hydroxyl group serves as a weak yet effective directing group, enabling selective three‐component carboamination of the enyne and overcoming potential two‐component side reactions such as C–O, C–N, or C–C coupling. Notably, a double carboamination sequence under the same reaction conditions allowed access to 3‐pyrrolines [58].

**SCHEME 2:**
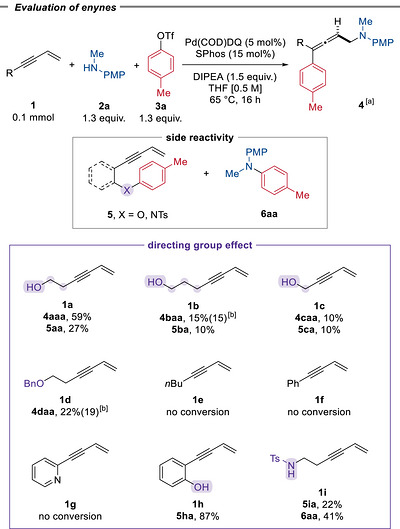
Evaluation of enynes. ^a1^H NMR yields were determined with TCE as an internal standard. ^b^Isolated yield (0.3 mmol scale).

After screening amine nucleophiles, aryl electrophiles, and enynes, promising results were obtained with hex‐5‐en‐3‐yn‐1‐ol (**1a**), bearing the alcohol directing group positioned two carbon atoms away from the enyne, secondary aniline **2a**, and *p*‐tolyl triflate (**3a**) (Scheme 2). Using a combination of Pd(COD)DQ as a Pd^0^ precursor [59] and SPhos as a ligand, allene **4aaa** was obtained in 59% yield. The main‐side product resulted from the C–O coupling between enyne **1a** and aryl triflate **3a** in 27% NMR yield (**5aa**). With a three‐carbon linker (**1b**), the allene **4baa** was obtained in 15% yield, alongside the O‐arylation product **5ba** in 10% yield, while shortening to a one‐carbon linker (**1c**) decreased the yield to 10% for both **4caa** and **5ca**. Using a benzyl protected alcohol (**1d**), the allene product **4daa** was isolated in 19% yield, whereas in absence of a coordinating O atom, no allene product was detected (**1e**, **1f**, and **1g**). In the case of 2‐(but‐3‐en‐1‐yn‐1‐yl)phenol (**1h**), O‐arylation was favored to give **5ha** in 87% yield.

When employing a tosyl amine directing group (**1i**), competitive C‐N coupling pathways dominated, yielding **5ia** in 22% and **6aa** in 41% yield. In addition, anilines were also not efficient as a directing group, and *N*‐triflimides led to an intramolecular attack of the nitrogen to give 5‐membered heterocycles (see Supporting Information, Section D.5). It was clear at this point that the aliphatic OH group on the enyne was key in yielding the desired allene, acting as a native directing group.

Having identified hex‐5‐en‐3‐yn‐1‐ol (**1a**) as the optimal enyne substrate, we proceeded to optimize the reaction conditions (Table 1). This optimization proved challenging, as most ligands that were successful in related diene and enyne transformations failed to provide satisfactory results. Notably, attempts to achieve enantiocontrol using various chiral ligands resulted in low to no enantioinduction (see Supporting Information, Section C). Using SPhos Pd G3 as the Pd source, SPhos as ligand, and 2.5 equivalents of DIPEA at 70°C for 4 h, afforded the desired allene **4aaa** in 76% yield with only 4% of the C–O coupling side product **5aa** (entry 1). Lowering the temperature to 65°C and extending the reaction time to 16 h with 1.5 equivalents of DIPEA resulted in a decreased yield of 56% and increased side product formation (9% of **5aa**, entry 2). Maintaining 65°C but shortening the reaction time to 4 h with 1.5 equivalents of DIPEA improved the yield to 70% with 6% of **5aa** (entry 3). Heating to 70°C for 4 h with 1.5 equivalents of DIPEA afforded **4aaa** in 72% yield with only trace amounts of **5aa** (entry 4), demonstrating that increasing the base equivalents was key to minimizing side product formation. Several control experiments were then conducted: without extra SPhos ligand, the yield of **4aaa** dropped to 56% (entry 5). In the absence of Pd or base, no product was detected (entry 6). In the presence of air, no desired product was observed (entry 7). In contrast, using a non‐dry solvent did not affect the yield (entry 8).

**TABLE 1:** Optimization of the reaction conditions.

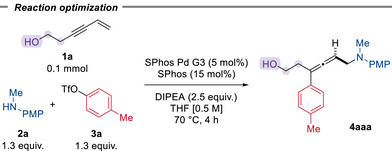			
Entry	Deviation from standard conditions	4aaa (%)^a^	5aa (%)
1	None	76	4
2	At 65°C, 16 h, with 1.5 equiv. of DIPEA	56	9
3	At 65°C, with 1.5 equiv. of DIPEA	70	6
4	With 1.5 equiv. of DIPEA	72	Trace
5	Without SPhos	56	Trace
6	Without Pd or base	nd	nd
7	Under air	nd	nd
8	With non‐dry solvent	76	Trace

^a^ ^1^H NMR yields were determined using TCE as an internal standard.

**SCHEME 3:**
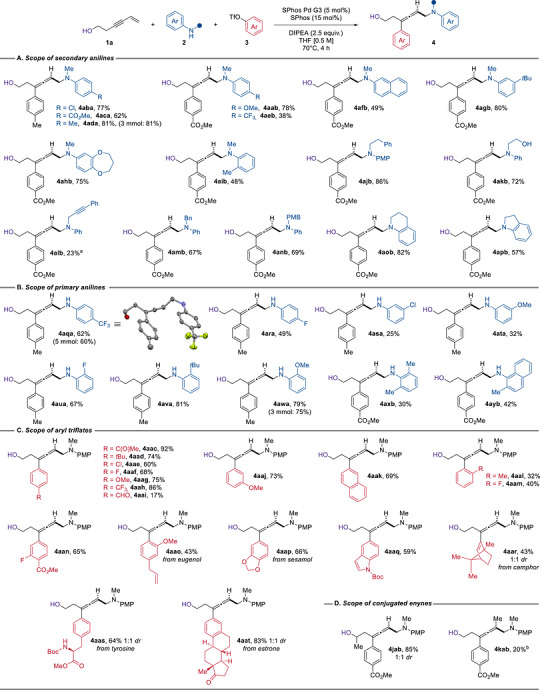
Substrate scope. Reaction conditions: **1** (1.0 equiv.), **2** (1.3 equiv.), **3** (1.3 equiv.), THF (0.5 M), 70°C, 4 h. ^a^8 h. ^b^16 h.

With optimized conditions in hand, we examined the scope of the carboamination, beginning with the amine nucleophile (Scheme 3A). Various substituents at the *para* position of the secondary aniline were compatible, including chloro (**4aba**), ester (**4aca**), and methyl group (**4ada**). The latter reaction was scaled up to 3 mmol, providing **4ada** in 81% yield. Using *p*‐CO_2_Me aryl triflate **3b**, electron‐donating (*p*‐OMe, **4aab**) and electron‐withdrawing groups (*p*‐CF_3_, **4aeb**) were tolerated. The 2‐naphthyl substituted **4afb** was obtained in 49% yield. *Meta*‐substitution was well tolerated, with *m*‐*t*Bu‐ and benzodioxepine‐substituted **4agb** and **4ahb** obtained in 80% and 75% yields. *Ortho*‐Me substitution afforded **4aib** in 48% yield. Alkyl chains on the nitrogen, a terminal phenyl group (**4ajb**, 86% yield), or a terminal unprotected alcohol (**4akb**, 72% yield) were tolerated. With an alkyne, **4alb** was obtained in 23% yield. Protecting groups on the nitrogen, including benzyl or *p*‐methoxybenzyl (PMB), also afforded the desired allenes **4amb** and **4anb** in 67% and 69% yields. Heterocyclic anilines gave **4aob** and **4apb** in 82% and 57% yields.

Using primary 4‐(trifluoromethyl)aniline as a nucleophile afforded the desired allene **4aqa** in 62% yield (Scheme 3B). Careful control of reaction time was crucial to prevent a second carboamination leading to 3‐pyrrolines. After 4 h, complete conversion of the conjugated enyne was observed with selective formation of the allene product. A crystal structure was obtained for compound **4aqa** [60]. Scaling up the reaction to 5 mmol proceeded smoothly. *Para*‐fluorinated product **4ara** was obtained in 49% yield, while *meta*‐substituted aryl amines provided allenes **4asa** (*m*‐Cl) and **4ata** (*m*‐OMe) in 25% and 32% yields. Various substituents were introduced at the ortho position of the aniline, including fluoro (**4aua**), *tert*‐butyl (**4ava**), and methoxy (**4awa**), with yields of 67%, 81%, and 79%, respectively. Scaling up to 3 mmol of **4awa** proceeded smoothly, providing a 75% yield. 2,6‐Dimethylaniline and 2‐methylnaphthalen‐1‐amine afforded products **4axb** and **4ayb** in 30% and 42% yields. Unsuccessful amine substrates included dialkyl and diaryl amines, carbamates, and sulfonamides (see Supporting Information, Section D5).

We then examined the scope of aryl triflates (Scheme 3C). Methyl ketone‐substituted product **4aac** was obtained in 92% yield, while a *tert*‐butyl substituent provided **4aad** in 74% yield. Halogen substituents, including chloro and fluoro, afforded products **4aae** and **4aaf** in 60% and 68% yield. No clear trend was observed based on electronic characteristics: product **4aag** bearing a *p*‐OMe group was obtained in 75% yield, while with a *p*‐CF_3_ group **4aah** was formed in 86% yield. An aldehyde yielded 17% of **4aai**. *Meta*‐substituted **4aaj** and **4aak** were obtained in 73% and 69% yields. *Ortho*‐substitution was detrimental, as **4aal** and **4aam** were isolated in only 32% and 40% yields. A bis‐substituted aryl triflate was successfully introduced in 65% yield (**4aan**), while a eugenol derivative also provided the desired product **4aao**. A sesamol derivative afforded allene **4aap** in 66% yield, and incorporation of a protected indole yielded **4aaq** in 59% yield. An alkenyl triflate derived from camphor provided **4aar** in 43% yield and 1:1 *dr*. Other alkenyl triflates were unsuccessful due to dominating C–O coupling. Functionalization of tyrosine proceeded in 64% yield (**4aas**), while an estrone derivative was obtained in 83% yield (**4aat**).

Various conjugated enynes were examined (Scheme 3D). A methyl group α to the alcohol provided **4jab** in 85% yield with 1:1 *dr*. A methyl substituent at the 2‐position afforded **4kab** in 20% yield, while substituents at the 1‐position were not tolerated.

**SCHEME 4:**
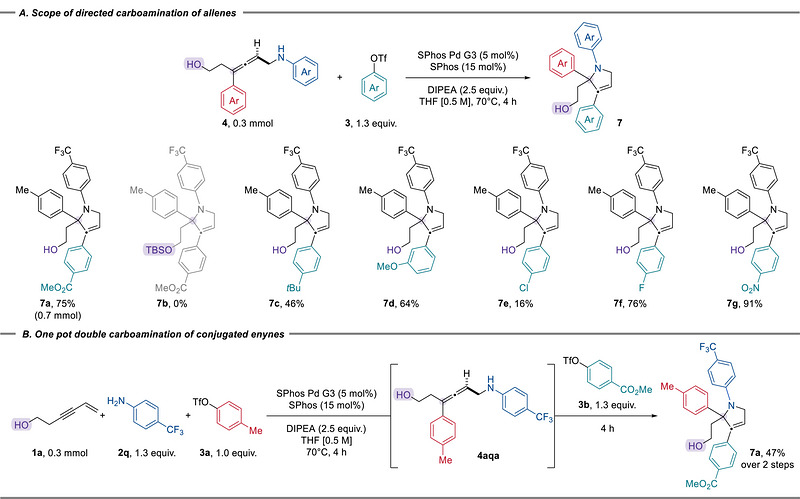
Sequential carboaminations for 3‐pyrroline synthesis. (A) Substrate scope. (B) One‐pot dual C‐N and C‐C bond formation strategy.

Primary anilines enabled a subsequent carboamination process utilizing the newly formed internal nucleophile (Scheme 4A). The transformation proceeded smoothly to afford **7a** in 75% yield. The free alcohol directing group remained crucial for this second carboamination event, as a protected alcohol failed to provide any conversion (**7b**). Other substituents were successfully introduced, including *para*‐*tert*‐butyl‐benzene (**7c**), and *ortho*‐OMe‐benzene (**7d**). Halogens were also tolerated, including chloride (**7e**, 16% yield) and fluoride (**7f**, 76% yield). A nitrobenzene group was introduced in 91% yield (**7** **g**). This transformation was also conducted as a one‐pot process to introduce two different aryl triflates and generate sequentially two C‐N bonds, affording 3‐pyrroline **7a** in 47% yield (Scheme 4B).

**SCHEME 5:**
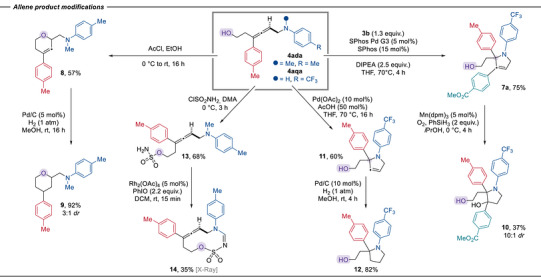
Modification of allene and 3‐pyrroline products.

To explore the synthetic utility, synthetic modifications were examined in addition to the carboamination (Scheme 5). Acid‐mediated cyclization of **4ada** proceeded smoothly to afford dihydropyran derivative **8** in 57% yield, effectively utilizing the alcohol as an internal nucleophile. Hydrogenation of **8** delivered the fully saturated 2,4‐substituted pyran **9** in 92% yield with a 3:1 *dr*. After a second carboamination reaction to generate 3‐pyrroline **7a**, pyrrolidine derivative **10** was obtained in 37% yield using a Mn‐catalyzed Mukaiyama hydration [61]. A palladium‐catalyzed intramolecular hydroamination enabled the synthesis of 3‐pyrroline **11** in 60% yield, which upon hydrogenation furnished α,α‐disubstituted pyrrolidine **12** in 82% yield. Finally, following a report by Schomaker [62], the homoallenic sulfamate **13** was engaged with Rh_2_(OAc)_4_ under oxidizing conditions to examine the reactivity with a nitrene intermediate. Rather than an aziridine, the 11‐membered macrocycle **14** was obtained in 35% yield, formed most probably via nitrene insertion and oxidation.

**SCHEME 6:**
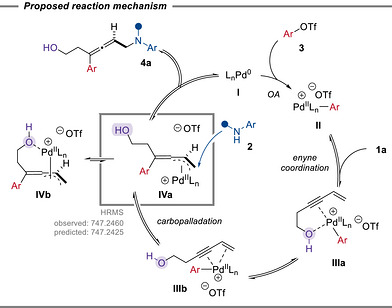
Proposed reaction mechanism.

Based on our experimental observations and literature precedents, a plausible Pd(0)/Pd(II) catalytic cycle is proposed in Scheme 6. First, oxidative addition of the aryl triflate **3** to Pd(0) complex **I** would afford oxidative addition complex **II**. Aryl bromides or iodides were not effective in the reaction and attempts to employ them in combination with AgOTf were unsuccessful, showing no conversion of the enyne. Coordination to enyne **1a** potentially assisted by the alcohol, would provide intermediate **IIIa**. Subsequently, carbopalladation may occur in a manner analogous to that reported by Huang and co‐workers [30] following decoordination (**IIIb**) and migratory insertion to generate an alkylene‐π‐allylpalladium intermediate (**IVa**, only one of the two possible enantiomers is drawn). Decoordination of the alcohol would be needed to allow insertion of the alkyne in the palladium‐aryl bond. Depending on the rate of nucleophile addition, isomerization to the potentially more stable π‐allyl intermediate **IVb** may be possible. Analysis of the reaction mixture by HRMS provided evidence of a species with a composition consistent with the proposed alkylene π‐allylpalladium intermediate **IV**. Finally, reductive nucleophilic addition of the amine would lead to allene **4a** and Pd(0) intermediate **I**. An outer‐sphere S_N_2‐like nucleophilic attack is tentatively proposed for this step, based on reports on Pd‐catalyzed allylic amination of anilines, although an inner sphere pathway cannot be excluded at this stage [63, 64]. The precise role of the alcohol in the catalytic cycle remains difficult to ascertain. It may facilitate initial coordination, formation of a stabilized π‐allyl palladacycle (**IVb**), or even interaction with the incoming nucleophile.

In conclusion, we have developed the first palladium‐catalyzed 1,4‐carboamination of conjugated enynes to synthesize highly functionalized allenic amines. This transformation proceeds through selective coupling of anilines with enynes and aryl triflates, with a free alcohol serving as a directing group and displays a broad substrate scope and excellent functional group compatibility. A key feature of this methodology is the ability to perform sequential transformations with primary anilines, wherein the initially formed allene intermediate undergoes a second carboamination event to generate multifunctionalized 3‐pyrroline heterocycles through dual C–N bond formation. The synthetic utility of the obtained allenes has been further demonstrated through their diversification into various heterocyclic frameworks, including pyrans and pyrrolidines.

## Conflicts of Interest

Th authors declare no conflicts of interest.

## Supporting information



General methods, experimental procedures, characterization data, and NMR spectra for new compounds. Raw NMR, MS, and IR is available at zenodo.org: https://doi.org/105281/zenodo.18851416.
**Supporting File**: anie72348‐sup‐0001‐SuppMat.pdf.

## Data Availability

The data that supports the findings of this study are available in the supplementary material of this article.
