# Supplementary material for: Alcohol‐Directed Carboamination of Conjugated Enynes
Source: Angew Chem Int Ed Engl. 2026 May 2;65(24):e6963888. doi: 10.1002/anie.6963888 (PMC13245598; doi:10.1002/anie.6963888)

Supporting Information for

"Alcohol Directed Carboamination of Conjugated Enynes"

Helena Solé-Àvila, Duncan K. Brownsey, Hugo Senelle and Jerome Waser*

Laboratory of Catalysis and Organic Synthesis, Ecole Polytechnique Fédérale de Lausanne, EPFL, SB ISIC LCSO, BCH 4306, 1015 Lausanne (Switzerland)

*Correspondence to: jerome.waser@epfl.ch

Table of Contents

A. General Information ... 3
B. Synthesis of starting materials ... 4
B.1 Synthesis of aryl triflates ... 4
B.2. Synthesis of enynes ... 8
B.3. Synthesis of amines ... 13
C. Ligand screening ... 15
D. Procedures and product characterization data of carboamination products ... 15
D.1. General Procedure D1 for the synthesis of allenes ... 15
D.2. Characterization of the allene products ... 16
D.3. General Procedure D3 for the synthesis of 3-pyrrolines ... 31
D.4. Characterization of the 3-pyrroline products ... 31
D.5. Unsuccessful substrates ... 33
E. Product modifications ... 34
E.1. Alcohol cyclization ... 34
E.2. Mukaiyama hydration ... 35
E.3. Hydroamination and hydrogenation ... 35
E.4. Macrocyclization ... 36
F. Mechanistic studies ... 37
G. X-ray crystallography ... 38
H. References ... 47
I. NMR Spectra ... 56
I.1. Spectra of starting materials ... 56
I.2. Spectra of allenes ... 62
I.3. Spectra of 3-pyrrolines ... 115
I.4. Spectra of product modifications ... 123

A. General Information

The NMR spectra were recorded on a Brucker DPX-400 spectrometer at 400 MHz for ^{1}H, 101 MHz for ^{13}C, 376 MHz for ^{19}F. The chemical shift (δ) for ^{1}H and ^{13}C are given in ppm relative to residual signals of the solvents ($CDCl_3$ - 7.26 ppm ^{1}H NMR and 77.16 ppm ^{13}C NMR). Carbon spectra have been measured using broadband {^{1}H} decoupling. Coupling constants are given in Hertz. The following abbreviations are used to indicate the multiplicity: s, singlet; d, doublet; q, quartet; m, multiplet; bs, broad signal; app, apparent. Infrared spectra were recorded on a JASCO FT-IR B4100 spectrophotometer with an ATR PRO410-S and a ZnSe prisma and are reported as cm^{-1} (w = weak, m = medium, s = strong, br = broad). High resolution mass spectrometric measurements were performed by the mass spectrometry service of ISIC at the EPFL on a MICROMASS (ESI) Q-TOF Ultima API. The raw data obtained from the Q-TOF Waters instrument does not take into account the mass of the electron for the ion, the obtained raw data has been therefore corrected by removing the mass of the electron (5 mDa). The diffraction data for crystal structures were collected by mass spectrometry service of ISIC at the EPFL at low temperature using Cu (323) or Mo (520) K_a radiation on a Rigaku SuperNova dual system in combination with Atlas type CCD detector. The data reduction and correction were carried out by *CrysAlis^{Pro}* (Rigaku Oxford Diffraction, release 1.171.40.68a, **2019**). The solutions and refinements were performed by *SHELXT*[1] and *SHELXL*[2], respectively. The crystal structures were refined using full-matrix least-squares based on F^2 with all non-H atoms defined in anisotropic manner. Hydrogen atoms were placed in calculated positions by means of the "riding" model. Yields of isolated products refer to materials of >95% purity as determined by ^{1}H NMR.

The authors are indebted to the team of the research support service of ISIC at EPFL, particularly to the NMR, X-Ray, and the High Resolution Mass Spectrometry Units.

General Procedures. All reactions were set up under a nitrogen atmosphere in oven-dried glassware using standard Schlenk techniques, unless otherwise stated. Synthesis grade solvents were used as purchased; anhydrous solvents (THF, Et_2O, Toluene and DCM) were taken from a commercial SPS solvent dispenser (H_2O content < 10 ppm, *Karl-Fischer* titration). Chromatographic purification of products was accomplished using flash chromatography (FC) on SiliaFlash P60 silica gel (230 - 400 mesh) or using Biotage Isolera Spektra One with pre-packaged silica cartridges purchased from Buchi, models: Sepacore or GraceResolve (4 g, 12 g, 25 g, 40 g, 80 g, 120 g). For thin layer chromatography (TLC) analysis throughout this work, Merck silica gel 60 F254 TLC glass plates were employed, using UV light as the visualizing agent and basic aqueous potassium permanganate ($KMnO_4$) stain solutions, and heat as developing agents. Organic solutions were concentrated under reduced pressure on a Büchi rotatory evaporator.

Materials. Most of the starting materials used in this study are commercial and were purchased in the highest purity available from Sigma-Aldrich, Fluka, Alfa Aesar, Fluorochem, Enamine and used as received, without further purifications.

B. Synthesis of starting materials

B.1 Synthesis of aryl triflates

Commercially available aryl triflates:

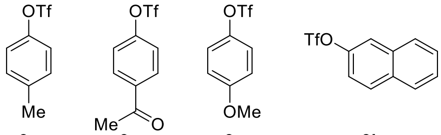

3a 3c 3g 3k

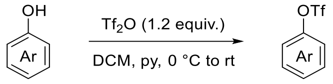

S1 3

General Procedure B1: According to a reported procedure,[1] a round bottom flask was charged with phenol derivative **S1** (1.0 equiv.), dichloromethane [0.6 M] and pyridine (2.0 equiv.). The reaction mixture was cooled to 0 °C and trifluoromethanesulfonic anhydride (1.2 equiv.) was added dropwise. The reaction mixture was stirred for 4 h before it was quenched with an aqueous saturated solution of NH_4Cl. The aqueous layer was extracted with ethyl acetate (3 × 15 mL). The combined organic layers were washed with water (15 mL) and brine (15 mL) and dried over Na_2SO_4. The solvent was removed under vacuum, and the residue was purified by column chromatography.

Methyl 4-(((trifluoromethyl)sulfonyl)oxy)benzoate (3b)

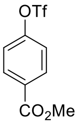

3b

Prepared according to the general procedure B1 using methylparaben (4.56 g, 30.0 mmol, 1.00 equiv.). The crude material was purified by column chromatography (20 % (v/v) EtOAc in hexane) to give **3b** (8.25 g, 29.0 mmol, 97% yield) as a colorless oil. Spectral data was consistent with the values reported in literature.[1]

^{1}H NMR (400 MHz, $CDCl_3$) δ 8.18 – 8.10 (m, 2H, Ar*H*), 7.38 – 7.33 (m, 2H, Ar*H*), 3.94 (s, 3H, CO_2CH_3).

^{13}C{^{1}H} NMR (101 MHz, $CDCl_3$) δ 165.6, 152.6, 132.1, 130.5, 121.6, 118.8 (q, J_{C-F} = 320.8 Hz), 52.7.

^{19}F NMR (376 MHz, $CDCl_3$) δ -72.8.

4-(*tert*-butyl)Phenyl trifluoromethanesulfonate (3d)

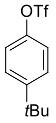

3d

Prepared according to the general procedure B1 using 4-(*tert*-butyl)phenol (1.502 g, 10.00 mmol, 1.000 equiv.). The crude material was purified by column chromatography (0 – 1% (v/v) EtOAc in hexanes) to give **3d** (1.625 g, 5.756 mmol, 58% yield) as a colorless oil. Spectral data was consistent with the values reported in literature.[2]

^{1}H NMR (400 MHz, $CDCl_3$) δ 7.48 – 7.41 (m, 2H, Ar*H*), 7.22 – 7.16 (m, 2H, Ar*H*), 1.33 (s, 9H, $C(CH_3)_3$).

^{13}C{^{1}H} NMR (101 MHz, $CDCl_3$) δ 151.8, 147.6, 127.3, 120.8, 118.9 (q, J_{C-F} = 320.8 Hz), 34.9, 31.4.

^{19}F NMR (376 MHz, $CDCl_3$) δ -72.9.

4-Chlorophenyl trifluoromethanesulfonate (3e)

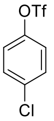

3e

Prepared according to the general procedure B1 using 4-chlorophenol (19.28 mg, 15.00 mmol, 1.000 equiv.). The crude material was purified by column chromatography (1 – 2 % (v/v) EtOAc in hexanes) to give **3e** (2.947 g, 11.31 mmol, 75% yield) as a colorless oil. Spectral data was consistent with the values reported in literature.[3]

^{1}H NMR (400 MHz, $CDCl_3$) δ 7.46 – 7.40 (m, 2H, Ar*H*), 7.25 – 7.20 (m, 2H, Ar*H*).

^{13}C{^{1}H} NMR (101 MHz, $CDCl_3$) δ 148.0, 134.5, 130.6, 122.9, 118.9 (q, J_{C-F} = 320.8 Hz).

^{19}F NMR (376 MHz, $CDCl_3$) δ -72.7.

4-Fluorophenyl trifluoromethanesulfonate (3f)

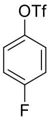

3f

Prepared according to the general procedure B1 using 4-fluorophenol (1.286 g, 10.00 mmol, 1.000 equiv.). The crude material was purified by column chromatography (1 – 2% (v/v) EtOAc in hexanes) to give **3f** (2.068 g, 8.469 mmol, 85% yield) as a colorless oil. Spectral data was consistent with the values reported in literature.[4]

^{1}H NMR (400 MHz, $CDCl_3$) δ 7.33 – 7.27 (m, 2H, Ar*H*), 7.21 – 7.13 (m, 2H, Ar*H*).

^{13}C{^{1}H} NMR (101 MHz, $CDCl_3$) δ 161.8 (d, J_{C-F} = 248.9 Hz), 145.4 (d, J_{C-F} = 3.1 Hz), 123.3 (d, J_{C-F} = 8.9 Hz), 118.9 (q, J_{C-F} = 321.1 Hz) 117.3 (d, J_{C-F} = 24.2 Hz).

^{19}F NMR (376 MHz, $CDCl_3$) δ -72.7, -112.3.

4-(Trifluoromethyl)phenyl trifluoromethanesulfonate (3h)

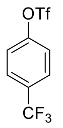

3h

Prepared according to the general procedure B1 using 4-(trifluoromethyl)phenol (612 mg, 3.78 mmol, 1.00 equiv.). The crude material was purified by column chromatography (10 % (v/v) EtOAc in pentane) to give **3h** (650 mg, 2.21 mmol, 59% yield) as a colorless oil. Spectral data was consistent with the values reported in literature.[1]

^{1}H NMR (400 MHz, $CDCl_3$) δ 7.75 (d, J = 8.7 Hz, 2H, ArH), 7.42 (d, J = 8.7 Hz, 2H, ArH).

^{13}C{^{1}H} NMR (101 MHz, $CDCl_3$) δ 151.7, 131.1 (q, J_{C-F} = 33.3 Hz), 127.9 (q, J_{C-F} = 3.6 Hz), 123.4 (q, J_{C-F} = 270.6 Hz), 122.2, 118.9 (q, J_{C-F} = 318.8 Hz).

^{19}F NMR (376 MHz, $CDCl_3$) δ -62.7, -72.7.

4-Formylphenyl trifluoromethanesulfonate (3i)

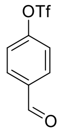

3i

Prepared according to the general procedure B1 using 4-hydroxybenzaldehyde (1.221 g, 10.00 mmol, 1.000 equiv.). The crude material was purified by column chromatography (1 – 15% (v/v) Et_2O in hexanes) to give **3i** (1.029 g, 4.049 mmol, 40% yield) as a colorless oil. Spectral data was consistent with the values reported in literature.[2]

^{1}H NMR (400 MHz, $CDCl_3$) δ 10.05 (s, 1H, CHO), 8.06 – 7.90 (m, 2H, ArH), 7.51 – 7.42 (m, 2H, ArH).

^{13}C{^{1}H} NMR (101 MHz, $CDCl_3$) δ 190.2, 153.4, 136.1, 131.9, 122.4, 118.8 (q, J_{C-F} = 320.8 Hz).

^{19}F NMR (376 MHz, $CDCl_3$) δ -72.7.

3-Methoxyphenyl trifluoromethanesulfonate (3j)

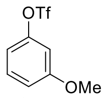

3j

Prepared according to the general procedure B1 using 3-methoxyphenol (1.10 mL, 10.0 mmol, 1.00 equiv.). The crude material was purified by column chromatography (1 – 2% (v/v) EtOAc in hexanes) to give **3j** (2.23 g, 8.70 mmol, 87% yield) as a colorless oil. Spectral data was consistent with the values reported in literature.[3]

^{1}H NMR (400 MHz, $CDCl_3$) δ 7.34 (t, J = 8.3 Hz, 1H, ArH), 6.93 (ddd, J = 8.5, 2.4, 0.8 Hz, 1H, ArH), 6.87 (ddd, J = 8.2, 2.4, 0.8 Hz, 1H, ArH), 6.80 (t, J = 2.4 Hz, 1H, ArH), 3.83 (s, 3H, ArOCH_3).

^{13}C{^{1}H} NMR (101 MHz, $CDCl_3$) δ 161.0, 150.4, 130.7, 118.9 (q, J_{C-F} = 320.8 Hz), 114.3, 113.4, 107.6, 55.8.

^{19}F NMR (376 MHz, $CDCl_3$) δ -72.9.

***o*-Tolyl trifluoromethanesulfonate (3l)**

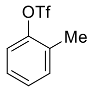

3l

Prepared according to the general procedure B1 using *o*-cresol (1.081 g, 10.00 mmol, 1.000 equiv.). The crude material was purified by column chromatography (0 – 1% (v/v) EtOAc in hexanes) to give **3l** (1.787 g, 7.439 mmol, 74% yield) as a colorless oil. Spectral data was consistent with the values reported in literature.[3]

^{1}H NMR (400 MHz, $CDCl_3$) δ 7.33 – 7.22 (m, 4H, ArH), 2.39 (s, 3H, ArCH_3).

^{13}C{^{1}H} NMR (101 MHz, $CDCl_3$) δ 148.7, 132.3, 131.0, 128.4, 127.8, 121.4, 118.8 (q, J_{C-F} = 320.1 Hz), 16.5.

^{19}F NMR (376 MHz, $CDCl_3$) δ -73.9.

2-Fluorophenyl trifluoromethanesulfonatee (3m)

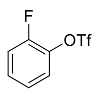

3m

Prepared according to the general procedure B1 using 2-fluorophenol (1.121 g, 15.00 mmol, 1.000 equiv.). The crude material was purified by column chromatography (1 – 5% (v/v) Et_2O in hexanes) to give **3m** (1.892 g, 7.749 mmol, 77% yield) as a colorless oil. Spectral data was consistent with the values reported in literature.[4]

^{1}H NMR (400 MHz, $CDCl_3$) δ 7.42 – 7.32 (m, 2H, ArH), 7.31 – 7.18 (m, 2H, ArH).

^{13}C{^{1}H} NMR (101 MHz, $CDCl_3$) δ 153.9 (d, J_{C-F} = 253.4 Hz), 137.1 (d, J_{C-F} = 13.4 Hz), 129.8 (d, J_{C-F} = 7.2 Hz), 125.2 (d, J_{C-F} = 4.1 Hz), 123.7, 118.9 (q, J_{C-F} = 320.8 Hz), 117.8 (d, J_{C-F} = 18.0 Hz).

^{19}F NMR (376 MHz, $CDCl_3$) δ -73.2 (d, J = 5.1 Hz), -126.4 – -128.4 (m).

Methyl 2-fluoro-4-(((trifluoromethyl)sulfonyl)oxy)benzoate (3n)

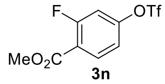

3n

Prepared according to the general procedure B1 using methyl 2-fluoro-4-hydroxybenzoate (0.851 g, 5.00 mmol, 1.00 equiv.). The crude material was purified by column chromatography (1 – 3% (v/v) Et_2O in hexanes) to give **3n** (1.34 g, 4.44 mmol, 89% yield) as a colorless oil. Spectral data was consistent with the values reported in literature.[5]

^{1}H NMR (400 MHz, $CDCl_3$) δ 8.12 – 7.96 (m, 1H, Ar*H*), 7.24 – 7.07 (m, 2H, Ar*H*), 3.96 (s, 3H, CO_2CH_3).

^{13}C{^{1}H} NMR (101 MHz, $CDCl_3$) δ 163.5 (d, J_{C-F} = 1.5 Hz), 162.2 (d, J_{C-F} = 271.1 Hz), 152.4 (d, J_{C-F} = 10.7 Hz), 134.1 (d, J_{C-F} = 2.2 Hz), 119.3 (d, J_{C-F} = 10.2 Hz), 118.8 (q, J_{C-F} = 321.0 Hz), 117.4 (d, J_{C-F} = 4.2 Hz), 111.3 (d, J_{C-F} = 26.9 Hz), 52.9.

^{19}F NMR (376 MHz, $CDCl_3$) δ -72.6, -103.0 (dd, J = 10.0, 7.9 Hz).

4-Allyl-2-methoxyphenyl trifluoromethanesulfonate (3o)

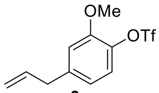

3o

Prepared according to the general procedure B1 using eugenol (0.821 g, 5.00 mmol, 1.00 equiv.). The crude material was purified by column chromatography (1 – 3% (v/v) Et_2O in hexanes) to give **3o** (1.25 g, 4.23 mmol, 85% yield) as a colorless oil. Spectral data was consistent with the values reported in literature.[6]

^{1}H NMR (400 MHz, $CDCl_3$) δ 7.13 (d, J = 8.3 Hz, 1H, Ar*H*), 6.85 (d, J = 2.0 Hz, 1H, Ar*H*), 6.79 (dd, J = 8.3, 2.0 Hz, 1H, Ar*H*), 6.02 – 5.85 (m, 1H, C=C*H*), 5.14 (t, J = 1.4 Hz, 1H, C=C*H*), 5.11 (dq, J = 7.8, 1.5 Hz, 1H, C=C*H*), 3.90 (s, 3H, ArOCH_3), 3.40 (dd, J = 6.8, 1.5 Hz, 2H, ArCH_2).

^{13}C{^{1}H} NMR (101 MHz, $CDCl_3$) δ 151.3, 141.9, 137.3, 136.4, 122.3, 120.9, 118.9 (q, J_{C-F} = 320.4 Hz), 117.0, 113.5, 56.2, 40.2.

^{19}F NMR (376 MHz, $CDCl_3$) δ -73.9.

Benzo[*d*][1,3]dioxol-5-yl trifluoromethanesulfonate (3p)

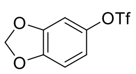

3p

Prepared according to the general procedure B1 using sesamol (0.691 g, 5.00 mmol, 1.00 equiv.). The crude material was purified by column chromatography (1 – 3% (v/v) Et_2O in hexanes) to give **3p** (0.988 g, 3.66 mmol, 73% yield) as a colorless oil. Spectral data was consistent with the values reported in literature.[2]

^{1}H NMR (400 MHz, $CDCl_3$) δ 6.92 – 6.63 (m, 3H, Ar*H*), 6.05 (s, 2H, ArOCH_2O).

^{13}C{^{1}H} NMR (101 MHz, $CDCl_3$) δ 148.7, 147.6, 143.6, 118.9 (q, J_{C-F} = 321.0 Hz), 114.6, 108.4, 103.5, 102.6.

^{19}F NMR (376 MHz, $CDCl_3$) δ -72.7.

***tert*-Butyl 5-(((trifluoromethyl)sulfonyl)oxy)-1*H*-indole-1-carboxylate (3q)**

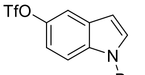

3q

Starting from 5-hydroxyindole (1.331 g, 10.00 mmol, 1.000 equiv.) following general procedure B1. The crude material was purified by column chromatography (10 – 30% (v/v) EtOAc in hexanes) to give intermediate indole **S2** (0.984 g, 3.71 mmol, 37% yield) as a brown oil, which was used immediately in the next step.

R_f = 0.39 (20% EtOAc in pentane).

To a solution of **S2** (0.530 g, 2.00 mmol, 1.00 equiv.) in anhydrous THF (20 mL) was added DMAP (24 mg, 0.20 mmol, 0.10 equiv.), followed by a solution of Boc anhydride (0.480 g, 2.20 mmol, 1.10 equiv.) in anhydrous THF (10 mL) and the reaction was stirred for 16 h at room temperature. The reaction was then concentrated *in vacuo*, and the resulting crude residue was purified by column chromatography (1 – 5% (v/v) EtOAc in hexanes) to give **3q** (0.692 g, 1.89 mmol, 95% yield) as a pale yellow oil. Spectral data was consistent with the values reported in literature.[2]

^{1}H NMR (400 MHz, $CDCl_3$) δ 8.21 (d, J = 9.1 Hz, 1H, Ar*H*), 7.70 (d, J = 3.7 Hz, 1H, Ar*H*), 7.48 (d, J = 2.5 Hz, 1H, Ar*H*), 7.21 (dd, J = 9.1, 2.5 Hz, 1H, Ar*H*), 6.60 (dd, J = 3.8, 0.8 Hz, 1H, Ar*H*), 1.68 (s, 9H, C(CH_3)$_3$).

^{13}C{^{1}H} NMR (101 MHz, $CDCl_3$) δ 149.4, 145.4, 134.3, 131.4, 128.4, 119.0 (d, J_{C-F} = 320.8 Hz), 117.2, 116.5, 113.6, 107.2, 84.7, 28.3.

^{19}F NMR (376 MHz, $CDCl_3$) δ -72.7.

(1*S*,4*R*)-1,7,7-Trimethylbicyclo[2.2.1]hept-2-en-2-yl trifluoromethanesulfonate (3r)

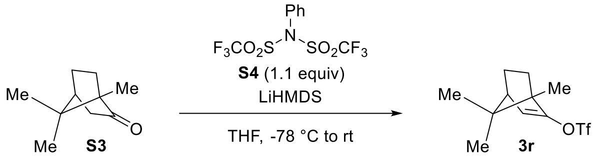

To a cooled solution (-78 °C) of camphor **S3** (1.522 g, 10.00 mmol, 1.000 equiv.) and *N*-phenyl-bis(trifluoromethanesulfonamide) **S4** (3.390 g, 11.00 mmol, 1.100 equiv.) in THF (60 mL) was slowly added a 1.0 M solution of LiHMDS in THF (11.0 mL, 1.10 equiv.) dropwise over 20 minutes. After slowly warming up the solution to room temperature the solution was stirred for 16 hours. The mixture was then concentrated *in vacuo* and the crude material was purified by column chromatography (1 – 3% (v/v) Et_2O in pentane) to afford **3r** (1.766 g, 3.211 mmol, 60%) as a colourless oil. Spectral data was consistent with the values reported in literature.[7]

^{1}H NMR (400 MHz, $CDCl_3$) δ 5.67 (d, J = 3.8 Hz, 1H, C=CH), 2.45 (t, J = 3.7 Hz, 1H, CH), 1.93 (ddt, J = 12.2, 8.6, 3.7 Hz, 1H, CH_2), 1.65 (ddd, J = 12.1, 8.5, 3.6 Hz, 1H, CH_2), 1.33 (ddd, J = 12.4, 9.1, 3.6 Hz, 1H, CH_2), 1.15 (ddd, J = 12.5, 9.1, 3.7 Hz, 1H, CH_2), 1.03 (s, 3H, CH_3), 0.92 (s, 3H, CH_3), 0.79 (s, 3H, CH_3).

^{13}C{^{1}H} NMR (101 MHz, $CDCl_3$) δ 155.2, 118.6 (q, $J_{C\text{-}F}$ = 320.6 Hz), 117.7, 57.0, 53.8, 50.1, 30.8, 25.3, 19.7, 19.0, 9.5.

^{19}F NMR (376 MHz, $CDCl_3$) δ -73.7.

Methyl (*S*)-2-((*tert*-butoxycarbonyl)amino)-3-(4-(((trifluoromethyl)sulfonyl)oxy)phenyl)propanoate (3s)

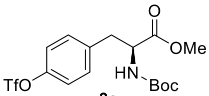

3s

Prepared according to the general procedure B1 using *N*-(*tert*-butoxycarbonyl)-L-tyrosine methyl ester (1.477 g, 5.000 mmol, 1.000 equiv.). The crude material was purified by column chromatography (15 – 50% (v/v) EtOAc in hexanes) to give **3s** (1.234 g, 2.887 mmol, 58% yield) as a colorless viscous oil. Spectral data was consistent with the values reported in literature.[8]

^{1}H NMR (400 MHz, $CDCl_3$) δ 7.23 – 7.14 (m, 4H, ArH), 5.11 (d, J = 7.9 Hz, 1H, NH), 4.76 (dt, J = 7.9, 5.9 Hz, 1H, NCH), 3.70 (s, 3H, CO_2CH_3), 3.17 – 2.95 (m, 2H, ArCH_2).

118.9 (q, $J_{C\text{-}F}$ = 320.8 Hz)

^{13}C{^{1}H} NMR (101 MHz, $CDCl_3$) δ 172.0, 155.1, 148.8, 137.1, 131.3, 121.5, 118.9 (q, $J_{C\text{-}F}$ = 320.8 Hz), 80.4, 54.4, 52.5, 38.1, 28.4.

^{19}F NMR (376 MHz, $CDCl_3$) δ -72.9.

(8*R*,9*S*,13*S*,14*S*)-13-Methyl-17-oxo-7,8,9,11,12,13,14,15,16,17-decahydro-6*H*-cyclopenta[a]phenanthren-3-yl trifluoromethanesulfonate (3t)

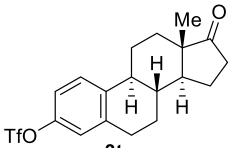

3t

Prepared according to a modified version of general procedure B1 using estrone (1.352 g, 5.000 mmol, 1.000 equiv.) and triethylamine (2.09 mL, 15.0 mmol, 3.00 equiv.) in lieu of pyridine. The crude material was purified by column chromatography (5 – 20% (v/v) Et_2O in hexanes) to give **3t** (1.214 g, 3.02 mmol, 60% yield) as a colorless oil. Spectral data was consistent with the values reported in literature.[9]

^{1}H NMR (400 MHz, $CDCl_3$) δ 7.34 (d, J = 8.6 Hz, 1H, ArH), 7.04 (dd, J = 8.6, 2.8 Hz, 1H, ArH), 6.99 (d, J = 2.7 Hz, 1H, ArH), 2.94 (dd, J = 9.0, 4.3 Hz, 2H, ArCH_2), 2.58 – 2.47 (m, 1H, CH), 2.45 – 2.35 (m, 1H, CH), 2.35 – 2.23 (m, 1H, CH), 2.22 – 1.93 (m, 4H, CH), 1.71 – 1.59 (m, 2H, CH), 1.56 – 1.40 (m, 4H, CH), 0.92 (s, 3H, CH_3).

^{13}C{^{1}H} NMR (101 MHz, $CDCl_3$) δ 220.5, 147.7, 140.4, 139.4, 127.3, 121.4, 118.9 (q, $J_{C\text{-}F}$ = 320.7 Hz), 118.5, 50.5, 48.0, 44.2, 37.9, 35.9, 31.6, 29.5, 26.2, 25.8, 21.7, 13.9.

^{19}F NMR (376 MHz, $CDCl_3$) δ -73.0.

4-Acetamidophenyl trifluoromethanesulfonate (S5)

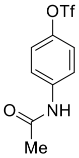

S5

Prepared according to the general procedure B1 using 4-acetaminophenol (1.512 g, 10.00 mmol, 1.000 equiv.). The crude material was purified by column chromatography (25 – 60% (v/v) EtOAc in hexanes) to give **S5** (0.461g, 1.63 mmol, 16% yield) as a tan solid. Spectral data was consistent with the values reported in literature.[10]

^{1}H NMR (400 MHz, $CDCl_3$) δ 7.65 – 7.55 (m, 2H, ArH), 7.35 (br s, 1H, NH), 7.25 – 7.18 (m, 2H, ArH), 2.20 (s, 3H, CH_3).

^{13}C{^{1}H} NMR (101 MHz, $CDCl_3$) δ 168.5, 145.5, 138.0, 122.1, 121.1, 118.9 (d, $J_{C\text{-}F}$ = 321.0 Hz), 24.7.

^{19}F NMR (376 MHz, $CDCl_3$) δ -72.7.

4-Cyanophenyl trifluoromethanesulfonate (S6)

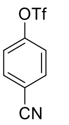

S6

Prepared according to the general procedure B1 using 4-hydroxybenzonitrile (1.191 g, 10.00 mmol, 1.000 equiv.). The crude material was purified by column chromatography (4 – 15% (v/v) Et_2O in hexanes) to give **S6** (1.887 g, 7.512 mmol, 75% yield) as a colorless oil. Spectral data was consistent with the values reported in literature.[3]

^{1}H NMR (400 MHz, $CDCl_3$) δ 7.84 – 7.74 (m, 2H, ArH), 7.47 – 7.37 (m, 2H, ArH).

^{13}C{^{1}H} NMR (101 MHz, $CDCl_3$) δ 152.1, 134.6, 122.8, 118.8 (q, $J_{C\text{-}F}$ = 321.0 Hz), 117.2, 113.1.

^{19}F NMR (376 MHz, $CDCl_3$) δ -72.6.

Naphthalen-1-yl trifluoromethanesulfonate (S7)

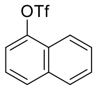

S7

Prepared according to the general procedure B1 using 1-naphthol (1.514 g, 10.50 mmol, 1.000 equiv.). The crude material was purified by column chromatography (0 – 1% (v/v) EtOAc in hexanes) to give **S7** (2.737 g, 9.908 mmol, 94% yield) as a pale yellow oil. Spectral data was consistent with the values reported in literature.[10]

^{1}H NMR (400 MHz, $CDCl_3$) δ 8.15 – 8.03 (m, 1H, Ar*H*), 7.90 (ddd, *J* = 16.6, 7.6, 1.9 Hz, 2H, Ar*H*), 7.71 – 7.56 (m, 2H, Ar*H*), 7.55 – 7.41 (m, 2H, Ar*H*).

^{13}C{^{1}H} NMR (101 MHz, $CDCl_3$) δ 145.8, 135.0, 128.7, 128.2, 128.0, 127.6, 126.5, 125.3, 120.9, 118.9 (q, J_{C-F} = 320.5 Hz), 117.9.

^{19}F NMR (376 MHz, $CDCl_3$) δ -73.4.

6-Methylpyridin-3-yl trifluoromethanesulfonate (S8)

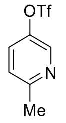

S8

Prepared according to the general procedure B1 using 1,3-benzothiazol-6-ol (1.09 g, 10.0 mmol, 1.00 equiv.). The crude material was purified by column chromatography (20 % (v/v) EtOAc in hexane) to give **S8** (2.0 g, 8.3 mmol, 83% yield) as a colorless oil. Spectral data was consistent with the values reported in literature.[11]

^{1}H NMR (400 MHz, $CDCl_3$) δ 8.48 (d, *J* = 2.7 Hz, 1H, Ar*H*), 7.53 (dd, *J* = 8.6, 2.8 Hz, 1H, Ar*H*), 7.30 – 7.27 (m, 1H, Ar*H*), 2.62 (s, 3H, CH_3).

^{13}C{^{1}H} NMR (101 MHz, $CDCl_3$) δ 159.2, 145.1, 142.0, 129.3, 124.4, 118.9 (q, J_{C-F} = 320.9 Hz), 24.1.

4-Nitrophenyl trifluoromethanesulfonate (S9)

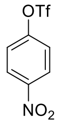

S9

Prepared according to the general procedure B1 using 4-nitrophenol (1.39 g, 10.0 mmol, 1.00 equiv.). The crude material was purified by column chromatography (10 % (v/v) EtOAc in pentane) to give **S9** (2.61 g, 9.63 mmol, 96% yield) as a colorless oil. Spectral data was consistent with the values reported in literature.[2]

^{1}H NMR (400 MHz, $CDCl_3$) δ 8.42 – 8.32 (m, 2H, Ar*H*), 7.54 – 7.44 (m, 2H, Ar*H*).

^{13}C{^{1}H} NMR (101 MHz, $CDCl_3$) δ 153.3, 147.3, 126.2, 122.7, 118.8 (q, J_{C-F} = 320.9 Hz).

^{19}F NMR (376 MHz, $CDCl_3$) δ -72.5.

B.2. Synthesis of enynes

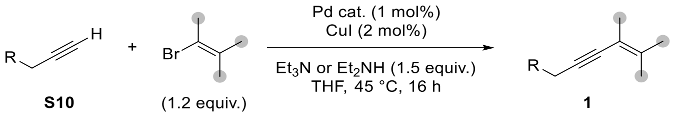

General Procedure B2: Pd(PPh_3)$_4$ (1 mol%) and CuI (2 mol%) were dissolved in triethylamine or diethylamine (1.5 equiv.) under N_2 at 0 °C. The alkyne (1.0 equiv.) was added, followed by the vinyl bromide (1.1 equiv. in THF) and the resulting mixture was stirred for 16 h at 45 °C or for 48 h at room temperature. Then, the reaction mixture was cooled to room temperature and filtered. After evaporation of the solvent under reduced pressure, the residue was purified by flash column chromatography on silica gel to afford the corresponding product.

Hex-5-en-3-yn-1-ol (1a)

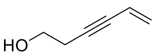

1a

Prepared according to the general procedure B2 using but-3-yn-1-ol (1.51 mL, 20.0 mmol, 1.00 equiv.) and vinyl bromide (22.0 mL, 22.0 mmol, 1.10 equiv., 2M in THF). The crude material was purified by column chromatography (10 – 30 % (v/v) EtOAc in hexane) to give **1a** (1.44 g, 15.0 mmol, 75% yield) as a pale-yellow oil. Spectral data was consistent with the values reported in literature.[12]

^{1}H NMR (400 MHz, $CDCl_3$) δ 5.78 (ddt, *J* = 17.5, 11.0, 2.1 Hz, 1H, C*H*=), 5.59 (dd, *J* = 17.5, 2.2 Hz, 1H, =CH_2), 5.43 (dd, *J* = 11.0, 2.2 Hz, 1H, =CH_2), 3.74 (t, *J* = 6.1 Hz, 2H, OCH_2), 2.59 (td, *J* = 6.2, 2.1 Hz, 2H, CCH_2), 1.82 (s, 1H, O*H*).

^{13}C{^{1}H} NMR (101 MHz, $CDCl_3$) δ 126.7, 117.3, 87.3, 81.3, 61.2, 23.9.

Hept-6-en-4-yn-1-ol (1b)

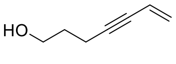

1b

Prepared according to the general procedure B2 using pent-4-yn-1-ol (1.86 mL, 20.0 mmol, 1.00 equiv.) and vinyl bromide (22.0 mL, 22.0 mmol, 1.10 equiv., 2M in THF). The crude material was purified by column chromatography (10 – 40 % (v/v) EtOAc in hexane) to give **1b** (1.93 g, 17.5 mmol, 80% yield) as a yellow oil. Spectral data was consistent with the values reported in literature.[13]

^{1}H NMR (400 MHz, $CDCl_3$) δ 5.77 (ddt, J = 17.5, 11.0, 2.1 Hz, 1H, CH=), 5.55 (dd, J = 17.5, 2.2 Hz, 1H, =CH_2), 5.39 (dd, J = 11.0, 2.2 Hz, 1H, =CH_2), 3.76 (q, J = 6.0 Hz, 2H, OCH_2), 2.44 (td, J = 6.9, 2.1 Hz, 2H, CH_2), 1.79 (p, J = 6.6 Hz, 2H, CH_2), 1.54 (br s, 1H, OH).

^{13}C{^{1}H} NMR (101 MHz, $CDCl_3$) δ 126.0, 117.5, 90.2, 80.0, 61.9, 31.4, 16.0.

Pent-4-en-2-yn-1-ol (1c)

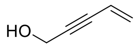

1c

Prepared according to the general procedure B2 using prop-2-yn-1-ol (1.16 mL, 20.0 mmol, 1.00 equiv.) and vinyl bromide (22.0 mL, 22.0 mmol, 1.10 equiv., 2M in THF). The crude material was purified by column chromatography (10 – 30 % (v/v) EtOAc in hexane) to give **1c** (0.867 g, 10.6 mmol, 53% yield) as a yellow oil. Spectral data was consistent with the values reported in literature.[14]

^{1}H NMR (400 MHz, $CDCl_3$) δ 5.82 (ddt, J = 17.6, 11.0, 1.9 Hz, 1H, CH=), 5.66 (dd, J = 17.6, 2.2 Hz, 1H, =CH_2), 5.50 (dd, J = 11.0, 2.2 Hz, 1H, =CH_2), 4.42 – 4.36 (m, 2H, OCH_2), 1.65 (t, J = 6.0 Hz, 1H, OH).

^{13}C{^{1}H} NMR (101 MHz, $CDCl_3$) δ 127.7, 116.7, 88.0, 84.6, 51.7.

((But-3-yn-1-yloxy)methyl)benzene (S13)

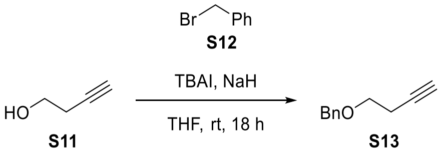

Following a reported procedure,[15] to a solution of but-3-yn-1-ol **S11** (0.76 mL, 10 mmol, 1.2 equiv.) in THF (8 mL) at 0 °C was added, sequentially, sodium hydride (400 mg, 60% in mineral oil, 10.0 mmol, 1.2 equiv.), tetrabutylammonium iodide (308 mg, 100 µmol, 0.10 equiv.), and benzyl bromide **S12** (1.0 mL, 8.3 mmol, 1 equiv.). The solution was warmed to rt and stirred for 16 h. The reaction was quenched by sat. aqueous NH_4Cl and extracted with Et_2O (3 × 15 mL). The combined organic layers were washed with water (15 mL) and brine (15 mL), dried over Na_2SO_4, filtered, and concentrated under vacuum. Column chromatography (10 % (v/v) EtOAc in pentane) provided **S13** as a pale-yellow oil (1.60 g, 10.0 mmol, quant.).[15]

^{1}H NMR (400 MHz, $CDCl_3$) δ 7.37 – 7.33 (m, 4H, ArH), 7.33 – 7.26 (m, 1H, ArH), 4.57 (s, 2H, PhCH_2O), 3.61 (t, J = 7.0 Hz, 2H, OCH_2C), 2.51 (td, J = 6.9, 2.7 Hz, 2H, CH_2CC), 1.99 (t, J = 2.7 Hz, 1H, CH).

^{13}C{^{1}H} NMR (101 MHz, $CDCl_3$) δ 138.2, 128.6, 127.9 (2C), 81.4, 73.2, 69.5, 68.3, 20.0.

((Hex-5-en-3-yn-1-yloxy)methyl)benzene (1d)

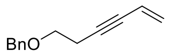

1d

Prepared according to the general procedure B2 using **S13** (1.60 g, 10.0 mmol, 1.00 equiv.) and vinyl bromide (11.0 mL, 11.0 mmol, 1.10 equiv., 2M in THF). The crude material was purified by column chromatography (10 – 30 % (v/v) EtOAc in hexane) to give **1d** (1.26 g, 6.77 mmol, 68% yield) as a colorless oil. Spectral data was consistent with the values reported in literature.[16]

^{1}H NMR (400 MHz, $CDCl_3$) δ 7.37 – 7.34 (m, 5H, ArH), 5.78 (ddt, J = 17.5, 11.0, 2.1 Hz, 1H, CH=), 5.57 (dd, J = 17.5, 2.2 Hz, 1H, =CH_2), 5.41 (dd, J = 11.0, 2.2 Hz, 1H, =CH_2), 4.57 (s, 2H, PhCH_2O), 3.61 (t, J = 7.0 Hz, 2H, OCH_2), 2.63 (td, J = 7.0, 2.0 Hz, 2H, CH_2CC).

^{13}C{^{1}H} NMR (101 MHz, $CDCl_3$) δ 138.2, 128.6, 127.9, 127.8, 126.3, 117.5, 87.7, 80.4, 73.1, 68.5, 20.9.

Oct-1-en-3-yne (1e)

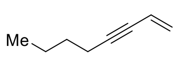

1e

Prepared according to the general procedure B2 using hex-1-yne (2.30 mL, 20.0 mmol, 1.00 equiv.) and vinyl bromide (22.0 mL, 22.0 mmol, 1.10 equiv., 2M in THF). The crude material was purified by column chromatography (10 – 30% (v/v) EtOAc in hexane) to give **1e** (0.89 g, 8.3 mmol, 41% yield) as a colorless oil. Spectral data was consistent with the values reported in literature.[17]

^{1}H NMR (400 MHz, $CDCl_3$) δ 5.78 (ddt, J = 17.5, 11.0, 2.1 Hz, 1H, CH=), 5.54 (dd, J = 17.5, 2.2 Hz, 1H, =CH_2), 5.37 (dd, J = 11.0, 2.3 Hz, 1H, =CH_2), 2.31 (td, J = 7.0, 2.1 Hz, 2H, CH_2), 1.56 – 1.47 (m, 2H, CH_2), 1.47 – 1.36 (m, 2H, CH_2), 0.92 (t, J = 7.3 Hz, 3H, CH_3).

^{13}C{^{1}H} NMR (101 MHz, $CDCl_3$) δ 125.6, 117.8, 91.4, 79.4, 30.9, 22.1, 19.2, 13.8.

But-3-en-1-yn-1-ylbenzene (1f)

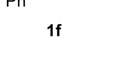

1f

Prepared according to the general procedure B2 using ethynylbenzene (1.10 mL, 10.0 mmol, 1.00 equiv.) and vinyl bromide (11.0 mL, 11.0 mmol, 1.10 equiv., 2M in THF). The crude material was purified by column chromatography (10 – 30 % (v/v) EtOAc in hexane) to give **1f** (1.15 g, 8.99 mmol, 90% yield) as an orange solid. Spectral data was consistent with the values reported in literature.

^{1}H NMR (400 MHz, $CDCl_3$) δ 7.49 – 7.40 (m, 2H, Ar*H*), 7.35 – 7.28 (m, 3H, Ar*H*), 6.03 (dd, J = 17.5, 11.1 Hz, 1H, C*H*=), 5.74 (dd, J = 17.5, 2.1 Hz, 1H, =CH_2), 5.55 (dd, J = 11.1, 2.1 Hz, 1H, =CH_2).

^{13}C{^{1}H} NMR (101 MHz, $CDCl_3$) δ 131.5, 128.3, 126.9, 123.1, 117.2, 90.0, 88.7.

2-(But-3-en-1-yn-1-yl)pyridine (1g)

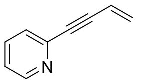

1g

Prepared according to the general procedure B2 using 2-ethynylpyridine (516 mg, 0.506 mL, 1.0 equiv.) and vinyl bromide (6.0 mL, 6.0 mmol, 1.20 equiv., 2M in THF). The crude material was purified by column chromatography (20% (v/v) EtOAc in hexane) to give **1g** (404 mg, 3.13 mmol, 63% yield) as a yellow oil. Spectral data was consistent with the values reported in literature.[18]

^{1}H NMR (400 MHz, $CDCl_3$) δ 8.59 (ddd, *J* = 4.9, 1.9, 1.0 Hz, 1H, Ar*H*), 7.65 (td, *J* = 7.7, 1.8 Hz, 1H, Ar*H*), 7.43 (dt, *J* = 7.8, 1.1 Hz, 1H, Ar*H*), 7.22 (ddd, *J* = 7.7, 4.9, 1.2 Hz, 1H, Ar*H*), 6.03 (dd, *J* = 17.6, 11.1 Hz, 1H, C*H*=), 5.85 (dd, *J* = 17.6, 2.1 Hz, 1H, =CH_2), 5.64 (dd, *J* = 11.1, 2.1 Hz, 1H, =CH_2).

^{13}C{^{1}H} NMR (101 MHz, $CDCl_3$) δ 150.1, 143.3, 136.1, 128.9, 127.0, 122.8, 116.6, 89.1, 87.8.

2-Ethynylphenol (S17)

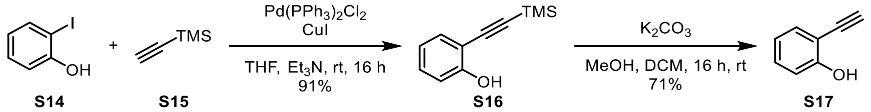

Prepared according to the general procedure B2 using $Pd(PPh_3)_2Cl_2$ (263 mg, 0.374 mmol, 2 mol%), 2-iodophenol **S14** (2.1 mL, 19 mmol, 1.25 equiv.) and ethynyl(trimethyl)silane **S15** (2.1 mL, 15 mmol, 1.0 equiv.). The crude material was purified by column chromatography (10 – 20% (v/v) EtOAc in hexane) to give **S16** (2.59 g, 13.6 mmol, 91% yield) as a brown oil.

^{1}H NMR (400 MHz, $CDCl_3$) δ 7.34 (dd, *J* = 7.7, 1.6 Hz, 1H, Ar*H*), 7.29 – 7.20 (m, 1H, Ar*H*), 6.94 (d, *J* = 8.3 Hz, 1H, Ar*H*), 6.85 (td, *J* = 7.6, 0.9 Hz, 1H, Ar*H*), 5.82 (s, 1H, O*H*), 0.28 (s, 9H, Si(CH_3)$_3$).

To a solution of **S16** (0.56 g, 3.0 mmol, 1.0 equiv.) in methanol (6 mL) and DCM (3 mL) (v/v = 2:1) was added K_2CO_3 (1.84 g, 13.3 mmol, 4.5 equiv.) and stirred at room temperature for 24 h. The resulting mixture was treated with water and extracted with diethyl ether (3 × 10 mL). The combined organic layer was washed with brine and dried with Na_2SO_4. The solvent was removed and the residue was purified by silica gel column chromatography (5 – 10 % (v/v) EtOAc in pentane) to afford **S17** (247 mg, 2.09 mmol, 71%). Spectral data was consistent with the values reported in literature.[19]

^{1}H NMR (400 MHz, $CDCl_3$) δ 7.38 (dd, *J* = 7.7, 1.6 Hz, 1H, Ar*H*), 7.30 – 7.27 (m, 1H, Ar*H*), 6.95 (d, *J* = 8.3 Hz, 1H, Ar*H*), 6.88 (td, *J* = 7.6, 1.0 Hz, 1H, Ar*H*), 5.78 (s, 1H, O*H*), 3.47 (s, 1H, C*H*).

^{13}C{^{1}H} NMR (101 MHz, $CDCl_3$) δ 157.5, 132.2, 131.1, 120.5, 115.0, 108.4, 84.5, 78.4.

2-(But-3-en-1-yn-1-yl)phenol (1h)

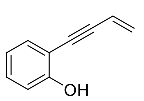

1h

Prepared according to the general procedure B2 using 2-ethynylphenol **S17** (247 mg, 2.09 mmol, 1.0 equiv.) and vinyl bromide (2.5 mL, 2.5 mmol, 1.2 equiv., 2M in THF). The crude material was purified by column chromatography (2 – 15 % (v/v) EtOAc in hexane) to give **1h** (199 mg, 1.38 mmol, 66% yield) as a yellow oil. Spectral data was consistent with the values reported in literature.[20]

^{1}H NMR (400 MHz, $CDCl_3$) δ 7.52 (d, *J* = 7.6 Hz, 1H, Ar*H*), 7.45 (d, *J* = 8.1 Hz, 1H, Ar*H*), 7.27 (td, *J* = 7.8, 1.3 Hz, 1H, Ar*H*), 7.19 (td, *J* = 7.4, 0.8 Hz, 1H, Ar*H*), 6.64 (dd, *J* = 17.5, 11.2 Hz, 1H, C*H*=), 6.60 (s, 1H, O*H*), 5.96 (d, *J* = 17.5 Hz, 1H, =CH_2), 5.38 (dd, *J* = 11.2, 1.1 Hz, 1H, =CH_2).

^{13}C{^{1}H} NMR (101 MHz, $CDCl_3$) δ 155.0, 154.9, 129.0, 125.4, 124.8, 122.9, 121.1, 115.9, 111.2, 104.9.

***N*-(But-3-yn-1-yl)-4-methylbenzenesulfonamide (S19)**

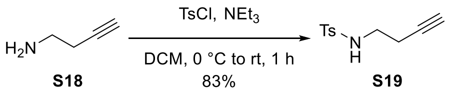

Following a reported procedure,[21] to a solution of 3-butyn-1-amine **S18** (553 mg, 8.00 mmol) and triethylamine (2.2 mL, 16 mmol) in DCM (25 mL) was added paratoluenesulfonyl chloride (1.68 g, 8.81 mmol) at 0 ºC. After being stirred at room temperature for 1 h, the reaction mixture was quenched with H_2O and extracted with CH_2Cl_2. The organic layer was dried over $MgSO_4$ and concentrated in vacuo to dryness. The residue was purified by silica gel column chromatography (25 % (v/v) EtOAc in hexane) to give **S19** (1.49 g, 6.67 mmol, 83%). Spectral data was consistent with the values reported in the literature.[21]

^{1}H NMR (400 MHz, $CDCl_3$) δ 7.79 – 7.71 (m, 2H, Ar*H*), 7.38 – 7.29 (m, 2H, Ar*H*), 4.71 (s, 1H, N*H*), 3.12 (q, *J* = 6.5 Hz, 2H, CH_2N), 2.43 (s, 3H, ArCH_3), 2.35 (td, *J* = 6.5, 2.6 Hz, 2H, CH_2), 2.00 (t, *J* = 2.6 Hz, 1H, ≡C*H*).

N-(Hex-5-en-3-yn-1-yl)-4-methylbenzenesulfonamide (1i)

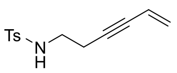

1i

Prepared according to the general procedure B2 using N-(but-3-yn-1-yl)-4-methylbenzenesulfonamide **S19** (560 mg, 2.51 mmol, 0.605 mL, 1.00 equiv.) and vinyl bromide (295 mg, 2.76 mmol, 2.76 mL, 1.10 equiv.). The crude material was purified by column chromatography (20 – 30% (v/v) EtOAc/hexane) to give **1i** (400 mg, 1.60 mmol, 64%) as an orange solid.

R_f (20% EtOAc/hexane) = 0.32.

^{1}H NMR (400 MHz, $CDCl_3$) δ 7.80 – 7.72 (m, 2H, Ar*H*), 7.32 (d, J = 8.2 Hz, 2H, Ar*H*), 5.79 – 5.66 (m, 1H, C*H*=), 5.56 (dd, J = 17.5, 2.2 Hz, 1H, =CH_2), 5.44 (dd, J = 11.0, 2.2 Hz, 1H, =CH_2), 4.66 (t, J = 6.4 Hz, 1H, N*H*), 3.12 (qd, J = 6.4, 1.1 Hz, 2H, CH_2N), 2.47 (td, J = 6.5, 2.0 Hz, 2H, CH_2), 1.54 (d, J = 0.9 Hz, 3H, ArCH_3).

^{13}C{^{1}H} NMR (101 MHz, $CDCl_3$) δ 143.6, 137.0, 129.8, 127.1, 127.0, 116.8, 86.2, 81.6, 41.8, 21.5, 20.6.

IR (cm^{-1}) 3284 (w), 2360 (w), 1719 (w), 1598 (w), 1420 (w), 1326 (m), 1158 (s), 1088 (m).

HRMS (nanochip-ESI/LTQ-Orbitrap) m/z: [M + Na]$^+$ Calcd for $C_{13}H_{15}NNaO_2S^+$ 272.0716; Found 272.0722.

Hept-6-en-4-yn-2-ol (1j)

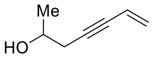

1j

Prepared according to the general procedure B2 using pent-4-yn-2-ol (0.94 mL, 10 mmol, 1.0 equiv.) and vinyl bromide (11.0 mL, 11.0 mmol, 1.10 equiv., 2 M in THF). The crude material was purified by column chromatography (10 – 30 % (v/v) EtOAc in hexane) to give **1j** (0.66 g, 6.9 mmol, 69% yield) as an orange oil.

R_f (20 % EtOAc/hexane) = 0.33.

^{1}H NMR (400 MHz, $CDCl_3$) δ 5.79 (ddt, J = 17.5, 11.0, 2.1 Hz, 1H, C*H*=), 5.59 (dd, J = 17.5, 2.2 Hz, 1H, =CH_2), 5.43 (dd, J = 11.0, 2.2 Hz, 1H, =CH_2), 4.04 – 3.91 (m, 1H, C*H*), 2.52 (ddd, J = 16.7, 5.1, 2.1 Hz, 1H, CH_2), 2.44 (ddd, J = 16.7, 6.6, 2.1 Hz, 1H, CH_2), 1.94 (s, 1H, O*H*), 1.27 (d, J = 6.2 Hz, 3H, CH_3).

^{13}C{^{1}H} NMR (101 MHz, $CDCl_3$) δ 126.6, 117.3, 87.0, 81.9, 66.6, 30.1, 22.5.

IR (cm^{-1}) 3362 (m), 2975 (m), 2912 (w), 2228 (w), 1605 (m), 1417 (m).

HRMS (Sicrit plasma/LTQ-Orbitrap) m/z: [M + H]$^+$ Calcd for $C_7H_{11}O^+$ 111.0804; Found 111.0804.

5-Methylhex-5-en-3-yn-1-ol (1k)

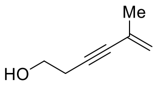

1k

Prepared according to the general procedure B2 using but-3-yn-1-ol (0.38 mL, 5.0 mmol, 1.0 equiv.) and 2-bromopropene (0.48 g, 5.5 mmol, 1.1 equiv.) in 5 mL of THF. The crude material was purified by column chromatography (10 – 40 % (v/v) EtOAc in hexane) to give **1k** (0.52 g, 4.7 mmol, 95% yield) as a pale-yellow oil. Spectral data was consistent with the values reported in literature.[22]

^{1}H NMR (400 MHz, $CDCl_3$) δ 5.24 (s, 1H, =CH_2), 5.21 – 5.15 (m, 1H, =CH_2), 3.78 – 3.69 (m, 2H, OCH_2), 2.58 (t, J = 6.3 Hz, 2H, CCH_2), 1.91 – 1.82 (m, 3H, CH_3), 1.81 (s, 1H, O*H*).

^{13}C{^{1}H} NMR (101 MHz, $CDCl_3$) δ 126.9, 121.5, 85.4, 83.9, 61.3, 23.9, 23.8.

5-Phenylhex-5-en-3-yn-1-ol (S20)

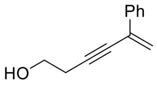

S20

Prepared according to the general procedure B2 using but-3-yn-1-ol (0.38 mL, 5.0 mmol, 1.0 equiv.) and α-bromostyrene (0.70 g, 5.5 mmol, 1.1 equiv.). The crude material was purified by column chromatography (10 – 40 % (v/v) EtOAc in hexane) to give **S20** (0.34 g, 3.5 mmol, 71% yield) as a yellow oil. Spectral data was consistent with the values reported in literature.[23]

^{1}H NMR (400 MHz, $CDCl_3$) δ 7.66 – 7.61 (m, 2H, Ar*H*), 7.39 – 7.27 (m, 3H, Ar*H*), 5.88 (s, 1H, =CH_2), 5.63 (s, 1H, =CH_2), 3.82 (q, J = 6.1 Hz, 2H, OCH_2), 2.71 (t, J = 6.3 Hz, 2H, CCH_2), 1.78 (t, J = 5.7 Hz, 1H, O*H*).

^{13}C{^{1}H} NMR (101 MHz, $CDCl_3$) δ 137.6, 130.7, 128.5, 128.4, 126.2, 120.5, 88.0, 81.9, 61.3, 24.0.

5-(Trifluoromethyl)hex-5-en-3-yn-1-ol (S21)

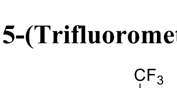

S21

Prepared according to the general procedure B2 using but-3-yn-1-ol (0.38 mL, 5.0 mmol, 1.0 equiv.) and 2-bromo-3,3,3-trifluoroprop-1-ene (0.96 g, 5.5 mmol, 1.1 equiv.). The crude material was purified by column chromatography (10 – 40 % (v/v) EtOAc in hexane) to give **S21** (0.41 g, 2.5 mmol, 50% yield) as a yellow oil. Spectral data was consistent with the values reported in literature.[24]

^{1}H NMR (400 MHz, $CDCl_3$) δ 6.02 (d, J = 1.3 Hz, 1H, =CH_2), 5.85 – 5.80 (m, 1H, =CH_2), 3.79 (q, J = 6.0 Hz, 2H, OCH_2), 2.64 (t, J = 6.3 Hz, 2H, CCH_2), 1.74 (s, 1H, O*H*).

^{13}C{^{1}H} NMR (101 MHz, $CDCl_3$) δ 126.7 (q, $J_{C\text{-}F}$ = 4.5 Hz), 122.8, 121.3 (q, $J_{C\text{-}F}$ = 237.8 Hz), 91.5, 75.0, 60.9, 23.8.

^{19}F NMR (376 MHz, $CDCl_3$) δ -68.3.

6-Phenylhex-5-en-3-yn-1-ol (S22)

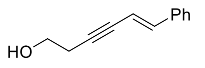

S22

Prepared according to the general procedure B2 using but-3-yn-1-ol (0.76 mL, 10 mmol, 1.0 equiv.) and 2-bromoethenylbenzene (2.01 g, 11.0 mmol, 1.1 equiv.). The crude material was purified by column chromatography (10 – 40 % (v/v) EtOAc in hexane) to give **S22** (1.56 g, 9.20 mmol, 92% yield) as an orange solid. Spectral data was consistent with the values reported in literature.[25]

^{1}H NMR (400 MHz, $CDCl_3$) δ 7.43 – 7.25 (m, 5H, Ar*H*), 6.94 (d, *J* = 16.3 Hz, 1H, =C*H*Ph), 6.17 (dt, *J* = 16.3, 2.2 Hz, 1H, =C*H*C), 3.81 (q, *J* = 5.9 Hz, 2H, OCH_2), 2.68 (td, *J* = 6.2, 2.2 Hz, 2H, CCH_2), 1.89 (t, *J* = 5.8 Hz, 1H, O*H*).

^{13}C{^{1}H} NMR (101 MHz, $CDCl_3$) δ 141.1, 136.4, 128.8, 128.6, 126.3, 108.3, 88.9, 81.8, 61.3, 24.2.

***N*-(But-3-yn-1-yl)-1,1,1-trifluoromethanesulfonamide (S23)**

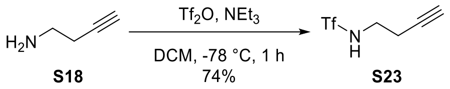

To a stirred solution of but-3-yn-1-amine **S18** (0.30 mL, 3.7 mmol) and triethylamine (0.51 mL, 3.7 mmol) in DCM (20 mL) was added triflic anhydride (0.65 mL, 3.8 mmol) slowly at –78 °C under inert atmosphere. After 1 hour, the crude reaction mixture was quenched with water and extracted with EtOAc. The combined organic layer was dried and concentrated under reduced pressure. The residue was purified by silica gel column chromatography (25 % (v/v) EtOAc in hexane) to give *N*-(but-3-yn-1-yl)-1,1,1-trifluoromethanesulfonamide **S23** (546 mg, 2.71 mmol, 74%).

^{1}H NMR (400 MHz, $CDCl_3$) δ 5.12 (s, 1H, N*H*), 3.46 (t, *J* = 6.1 Hz, 2H, CH_2N), 2.53 (td, *J* = 6.3, 2.6 Hz, 2H, CH_2), 2.13 (t, *J* = 2.7 Hz, 1H, ≡C*H*).

^{13}C{^{1}H} NMR (101 MHz, $CDCl_3$) δ 119.6 (d, *J* = 320.8 Hz), 79.2, 71.9, 42.8, 20.6.

HRMS (Sicrit plasma/LTQ-Orbitrap) m/z: [M + H]+ Calcd for $C_5H_7F_3NO_2S^+$ 202.0144; Found 202.0143.

1,1,1-Trifluoro-*N*-(hex-5-en-3-yn-1-yl)methanesulfonamide (S24)

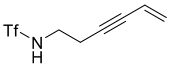

xx

Prepared according to the general procedure B2 using *N*-(but-3-yn-1- yl)-1,1,1-trifluoromethanesulfonamide **S23** (546 mg, 2.71 mmol, 1.00 equiv.) and vinyl bromide (2.98 mL, 2.98 mmol, 1.10 equiv., 1 M in THF). The crude material was purified by column chromatography (10 – 30% (v/v) EtOAc/hexane) to give **S24** (400 mg, 1.60 mmol, 64%).

^{1}H NMR (400 MHz, $CDCl_3$) δ 5.83 – 5.71 (m, 1H, C*H*=), 5.68 – 5.59 (m, 1H, =CH_2), 5.50 (dd, *J* = 10.9, 2.2 Hz, 1H, =CH_2), 3.46 (t, *J* = 6.3 Hz, 2H, CH_2N), 2.64 (td, *J* = 6.3, 2.0 Hz, 2H, CCH_2).

^{13}C{^{1}H} NMR (101 MHz, $CDCl_3$) δ 127.6, 119.6 (q, J_{C-F} = 320.8 Hz), 116.5, 85.0, 82.4, 43.0, 21.4.

HRMS (Sicrit plasma/LTQ-Orbitrap) m/z: $[M + H]^+$ Calcd for $C_7H_9F_3NO_2S^+$ 228.0301; Found 228.0301.

***N*-(hex-5-en-3-yn-1-yl)-4-methoxyaniline (S27)**

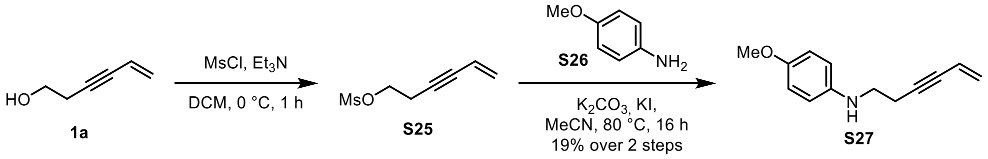

Following a reported procedure, a flask equipped with a magnetic stirrer was charged with hex-5-en-3-yn-1-ol **1a** (500 mg, 5.20 mmol, 1.00 equiv.), methanesulfonyl chloride (0.44 mL, 5.7 mmol, 1.1 equiv.), and DCM (15 mL). The solution was allowed to cool to 0 °C and then triethylamine (0.87 mL, 6.2 mmol, 1.2 equiv.) was added dropwise over 10 min. The reaction mixture was stirred at 0 °C for 1 hour. After that, the reaction mixture was quenched with saturated aq. NH_4Cl and extracted with DCM (3 x 15 mL). The combined organic layers were dried over anhydrous Na_2SO_4 and the solvent was removed under reduced pressure. The crude mixture of **S25** was directly engaged in the next step.

^{1}H NMR (400 MHz, $CDCl_3$) δ 5.76 (ddt, *J* = 17.6, 10.9, 2.1 Hz, 1H, C*H*=), 5.61 (dd, *J* = 17.6, 2.1 Hz, 1H, =CH_2), 5.46 (dd, *J* = 11.0, 2.3 Hz, 1H, =CH_2), 4.32 (t, *J* = 6.8 Hz, 2H, CH_2OMs), 3.05 (s, 3H, CH_3), 2.78 (td, *J* = 6.8, 2.1 Hz, 2H, CH_2).

To the crude **S25**, potassium carbonate (1.73 g, 12.5 mmol, 2.4 equiv.), potassium iodide (2.59 g, 15.6 mmol), *p*-methoxyaniline **S26** (3.20 g, 26.0 mmol, 5.0 equiv.) were added and dissolved in acetonitrile (50 mL) and the mixture was stirred at 80 °C for 18 h. The crude was purified by silica gel column chromatography (hexane/EtOAc 9:1 (v/v)) to obtain **S27** as a yellow oil (198 mg, 984 μmol, 19% over 2 steps).

^{1}H NMR (400 MHz, $CDCl_3$) δ 6.84 – 6.75 (m, 2H, Ar*H*), 6.66 – 6.56 (m, 2H, Ar*H*), 5.79 (ddt, *J* = 17.5, 11.0, 2.1 Hz, 1H, C*H*=), 5.59 (dd, *J* = 17.5, 2.2 Hz, 1H, =C*H*$_2$), 5.43 (dd, *J* = 11.0, 2.2 Hz, 1H, =C*H*$_2$), 3.75 (s, 3H, OC*H*$_3$), 3.66 (s, 1H, N*H*), 3.28 (t, *J* = 6.6 Hz, 2H, NC*H*$_2$), 2.61 (td, *J* = 6.6, 1.9 Hz, 2H, CC*H*$_2$).

^{13}C{^{1}H} NMR (101 MHz, $CDCl_3$) δ 152.6, 142.0, 126.5, 117.4, 115.1, 114.8, 88.2, 81.1, 56.0, 43.8, 20.3.

IR (cm^{-1}) 3386 (w), 2950 (w), 2834 (w), 2222 (w), 1847 (w), 1606 (w), 1511 (s), 1235 (s).

HRMS (ESI/QTOF) m/z: [M + H]$^+$ Calcd for $C_{13}H_{16}NO^+$ 202.1226; Found 202.1224.

B.3. Synthesis of amines

Commercially available anilines:

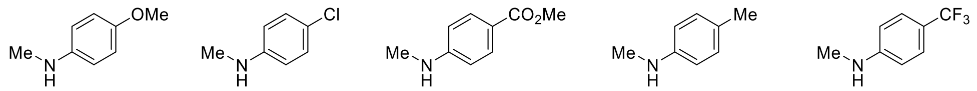

2a **2b** **2c** **2d** **2e**

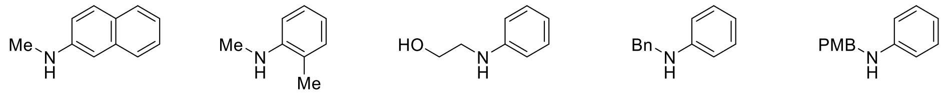

2f **2l** **2k** **2m** **2n**

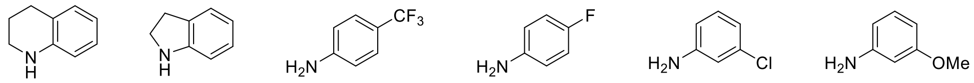

2o **2p** **2q** **2r** **2s** **2t**

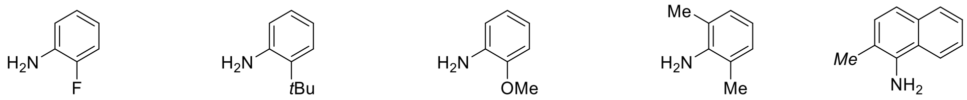

2u **2v** **2w** **2x** **2y**

3-(*Tert*-butyl)-*N*-methylaniline (2g)

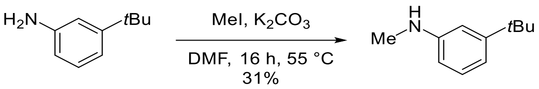

S28 **2g**

Following a reported procedure,[26] to a solution of 3-*tert*-butylaniline **S28** (1.49 g, 10.0 mmol, 1.0 equiv.) in DMF (25 mL) at 0 °C, K_2CO_3 (1.80 g, 13.0 mmol, 1.3 equiv.) was added slowly. The reaction mixture was stirred at 0 °C for 15 minutes and MeI (750 μL, 12.0 mmol, 1.2 equiv.) was added. Then the reaction mixture was heated to 55 °C for 16 h. After complete consumption of the starting material, the reaction mixture was cooled to room temperature and extracted with DCM (3 × 20 mL). The combined organic layers were washed with brine (5 × 20 mL), dried over Na_2SO_4 and concentrated under vacuum. The residue was purified by flash column chromatography on silica gel (20% EtOAc in hexane) to afford **2g** (506 mg, 3.10 mmol, 31%) as a colorless oil. Spectral data were consistent with the values reported in literature.[26]

^{1}H NMR (400 MHz, $CDCl_3$) δ 7.16 (t, *J* = 7.9 Hz, 1H, Ar*H*), 6.78 (ddd, *J* = 7.8, 1.7, 0.9 Hz, 1H, Ar*H*), 6.66 (t, *J* = 2.1 Hz, 1H, Ar*H*), 6.46 (ddd, *J* = 8.0, 2.4, 0.8 Hz, 1H, Ar*H*), 3.67 (s, 1H, N*H*), 2.86 (s, 3H, C*H*$_3$), 1.31 (s, 9H, C(C*H*$_3$)$_3$).

^{13}C{^{1}H} NMR (101 MHz, $CDCl_3$) δ 152.4, 149.3, 129.0, 114.8, 110.2, 109.5, 34.8, 31.5, 31.0.

N-Methyl-3,4-dihydro-2*H*-benzo[b][1,4]dioxepin-7-amine (2h)

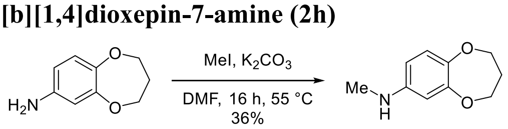

S29 **2h**

Following a reported procedure,[26] to a solution of 3,4-dihydro-2*H*-benzo[*b*][1,4]dioxepin-7-amine **S29** (200 mg, 1.21 mmol, 1.0 equiv.) in DMF (3 mL) at 0 °C, K_2CO_3 (218 mg, 1.57 mmol, 1.3 equiv.) was added slowly. The reaction mixture was stirred at 0 °C for 15 minutes and MeI (91 μL, 1.5 mmol, 1.2 equiv.) was added. Then the reaction mixture was heated to 55 °C for 16 h. After complete consumption of the starting material, the reaction mixture was cooled to room temperature and extracted with DCM (3 × 10 mL). The combined organic layers were washed with brine (5 × 10 mL), dried over Na_2SO_4 and concentrated under vacuum. The residue was purified by flash column

chromatography on silica gel (20% to 50% EtOAc in hexane) to afford **2h** (77.5 mg, 432 μmol, 36%) as a colorless oil.

R_f(50% EtOAc/hexane) = 0.59.

^{1}H NMR (400 MHz, $CDCl_3$) δ 6.84 (d, *J* = 8.6 Hz, 1H, Ar*H*), 6.28 (d, *J* = 2.8 Hz, 1H, Ar*H*), 6.21 (dd, *J* = 8.6, 2.8 Hz, 1H, Ar*H*), 4.20 – 4.12 (m, 2H, OCH_2), 4.11 – 4.02 (m, 2H, OCH_2), 3.52 (s, 1H, N*H*), 2.78 (s, 3H, NCH_3), 2.19 – 2.09 (m, 2H, OCH$_2$CH_2).

^{13}C{^{1}H} NMR (101 MHz, $CDCl_3$) δ 152.4, 146.2, 143.2, 122.3, 107.7, 105.3, 71.1, 71.0, 32.7, 31.4.

IR (cm^{-1}) 3401 (w), 2947 (w), 2875 (w), 2811 (w), 1616 (m), 1509 (s).

HRMS (ESI/QTOF) m/z: $[M + H]^+$ Calcd for $C_{10}H_{14}NO_2^+$ 180.1019; Found 180.1027.

4-Methoxy-*N*-phenethylaniline (2j)

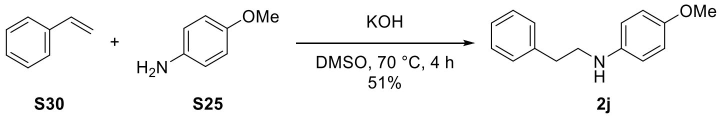

Following a reported procedure,[27] an oven-dried microwave tube equipped with a magnetic stirring bar was charged with *p*-anisidine **S26** (394 mg, 3.20 mmol, 1.6 equiv.), styrene **S30** (208 mg, 2.00 mmol, 1.0 equiv.) and KOH (5.6 mg, 0.10 mmol, 5 mol%). The tube was evacuated and back-filled with N_2 three times. Then, dry DMSO (1 mL) was added and the resulting mixture was heated to 70 °C for 4 h. Next, the reaction mixture was allowed to cool down to room temperature and was poured into an aq. NaOH solution (1 M, 10 mL) and extracted with DCM (3 × 10 mL). The combined organic layers were dried with Na_2SO_4 and concentrated under vacuum. The crude product was purified by flash chromatography in silica gel (9:1 hexane/EtOAc) to give **2j** (230 mg, 1.01 mmol, 51%) as a pale-yellow oil. Spectral data were consistent with the values reported in literature.[27]

^{1}H NMR (400 MHz, $CDCl_3$) δ 7.35 (t, *J* = 7.3 Hz, 2H, Ar*H*), 7.26 (dd, *J* = 9.3, 7.8 Hz, 3H, Ar*H*), 6.86 – 6.77 (m, 2H, Ar*H*), 6.66 – 6.57 (m, 2H, Ar*H*), 3.78 (s, 3H, OCH_3), 3.43 (d, *J* = 10.2 Hz, 1H, N*H*), 3.39 (t, *J* = 7.0 Hz, 2H, NCH_2), 2.94 (t, *J* = 7.0 Hz, 2H, PhCH_2).

^{13}C{^{1}H} NMR (101 MHz, $CDCl_3$) δ 152.4, 142.4, 139.6, 128.9, 128.7, 126.5, 115.1, 114.5, 56.0, 46.2, 35.8.

***N*-(3-Phenylprop-2-yn-1-yl)aniline (2l)**

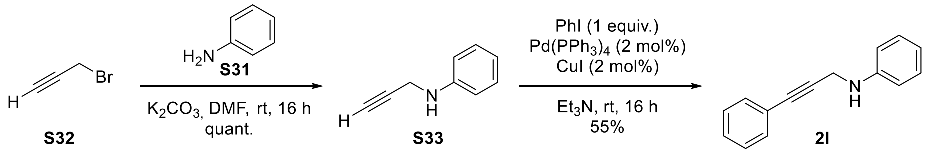

Following a reported procedure,[28] to a solution of aniline **S31** (6.75 mL, 74.8 mmol, 4.0 equiv.) in DMF (15 mL) was added K_2CO_3 (5.17 g, 2.0 equiv.). Five minutes later, a solution of 3-bromopropyne **S32** (2.00 mL, 18.7 mmol, 1.0 equiv.) in DMF (5 mL) was added dropwise. The reaction mixture was stirred at room temperature overnight. The mixture was extracted with EtOAc, and the combined extracts were washed with brine and dried over Na_2SO_4. After removal of the solvent in vacuo, the residue was purified by column chromatography on a silica gel (hexane/EtOAc = 9/1) to afford **S33** (3.88 g, 74.8 mmol, 100% yield) as a yellow liquid. Spectral data were consistent with the values reported in literature.[28]

^{1}H NMR (400 MHz, $CDCl_3$) δ 7.26 – 7.19 (m, 2H, Ar*H*), 6.84 – 6.74 (m, 1H, Ar*H*), 6.74 – 6.66 (m, 2H, Ar*H*), 3.95 (d, *J* = 2.4 Hz, 2H, CH_2), 3.86 (s, 1H, N*H*), 2.22 (t, *J* = 2.4 Hz, 1H, C*H*).

Following a reported procedure,[29] to a solution of Et_3N (30 mL), $Pd(PPh_3)_4$ (352 mg, 305 μmol, 0.02 equiv.), CuI (58.9 mg, 305 μmol, 0.02 equiv.), was added phenyl iodide (1.70 mL, 15.3 mmol, 1.0 equiv.) and **S33** (2.00 g, 15.3 mmol, 1.0 equiv.). The reaction proceeded at rt overnight under N_2 atmosphere before Et_3N was removed by rotary evaporation. The remaining mixture was extracted with EtOAc, and the combined organic layers were washed with brine, dried over Na_2SO_4, and concentrated in vacuo. The residue was purified by column chromatography on a silica gel (hexane/EtOAc = 9/1) to provide **2l** (1.72 g, 8.32 mmol, 55% yield) as a yellow liquid. Spectral data were consistent with the values reported in literature.[29]

^{1}H NMR (400 MHz, $CDCl_3$) δ 7.41 (dd, *J* = 6.6, 3.1 Hz, 2H, Ar*H*), 7.34 – 7.20 (m, 5H, Ar*H*), 6.80 (t, *J* = 7.3 Hz, 1H, Ar*H*), 6.75 (dd, *J* = 8.5, 0.9 Hz, 2H, Ar*H*), 4.16 (s, 2H, CH_2), 3.96 (s, 1H, N*H*).

^{13}C{^{1}H} NMR (101 MHz, $CDCl_3$) δ 147.3, 131.8, 129.4, 128.38, 128.35, 123.0, 118.6, 113.7, 86.5, 83.4, 34.7.

C. Ligand screening

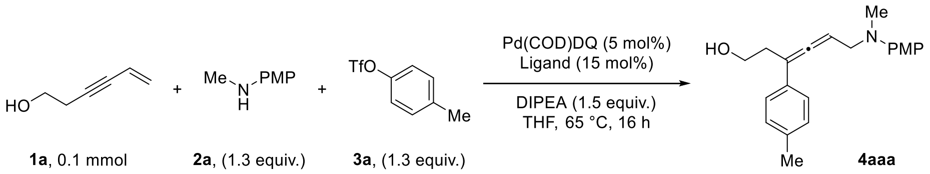

General Procedure for Optimization: An oven-dried 8 mL microwave tube equipped with a Teflon coated stirring bar was charged with Pd(COD)DQ (1.9 mg, 5.0 µmol, 5 mol%), Ligand (15 µmol, 15 mol%) and amine **2a** (0.13 mmol, 1.3 equiv.). The tube was evacuated and back-filled with N_2 three times. Then, THF (0.2 mL) and DIPEA (26 µL, 0.25 mmol, 1.5 equiv.) were added and the mixture was stirred at 70 °C for 5 minutes. Afterwards, the corresponding enyne **1a** (0.1 mmol) and aryl triflate **3a** (0.13 mmol, 1.3 equiv.) were added. The resulting solution was then stirred at 65 °C for 16 h. Next, the reaction mixture was allowed to cool down to room temperature and concentrated under vacuum. The crude material was analyzed by ^{1}H NMR using trichloroethylene as internal standard.

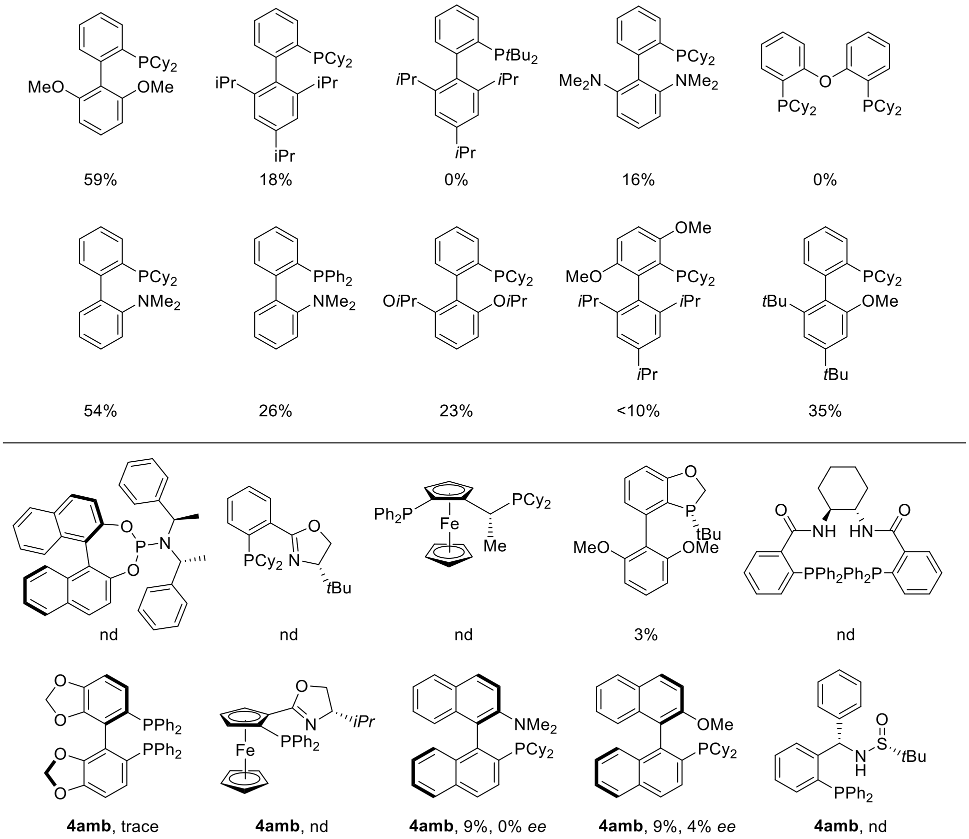

D. Procedures and product characterization data of carboamination products

D.1. General Procedure D1 for the synthesis of allenes

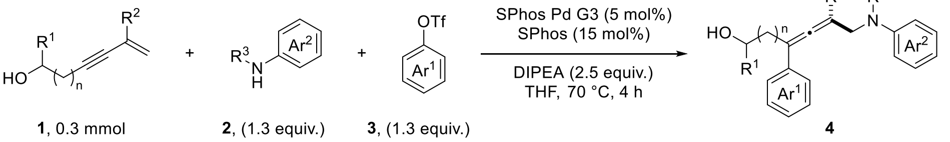

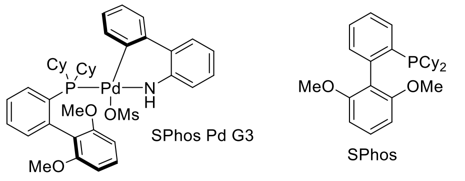

An oven-dried 8 mL microwave tube equipped with a Teflon coated stirring bar was charged with SPhos Pd G3 (11.7 mg, 15.0 µmol, 5 mol%), SPhos (18.5 mg, 45.0 µmol, 15 mol%) and amine **2** (0.39 mmol, 1.3 equiv.). The tube was evacuated and back-filled with N_2 three times. Then, THF (0.6 mL) and DIPEA (131 µL, 750 µmol, 2.5 equiv.) were added and the mixture was stirred at 70 °C for 5 minutes. Afterwards, the corresponding enyne **1** (0.3 mmol) and aryl triflate **3** (0.39 mmol, 1.3 equiv.) were added. The resulting solution was then stirred at 70 °C for 4 h. Next, the reaction

mixture was allowed to cool down to room temperature and concentrated under vacuum. The crude material was purified by flash column chromatography on silica gel using a Biotage flash chromatography machine to afford the corresponding product **4**. For long-term storage, allenes should be kept in a freezer to prevent decomposition.

D.2. Characterization of the allene products

6-((4-Methoxyphenyl)(methyl)amino)-3-(p-tolyl)hexa-3,4-dien-1-ol (4aaa)

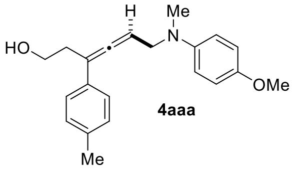

4aaa

Prepared according to the general procedure D1 using hex-5-en-3-yn-1-ol **1a** (28.8 mg, 300 µmol, 1.0 equiv.), 4-methoxy-*N*-methyl-aniline **2a** (53.5 mg, 390 µmol, 1.30 equiv.) and **3a** (93.7 mg, 390 µmol, 1.30 equiv.). The crude material was purified by column chromatography (20 – 50 % (v/v) EtOAc in hexane) to give **4aaa** (74.1 mg, 229 µmol, 76% yield) as a yellow oil.

R_f(50% EtOAc/hexane) = 0.60.

^{1}H NMR (400 MHz, $CDCl_3$) δ 7.19 (d, *J* = 8.2 Hz, 2H, Ar*H*), 7.10 (d, *J* = 8.1 Hz, 2H, Ar*H*), 6.81 (s, 4H, Ar*H*), 5.58 (dt, *J* = 5.7, 2.8 Hz, 1H, C=C*H*), 3.95 (d, *J* = 5.8 Hz, 2H, NCH_2), 3.80 – 3.69 (m, 5H, OCH_3 and OCH_2), 2.87 (s, 3H, NCH_3), 2.62 (tt, *J* = 6.2, 2.6 Hz, 2H, CCH_2), 2.33 (s, 3H, ArCH_3).

^{13}C{^{1}H} NMR (101 MHz, $CDCl_3$) δ 204.2, 136.8, 133.0, 129.3, 127.5, 126.0, 124.8, 116.6, 114.8, 103.9, 91.2, 61.0, 55.8, 53.8, 40.1, 33.5, 21.2.

IR (cm^{-1}) 3457 (w), 2915 (w), 2831 (w), 2245 (w), 2049 (w), 1941 (w), 1512 (s), 1444 (m), 1245 (s).

HRMS (ESI/QTOF) m/z: $[M + H]^+$ Calcd for $C_{21}H_{26}NO_2^+$ 324.1958; Found 324.1960.

7-((4-Methoxyphenyl)(methyl)amino)-4-(*p*-tolyl)hepta-4,5-dien-1-ol (4baa)

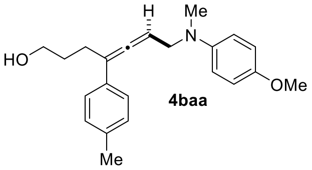

4baa

Prepared according to the general procedure D1 using **1b** (22.0 mg, 200 µmol, 1.0 equiv.), 4-methoxy-*N*-methyl-aniline **2a** (35.7 mg, 260 µmol, 1.30 equiv.) and (4-methylphenyl) trifluoromethanesulfonate **3a** (62.5 mg, 260 µmol, 1.30 equiv.). The crude material was purified by column chromatography (20 – 50 % (v/v) EtOAc in hexane) to give **4baa** (12.3 mg, 30.0 µmol, 15% yield) as a yellow oil.

R_f(50 % EtOAc/hexane) = 0.46.

^{1}H NMR (400 MHz, $CDCl_3$) δ 7.21 (d, *J* = 8.2 Hz, 2H, Ar*H*), 7.10 (d, *J* = 8.0 Hz, 2H, Ar*H*), 6.85 – 6.80 (m, 2H, Ar*H*), 6.80 – 6.74 (m, 2H, Ar*H*), 5.50 (tt, *J* = 6.0, 2.9 Hz, 1H, C=C*H*), 3.99 (dd, *J* = 6.0, 2.8 Hz, 2H, NCH_2), 3.76 (s, 3H, OCH_3), 3.65 (td, *J* = 6.4, 0.9 Hz, 2H, OHCH_2CH$_2$), 2.89 (s, 3H, NCH_3), 2.45 – 2.38 (m, 2H, OHCH$_2$CH_2), 2.33 (s, 3H, ArCH_3), 1.71 – 1.62 (m, 3H, OHCH$_2$CH$_2$CH_2 and O*H*).

^{13}C{^{1}H} NMR (101 MHz, $CDCl_3$) δ 204.2, 152.2, 144.0, 136.6, 133.5, 129.2, 126.0, 115.8, 114.8, 106.2, 90.5, 62.6, 55.9, 53.5, 39.2, 31.0, 26.4, 21.2.

IR (cm^{-1}) 3383 (w), 2939 (m), 1947 (w), 1510 (s), 1444 (w), 1243 (s).

HRMS (ESI/QTOF) m/z: $[M + H]^+$ Calcd for $C_{22}H_{28}NO_2^+$ 338.2115; Found 338.2112.

N-(6-(Benzyloxy)-4-(*p*-tolyl)hexa-2,3-dien-1-yl)-4-methoxy-*N*-methylaniline (4daa)

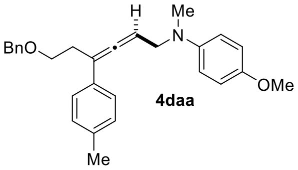

4daa

Prepared according to the general procedure D1 using **1d** (55.9 mg, 300 µmol, 1.0 equiv.), 4-methoxy-*N*-methyl-aniline **2a** (53.5 mg, 390 µmol, 1.30 equiv.) and (4-methylphenyl) trifluoromethanesulfonate **3a** (93.7 mg, 390 µmol, 1.30 equiv.). The crude material was purified by column chromatography (20 – 50 % (v/v) EtOAc in hexane) to give **4daa** (23.1 mg, 55.8 µmol, 19% yield) as a yellow oil.

R_f(50 % EtOAc/hexane) = 0.52.

^{1}H NMR (400 MHz, $CDCl_3$) δ 7.37 – 7.27 (m, 5H, Ar*H*), 7.20 (d, *J* = 8.2 Hz, 2H, Ar*H*), 7.09 (d, *J* = 8.0 Hz, 2H, Ar*H*), 6.86 – 6.77 (m, 2H, Ar*H*), 6.78 – 6.70 (m, 2H, Ar*H*), 5.48 (dt, *J* = 6.1, 3.1 Hz, 1H, C=C*H*), 4.47 (s, 2H, PhCH_2O), 4.04 – 3.87 (m, 2H, NCH_2), 3.75 (s, 3H, OCH_3), 3.56 (t, *J* = 7.3 Hz, 2H, OCH_2CH$_2$), 2.86 (s, 3H, NCH_3), 2.68 (td, *J* = 7.3, 3.0 Hz, 2H, OCH$_2$CH_2), 2.32 (s, 3H, ArCH_3). O*H* was not resolved.

^{13}C{^{1}H} NMR (101 MHz, $CDCl_3$) δ 204.3, 152.1, 143.9, 138.6, 136.6, 133.3, 129.2, 128.5, 127.8, 127.7, 126.0, 115.5, 114.8, 103.3, 90.4, 73.2, 69.1, 55.9, 53.3, 39.1, 30.4, 21.2.

HRMS (ESI/QTOF) m/z: $[M + H]^+$ Calcd for $C_{28}H_{32}NO_2^+$ 414.2428; Found 414.2431.

6-((4-Chlorophenyl)(methyl)amino)-3-(*p*-tolyl)hexa-3,4-dien-1-ol (4aba)

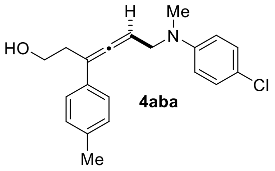

4aba

Prepared according to the general procedure D1 using hex-5-en-3-yn-1-ol **1a** (28.8 mg, 300 µmol, 1.00 equiv.) 4-chloro-*N*-methylaniline **2b** (55.2 mg, 390 µmol, 1.30 equiv.) and **3a** (93.7 mg, 390 µmol, 1.30 equiv.). The crude material was purified by column chromatography (15 – 50 % (v/v) hexanes in EtOAc) to give **4aba** (75.4 mg, 230 µmol, 77% yield) as a yellow oil.

R_f = 0.63 (40% EtOAc in pentane).

^{1}H NMR (400 MHz, CDCl$_3$) δ 7.21 – 7.06 (m, 6H, Ar*H*), 6.75 – 6.59 (m, 2H, Ar*H*), 5.54 (tt, *J* = 5.6, 2.8 Hz, 1H, C=C*H*), 4.09 – 3.94 (m, 2H, NC*H*$_2$), 3.72 (td, *J* = 6.4, 2.6 Hz, 2H, HOC*H*$_2$), 2.93 (s, 3H, NC*H*$_3$), 2.60 (tt, *J* = 6.3, 2.6 Hz, 2H, HOCH$_2$C*H*$_2$), 2.34 (s, 3H, ArC*H*$_3$).

^{13}C{^{1}H} NMR (101 MHz, CDCl$_3$) δ 204.1, 147.7, 137.0, 132.7, 129.3, 129.0, 126.0, 122.1, 114.6, 104.3, 90.6, 61.1, 52.3, 39.1, 33.5, 21.2.

IR (cm^{-1}) 3465 (w), 2949 (m), 2250 (w), 1947 (w), 1701 (s), 1604 (s), 1523 (m), 1434 (m), 1378 (m), 1283 (s), 1184 (s), 1112 (s), 1041 (m), 912 (m), 824 (m).

HRMS (nanochip-ESI/LTQ-Orbitrap) m/z: [M + H]$^+$ Calcd for $C_{20}H_{23}ClNO^+$ 328.1463; Found 328.1477.

Methyl 4-((6-hydroxy-4-(*p*-tolyl)hexa-2,3-dien-1-yl)(methyl)amino)benzoate (4aca)

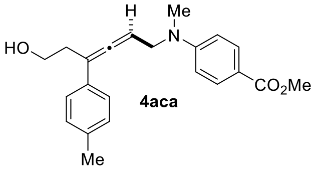

4aca

Prepared according to the general procedure D1 using hex-5-en-3-yn-1-ol **1a** (28.8 mg, 300 µmol, 1.00 equiv.) methyl 4-(methylamino)benzoate **2c** (64.4 mg, 390 µmol, 1.30 equiv.) and **3a** (93.7 mg, 390 µmol, 1.30 equiv.). The crude material was purified by column chromatography (15 – 50 % (v/v) hexanes in EtOAc) to give **4aca** (65.6 mg, 187 µmol, 62% yield) as a yellow oil.

R_f = 0.54 (40% EtOAc in pentane).

^{1}H NMR (400 MHz, CDCl$_3$) δ 7.90 – 7.85 (m, 2H, Ar*H*), 7.22 – 7.17 (m, 2H, Ar*H*), 7.13 – 7.06 (m, 2H, Ar*H*), 6.70 – 6.64 (m, 2H, Ar*H*), 5.55 (tt, *J* = 5.4, 2.8 Hz, 1H, C=C*H*), 4.18 – 4.05 (m, 2H, NC*H*$_2$), 3.86 (s, 3H, CO$_2$C*H*$_3$), 3.72 – 3.66 (m, 2H, HOC*H*$_2$), 3.01 (s, 3H, NC*H*$_3$), 2.64 – 2.52 (m, 2H, HOCH$_2$C*H*$_2$), 2.33 (s, 3H, ArC*H*$_3$).

^{13}C{^{1}H} NMR (101 MHz, CDCl$_3$) δ 204.0, 167.4, 152.1, 137.0, 132.5, 131.3, 129.2, 125.9, 117.6, 111.3, 104.8, 90.3, 61.0, 51.5, 51.4, 38.6, 33.4, 21.1.

IR (cm^{-1}) 3465 (w), 2949 (m), 2250 (w), 1947 (w), 1701 (s), 1604 (s), 1523 (m), 1434 (m), 1378 (m), 1283 (s), 1184 (s), 1112 (s), 1041 (m), 912 (m), 824 (m).

HRMS (nanochip-ESI/LTQ-Orbitrap) m/z: [M + H]$^+$ Calcd for $C_{22}H_{26}NO_3^+$ 352.1907; Found 352.1921.

6-(Methyl(*p*-tolyl)amino)-3-(*p*-tolyl)hexa-3,4-dien-1-ol (4ada)

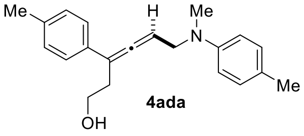

4ada

Prepared according to the general procedure D1 using hex-5-en-3-yn-1-ol **1a** (28.8 mg, 300 µmol, 1.00 equiv.) *N*-methyl-*p*-toluidine **2d** (47.3 mg, 390 µmol, 1.30 equiv.) and **3a** (93.7 mg, 390 µmol, 1.30 equiv.). The crude material was purified by column chromatography (15 – 50 % (v/v) hexanes in EtOAc) to give **4ada** (74.4 mg, 242 µmol, 81% yield) as a yellow oil. The reaction could be run on 3.0 mmol scale using hex-5-en-3-yn-1-ol **1a** (288 mg, 3.00 mmol, 1.00 equiv.) *N*-methyl-*p*-toluidine **2d** (473 mg, 3.90 mmol, 1.30 equiv.) and **3a** (937 mg, 3.90 mmol, 1.30 equiv.), purified as above to afford **4ada** (747 mg, 2.43 mmol, 81%).

R_f = 0.47 (25% EtOAc in pentane).

^{1}H NMR (400 MHz, CDCl$_3$) δ 7.26 – 7.21 (m, 2H, Ar*H*), 7.16 – 7.10 (m, 2H, Ar*H*), 7.09 – 7.02 (m, 2H, Ar*H*), 6.78 – 6.71 (m, 2H, Ar*H*), 5.59 (tt, *J* = 5.8, 2.9 Hz, 1H, C=C*H*), 4.09 – 3.96 (m, 2H, NC*H*$_2$), 3.81 – 3.66 (m, 2H, HOC*H*$_2$), 2.93 (s, 3H, NC*H*$_3$), 2.63 (td, *J* = 6.3, 2.9 Hz, 2H, HOCH$_2$C*H*$_2$), 2.36 (s, 3H, ArC*H*$_3$), 2.28 (s, 3H, ArC*H*$_3$).

^{13}C{^{1}H} NMR (101 MHz, CDCl$_3$) δ 204.0, 147.2, 136.8, 133.0, 129.8, 129.2, 127.0, 126.0, 114.3, 103.8, 91.1, 61.0, 52.8, 39.1, 33.4, 21.2, 20.4.

IR (cm^{-1}) 3347 (m), 3005 (m), 2922 (m), 2872 (m), 1944 (w), 1616 (m), 1516 (s), 1358 (m), 1110 (m), 1037 (s), 914 (m), 821 (m).

HRMS (ESI/QTOF) m/z: [M + H]$^+$ Calcd for $C_{21}H_{26}NO^+$ 308.2009; Found 308.2014.

Methyl 4-(1-hydroxy-6-((4-methoxyphenyl)(methyl)amino)hexa-3,4-dien-3-yl)benzoate (4aab)

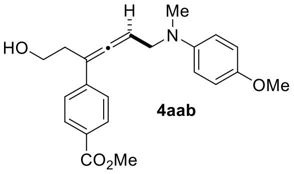

4aab

Prepared according to the general procedure D1 using hex-5-en-3-yn-1-ol **1a** (28.8 mg, 300 µmol, 1.0 equiv.), 4-methoxy-*N*-methyl-aniline **2a** (53.5 mg, 390 µmol, 1.30 equiv.) and methyl 4-(trifluoromethylsulfonyloxy)benzoate **3b** (111 mg, 390 µmol, 1.30 equiv.). The crude material was purified by column chromatography (20 – 50 % (v/v) EtOAc in hexane) to give **4aab** (86.1 mg, 234 µmol, 78% yield) as a yellow oil.

R_f(50% EtOAc/hexane) = 0.52.

^{1}H NMR (400 MHz, $CDCl_3$) δ 7.93 (d, *J* = 8.5 Hz, 2H, Ar*H*), 7.33 (d, *J* = 8.4 Hz, 2H, Ar*H*), 6.78 (s, 4H, Ar*H*), 5.65 (tt, *J* = 5.6, 2.9 Hz, 1H, C=C*H*), 3.98 (d, *J* = 5.6 Hz, 2H, NCH_2), 3.91 (s, 3H, CO_2CH_3), 3.81 – 3.74 (m, 2H, OCH_2), 3.73 (s, 3H, OCH_3), 2.86 (s, 3H, NCH_3), 2.63 (tt, *J* = 6.1, 2.6 Hz, 2H, CCH_2). O*H* was not resolved.

$^{13}C\{^{1}H\}$ NMR (101 MHz, $CDCl_3$) δ 205.3, 167.0, 152.9, 143.9, 141.0, 129.8, 128.5, 125.9, 116.7, 114.8, 103.9, 91.9, 60.8, 55.8, 53.3, 52.2, 40.3, 33.2.

IR (cm^{-1}) 3416 (w), 2953 (w), 1944 (w), 1720 (s), 1608 (m), 1512 (s).

HRMS (ESI/QTOF) m/z: $[M + H]^+$ Calcd for $C_{22}H_{26}NO_4^+$ 368.1856; Found 368.1865.

Methyl 4-(1-hydroxy-6-(methyl(4-(trifluoromethyl)phenyl)amino)hexa-3,4-dien-3-yl)benzoate (4aeb)

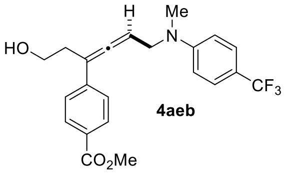

Prepared according to the general procedure D1 using **1a** (28.8 mg, 300 µmol, 1.0 equiv.), *N*-methyl-4-(trifluoromethyl)aniline **2e** (53.5 mg, 390 µmol, 1.30 equiv.) and methyl 4-(trifluoromethylsulfonyloxy)benzoate **3b** (111 mg, 390 µmol, 1.30 equiv.). The crude material was purified by column chromatography (20 – 50 % (v/v) EtOAc in hexane) to give **4aeb** (45.9 mg, 113 µmol, 38% yield) as a yellow oil.

R_f(50% EtOAc/hexane) = 0.32.

^{1}H NMR (400 MHz, $CDCl_3$) δ 7.97 – 7.89 (m, 2H, Ar*H*), 7.39 (d, *J* = 8.7 Hz, 2H, Ar*H*), 7.33 (d, *J* = 8.4 Hz, 2H, Ar*H*), 6.71 (d, *J* = 8.8 Hz, 2H, Ar*H*), 5.63 (tt, *J* = 5.4, 2.8 Hz, 1H, =C*H*), 4.21 – 4.05 (m, 2H, NCH_2), 3.91 (s, 3H, CO_2CH_3), 3.72 (t, *J* = 6.2 Hz, 2H, OCH_2), 2.99 (s, 3H, NCH_3), 2.60 (qd, *J* = 6.3, 2.8 Hz, 2H, CCH_2). O*H* was not resolved.

$^{13}C\{^{1}H\}$ NMR (101 MHz, $CDCl_3$) δ 205.2, 167.0, 151.0, 140.6, 129.9, 128.8, 126.6 (q, *J* = 3.8 Hz), 126.0, 125.1 (q, *J* = 270.1 Hz), 118.4 (q, *J* = 32.7 Hz), 112.0, 104.7, 91.1, 61.0, 52.2, 51.3, 38.8, 33.2.

^{19}F NMR (376 MHz, $CDCl_3$) δ -61.0.

IR (cm^{-1}) 3410 (w), 2954 (w), 2881 (w), 1941 (w), 1713 (m), 1615 (m), 1327 (s).

HRMS (ESI/QTOF) m/z: $[M + H]^+$ Calcd for $C_{22}H_{23}F_3NO_3^+$ 406.1625; Found 406.1619.

Methyl 4-(1-hydroxy-6-(methyl(naphthalen-2-yl)amino)hexa-3,4-dien-3-yl)benzoate (4afb)

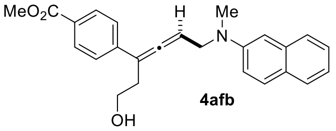

Prepared according to the general procedure D1 using **1a** (28.8 mg, 300 µmol, 1.0 equiv.), *N*-methylnaphthalen-2-amine **2f** (61.3 mg, 390 µmol, 1.30 equiv.) and methyl 4-(trifluoromethylsulfonyloxy)benzoate **3b** (111 mg, 390 µmol, 1.30 equiv.). The crude material was purified by column chromatography (20 – 50 % (v/v) EtOAc in hexane) to give **4afb** (57.1 mg, 147 µmol, 49% yield) as a yellow oil.

R_f(50% EtOAc/hexane) = 0.42.

^{1}H NMR (400 MHz, $CDCl_3$) δ 8.22 (d, *J* = 5.3 Hz, 1H, Ar*H*), 7.92 – 7.84 (m, 2H, Ar*H*), 7.82 – 7.73 (m, 1H, Ar*H*), 7.52 (d, *J* = 8.2 Hz, 1H, Ar*H*), 7.43 (dt, *J* = 6.8, 3.9 Hz, 2H, Ar*H*), 7.37 (t, *J* = 7.8 Hz, 1H, Ar*H*), 7.24 (d, *J* = 8.4 Hz, 2H, Ar*H*), 7.15 (d, *J* = 7.4 Hz, 1H, Ar*H*), 5.75 (tt, *J* = 5.9, 2.9 Hz, 1H, C=C*H*), 4.01 – 3.92 (m, 2H, NCH_2), 3.91 (s, 3H, CH_3), 3.84 – 3.65 (m, 2H, OCH_2), 2.95 (s, 3H, CH_3), 2.62 (tt, *J* = 6.1, 3.0 Hz, 2H, OCH_2CH_2), 1.63 (br s, 1H, O*H*).

$^{13}C\{^{1}H\}$ NMR (101 MHz, $CDCl_3$) δ 205.5, 167.1, 148.8, 141.0, 135.0, 129.7, 129.3, 128.54, 128.45, 126.0, 125.8, 125.7, 124.0, 123.8, 116.0, 103.7, 92.7, 60.8, 56.0, 52.2, 42.6, 33.1.

IR (cm^{-1}) 3404 (m), 2961 (w), 2814 (w), 2340 (m), 1930 (w), 1720 (s), 1279 (s).

HRMS (nanochip-ESI/LTQ-Orbitrap) m/z: $[M + H]^+$ Calcd for $C_{25}H_{26}NO_3^+$ 388.1907; Found 388.1916.

Methyl 4-(6-((3-(*tert*-butyl)phenyl)(methyl)amino)-1-hydroxyhexa-3,4-dien-3-yl)benzoate (4agb)

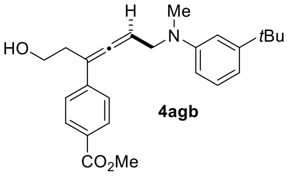

Prepared according to the general procedure D1 using **1a** (28.8 mg, 300 µmol, 1.0 equiv.), 3-*tert*-butyl-*N*-methylaniline **2g** (63.7 mg, 390 µmol, 1.30 equiv.) and methyl 4-(trifluoromethylsulfonyloxy)benzoate **3b** (111 mg, 390 µmol, 1.30 equiv.). The crude material was purified by column chromatography (20 – 50 % (v/v) EtOAc in hexane) to give **4agb** (94.3 mg, 240 µmol, 80% yield) as a yellow oil.

R_f(50% EtOAc/hexane) = 0.42.

^{1}H NMR (400 MHz, $CDCl_3$) δ 7.93 (d, *J* = 8.5 Hz, 2H, Ar*H*), 7.35 (d, *J* = 8.4 Hz, 2H, Ar*H*), 7.16 (t, *J* = 8.2 Hz, 1H, Ar*H*), 6.83 – 6.76 (m, 2H, Ar*H*), 6.66 – 6.58 (m, 1H, Ar*H*), 5.68 (tt, *J* = 5.5, 2.8 Hz, 1H, C=C*H*), 4.18 – 4.01 (m, 2H, NCH_2), 3.91 (s, 3H, CO_2CH_3), 3.70 (tq, *J* = 8.6, 4.6 Hz, 2H, HOCH_2), 2.94 (s, 3H, NCH_3), 2.61 (td, *J* = 6.3, 2.9 Hz, 2H, $HOCH_2CH_2$), 1.72 (s, 1H, O*H*), 1.29 (s, 9H, $C(CH_3)_3$).

$^{13}C\{^{1}H\}$ NMR (101 MHz, $CDCl_3$) δ 205.1, 167.0, 152.4, 149.0, 141.1, 129.8, 129.0, 128.5, 126.0, 115.1, 111.1, 111.0, 104.0, 92.0, 61.0, 52.2, 52.1, 39.1, 34.9, 33.2, 31.5.

IR (cm^{-1}) 3457 (w), 2953 (m), 2871 (w), 2811 (w), 1944 (w), 1720 (s), 1602 (s).

HRMS (ESI/QTOF) m/z: $[M + H]^+$ Calcd for $C_{25}H_{32}NO_3^+$ 394.2377; Found 394.2378.

Methyl 4-(6-((3,4-dihydro-2H-benzo[b][1,4]dioxepin-7-yl)(methyl)amino)-1-hydroxyhexa-3,4-dien-3-yl)benzoate (4ahb)

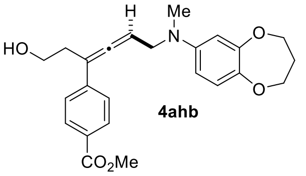

Prepared according to the general procedure D1 using **1a** (28.8 mg, 300 µmol, 1.0 equiv.), *N*-methyl-3,4-dihydro-2*H*-1,5-benzodioxepin-7-amine **2h** (69.9 mg, 390 µmol, 1.30 equiv.) and methyl 4-(trifluoromethylsulfonyloxy)benzoate **3b** (111 mg, 390 µmol, 1.30 equiv.). The crude material was purified by column chromatography (20 – 50 % (v/v) EtOAc in hexane) to give **4ahb** (92.6 mg, 226 µmol, 75% yield) as a yellow oil.

R_f(50% EtOAc/hexane) = 0.23.

^{1}H NMR (400 MHz, $CDCl_3$) δ 7.98 – 7.91 (m, 2H, Ar*H*), 7.41 – 7.33 (m, 2H, Ar*H*), 6.85 (d, *J* = 8.8 Hz, 1H, Ar*H*), 6.44 (d, *J* = 3.0 Hz, 1H, Ar*H*), 6.36 (dd, *J* = 8.8, 3.0 Hz, 1H, Ar*H*), 5.62 (tt, *J* = 5.6, 2.8 Hz, 1H, C=C*H*), 4.20 – 4.04 (m, 4H, OCH_2 x2), 4.01 (d, *J* = 5.6 Hz, 2H, CH_2N), 3.91 (s, 3H, CO_2CH_3), 3.74 (tt, *J* = 11.0, 5.5 Hz, 2H, CH_2), 2.88 (s, 3H, NCH_3), 2.63 (dtt, *J* = 6.4, 2.8, 1.6 Hz, 2H, CH_2), 2.13 (dtt, *J* = 9.1, 4.7, 2.7 Hz, 2H, CH_2), 1.94 (s, 1H, O*H*).

$^{13}C\{^1H\}$ NMR (101 MHz, $CDCl_3$) δ 205.2, 167.0, 152.0, 145.8, 143.3, 141.1, 130.0, 128.5, 125.9, 122.2, 109.0, 107.1, 103.9, 91.6, 71.0, 70.9, 60.9, 52.4, 52.2, 39.4, 33.2, 32.6.

IR (cm^{-1}) 3422 (m), 2957 (m), 2863 (w), 1944 (w), 1720 (s), 1605 (m).

HRMS (ESI/QTOF) m/z: $[M + H]^+$ Calcd for $C_{24}H_{28}NO_5^+$ 410.1962; Found 410.1964.

Methyl 4-(1-hydroxy-6-(methyl(o-tolyl)amino)hexa-3,4-dien-3-yl)benzoate (4aib)

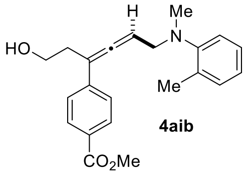

Prepared according to the general procedure D1 using hex-5-en-3-yn-1-ol **1a** (28.8 mg, 300 µmol, 1.0 equiv.) *N*-methyl-o-toluidine **2i** (47.3 mg, 48.4 µL, 390 µmol, 1.30 equiv.) and **3b** (111 mg, 390 µmol, 1.30 equiv.). The crude material was purified by column chromatography (15 – 50 % (v/v) hexanes in EtOAc) to give **4aib** (50.7 mg, 144 µmol, 48% yield) as a yellow oil.

R_f = 0.56 (50% EtOAc in pentane).

^{1}H NMR (400 MHz, $CDCl_3$) δ 7.97 – 7.83 (m, 2H, Ar*H*), 7.31 – 7.22 (m, 2H, Ar*H*), 7.21 – 7.03 (m, 3H, Ar*H*), 6.97 (td, *J* = 7.3, 1.5 Hz, 1H, Ar*H*), 5.68 (tt, *J* = 6.1, 3.0 Hz, 1H, C=C*H*), 3.90 (s, 3H, CO_2CH_3), 3.81 – 3.73 (m, 2H, NCH_2), 3.73 – 3.67 (m, 2H, $HOCH_2$), 2.75 (s, 3H, NCH_3), 2.69 – 2.60 (m, 2H, $HOCH_2CH_2$), 2.28 (s, 3H, $ArCH_3$).

$^{13}C\{^1H\}$ NMR (101 MHz, $CDCl_3$) δ 205.4, 167.1, 150.7, 141.1, 133.1, 131.5, 129.8, 128.4, 126.5, 125.8, 123.5, 120.1, 103.3, 92.8, 60.8, 55.0, 52.2, 42.0, 33.0, 18.4.

IR (cm^{-1}) 3417 (w), 2947 (m), 1943 (w), 1717 (s), 1604 (m), 1492 (m), 1436 (m), 1278 (s), 1185 (m), 1108 (s), 912 (m), 860 (w).

HRMS (ESI/QTOF) m/z: $[M + H]^+$ Calcd for $C_{22}H_{26}NO_3^+$ 352.1907; Found 352.1911.

Methyl 4-(1-hydroxy-6-((4-methoxyphenyl)(phenethyl)amino)hexa-3,4-dien-3-yl)benzoate (4ajb)

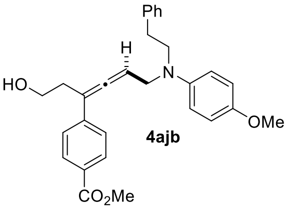

Prepared according to the general procedure D1 using **1a** (28.8 mg, 300 µmol, 1.0 equiv.), 4-methoxy-*N*-(2-phenylethyl)aniline **2j** (88.7 mg, 390 µmol, 1.30 equiv.) and methyl 4-(trifluoromethylsulfonyloxy)benzoate **3b** (111 mg, 390 µmol, 1.30 equiv.). The crude material was purified by column chromatography (20 – 50 % (v/v) EtOAc in hexane) to give **4ajb** (118.4 mg, 259 µmol, 86% yield) as a yellow oil.

R_f(50% EtOAc/hexane) = 0.39.

^{1}H NMR (400 MHz, $CDCl_3$) δ 7.88 – 7.80 (m, 2H, ArH), 7.24 (d, *J* = 8.5 Hz, 2H, Ar*H*), 7.17 – 7.06 (m, 3H, Ar*H*), 7.03 – 6.96 (m, 2H, Ar*H*), 6.79 – 6.67 (m, 4H, Ar*H*), 5.54 (tt, *J* = 5.4, 2.8 Hz, 1H, C=C*H*), 3.92 – 3.76 (m, 5H, CH_3, NCH_2C=C), 3.75 – 3.67 (m, 2H, $OHCH_2$), 3.66 (s, 3H, CH_3), 3.41 – 3.28 (m, 2H, CH_2, NCH_2), 2.71 (dd, *J* = 9.0, 6.7 Hz, 2H, C=CCH_2), 2.57 (qdq, *J* = 12.2, 5.8, 2.8 Hz, 2H, $PhCH_2$), 2.29 (s, 1H, O*H*).

$^{13}C\{^1H\}$ NMR (101 MHz, $CDCl_3$) δ 205.2, 167.0, 153.1, 142.3, 141.0, 139.8, 129.8, 128.8, 128.6, 128.5, 126.3, 126.0, 117.6, 114.9, 104.1, 92.8, 60.8, 55.8, 55.0, 52.2, 51.7, 33.8, 33.2.

IR (cm^{-1}) 3435 (w), 2950 (m), 2838 (w), 1944 (w), 1716 (s), 1605 (m), 1510 (s).

HRMS (ESI/QTOF) m/z: $[M + H]^+$ Calcd for $C_{29}H_{32}NO_4^+$ 458.2326; Found 458.2318.

Methyl 4-(1-hydroxy-6-((2-hydroxyethyl)(phenyl)amino)hexa-3,4-dien-3-yl)benzoate (4akb)

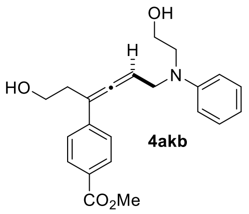

Prepared according to the general procedure D1 using hex-5-en-3-yn-1-ol **1a** (28.8 mg, 300 µmol, 1.0 equiv.) 2-anilinoethanol **2k** (53.5 mg, 48.6 µL, 390 µmol, 1.30 equiv.) and **3b** (111 mg, 390 µmol, 1.30 equiv.). The crude material was purified by column chromatography (25 – 100 % (v/v) hexanes in EtOAc) to give **4akb** (79.8 mg, 217 µmol, 72% yield) as a yellow oil.

R_f (EtOAc) = 0.57.

^{1}H NMR (400 MHz, $CDCl_3$) δ 7.92 (d, J = 8.5 Hz, 2H, ArH), 7.32 (d, J = 8.5 Hz, 2H, ArH), 7.19 (dd, J = 8.7, 7.3 Hz, 2H, ArH), 6.80 (d, J = 8.1 Hz, 2H, ArH), 6.73 (t, J = 7.3 Hz, 1H, ArH), 5.67 (tt, J = 5.3, 2.7 Hz, 1H, C=CH), 4.20 – 4.01 (m, 2H, NCH_2), 3.90 (s, 3H, CO_2CH_3), 3.79 – 3.62 (m, 4H, HOCH_2), 3.55 – 3.37 (m, 2H, $HOCH_2CH_2N$), 2.67 – 2.53 (m, 2H, $HOCH_2CH_2$), 2.41 (br s, 2H, HO).

$^{13}C\{^{1}H\}$ NMR (101 MHz, $CDCl_3$) δ 205.4, 167.0, 148.1, 140.9, 129.8, 129.4, 128.5, 126.0, 117.9, 113.9, 104.2, 92.1, 60.7, 60.3, 54.1, 52.2, 50.6, 33.2.

IR (cm^{-1}) 3388 (m), 2952 (m), 2880 (m), 1941 (w), 1715 (s), 1600 (s), 1503 (s), 1436 (m), 1279 (s), 1186 (m), 1110 (s), 1037 (s), 911 (m), 860 (m).

HRMS (ESI/QTOF) m/z: $[M + H]^+$ Calcd for $C_{22}H_{26}NO_4^+$ 368.1856; Found 368.1857.

Methyl 4-(1-hydroxy-6-(phenyl(3-phenylprop-2-yn-1-yl)amino)hexa-3,4-dien-3-yl)benzoate (4alb)

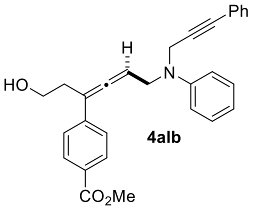

4alb

Prepared according to the general procedure D1 using **1a** (28.8 mg, 300 µmol, 1.0 equiv.), N-(3-phenylprop-2-ynyl)aniline **2l** (80.8 mg, 390 µmol, 1.30 equiv.) and methyl 4-(trifluoromethylsulfonyloxy)benzoate **3b** (111 mg, 390 µmol, 1.30 equiv.). The crude material was purified by column chromatography (20 – 50 % (v/v) EtOAc in hexane) to give **4alb** (30.4 mg, 69.5 µmol, 23% yield) as a yellow oil.

R_f (50 % EtOAc/hexane) = 0.48.

^{1}H NMR (400 MHz, $CDCl_3$) δ 7.85 (d, J = 8.5 Hz, 2H, ArH), 7.31 (d, J = 8.4 Hz, 2H, ArH), 7.29 – 7.14 (m, 7H, ArH), 6.88 (d, J = 8.1 Hz, 2H, ArH), 6.75 (t, J = 7.3 Hz, 1H, ArH), 5.70 (tt, J = 5.6, 2.8 Hz, 1H, C=CH), 4.19 (s, 2H, NCH_2CC), 4.14 (dd, J = 5.7, 3.0 Hz, 2H, NCH_2C=C), 3.83 (s, 3H, CO_2CH_3), 3.69 (tt, J = 10.8, 5.4 Hz, 2H, OHCH_2), 2.60 (qt, J = 6.3, 3.1 Hz, 2H, $OHCH_2CH_2$). OH was not resolved.

$^{13}C\{^{1}H\}$ NMR (101 MHz, $CDCl_3$) δ 205.4, 167.0, 148.0, 141.0, 131.8, 129.8, 129.3, 128.6, 128.4 (2C), 126.0, 122.9, 119.1, 115.4, 104.2, 92.5, 85.2, 84.7, 61.0, 52.2, 50.3, 41.9, 33.2.

IR (cm^{-1}) 3400 (w), 2947 (m), 2255 (w), 1943 (w), 1713 (s), 1602 (m), 1502 (m).

HRMS (nanochip-ESI/LTQ-Orbitrap) m/z: $[M + H]^+$ Calcd for $C_{29}H_{28}NO_3^+$ 438.2064; Found 438.2069.

Methyl 4-(6-(benzyl(phenyl)amino)-1-hydroxyhexa-3,4-dien-3-yl)benzoate (4amb)

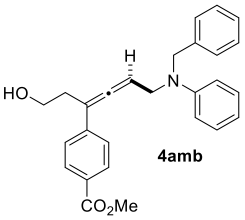

4amb

Prepared according to the general procedure D1 using hex-5-en-3-yn-1-ol **1a** (28.8 mg, 300 µmol, 1.0 equiv.), N-benzylaniline **2m** (71.5 mg, 390 µmol, 1.30 equiv.) and methyl 4-(trifluoromethylsulfonyloxy)benzoate **3b** (111 mg, 390 µmol, 1.30 equiv.). The crude material was purified by column chromatography (20 – 50 % (v/v) EtOAc in hexane) to give **4amb** (83.2 mg, 201 µmol, 67% yield) as a yellow oil.

R_f (50% EtOAc/hexane) = 0.52.

^{1}H NMR (400 MHz, $CDCl_3$) δ 7.95 (d, J = 8.5 Hz, 2H, ArH), 7.37 (d, J = 8.4 Hz, 2H, ArH), 7.34 – 7.14 (m, 7H, ArH), 6.81 – 6.68 (m, 3H, ArH), 5.71 (tt, J = 5.5, 2.7 Hz, 1H, C=CH), 4.53 (s, 2H, NCH_2Ph), 4.15 (d, J = 5.6 Hz, 2H, NCH_2), 3.92 (s, 3H, CO_2CH_3), 3.74 – 3.66 (m, 2H, OHCH_2), 2.63 (td, J = 6.4, 2.8 Hz, 2H, $OHCH_2CH_2$). OH was not resolved.

$^{13}C\{^{1}H\}$ NMR (101 MHz, $CDCl_3$) δ 205.3, 167.0, 148.5, 141.0, 138.7, 129.9, 129.4, 128.7, 128.6, 127.1, 126.9, 126.0, 117.4, 113.3, 104.2, 92.0, 61.1, 55.0, 52.2, 49.8, 33.3.

IR (cm^{-1}) 3399 (w), 2954 (w), 2863 (w), 1943 (w), 1718 (s), 1601 (s), 1504 (m).

HRMS (ESI/QTOF) m/z: $[M + H]^+$ Calcd for $C_{27}H_{28}NO_3^+$ 414.2064; Found 414.2070.

Methyl 4-(1-hydroxy-6-((4-methoxybenzyl)(phenyl)amino)hexa-3,4-dien-3-yl)benzoate (4anb)

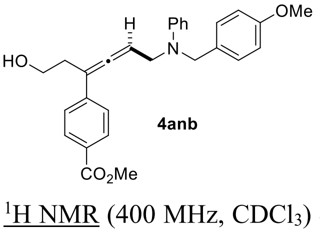

4anb

Prepared according to the general procedure D1 using **1a** (28.8 mg, 300 µmol, 1.0 equiv.), N-[(4-methoxyphenyl)methyl]aniline **2n** (83.2 mg, 390 µmol, 1.30 equiv.) and methyl 4-(trifluoromethylsulfonyloxy)benzoate **3b** (111 mg, 390 µmol, 1.30 equiv.). The crude material was purified by column chromatography (20 – 50 % (v/v) EtOAc in hexane) to give **4anb** (92.0 mg, 207 µmol, 69% yield) as a yellow oil.

R_f (50% EtOAc/hexane) = 0.42.

^{1}H NMR (400 MHz, $CDCl_3$) δ 7.99 – 7.90 (m, 2H, ArH), 7.39 – 7.34 (m, 2H, ArH), 7.22 – 7.10 (m, 4H, ArH), 6.80 (ddd, J = 17.5, 7.7, 1.4 Hz, 4H, ArH), 6.72 (t, J = 7.3 Hz, 1H, ArH), 5.68 (tt, J = 5.5, 2.8 Hz, 1H, C=CH), 4.46 (s, 2H, ArCH_2), 4.12 (d, J = 5.6 Hz, 2H, NCH_2), 3.92 (s, 3H, CO_2CH_3), 3.78 (s, 3H, OCH_3), 3.72 (d, J = 10.0 Hz, 2H, CH_2OH), 2.62 (td, J = 6.4, 2.8 Hz, 2H, $OHCH_2CH_2$), 1.54 (s, 1H, OH).

$^{13}C\{^{1}H\}$ NMR (101 MHz, $CDCl_3$) δ 205.3, 167.0, 158.8, 148.5, 141.0, 130.5, 129.8, 129.4, 128.6, 128.1, 126.0, 117.5, 114.1, 113.5, 104.1, 92.1, 61.1, 55.4, 54.4, 52.2, 49.5, 33.3.

IR (cm^{-1}) 3442 (w), 2951 (w), 2845 (w), 1947 (w), 1717 (s), 1602 (s), 1507 (s).

HRMS (ESI/QTOF) m/z: $[M + H]^+$ Calcd for $C_{28}H_{30}NO_4^+$ 444.2169; Found 444.2176.

Methyl 4-(6-(3,4-dihydroquinolin-1(2*H*)-yl)-1-hydroxyhexa-3,4-dien-3-yl)benzoate (4aob)

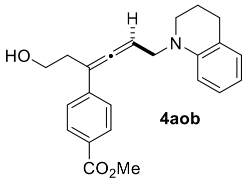

4aob

Prepared according to the general procedure D1 using hex-5-en-3-yn-1-ol **1a** (28.8 mg, 300 μmol, 1.0 equiv.), 1,2,3,4-tetrahydroquinoline **2o** (51.9 mg, 390 μmol, 1.30 equiv.) and methyl 4-(trifluoromethylsulfonyloxy)benzoate **3b** (111 mg, 390 μmol, 1.30 equiv.). The crude material was purified by column chromatography (20 – 50 % (v/v) EtOAc in hexane) to give **4aob** (89.8 mg, 247 μmol, 82% yield) as a yellow oil.

R_f(50 % EtOAc/hexane) = 0.38.

^{1}H NMR (400 MHz, $CDCl_3$) δ 7.99 – 7.91 (m, 2H, Ar*H*), 7.39 (d, *J* = 8.5 Hz, 2H, Ar*H*), 7.02 (td, *J* = 8.1, 1.4 Hz, 1H, Ar*H*), 6.94 (d, *J* = 7.3 Hz, 1H, Ar*H*), 6.65 (d, *J* = 8.2 Hz, 1H, Ar*H*), 6.60 (t, *J* = 7.3 Hz, 1H, Ar*H*), 5.67 (tt, *J* = 5.5, 2.8 Hz, 1H, C=C*H*), 4.14 – 3.94 (m, 2H, NCH_2), 3.91 (s, 3H, CO_2CH_3), 3.72 (q, *J* = 5.8 Hz, 2H, $HOCH_2$), 3.35 – 3.21 (m, 2H, NCH_2CH_2), 2.70 (td, *J* = 6.4, 3.2 Hz, 2H, $ArCH_2$), 2.62 (td, *J* = 6.4, 2.9 Hz, 2H, $HOCH_2CH_2$), 2.01 – 1.81 (m, 2H, NCH_2CH_2). O*H* was not resolved.

$^{13}C\{^1H\}$ NMR (101 MHz, $CDCl_3$) δ 205.0, 167.1, 144.7, 141.1, 129.8, 129.4, 128.5, 127.2, 126.0, 123.2, 116.7, 111.8, 104.1, 91.7, 61.1, 52.2, 50.3, 49.7, 33.2, 28.1, 22.2.

IR (cm^{-1}) 3416 (w), 2949 (w), 2846 (w), 1937 (w), 1717 (s), 1605 (m).

HRMS (ESI/QTOF) m/z: $[M + H]^+$ Calcd for $C_{23}H_{26}NO_3^+$ 364.1907; Found 364.1912.

Methyl 4-(1-hydroxy-6-(indolin-1-yl)hexa-3,4-dien-3-yl)benzoate (4apb)

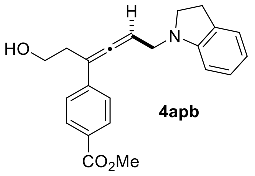

4apb

Prepared according to the general procedure D1 using **1a** (28.8 mg, 300 μmol, 1.0 equiv.), 2,3-dihydro-1*H*-indole **2p** (46.5 mg, 390 μmol, 1.30 equiv.) and methyl 4-(trifluoromethylsulfonyloxy)benzoate **3b** (111 mg, 390 μmol, 1.30 equiv.). The crude material was purified by column chromatography (20 – 50 % (v/v) EtOAc in hexane) to give **4apb** (59.7 mg, 171 μmol, 57% yield) as a yellow oil.

R_f(50% EtOAc/hexane) = 0.35.

^{1}H NMR (400 MHz, $CDCl_3$) δ 7.98 – 7.85 (m, 2H, Ar*H*), 7.40 – 7.31 (m, 2H, Ar*H*), 7.00 (dd, *J* = 7.2, 1.0 Hz, 1H, Ar*H*), 6.95 (t, *J* = 7.7 Hz, 1H, Ar*H*), 6.60 (td, *J* = 7.5, 0.8 Hz, 1H, Ar*H*), 6.46 (d, *J* = 7.8 Hz, 1H, Ar*H*), 5.64 (tt, *J* = 5.8, 2.9 Hz, 1H, C=C*H*), 3.90 – 3.65 (m, 7H, $HOCH_2$, CO_2CH_3, NCH_2CC), 3.38 – 3.24 (m, 2H, NCH_2CH_2), 2.87 (hept, *J* = 7.5 Hz, 2H, NCH_2CH_2), 2.59 (hd, *J* = 9.3, 2.9 Hz, 2H, $HOCH_2CH_2$). O*H* was not resolved.

$^{13}C\{^1H\}$ NMR (101 MHz, $CDCl_3$) δ 205.3, 167.0, 151.5, 141.0, 130.5, 129.9, 128.6, 127.4, 126.0, 124.7, 118.7, 108.3, 103.9, 91.8, 60.8, 53.6, 52.2, 48.4, 33.2, 28.7.

IR (cm^{-1}) 3462 (w), 2952 (w), 2856 (w), 1942 (w), 1718 (s), 1605 (m).

HRMS (nanochip-ESI/LTQ-Orbitrap) m/z: $[M + H]^+$ Calcd for $C_{22}H_{24}NO_3^+$ 350.1751; Found 350.1759.

3-(*p*-Tolyl)-6-((4-(trifluoromethyl)phenyl)amino)hexa-3,4-dien-1-ol (4aqa)

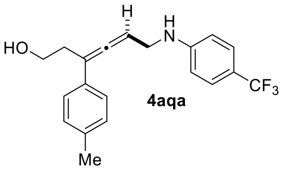

4aqa

Prepared according to the general procedure D1 using **1a** (28.9 mg, 0.300 mmol, 1.00 equiv.), 4-(trifluoromethyl)aniline **2q** (63.0 mg, 0.390 mmol, 1.30 equiv.) and (4-methylphenyl) trifluoromethanesulfonate **3a** (94.0 mg, 0.390 mmol, 1.30 equiv.). The crude material was purified by column chromatography (5 – 30 % (v/v) EtOAc in hexane) to give **4aqa** (64.9 mg, 0.186 mmol, 62%) as a pale-yellow solid. The reaction could be run on 5.0 mmol scale using hex-5-en-3-yn-1-ol **1a** (481 mg, 5.00 mmol, 1.00 equiv.) 1.05 g, **2q** (1.05 g, 3.90 mmol, 1.30 equiv.) and **3a** (1.56 g, 3.90 mmol, 1.30 equiv.), purified as above to afford **4aqa** (1.05 g, 3.01 mmol, 60%).

R_f(20 % EtOAc/hexane) = 0.22.

^{1}H NMR (400 MHz, $CDCl_3$) δ 7.39 – 7.30 (m, 2H, Ar*H*), 7.24 – 7.18 (m, 2H, Ar*H*), 7.14 – 7.07 (m, 2H, Ar*H*), 6.65 – 6.58 (m, 2H, Ar*H*), 5.72 (tt, *J* = 5.1, 2.8 Hz, 1H, C=C*H*), 4.53 (s, 1H, N*H*), 3.99 – 3.76 (m, 4H, CH_2OH and CH_2N), 2.77 – 2.61 (m, 2H, CH_2), 2.33 (s, 3H, $ArCH_3$), 1.67 (s, 1H, O*H*).

$^{13}C\{^1H\}$ NMR (101 MHz, $CDCl_3$) δ 203.0, 150.3, 137.2, 132.6, 129.3, 126.5 (q, *J* = 3.8 Hz), 126.0, 124.9 (q, $J_{C\text{-}F}$ = 270.1 Hz), 119.2 (q, $J_{C\text{-}F}$ = 32.5 Hz), 112.5, 106.2, 92.5, 60.7, 42.0, 33.5, 21.1.

^{19}F NMR (376 MHz, $CDCl_3$) δ -61.1.

IR (cm^{-1}) 3384 (w), 2933 (w), 2826 (w), 2367 (w), 1614 (m), 1528 (m), 1324 (s), 1105 (s).

HRMS (ESI/QTOF) m/z: $[M + H]^+$ Calcd for $C_{20}H_{21}F_3NO^+$ 348.1570; Found 348.1574.

6-((4-Fluorophenyl)amino)-3-(*p*-tolyl)hexa-3,4-dien-1-ol (4ara)

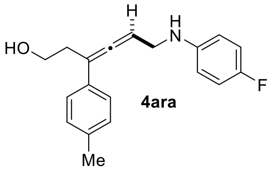

Prepared according to the general procedure D1 using hex-5-en-3-yn-1-ol **1a** (28.8 mg, 300 µmol, 1.00 equiv.) 4-fluoroaniline **2r** (43.3 mg, 390 µmol, 1.30 equiv.) and **3a** (93.7 mg, 390 µmol, 1.30 equiv.). The crude material was purified by column chromatography (15 – 50 % (v/v) hexanes in EtOAc) to give **4ara** (43.4 mg, 146 µmol, 49% yield) as a yellow oil.

R_f = 0.54 (30% EtOAc in pentane).

^{1}H NMR (400 MHz, $CDCl_3$) δ 7.26 – 7.22 (m, 2H, Ar*H*), 7.12 (d, *J* = 8.1 Hz, 2H, Ar*H*), 6.88 – 6.82 (m, 2H, Ar*H*), 6.61 – 6.55 (m, 2H, Ar*H*), 5.73 (tt, *J* = 5.0, 2.9 Hz, 1H, C=C*H*), 3.90 – 3.69 (m, 4H, NCH_2, $HOCH_2$), 2.76 – 2.58 (m, 2H, $HOCH_2CH_2$), 2.34 (s, 3H, $ArCH_3$). N*H* and O*H* were not resolved.

$^{13}C\{^1H\}$ NMR (101 MHz, $CDCl_3$) δ 202.9, 156.4 (d, $J_{C\text{-}F}$ = 235.7 Hz), 144.1 (d, $J_{C\text{-}F}$ = 1.9 Hz), 137.2, 132.9, 129.4, 126.1, 115.7 (d, $J_{C\text{-}F}$ = 22.3 Hz), 114.8 (d, $J_{C\text{-}F}$ = 7.5 Hz), 106.0, 93.5, 60.7, 43.5, 33.6, 21.2.

^{19}F NMR (376 MHz, $CDCl_3$) δ -126.8 (tt, *J* = 8.5, 4.4 Hz).

IR (cm^{-1}) 3567 (w), 3350 (m), 2920 (m), 2361 (w), 2337 (w), 2248 (w), 1942 (w), 1908 (w), 1597 (s), 1507 (s), 1487 (s), 1323 (m), 1258 (m), 1166 (w), 1077 (m), 1040 (m), 989 (m).

HRMS (nanochip-ESI/LTQ-Orbitrap) m/z: $[M + H]^+$ Calcd for $C_{19}H_{21}FNO^+$ 298.1602; Found 298.1607.

6-((3-Chlorophenyl)amino)-3-(*p*-tolyl)hexa-3,4-dien-1-ol (4asa)

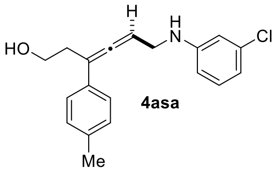

Prepared according to the general procedure D1 using hex-5-en-3-yn-1-ol **1a** (28.8 mg, 300 µmol, 1.00 equiv.) 3-chloroaniline **2s** (49.8 mg, 390 µmol, 1.30 equiv.) and **3a** (93.7 mg, 390 µmol, 1.30 equiv.). The crude material was purified by column chromatography (15 – 50 % (v/v) hexanes in EtOAc) to give **4asa** (23.3 mg, 74.2 µmol, 25% yield) as a yellow oil.

R_f = 0.51 (30% EtOAc in pentane).

^{1}H NMR (400 MHz, $CDCl_3$) δ 7.29 – 7.21 (m, 2H, Ar*H*), 7.17 – 7.09 (m, 2H, Ar*H*), 7.03 (t, *J* = 8.0 Hz, 1H, Ar*H*), 6.66 (ddd, *J* = 7.8, 2.0, 0.9 Hz, 1H, Ar*H*), 6.62 (t, *J* = 2.1 Hz, 1H, Ar*H*), 6.49 (ddd, *J* = 8.1, 2.3, 0.9 Hz, 1H, Ar*H*), 5.71 (tt, *J* = 5.1, 2.9 Hz, 1H, C=C*H*), 3.92 – 3.72 (m, 4H, NCH_2, $HOCH_2$), 2.78 – 2.59 (m, 2H, $HOCH_2CH_2$), 2.34 (s, 3H, $ArCH_3$). N*H* and O*H* were not resolved.

$^{13}C\{^1H\}$ NMR (101 MHz, $CDCl_3$) δ 203.0, 149.1, 137.2, 135.1, 132.8, 130.2, 129.4, 126.1, 117.7, 113.1, 111.9, 106.0, 93.0, 60.8, 42.5, 33.6, 21.2.

IR (cm^{-1}) 3670 (w), 3357 (w), 2969 (m), 2922 (m), 2360 (w), 1944 (w), 1600 (w), 1510 (s), 1222 (m), 1045 (m), 820 (m).

HRMS (nanochip-ESI/LTQ-Orbitrap) m/z: $[M + H]^+$ Calcd for $C_{19}H_{21}ClNO^+$ 314.1306; Found 314.1311.

6-((3-Methoxyphenyl)amino)-3-(*p*-tolyl)hexa-3,4-dien-1-ol (4ata)

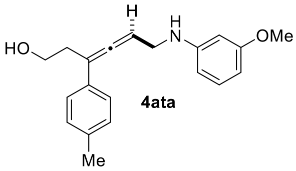

Prepared according to the general procedure D1 using hex-5-en-3-yn-1-ol **1a** (28.8 mg, 300 µmol, 1.00 equiv.) 3-methoxyaniline **2t** (48.0 mg, 390 µmol, 1.30 equiv.) and **3a** (93.7 mg, 390 µmol, 1.30 equiv.). The crude material was purified by column chromatography (15 – 50 % (v/v) hexanes in EtOAc) to give **4ata** (29.8 mg, 96.3 µmol, 32% yield) as a yellow oil.

R_f = 0.34 (30% EtOAc in pentane).

^{1}H NMR (400 MHz, $CDCl_3$) δ 7.31 – 7.23 (m, 2H, Ar*H*), 7.16 – 7.11 (m, 2H, Ar*H*), 7.06 (t, *J* = 8.1 Hz, 1H, Ar*H*), 6.28 (dddd, *J* = 9.0, 8.1, 2.3, 0.9 Hz, 2H, Ar*H*), 6.22 (t, *J* = 2.3 Hz, 1H, Ar*H*), 5.73 (tt, *J* = 5.2, 2.9 Hz, 1H, C=C*H*), 3.89 – 3.75 (m, 4H, NCH_2, $HOCH_2$), 3.74 (s, 3H, $ArOCH_3$), 2.77 – 2.58 (m, 2H, $HOCH_2CH_2$), 2.34 (s, 3H, $ArCH_3$). N*H* and O*H* were not resolved.

$^{13}C\{^1H\}$ NMR (101 MHz, $CDCl_3$) δ 202.9, 160.9, 149.2, 137.1, 133.0, 130.1, 129.3, 126.1, 106.8, 105.8, 103.3, 99.7, 93.4, 60.8, 55.2, 42.8, 33.6, 21.2.

IR (cm^{-1}) 3559 (w), 3378 (m), 3023 (m), 2932 (m), 2835 (m), 2360 (w), 2246 (w), 1944 (w), 1909 (w), 1609 (s), 1498 (s), 1460 (m), 1338 (m), 1209 (s), 1161 (s), 1039 (s), 821 (s).

HRMS (nanochip-ESI/LTQ-Orbitrap) m/z: $[M + H]^+$ Calcd for $C_{20}H_{24}NO_2^+$ 310.1802; Found 310.1810.

6-((2-Fluorophenyl)amino)-3-(*p*-tolyl)hexa-3,4-dien-1-ol (4aua)

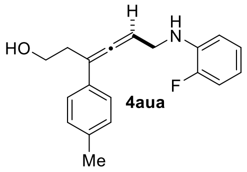

Prepared according to the general procedure D1 using hex-5-en-3-yn-1-ol **1a** (28.8 mg, 300 µmol, 1.0 equiv.) 2-fluoroaniline **2u** (43.3 mg, 390 µmol, 1.30 equiv.) and **3a** (93.7 mg, 390 µmol, 1.30 equiv.). The crude material was purified by column chromatography (15 – 50 % (v/v) hexanes in EtOAc) to give **4aua** (59.7 mg, 0.201 µmol, 67% yield) as a pale yellow oil.

R_f = 0.70 (50% EtOAc in pentane).

^{1}H NMR (400 MHz, $CDCl_3$) δ 7.25 (d, *J* = 8.2 Hz, 2H, Ar*H*), 7.12 (d, *J* = 8.0 Hz, 2H, Ar*H*), 7.02 – 6.89 (m, 2H, Ar*H*), 6.81 – 6.72 (m, 1H, Ar*H*), 6.69 – 6.60 (m, 1H, Ar*H*), 5.72 (tt, *J* = 5.4, 2.9 Hz, 1H, C=C*H*), 3.96 – 3.86 (m, 2H, NCH_2), 3.85 – 3.73 (m, 2H, HOCH_2), 2.68 (d, *J* = 2.9 Hz, 2H, HOCH$_2$CH_2), 2.35 (s, 3H, ArCH_3). N*H* and O*H* were not resolved.

^{13}C{^{1}H} NMR (101 MHz, $CDCl_3$) δ 203.1, 151.9 (d, $J_{C\text{-}F}$ = 238.7 Hz), 137.1, 136.2 (d, $J_{C\text{-}F}$ = 11.6 Hz), 132.9, 129.3, 126.1, 124.6 (d, $J_{C\text{-}F}$ = 3.5 Hz), 117.5 (d, $J_{C\text{-}F}$ = 7.0 Hz), 114.6 (d, $J_{C\text{-}F}$ = 18.4 Hz), 113.2 (d, $J_{C\text{-}F}$ = 3.3 Hz), 105.8, 93.1, 60.9, 42.5, 33.6, 21.2.

^{19}F NMR (376 MHz, $CDCl_3$) δ -135.6 – -136.1 (m).

IR (cm^{-1}) 3432 (m), 3023 (w), 2921 (m), 2877 (m), 1944 (w), 1619 (s), 1511 (s), 1453 (m), 1328 (m), 1255 (m), 1188 (m), 1112 (m), 1036 (s), 914 (m), 821 (m).

HRMS (ESI/QTOF) m/z: [M + H]$^+$ Calcd for $C_{19}H_{21}FNO^+$ 298.1602; Found 298.1608.

6-((2-(*tert*-Butyl)phenyl)amino)-3-(*p*-tolyl)hexa-3,4-dien-1-ol (4ava)

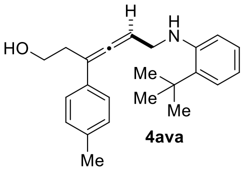

4ava

Prepared according to the general procedure D1 using hex-5-en-3-yn-1-ol **1a** (28.8 mg, 300 µmol, 1.00 equiv.) 2-*tert*-butylaniline **2v** (58.2 mg, 390 µmol, 1.30 equiv.) and **3a** (93.7 mg, 390 µmol, 1.30 equiv.). The crude material was purified by column chromatography (15 – 50 % (v/v) hexanes in EtOAc) to give **4ava** (81.7 mg, 244 µmol, 81% yield) as a yellow oil.

R_f = 0.63 (25% EtOAc in pentane).

^{1}H NMR (400 MHz, $CDCl_3$) δ 7.34 – 7.28 (m, 2H, Ar*H*), 7.23 (dd, *J* = 7.8, 1.6 Hz, 1H, Ar*H*), 7.17 – 7.11 (m, 3H, Ar*H*), 6.79 – 6.68 (m, 2H, Ar*H*), 5.88 (td, *J* = 4.6, 2.3 Hz, 1H, C=C*H*), 4.01 – 3.87 (m, 2H, NHCH_2), 3.87 – 3.78 (m, 2H, HOCH_2), 2.72 (td, *J* = 6.2, 3.1 Hz, 2H, HOCH$_2$CH_2), 2.36 (s, 3H, ArCH_3), 1.29 (s, 9H, C(CH_3)$_3$). N*H* and O*H* were not resolved.

^{13}C{^{1}H} NMR (101 MHz, $CDCl_3$) δ 202.2, 145.9, 137.2, 134.0, 132.8, 129.3, 127.2, 126.4, 126.0, 117.8, 112.9, 106.8, 94.0, 61.0, 43.2, 34.1, 33.4, 29.9, 21.2.

IR (cm^{-1}) 3456 (w), 2967 (m), 2875 (w), 2247 (w), 1946 (w), 1682 (w), 1507 (m), 1446 (m), 1309 (w), 1051 (m), 907 (s), 821 (w).

HRMS (nanochip-ESI/LTQ-Orbitrap) m/z: [M + H]$^+$ Calcd for $C_{23}H_{30}NO^+$ 336.2322; Found 336.2335.

6-((2-Methoxyphenyl)amino)-3-(p-tolyl)hexa-3,4-dien-1-ol (4awa)

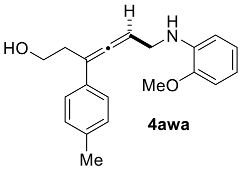

4awa

Prepared according to the general procedure D1 using hex-5-en-3-yn-1-ol **1a** (28.8 mg, 300 µmol, 1.00 equiv.) 2-methoxyaniline **2w** (48.0 mg, 390 µmol, 1.30 equiv.) and **3a** (93.7 mg, 390 µmol, 1.30 equiv.). The crude material was purified by column chromatography (15 – 50 % (v/v) hexanes in EtOAc) to give **4awa** (73.2 mg, 237 µmol, 79% yield) as an orange oil. The reaction could be run on 3.0 mmol scale using hex-5-en-3-yn-1-ol **1a** (288 mg, 3.00 mmol, 1.00 equiv.), 2-methoxyaniline **2w** (481 mg, 3.90 mmol, 1.30 equiv.) and **3a** (937 mg, 3.90 mmol, 1.30 equiv.), purified as above to afford **4awa** (696 mg, 2.25 mmol, 75%).

R_f = 0.33 (25% EtOAc in pentane).

^{1}H NMR (400 MHz, $CDCl_3$) δ 7.26 – 7.22 (m, 2H, Ar*H*), 7.14 – 7.08 (m, 2H, Ar*H*), 6.87 – 6.81 (m, 1H, Ar*H*), 6.74 (dd, *J* = 8.3, 1.6 Hz, 1H, Ar*H*), 6.72 – 6.65 (m, 2H, Ar*H*), 5.71 (tt, *J* = 5.4, 2.9 Hz, 1H, C=C*H*), 3.89 (d, *J* = 5.2 Hz, 2H, NHCH_2), 3.81 – 3.72 (m, 5H, HOCH_2, ArOCH_3), 2.73 – 2.58 (m, 2H, HOCH$_2$CH_2), 2.33 (s, 3H, ArCH_3). N*H* and O*H* were not resolved.

^{13}C{^{1}H} NMR (101 MHz, $CDCl_3$) δ 203.0, 147.3, 137.7, 136.9, 133.2, 129.3, 126.1, 121.4, 117.4, 111.2, 109.8, 105.6, 93.6, 61.0, 55.6, 42.7, 33.7, 21.2.

IR (cm^{-1}) 3414 (w), 2941 (w), 2837 (w), 2245 (w), 1944 (w), 1682 (w), 1601 (m), 1510 (s), 1457 (m), 1337 (w), 1248 (m), 1223 (m), 1178 (m), 1125 (m), 1029 (m), 907 (s).

HRMS (nanochip-ESI/LTQ-Orbitrap) m/z: [M + H]$^+$ Calcd for $C_{20}H_{24}NO_2^+$ 310.1802; Found 310.1815.

Methyl 4-(6-((2,6-dimethylphenyl)amino)-1-hydroxyhexa-3,4-dien-3-yl)benzoate (4axb)

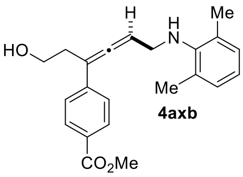

4axb

Prepared according to the general procedure D1 using **1a** (28.8 mg, 300 µmol, 1.0 equiv.), 2,6-dimethylaniline **2x** (47.3 mg, 390 µmol, 1.30 equiv.) and methyl 4-(trifluoromethylsulfonyloxy)benzoate **3b** (111 mg, 390 µmol, 1.30 equiv.). The crude material was purified by column chromatography (20 – 50 % (v/v) EtOAc in hexane) to give **4axb** (32.0 mg, 91.1 µmol, 30% yield) as a yellow oil.

R_f(50% EtOAc/hexane) = 0.29.

^{1}H NMR (400 MHz, $CDCl_3$) δ 7.98 (d, J = 8.3 Hz, 2H, ArH), 7.43 (d, J = 8.3 Hz, 2H, ArH), 6.99 (d, J = 7.4 Hz, 2H, ArH), 6.87 (t, J = 7.5 Hz, 1H, ArH), 5.84 (s, 1H, C=CH), 3.92 (s, 3H, CO_2CH_3), 3.89 – 3.62 (m, 4H, NCH_2, HOCH_2), 2.80 – 2.60 (m, 2H, $HOCH_2CH_2$), 2.25 (s, 6H, CH_3). NH and OH were not resolved.

^{13}C{^{1}H} NMR (101 MHz, $CDCl_3$) δ 203.5, 167.0, 144.7, 141.2, 130.4, 130.0, 129.1, 128.8, 125.9, 123.2, 105.9, 95.2, 60.4, 52.3, 46.3, 33.2, 18.4.

IR (cm^{-1}) 3413 (w), 2950 (w), 2845 (w), 1947 (w), 1716 (s), 1608 (m), 1472 (m).

HRMS (ESI/QTOF) m/z: $[M + H]^+$ Calcd for $C_{22}H_{26}NO_3^+$ 352.1907; Found 352.1912.

Methyl 4-(1-hydroxy-6-((2-methylnaphthalen-1-yl)amino)hexa-3,4-dien-3-yl)benzoate (4ayb)

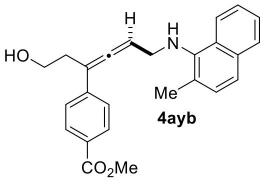

4ayb

Prepared according to the general procedure D1 using **1a** (28.8 mg, 300 µmol, 1.0 equiv.), 2-methylnaphthalen-1-amine **2y** (61.3 mg, 390 µmol, 1.30 equiv.) and methyl 4-(trifluoromethylsulfonyloxy)benzoate **3b** (111 mg, 390 µmol, 1.30 equiv.). The crude material was purified by column chromatography (20 – 50 % (v/v) EtOAc in hexane) to give **4ayb** (49.3 mg, 127 µmol, 42% yield) as a yellow oil.

R_f (50% EtOAc/hexane) = 0.29.

^{1}H NMR (400 MHz, $CDCl_3$) δ 8.03 (d, J = 8.3 Hz, 1H, ArH), 8.01 – 7.96 (m, 2H, ArH), 7.81 – 7.76 (m, 1H, ArH), 7.52 (d, J = 8.3 Hz, 1H, ArH), 7.48 – 7.36 (m, 4H, ArH), 7.28 (d, J = 8.4 Hz, 1H, ArH), 5.90 (tt, J = 5.4, 3.0 Hz, 1H, C=CH), 3.98 – 3.83 (m, 8H, NH, CO_2CH_3, NCH_2 and HOCH_{2a}), 3.76 (ddd, J = 11.1, 6.1, 4.7 Hz, 1H, HOCH_{2b}), 2.79 – 2.60 (m, 2H, $HOCH_2CH_2$), 2.44 (s, 3H, ArCH_3). OH was not resolved.

^{13}C{^{1}H} NMR (101 MHz, $CDCl_3$) δ 203.9, 167.0, 141.1, 140.7, 133.6, 130.1, 130.0, 129.4, 128.8, 128.6, 126.7, 126.0, 125.9, 125.2, 124.0, 122.4, 105.7, 94.9, 60.4, 52.3, 48.1, 33.2, 18.2.

IR (cm^{-1}) 3401 (w), 2953 (w), 2861 (w), 1941 (w), 1716 (s), 1605 (m).

HRMS (ESI/QTOF) m/z: $[M + H]^+$ Calcd for $C_{25}H_{26}NO_3^+$ 388.1907; Found 388.1910.

1-(4-(1-Hydroxy-6-((4-methoxyphenyl)(methyl)amino)hexa-3,4-dien-3-yl)phenyl)ethan-1-one (4aac)

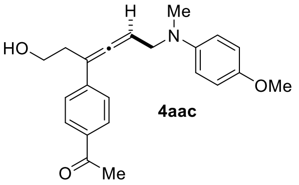

4aac

Prepared according to the general procedure D1 using hex-5-en-3-yn-1-ol **1a** (28.8 mg, 300 µmol, 1.0 equiv.), 4-methoxy-N-methyl-aniline **2a** (53.5 mg, 390 µmol, 1.30 equiv.) and **3c** (105 mg, 390 µmol, 1.30 equiv.). The crude material was purified by column chromatography (15 – 50 % (v/v) hexanes in EtOAc) to give **4aac** (96.8 mg, 275 µmol, 92% yield) as an orange oil.

R_f = 0.35 (50% EtOAc in pentane).

^{1}H NMR (400 MHz, $CDCl_3$) δ 7.90 – 7.76 (m, 2H, ArH), 7.38 – 7.30 (m, 2H, ArH), 6.78 (s, 4H, ArH), 5.65 (tt, J = 5.7, 2.9 Hz, 1H, C=CH), 3.97 (d, J = 5.7 Hz, 2H, NCH_2), 3.83 – 3.66 (m, 5H, OCH_3, HOCH_2), 2.86 (s, 3H, NCH_3), 2.66 – 2.59 (m, 2H, $HOCH_2CH_2$), 2.58 (s, 3H, NCH_3). OH was not resolved.

^{13}C{^{1}H} NMR (101 MHz, $CDCl_3$) δ 205.4, 197.7, 152.9, 143.8, 141.2, 135.5, 128.6, 126.1, 116.7, 114.8, 103.8, 91.8, 60.8, 55.7, 53.3, 40.3, 33.1, 26.7.

IR (cm^{-1}) 3439 (w), 2950 (m), 2901 (m), 1944 (w), 1677 (s), 1600 (m), 1512 (s), 1359 (m), 1269 (s), 1246 (s), 1038 (s), 914 (s), 818 (m).

HRMS (APCI/QTOF) m/z: $[M + H]^+$ Calcd for $C_{22}H_{26}NO_3^+$ 352.1907; Found 352.1895.

3-(4-(*tert*-Butyl)phenyl)-6-((4-methoxyphenyl)(methyl)amino)hexa-3,4-dien-1-ol (4aad)

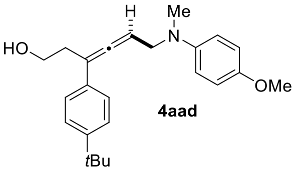

4aad

Prepared according to the general procedure D1 using hex-5-en-3-yn-1-ol **1a** (28.8 mg, 300 µmol, 1.0 equiv.), 4-methoxy-N-methyl-aniline **2a** (53.5 mg, 390 µmol, 1.30 equiv.) and **3d** (110 mg, 390 µmol, 1.30 equiv.). The crude material was purified by column chromatography (15 – 50 % (v/v) hexanes in EtOAc) to give **4aad** (81.0 mg, 222 µmol, 74% yield) as a yellow oil.

R_f = 0.55 (50% EtOAc in pentane).

^{1}H NMR (400 MHz, $CDCl_3$) δ 7.35 – 7.30 (m, 2H, ArH), 7.28 – 7.22 (m, 2H, ArH), 6.82 (s, 4H, ArH), 5.59 (tt, J = 5.8, 2.9 Hz, 1H, C=CH), 3.96 (d, J = 5.8 Hz, 2H, NCH_2), 3.81 – 3.70 (m, 5H, OCH_3, HOCH_2), 2.89 (s, 3H, NCH_3), 2.69 – 2.57 (m, 2H, $HOCH_2CH_2$), 1.32 (s, 9H, $C(CH_3)_3$). OH was not resolved.

^{13}C{^{1}H} NMR (101 MHz, $CDCl_3$) δ 204.3, 152.8, 150.1, 144.1, 132.9, 125.8, 125.5, 116.6, 114.8, 103.7, 91.2, 61.0, 55.8, 53.8, 40.0, 34.6, 33.4, 31.4.

IR (cm^{-1}) 3390 (w), 2959 (m), 2904 (m), 2833 (w), 1945 (w), 1511 (s), 1461 (m), 1244 (s), 1182 (m), 1112 (m), 1038 (s), 913 (m), 815 (m).

HRMS (APCI/QTOF) m/z: $[M + H]^+$ Calcd for $C_{24}H_{32}NO_2^+$ 366.2428; Found 366.2412.

3-(4-Chlorophenyl)-6-((4-methoxyphenyl)(methyl)amino)hexa-3,4-dien-1-ol (4aae)

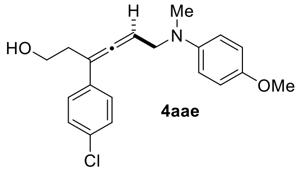

4aae

Prepared according to the general procedure D1 using hex-5-en-3-yn-1-ol **1a** (28.8 mg, 300 μmol, 1.0 equiv.), 4-methoxy-*N*-methyl-aniline **2a** (53.5 mg, 390 μmol, 1.30 equiv.) and **3e** (102 mg, 390 μmol, 1.30 equiv.). The crude material was purified by column chromatography (20 – 50 % (v/v) hexane in EtOAc) to give **4aae** (62.2 mg, 181 μmol, 60% yield) as a yellow oil.

R_f = 0.38 (50% EtOAc in pentane).

^{1}H NMR (400 MHz, CDCl$_3$) δ 7.25 – 7.20 (m, 2H, Ar*H*), 7.20 – 7.14 (m, 2H, Ar*H*), 6.78 (s, 4H, Ar*H*), 5.59 (tt, J = 5.7, 2.9 Hz, 1H, C=C*H*), 3.95 (d, J = 5.6 Hz, 2H, NCH_2), 3.73 (d, J = 7.9 Hz, 5H, OCH_3, HOCH_2), 2.86 (s, 3H, NCH_3), 2.61 – 2.53 (m, 2H, HOCH$_2$CH_2). O*H* was not resolved.

^{13}C{^{1}H} NMR (101 MHz, CDCl$_3$) δ 204.4, 152.9, 143.8, 134.6, 132.6, 128.5, 127.3, 116.7, 114.7, 103.4, 91.7, 60.7, 55.7, 53.4, 40.3, 33.2.

IR (cm^{-1}) 3378 (w), 2937 (m), 2835 (w), 1945 (w), 1511 (s), 1243 (s), 1182 (m), 1093 (m), 1036 (s), 816 (m).

HRMS (APCI/QTOF) m/z: [M + H]$^+$ Calcd for $C_{20}H_{23}ClNO_2^+$ 344.1412; Found 344.1400.

3-(4-Fluorophenyl)-6-((4-methoxyphenyl)(methyl)amino)hexa-3,4-dien-1-ol (4aaf)

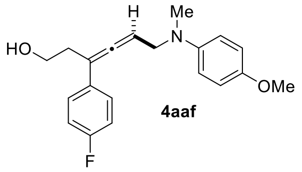

4aaf

Prepared according to the general procedure D1 using hex-5-en-3-yn-1-ol **1a** (28.8 mg, 300 μmol, 1.0 equiv.), 4-methoxy-*N*-methyl-aniline **2a** (53.5 mg, 390 μmol, 1.30 equiv.) and **3f** (95.2 mg, 390 μmol, 1.30 equiv.). The crude material was purified by column chromatography (20 – 50 % (v/v) hexane in EtOAc) to give **4aaf** (66.4 mg, 203 μmol, 68% yield) as a yellow oil.

R_f = 0.33 (50% EtOAc in pentane).

^{1}H NMR (400 MHz, CDCl$_3$) δ 7.25 – 7.18 (m, 2H, Ar*H*), 7.00 – 6.89 (m, 2H, Ar*H*), 6.79 (s, 4H, Ar*H*), 5.58 (tt, J = 8.6, 3.1 Hz, 1H, C=C*H*), 3.95 (d, J = 5.7 Hz, 2H, NCH_2), 3.80 – 3.65 (m, 5H, OCH_3, HOCH_2), 2.86 (s, 3H, NCH_3), 2.63 – 2.54 (m, 2H, HOCH$_2$CH_2). O*H* was not resolved.

^{13}C{^{1}H} NMR (101 MHz, CDCl$_3$) δ 204.2 (d, $J_{C\text{-}F}$ = 1.9 Hz), 161.9 (d, $J_{C\text{-}F}$ = 246.4 Hz), 152.8, 143.9, 132.0 (d, $J_{C\text{-}F}$ = 3.3 Hz), 127.6 (d, $J_{C\text{-}F}$ = 8.0 Hz), 116.6, 115.3 (d, $J_{C\text{-}F}$ = 21.6 Hz), 114.7, 103.3, 91.5, 60.8, 55.8, 53.5, 40.2, 33.5.

^{19}F NMR (376 MHz, CDCl$_3$) δ -115.7 (ddd, J = 14.0, 8.8, 5.3 Hz).

IR (cm^{-1}) 3397 (w), 2940 (w), 2834 (w), 1947 (w), 1600 (w), 1509 (s), 1237 (s), 1036 (s), 836 (m), 816 (m).

HRMS (APCI/QTOF) m/z: [M + H]$^+$ Calcd for $C_{20}H_{23}FNO_2^+$ 328.1707; Found 328.1705.

3-(4-Methoxyphenyl)-6-((4-methoxyphenyl)(methyl)amino)hexa-3,4-dien-1-ol (4aag)

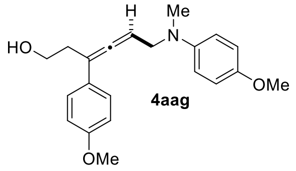

4aag

Prepared according to the general procedure D1 using **1a** (28.8 mg, 300 μmol, 1.0 equiv.), 4-methoxy-*N*-methyl-aniline **2a** (53.5 mg, 390 μmol, 1.30 equiv.) and (4-methoxyphenyl) trifluoromethanesulfonate **3g** (99.9 mg, 390 μmol, 1.30 equiv.). The crude material was purified by column chromatography (20 – 50 % (v/v) EtOAc in hexane) to give **4aag** (76.8 mg, 226 μmol, 75% yield) as a yellow oil.

R_f(50% EtOAc/hexane) = 0.32.

^{1}H NMR (400 MHz, CDCl$_3$) δ 7.26 – 7.17 (m, 2H, Ar*H*), 6.85 – 6.80 (m, 2H, Ar*H*), 6.79 (s, 4H, Ar*H*), 5.57 (tt, J = 5.7, 2.9 Hz, 1H, C=C*H*), 3.94 (d, J = 5.8 Hz, 2H, NCH_2), 3.80 (s, 3H, OCH_3), 3.75 (s, 3H, OCH_3), 3.74 (q, J = 6.4 Hz, 2H, HOCH_2), 2.87 (s, 3H, NCH_3), 2.66 – 2.54 (m, 2H, HOCH$_2$CH_2). O*H* was not resolved.

^{13}C{^{1}H} NMR (101 MHz, CDCl$_3$) δ 203.9, 158.8, 152.8, 144.2, 128.2, 127.3, 116.6, 114.8, 114.0, 103.6, 91.4, 61.0, 55.8, 55.4, 53.8, 40.1, 33.6.

IR (cm^{-1}) 3372 (m), 2945 (w), 2836 (w), 1608 (m), 1510 (s), 1245 (s).

HRMS (ESI/QTOF) m/z: [M + H]$^+$ Calcd for $C_{21}H_{26}NO_3^+$ 340.1907; Found 340.1898.

6-((4-Methoxyphenyl)(methyl)amino)-3-(4-(trifluoromethyl)phenyl)hexa-3,4-dien-1-ol (4aah)

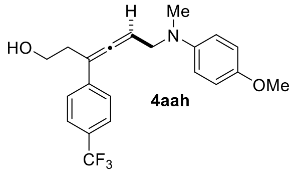

4aah

Prepared according to the general procedure D1 using **1a** (28.8 mg, 300 μmol, 1.0 equiv.), 4-methoxy-*N*-methyl-aniline **2a** (53.5 mg, 390 μmol, 1.30 equiv.) and and 4-(trifluoromethyl)phenyl trifluoromethanesulfonate **3h** (115 mg, 390 μmol, 1.30 equiv.). The crude material was purified by column chromatography (20 – 50 % (v/v) EtOAc in hexane) to give **4aah** (97.7 mg, 259 μmol, 86% yield) as a yellow oil.

R_f(50% EtOAc/hexane) = 0.29.

^{1}H NMR (400 MHz, CDCl$_3$) δ 7.43 (d, J = 8.3 Hz, 2H, Ar*H*), 7.26 (d, J = 8.3 Hz, 2H, Ar*H*), 6.70 (s, 4H, Ar*H*), 5.58 (dt, J = 5.4, 2.7 Hz, 1H, C=C*H*), 3.90 (d, J = 5.6 Hz, 2H, CH_2N), 3.74 – 3.66 (m, 2H, OHCH_2CH$_2$), 3.65 (s, 3H, CH_3), 2.79 (s, 3H, CH_3), 2.55 (tt, J = 6.1, 2.7 Hz, 2H, OHCH$_2$CH_2). O*H* was not resolved.

$^{13}C\{^1H\}$ NMR (101 MHz, $CDCl_3$) δ 205.1, 153.0, 143.8, 140.0, 128.8 (q, J = 32.4 Hz), 126.3, 125.3 (q, J = 3.8 Hz), 124.4 (q, J = 271.6 Hz), 116.8, 114.8, 103.5, 92.0, 60.8, 55.7, 53.3, 40.5, 33.2.

^{19}F NMR (376 MHz, $CDCl_3$) δ -62.4.

IR (cm^{-1}) 3382 (m), 2940 (w), 2838 (w), 1944 (w), 1616 (w), 1512 (s), 1324 (s).

HRMS (nanochip-ESI/LTQ-Orbitrap) m/z: $[M + H]^+$ Calcd for $C_{21}H_{23}F_3NO_2^+$ 378.1675; Found 378.1685.

4-(1-Hydroxy-6-((4-methoxyphenyl)(methyl)amino)hexa-3,4-dien-3-yl)benzaldehyde (4aai)

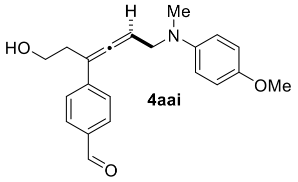

Prepared according to the general procedure D1 using hex-5-en-3-yn-1-ol **1a** (28.8 mg, 300 µmol, 1.0 equiv.) 4-methoxy-*N*-methyl-aniline **2a** (53.5 mg, 390 µmol, 1.30 equiv.) and **3i** (99.1 mg, 390 µmol, 1.30 equiv.). The crude material was purified by column chromatography (15 – 50 % (v/v) hexanes in EtOAc) to give **4aai** (17.6 mg, 52.2 µmol, 17% yield) as a yellow oil.

R_f = 0.28 (50% EtOAc in pentane).

^1H NMR (400 MHz, $CDCl_3$) δ 9.96 (s, 1H, C*H*O), 7.76 (d, J = 8.3 Hz, 2H, Ar*H*), 7.41 (d, J = 8.3 Hz, 2H, Ar*H*), 6.78 (s, 4H, Ar*H*), 5.68 (tt, J = 5.5, 2.8 Hz, 1H, C=C*H*), 3.99 (d, J = 5.6 Hz, 2H, NCH_2), 3.81 – 3.74 (m, 2H, $HOCH_2$), 3.72 (s, 3H, OCH_3), 2.87 (s, 3H, NCH_3), 2.67 – 2.59 (m, 2H, $HOCH_2CH_2$). O*H* was not resolved.

$^{13}C\{^1H\}$ NMR (101 MHz, $CDCl_3$) δ 205.8, 191.8, 153.1, 143.7, 142.8, 134.9, 129.9, 126.5, 116.8, 114.8, 104.0, 92.0, 60.8, 55.8, 53.3, 40.5, 33.1.

IR (cm^{-1}) 3464 (w), 2950 (w), 2835 (w), 1944 (w), 1697 (m), 1601 (m), 1512 (m), 1245 (m), 1173 (w), 1037 (m), 907 (s), 831 (m).

HRMS (ESI/QTOF) m/z: $[M + H]^+$ Calcd for $C_{21}H_{24}NO_3^+$ 338.1751; Found 338.1757.

Methyl 4-(1-hydroxy-6-((4-methoxyphenyl)(methyl)amino)hexa-3,4-dien-3-yl)benzoate (4aaj)

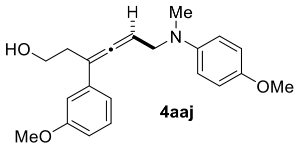

Prepared according to the general procedure D1 using hex-5-en-3-yn-1-ol **1a** (28.8 mg, 300 µmol, 1.0 equiv.), 4-methoxy-*N*-methyl-aniline **2a** (53.5 mg, 390 µmol, 1.30 equiv.) and **3j** (99.9 mg, 390 µmol, 1.30 equiv.). The crude material was purified by column chromatography (20 – 50 % (v/v) hexane in EtOAc) to give **4aaj** (74.2 mg, 219 µmol, 73% yield) as a yellow oil.

R_f = 0.32 (50% EtOAc in pentane).

^1H NMR (400 MHz, $CDCl_3$) δ 7.21 (t, J = 7.9 Hz, 1H, Ar*H*), 6.95 – 6.86 (m, 2H, Ar*H*), 6.84 – 6.72 (m, 5H, Ar*H*), 5.59 (tt, J = 5.7, 2.9 Hz, 1H, C=C*H*), 3.96 (d, J = 5.8 Hz, 2H, NCH_2), 3.77 (s, 3H, OCH_3), 3.77 – 3.70 (m, 5H, OCH_3, $HOCH_2$), 2.87 (s, 3H, NCH_3), 2.66 – 2.57 (m, 2H, $HOCH_2CH_2$). O*H* was not resolved.

$^{13}C\{^1H\}$ NMR (101 MHz, $CDCl_3$) δ 204.4, 159.8, 152.7, 144.0, 137.6, 129.4, 118.6, 116.4, 114.7, 112.3, 112.0, 103.9, 91.3, 60.9, 55.8, 55.3, 53.5, 39.9, 33.4.

IR (cm^{-1}) 3397 (m), 2993 (w), 2940 (m), 2834 (m), 1944 (w), 1600 (m), 1512 (s), 1454 (m), 1288 (m), 1244 (s), 1038 (s), 817 (m).

HRMS (ESI/QTOF) m/z: $[M + H]^+$ Calcd for $C_{21}H_{26}NO_3^+$ 340.1907; Found 340.1924.

6-((4-Methoxyphenyl)(methyl)amino)-3-(naphthalen-2-yl)hexa-3,4-dien-1-ol (4aak)

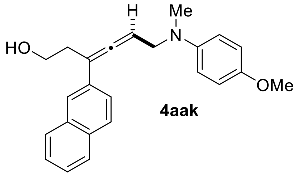

Prepared according to the general procedure D1 using hex-5-en-3-yn-1-ol **1a** (28.8 mg, 300 µmol, 1.0 equiv.), 4-methoxy-*N*-methyl-aniline **2a** (53.5 mg, 390 µmol, 1.30 equiv.) and **4k** (108 mg, 390 µmol, 1.30 equiv.). The crude material was purified by column chromatography (15 – 50 % (v/v) hexanes in EtOAc) to give **4aak** (74.0 mg, 206 µmol, 69% yield) as an orange oil.

R_f = 0.50 (50% EtOAc in pentane).

^1H NMR (400 MHz, $CDCl_3$) δ 7.84 – 7.75 (m, 2H, Ar*H*), 7.75 – 7.65 (m, 2H, Ar*H*), 7.55 – 7.36 (m, 3H, Ar*H*), 6.87 – 6.71 (m, 4H, Ar*H*), 5.68 (tt, J = 5.7, 2.8 Hz, 1H, C=C*H*), 4.01 (dd, J = 5.7, 1.1 Hz, 2H, NCH_2), 3.86 – 3.78 (m, 2H, $HOCH_2$), 3.70 (s, 3H, OCH_3), 2.90 (s, 3H, NCH_3), 2.79 – 2.72 (m, 2H, $HOCH_2CH_2$). O*H* was not resolved.

$^{13}C\{^1H\}\{^1H\}$ NMR (101 MHz, $CDCl_3$) δ 205.0, 152.8, 144.0, 133.6, 133.4, 132.6, 128.1, 127.9, 127.6, 126.3, 125.9, 125.2, 123.9, 116.6, 114.8, 104.3, 91.7, 61.0, 55.7, 53.6, 40.2, 33.4.

IR (cm^{-1}) 3378 (w), 3054 (w), 2950 (m), 2900 (m), 2834 (w), 1941 (w), 1512 (s), 1246 (m), 1039 (m), 913 (s), 819 (m).

HRMS (APCI/QTOF) m/z: $[M + H]^+$ Calcd for $C_{24}H_{26}NO_2^+$ 360.1958; Found 360.1946.

6-((4-Methoxyphenyl)(methyl)amino)-3-(o-tolyl)hexa-3,4-dien-1-ol (4aal)

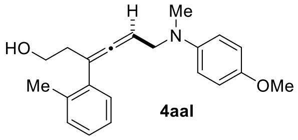

4aal

Prepared according to the general procedure D1 using hex-5-en-3-yn-1-ol **1a** (28.8 mg, 300 µmol, 1.0 equiv.), 4-methoxy-*N*-methyl-aniline **2a** (53.5 mg, 390 µmol, 1.30 equiv.) and **3l** (93.7 mg, 390 µmol, 1.30 equiv.). The crude material was purified by column chromatography (15– 45 % (v/v) hexane in EtOAc) to give **4aal** (31.1 mg, 96.2 µmol, 32% yield) as a yellow oil.

R_f = 0.60 (50% EtOAc in pentane).

^{1}H NMR (400 MHz, $CDCl_3$) δ 7.20 – 7.10 (m, 3H, Ar*H*), 7.09 – 7.02 (m, 1H, Ar*H*), 6.83 – 6.70 (m, 4H, Ar*H*), 5.31 (tt, *J* = 5.8, 2.8 Hz, 1H, C=C*H*), 3.88 (d, *J* = 5.8 Hz, 2H, NC*H*$_2$), 3.75 (s, 3H, OC*H*$_3$), 3.72 – 3.63 (m, 2H, HOC*H*$_2$), 2.85 (s, 3H, NC*H*$_3$), 2.54 – 2.45 (m, 2H, HOCH$_2$C*H*$_2$), 2.31 (s, 3H, ArC*H*$_3$). O*H* was not resolved.

^{13}C{^{1}H} NMR (101 MHz, $CDCl_3$) δ 203.1, 152.6, 144.1, 137.1, 135.8, 130.6, 128.3, 127.2, 125.9, 116.2, 114.7, 103.2, 88.4, 60.7, 55.8, 53.5, 39.8, 37.2, 20.4.

IR (cm^{-1}) 3427 (w), 2954 (m), 1955 (w), 1513 (s), 1457 (m), 1246 (s), 1040 (s), 914 (w), 817 (m).

HRMS (APCI/QTOF) m/z: $[M + H]^+$ Calcd for $C_{21}H_{26}NO_2^+$ 324.1958; Found 324.1950.

3-(2-Fluorophenyl)-6-((4-methoxyphenyl)(methyl)amino)hexa-3,4-dien-1-ol (4aam)

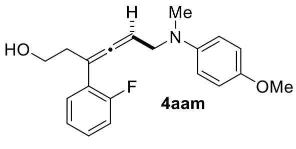

4aam

Prepared according to the general procedure D1 using hex-5-en-3-yn-1-ol **1a** (28.8 mg, 300 µmol, 1.00 equiv.) 4-methoxy-*N*-methylaniline **2a** (53.5 mg, 390 µmol, 1.30 equiv.) and **3m** (95.2 mg, 390 µmol, 1.30 equiv.). The crude material was purified by column chromatography (15 – 50 % (v/v) hexanes in EtOAc) to give **4aam** (39.7 mg, 121 µmol, 40% yield) as a yellow oil.

R_f = 0.63 (30% EtOAc in pentane).

^{1}H NMR (400 MHz, $CDCl_3$) δ 7.23 – 7.10 (m, 2H, Ar*H*), 7.07 – 6.97 (m, 2H, Ar*H*), 6.78 (s, 4H, Ar*H*), 5.44 (tt, *J* = 5.7, 2.8 Hz, 1H, C=CH), 4.00 – 3.86 (m, 2H, NC*H*$_2$), 3.74 (s, 3H, ArOC*H*$_3$), 3.73 – 3.66 (m, 2H, HOC*H*$_2$), 2.87 (s, 3H, NC*H*$_3$), 2.65 – 2.54 (m, 2H, HOCH$_2$C*H*$_2$). O*H* was not resolved.

^{13}C{^{1}H} NMR (101 MHz, $CDCl_3$) δ 205.4 (d, J_{C-F} = 1.4 Hz), 160.1 (d, J_{C-F} = 248.7 Hz), 152.6, 144.1, 129.6 (d, J_{C-F} = 3.5 Hz), 128.7 (d, J_{C-F} = 8.4 Hz), 124.9 (d, J_{C-F} = 12.4 Hz), 124.1 (d, J_{C-F} = 3.7 Hz), 116.3, 116.1 (d, J_{C-F} = 22.8 Hz), 114.7, 99.5, 89.3, 60.8, 55.8, 53.3, 39.9, 35.5 (d, J_{C-F} = 2.5 Hz).

^{19}F NMR (376 MHz, $CDCl_3$) δ -113.3 – -113.5 (m).

IR (cm^{-1}) 3383 (m), 2970 (m), 2357 (w), 1951 (w), 1643 (w), 1514 (s), 1490 (m), 1453 (m), 1247 (s), 1038 (s), 820 (m).

HRMS (nanochip-ESI/LTQ-Orbitrap) m/z: $[M + H]^+$ Calcd for $C_{20}H_{23}FNO_2^+$ 328.1707; Found 328.1708.

Methyl 2-fluoro-4-(1-hydroxy-6-((4-methoxyphenyl)(methyl)amino)hexa-3,4-dien-3-yl)benzoate (4aan)

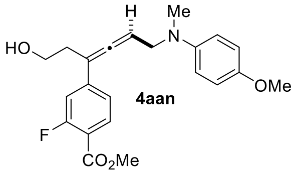

4aan

Prepared according to the general procedure D1 using hex-5-en-3-yn-1-ol **1a** (28.8 mg, 300 µmol, 1.0 equiv.), 4-methoxy-*N*-methyl-aniline **2a** (53.5 mg, 390 µmol, 1.30 equiv.) and **3n** (118 mg, 390 µmol, 1.30 equiv.). The crude material was purified by column chromatography (15 – 50 % (v/v) hexanes in EtOAc) to give **4aan** (75.4 mg, 196 µmol, 65% yield) as an orange oil.

R_f = 0.31 (50% EtOAc in pentane).

^{1}H NMR (400 MHz, $CDCl_3$) δ 7.81 (t, *J* = 8.0 Hz, 1H, Ar*H*), 7.06 (dd, *J* = 8.3, 1.8 Hz, 1H, Ar*H*), 7.00 (dd, *J* = 12.5, 1.8 Hz, 1H, Ar*H*), 6.78 (s, 4H, Ar*H*), 5.66 (tt, *J* = 5.6, 2.9 Hz, 1H, C=C*H*), 3.98 (d, *J* = 5.5 Hz, 2H, NC*H*$_2$), 3.91 (s, 3H, OC*H*$_3$), 3.79 – 3.67 (m, 5H, HOC*H*$_2$, CO$_2$C*H*$_3$), 2.86 (s, 3H, NC*H*$_3$), 2.61 – 2.53 (m, 2H, HOCH$_2$C*H*$_2$). O*H* was not resolved.

^{13}C{^{1}H} NMR (101 MHz, $CDCl_3$) δ 205.5, 164.9 (d, J_{C-F} = 4.1 Hz), 162.1 (d, J_{C-F} = 259.1 Hz), 153.1, 143.6 (d, J_{C-F} = 8.6 Hz), 132.1, 132.1, 121.4 (d, J_{C-F} = 3.4 Hz), 116.8, 116.5 (d, J_{C-F} = 10.3 Hz), 114.8, 114.3 (d, J_{C-F} = 24.1 Hz), 103.3 (d, J_{C-F} = 2.2 Hz), 92.3, 60.6, 55.7, 53.1, 52.4, 40.6, 33.0.

^{19}F NMR (376 MHz, $CDCl_3$) δ -109.5 – -109.6 (m).

IR (cm^{-1}) 3424 (w), 2991 (w), 2953 (m), 2835 (w), 1941 (w), 1718 (s), 1617 (s), 1512 (s), 1437 (m), 1292 (s), 1245 (s), 1094 (m), 1037 (s), 816 (m).

HRMS (ESI/QTOF) m/z: $[M + H]^+$ Calcd for $C_{22}H_{25}FNO_4^+$ 386.1762; Found 386.1761.

3-(4-Allyl-2-methoxyphenyl)-6-((4-methoxyphenyl)(methyl)amino)hexa-3,4-dien-1-ol (4aao)

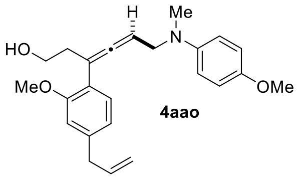

Prepared according to the general procedure D1 using hex-5-en-3-yn-1-ol **1a** (28.8 mg, 300 µmol, 1.0 equiv.), 4-methoxy-*N*-methyl-aniline **2a** (53.5 mg, 390 µmol, 1.30 equiv.) and **3o** (115 mg, 390 µmol, 1.30 equiv.). The crude material was purified by column chromatography (15 – 50 % (v/v) hexanes in EtOAc) to give **4aao** (48.9 mg, 129 µmol, 43% yield) as a brown oil.

R_f = 0.64 (40% EtOAc in pentane).

^{1}H NMR (400 MHz, $CDCl_3$) δ 6.96 (d, *J* = 7.6 Hz, 1H, Ar*H*), 6.82 – 6.75 (m, 4H, Ar*H*), 6.75 – 6.67 (m, 2H, Ar*H*), 6.05 – 5.87 (m, 1H, C=C-*H*), 5.30 (tt, *J* = 8.4, 2.7 Hz, 1H, C=C*H*), 5.13 (br s, 1H, O*H*), 5.12 – 5.04 (m, 2H, C=CH_2), 3.91 (dd, *J* = 5.9, 2.4 Hz, 2H, NCH_2), 3.79 (s, 3H, OCH_3), 3.75 (s, 3H, OCH_3), 3.69 – 3.58 (m, 2H, HOCH_2), 3.37 (d, *J* = 6.8 Hz, 2H, C=C-CH_2), 2.88 (s, 3H, NCH_3), 2.62 – 2.51 (m, 2H, HOCH_2CH_2). O*H* was not resolved.

^{13}C{^{1}H} NMR (101 MHz, $CDCl_3$) δ 204.7, 156.7, 152.4, 144.2, 140.9, 137.2, 129.9, 124.2, 120.9, 116.2, 116.0, 114.7, 111.5, 101.3, 87.6, 60.9, 55.8, 55.6, 53.6, 40.3, 39.5, 36.5.

IR (cm^{-1}) 3418 (w), 2949 (m), 2906 (m), 1950 (w), 1512 (s), 1247 (s), 1039 (s), 914 (s), 818 (m).

HRMS (APCI/QTOF) m/z: [M + H]$^+$ Calcd for $C_{24}H_{30}NO_3^+$ 380.2220; Found 380.2209.

3-(Benzo[*d*][1,3]dioxol-5-yl)-6-((4-methoxyphenyl)(methyl)amino)hexa-3,4-dien-1-ol (4aap)

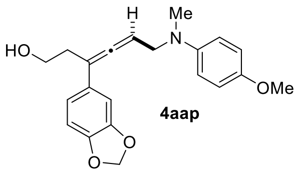

Prepared according to the general procedure D1 using hex-5-en-3-yn-1-ol **1a** (28.8 mg, 300 µmol, 1.0 equiv.), 4-methoxy-*N*-methyl-aniline **2a** (53.5 mg, 390 µmol, 1.30 equiv.) and **3p** (105 mg, 390 µmol, 1.30 equiv.). The crude material was purified by column chromatography (15 – 50 % (v/v) hexanes in EtOAc) to give **4aap** (69.7 mg, 197 µmol, 66% yield) as an orange oil.

R_f = 0.43 (40% EtOAc in pentane).

^{1}H NMR (400 MHz, $CDCl_3$) δ 6.84 – 6.69 (m, 7H, Ar*H*), 5.93 (s, 2H, OCH_2O), 5.56 (tt, *J* = 5.7, 2.9 Hz, 1H, C=C*H*), 3.96 – 3.91 (m, 2H, NCH_2), 3.73 (d, *J* = 11.8 Hz, 5H, OCH_3, HOCH_2), 2.86 (s, 3H, NCH_3), 2.61 – 2.48 (m, 2H, HOCH_2CH_2). O*H* was not resolved.

^{13}C{^{1}H} NMR (101 MHz, $CDCl_3$) δ 204.0, 152.8, 147.9, 146.8, 144.0, 130.1, 119.1, 116.6, 114.7, 108.1, 106.9, 103.9, 101.1, 91.4, 60.8, 55.7, 53.5, 40.2, 33.6.

IR (cm^{-1}) 3392 (w), 2955 (m), 2890 (m), 1941 (w), 1613 (w), 1510 (s), 1486 (s), 1106 (m), 1036 (s), 914 (s), 813 (m).

HRMS (APCI/QTOF) m/z: [M + H]$^+$ Calcd for $C_{21}H_{24}NO_4^+$ 354.1700; Found 354.1687.

***tert*-Butyl 5-(1-hydroxy-6-((4-methoxyphenyl)(methyl)amino)hexa-3,4-dien-3-yl)-1H-indole-1-carboxylate (4aaq)**

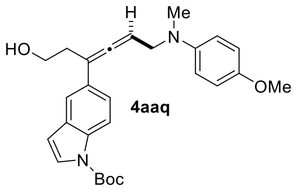

Prepared according to the general procedure D1 using hex-5-en-3-yn-1-ol **1a** (28.8 mg, 300 µmol, 1.0 equiv.), 4-methoxy-*N*-methyl-aniline **2a** (53.5 mg, 390 µmol, 1.30 equiv.) and **3q** (143 mg, 390 µmol, 1.30 equiv.). The crude material was purified by column chromatography (15 – 50 % (v/v) hexanes in EtOAc) to give **4aaq** (92.8 mg, 177 µmol, 59% yield) as an orange oil.

R_f = 0.45 (50% EtOAc in pentane).

^{1}H NMR (400 MHz, $CDCl_3$) δ 8.03 (d, *J* = 8.4 Hz, 1H, Ar*H*), 7.57 (d, *J* = 3.5 Hz, 1H, Ar*H*), 7.43 (d, *J* = 1.5 Hz, 1H, Ar*H*), 7.29 (dd, *J* = 8.7, 1.7 Hz, 1H, Ar*H*), 6.80 (s, 4H, Ar*H*), 6.51 (d, *J* = 3.6 Hz, 1H Ar*H*), 5.61 (tt, *J* = 5.5, 2.7 Hz, 1H, C=C*H*), 4.04 – 3.91 (m, 2H, NCH_2), 3.84 – 3.75 (m, 2H, HOCH_2), 3.72 (s, 3H, OCH_3), 2.88 (s, 3H, NCH_3), 2.74 – 2.66 (m, 2H, HOCH_2CH_2), 1.68 (s, 9H, C(CH_3)$_3$). O*H* was not resolved.

^{13}C{^{1}H} NMR (101 MHz, $CDCl_3$) δ 204.3, 152.7, 149.8, 144.1, 134.3, 130.9, 130.5, 126.4, 123.0, 118.2, 116.5, 115.1, 114.8, 107.5, 104.3, 91.1, 83.8, 61.0, 55.7, 53.6, 40.1, 33.8, 28.3.

IR (cm^{-1}) 3383 (w), 2976 (m), 2901 (m), 1943 (w), 1733 (m), 1513 (m), 1369 (m), 1247 (m), 1043 (m), 913 (s), 819 (w).

HRMS (nanochip-ESI/LTQ-Orbitrap) m/z: [M + H]$^+$ Calcd for $C_{27}H_{33}N_2O_4^+$ 449.2435; Found 449.2444.

6-((4-Methoxyphenyl)(methyl)amino)-3-(1,7,7-trimethylbicyclo[2.2.1]hept-2-en-2-yl)hexa-3,4-dien-1-ol (4aar)

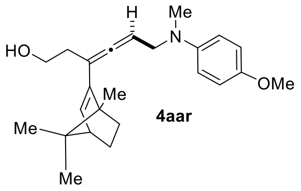

Prepared according to the general procedure D1 using hex-5-en-3-yn-1-ol **1a** (28.8 mg, 300 µmol, 1.0 equiv.), 4-methoxy-*N*-methyl-aniline **2a** (53.5 mg, 390 µmol, 1.30 equiv.) and **3r** (111 mg, 390 µmol, 1.30 equiv.). The crude material was purified by column chromatography (15 – 50 % (v/v) hexanes in EtOAc) to give **4aar** (47.9 mg, 130 µmol, 43% yield) as an orange oil, as a mixture of diastereomers in a 1:1 ratio that is not resolved in ^{1}H NMR, but can be resolved in ^{13}C NMR.

R_f = 0.54 (50% EtOAc in pentane).

^{1}H NMR (400 MHz, $CDCl_3$) δ 6.89 – 6.71 (m, 4H, Ar*H*), 5.88 (d, *J* = 3.0 Hz, 1H, C=C*H*), 5.41 – 5.30 (m, 1H, C=C=C*H*), 3.90 – 3.82 (m, 2H, NC*H*$_2$), 3.78 – 3.73 (m, 3H, OC*H*$_3$), 3.70 – 3.62 (m, 2H, HOC*H*$_2$), 2.90 – 2.84 (m, 3H, NC*H*$_3$), 2.39 – 2.33 (m, 2H, HOCH$_2$C*H*$_2$), 2.30 (dd, *J* = 3.4, 3.4 Hz, 1H, C*H*), 1.91 – 1.78 (m, 1H, C*H*$_2$), 1.54 – 1.42 (m, 1H, C*H*$_2$), 1.11 – 1.06 (m, 3H, C*H*$_3$), 1.05 – 0.92 (m, 2H, C*H*$_2$), 0.80 – 0.71 (m, 6H, C*H*$_3$). O*H* was not resolved.

$^{13}C\{^1H\}$ NMR (101 MHz, $CDCl_3$) δ 204.5 (major), 204.1 (minor), 152.7 (major), 152.6 (minor), 144.4 (major), 144.3 (minor), 143.7 (major), 143.7 (minor), 131.6 (major), 131.5 (minor), 116.4 (major), 116.3 (minor), 114.8 (major), 114.8 (minor), 99.4 (major), 99.2 (minor), 90.0 (major), 89.6 (minor), 61.1 (major), 61.0 (minor), 56.9 (major), 56.6 (minor), 55.9 (major), 55.8 (minor), 55.4 (major), 55.1 (minor), 54.3 (major), 54.2 (minor), 51.5 (major), 51.3 (minor), 39.8 (major), 39.8 (minor), 35.0 (major), 34.9 (minor), 31.7 (major), 31.6 (minor), 26.1 (major), 26.1 (minor), 19.9 (major), 19.8 (minor), 19.7 (major & minor), 12.9 (major), 12.8 (minor).

IR (cm^{-1}) 3387 (m), 2949 (m), 2872 (m), 2832 (w), 1936 (w), 1512 (s), 1455 (m), 1244 (s), 1038 (s), 914 (m), 819 (m).

HRMS (ESI/QTOF) m/z: [M + H]$^+$ Calcd for $C_{24}H_{34}NO_2^+$ 368.2584; Found 368.2581.

Methyl (2*S*)-2-((tert-butoxycarbonyl)amino)-3-(4-(1-hydroxy-6-((4-methoxyphenyl)(methyl)amino)hexa-3,4-dien-3-yl)phenyl)propanoate (4aas)

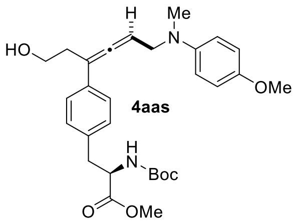

Prepared according to the general procedure D1 using hex-5-en-3-yn-1-ol **1a** (28.8 mg, 300 μmol, 1.0 equiv.), 4-methoxy-*N*-methyl-aniline **2a** (53.5 mg, 390 μmol, 1.30 equiv.) and **3s** (167 mg, 390 μmol, 1.30 equiv.). The crude material was purified by column chromatography (15 – 50 % (v/v) hexanes in EtOAc) to give **4aas** (97.9 mg, 192 μmol, 64% yield) as an orange oil.

R_f = 0.30 (50% EtOAc in pentane).

^{1}H NMR (400 MHz, $CDCl_3$) δ 7.24 – 7.19 (m, 2H, Ar*H*), 7.04 (d, *J* = 8.0 Hz, 2H, Ar*H*), 6.83 – 6.75 (m, 4H, Ar*H*), 5.58 (tt, *J* = 5.8, 2.9 Hz, 1H, C=C*H*), 5.02 (d, *J* = 8.3 Hz, 1H, N*H*), 4.63 – 4.46 (m, 1H, COC*H*NH), 3.94 (d, *J* = 5.8 Hz, 2H, NC*H*$_2$), 3.77 – 3.68 (m, 8H, CO$_2$C*H*$_3$, OC*H*$_3$, HOC*H*$_2$), 3.05 (t, *J* = 6.0 Hz, 2H, ArC*H*$_2$), 2.86 (s, 3H, NC*H*$_3$), 2.63 – 2.56 (m, 2H, HOCH$_2$C*H*$_2$), 1.42 (s, 9H, C(C*H*$_3$)$_3$). O*H* was not resolved.

$^{13}C\{^1H\}$ NMR (101 MHz, $CDCl_3$) δ 204.3, 172.4, 155.2, 152.8, 144.0, 134.8, 129.4, 126.2, 116.5, 116.5, 114.8, 103.7, 91.4, 80.1, 60.9, 55.8, 54.5, 53.6, 52.3, 39.9, 38.0, 33.3, 28.4.

IR (cm^{-1}) 3389 (w), 2976 (m), 2935 (m), 2248 (w), 1943 (w), 1742 (m), 1711 (m), 1511 (s), 1365 (m), 1246 (s), 1166 (s), 1039 (s), 913 (s), 818 (m).

HRMS (nanochip-ESI/LTQ-Orbitrap) m/z: [M + H]$^+$ Calcd for $C_{29}H_{39}N_2O_6^+$ 511.2803; Found 511.2824.

(8*R*,9*S*,13*S*,14*S*)-3-(1-Hydroxy-6-((4-methoxyphenyl)(methyl)amino)hexa-3,4-dien-3-yl)-13-methyl-6,7,8,9,11,12,13,14,15,16-decahydro-17*H*-cyclopenta[a]phenanthren-17-one (4aat)

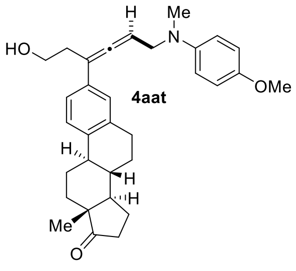

Prepared according to the general procedure D1 using hex-5-en-3-yn-1-ol **1a** (28.8 mg, 300 μmol, 1.0 equiv.), 4-methoxy-*N*-methyl-aniline **2a** (53.5 mg, 390 μmol, 1.30 equiv.) and **3t** (157 mg, 390 μmol, 1.30 equiv.). The crude material was purified by column chromatography (15 – 50 % (v/v) hexanes in EtOAc) to give **4aat** (121 mg, 249 μmol, 83% yield) as an orange oil.

R_f = 0.27 (50% EtOAc in pentane)

^{1}H NMR (400 MHz, $CDCl_3$) δ 7.22 (d, *J* = 8.6 Hz, 1H, Ar*H*), 7.10 (dd, *J* = 8.2, 2.1 Hz, 1H, Ar*H*), 7.03 (d, *J* = 2.1 Hz, 1H, Ar*H*), 6.80 (s, 4H, Ar*H*), 5.57 (tt, *J* = 5.8, 2.8 Hz, 1H, C=C*H*), 3.95 (d, *J* = 5.8 Hz, 2H, NC*H*$_2$), 3.81 – 3.68 (m, 5H, OC*H*$_3$, HOC*H*$_2$), 2.92 – 2.81 (m, 5H, NC*H*$_3$, ArC*H*$_2$), 2.65 – 2.57 (m, 2H, HOCH$_2$C*H*$_2$), 2.55 – 2.47 (m, 1H, C*H*$_2$), 2.45 – 2.37 (m, 1H, C*H*$_2$), 2.33 – 2.23 (m, 1H, C*H*$_2$), 2.20 – 2.09 (m, 1H, C*H*), 2.07 – 1.94 (m, 3H, C*H*$_2$), 1.69 – 1.57 (m, 2H, C*H*$_2$), 1.57 – 1.40 (m, 4H, C*H* and C*H*$_2$), 0.91 (s, 3H. C*H*$_3$). O*H* was not resolved.

$^{13}C\{^1H\}$ NMR (101 MHz, $CDCl_3$) δ 221.0, 204.2, 152.6, 144.0, 138.7, 136.6, 133.4, 126.6, 125.5, 123.6, 116.3, 114.7, 103.6, 91.1, 61.0, 55.8, 53.6, 50.6, 48.1, 44.4, 39.9, 38.2, 35.9, 33.4, 31.7, 29.6, 26.6, 25.8, 21.7, 13.9.

IR (cm^{-1}) 3447 (w), 2931 (m), 2247 (w), 1942 (w), 1734 (m), 1511 (s), 1245 (s), 1039 (m), 912 (s), 818 (m).

HRMS (nanochip-ESI/LTQ-Orbitrap) m/z: [M + H]$^+$ Calcd for $C_{32}H_{40}NO_3^+$ 486.3003; Found 486.3011.

Methyl 4-(6-hydroxy-1-((4-methoxyphenyl)(methyl)amino)hepta-2,3-dien-4-yl)benzoate (4jab)

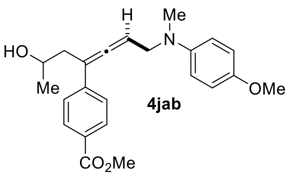

Prepared according to the general procedure D1 using **1j** (33.1 mg, 300 μmol, 1.0 equiv.), 4-methoxy-*N*-methyl-aniline **2a** (53.5 mg, 390 μmol, 1.30 equiv.) and methyl 4-(trifluoromethylsulfonyloxy)benzoate **3b** (111 mg, 390 μmol, 1.30 equiv.). The crude material was purified by column chromatography (20 – 50 % (v/v) EtOAc in hexane) to give **4jab** (96.8 mg, 254 μmol, 85% yield, 1:1 *dr*) as a yellow oil.

R_f(50% EtOAc/hexane) = 0.32.

^{1}H NMR (400 MHz, $CDCl_3$) δ 7.96 – 7.87 (m, 2H, Ar*H*), 7.30 (ddt, *J* = 10.6, 9.0, 2.1 Hz, 2H, Ar*H*), 6.83 – 6.72 (m, 4H, Ar*H*), 5.64 (tt, *J* = 5.4, 2.8 Hz, 1H, C=C*H*), 3.97 (ddd, *J* = 12.6, 5.7, 1.4 Hz, 2H, NCH_2C=C), 3.91 (s, 3H, CO_2CH_3), 3.89 – 3.83 (m, 1H, C*H*CH_3) 3.72 (d, *J* = 2.1 Hz, 3H, OCH_3), 2.85 (d, *J* = 5.6 Hz, 3H, NCH_3), 2.59 – 2.39 (m, 2H, $OHCHCH_2$), 1.25 (t, *J* = 6.0 Hz, 3H, $CHCH_3$). O*H* was not resolved.

Given as a list of peaks due to the heavy overlap of peaks:

$^{13}C\{^1H\}$ NMR (101 MHz, $CDCl_3$) δ 205.7, 167.03, 167.02, 153.1, 152.9, 144.0, 143.8, 141.2, 141.0, 129.7, 128.46, 128.43, 126.1, 126.0, 117.0, 116.6, 114.8, 114.8, 104.4, 104.2, 91.8, 91.3, 66.2, 66.0, 55.7, 53.4, 53.2, 52.2, 40.6, 40.3, 40.2, 39.8, 23.1.

IR (cm^{-1}) 3410 (w), 2952 (w), 2251 (w), 1937 (w), 1713 (m), 1511 (s), 1281 (s).

HRMS (nanochip-ESI/LTQ-Orbitrap) m/z: $[M + H]^+$ Calcd for $C_{23}H_{28}NO_4^+$ 382.2013; Found 382.2017.

Methyl 4-(1-hydroxy-6-((4-methoxyphenyl)(methyl)amino)-5-methylhexa-3,4-dien-3-yl)benzoate (4kab)

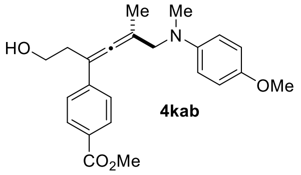

Prepared according to the general procedure D1 using **1k** (33.1 mg, 300 µmol, 1.0 equiv.), 4-methoxy-*N*-methyl-aniline **2a** (53.5 mg, 390 µmol, 1.30 equiv.) and methyl 4-(trifluoromethylsulfonyloxy)benzoate **3b** (111 mg, 390 µmol, 1.30 equiv.). The crude material was purified by column chromatography (20 – 50 % (v/v) EtOAc in hexane) to give **4kab** (22.5 mg, 59.0 µmol, 20% yield) as a yellow oil.

R_f(50% EtOAc/hexane) = 0.41.

^{1}H NMR (400 MHz, $CDCl_3$) δ 7.94 – 7.86 (m, 2H, Ar*H*), 7.30 – 7.23 (m, 2H, Ar*H*), 6.71 (s, 4H, Ar*H*), 3.90 (s, 3H, CO_2CH_3), 3.85 (d, *J* = 6.3 Hz, 2H, NCH_2), 3.69 (tq, *J* = 11.0, 5.5 Hz, 2H, $OHCH_2CH_2$), 3.69 (s, 3H, OCH_3), 2.81 (s, 3H, NCH_3), 2.56 (t, *J* = 6.0 Hz, 2H, $OHCH_2CH_2$), 1.83 (s, 3H, C=CCH_3). O*H* was not resolved.

$^{13}C\{^1H\}$ NMR (101 MHz, $CDCl_3$) δ 202.7, 167.1, 152.8, 144.3, 142.0, 129.7, 128.2, 125.9, 116.3, 114.7, 103.6, 102.0, 60.8, 57.7, 55.7, 52.2, 41.3, 33.5, 16.9.

IR (cm^{-1}) 3420 (w), 2943 (w), 1946 (w), 1717 (s), 1605 (m), 1512 (s).

HRMS (ESI/QTOF) m/z: $[M + H]^+$ Calcd for $C_{23}H_{28}NO_4^+$ 382.2013; Found 382.2016.

***N*-(6-((tert-butyldimethylsilyl)oxy)-4-(*p*-tolyl)hexa-2,3-dien-1-yl)-4-(trifluoromethyl)aniline (S34)**

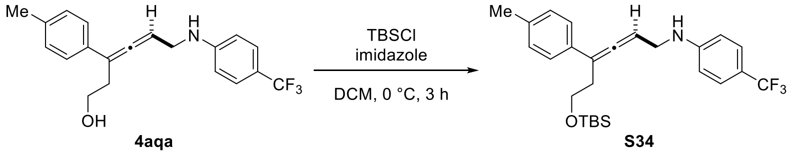

TBSCl (49.7 mg, 0.33 mmol, 1.1 equiv.) and imidazole (61.3 mg, 0.90 mmol, 3.0 equiv.) were added to a solution of **4aqa** (104.2 g, 300.0 µmol, 1.0 equiv.) in DCM (6 mL) at room temperature. The reaction was stirred at room temperature for 3 h. The mixture was diluted with sat. NH_4Cl (10 mL) and DCM (10 mL). The layers were separated, and the aqueous layer was extracted with DCM (2 × 10 mL). The combined organic layers were washed with brine (20 mL), dried with Na_2SO_4, filtered and concentrated under vacuum. The crude was purified by column chromatography on silica gel (10% EtOAc in hexane) to afford **S34** (132 mg, 286 µmol, 95%).

R_f(10% EtOAc/hexane) = 0.69.

^{1}H NMR (400 MHz, $CDCl_3$) δ 7.34 (d, *J* = 8.4 Hz, 2H, Ar*H*), 7.20 (d, *J* = 8.2 Hz, 2H, Ar*H*), 7.09 (d, *J* = 8.0 Hz, 2H, Ar*H*), 6.59 (d, *J* = 8.5 Hz, 2H, Ar*H*), 5.60 (dt, *J* = 5.2, 2.6 Hz, 1H, C=C*H*), 4.35 – 4.25 (m, 1H, N*H*), 3.94 – 3.77 (m, 2H, NCH_2), 3.74 (t, *J* = 7.0 Hz, 2H, OCH_2), 2.60 (td, *J* = 7.0, 2.7 Hz, 2H, OCH_2CH_2), 2.30 (s, 3H, $ArCH_3$), 0.85 (s, 9H, $C(CH_3)_3$), -0.00 (d, *J* = 1.8 Hz, 6H, $SiCH_3$ x2).

$^{13}C\{^1H\}$ NMR (101 MHz, $CDCl_3$) δ 203.5, 150.2, 136.9, 132.9, 129.1, 126.5 (q, J_{C-F} = 3.7 Hz), 126.0, 125.0 (q, J_{C-F} = 270.3 Hz), 119.0 (q, J_{C-F} = 32.6 Hz), 112.3, 105.8, 91.9, 62.0, 42.2, 33.6, 26.0, 21.1, 18.4, -5.23, -5.26.

^{19}F NMR (376 MHz, $CDCl_3$) δ -61.0.

IR (cm^{-1}) 2952 (m), 2930 (m), 2858 (w), 1717 (w), 1616 (m), 1325 (s), 1109 (s).

HRMS (ESI/QTOF) m/z: $[M + H]^+$ Calcd for $C_{26}H_{35}F_3NOSi^+$ 462.2435; Found 462.2441.

D.3. General Procedure D3 for the synthesis of 3-pyrrolines

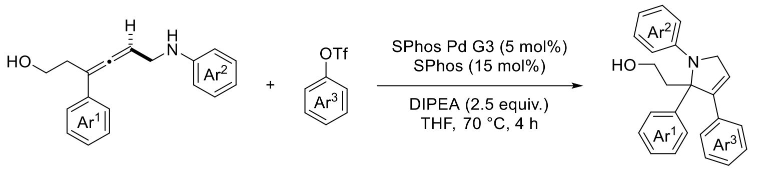

4, 0.3 mmol **3**, (1.3 equiv.) **7**

An oven-dried 8 mL microwave tube equipped with a Teflon coated stirring bar was charged with SPhos Pd G3 (11.7 mg, 15.0 µmol, 5 mol%), SPhos (18.5 mg, 45.0 µmol, 15 mol%) and allene **4** (0.3 mmol). The tube was evacuated and back-filled with N_2 three times. Then, THF (0.6 mL), DIPEA (131 µL, 750 µmol 2.5 equiv.) and aryl triflate **3** (0.39 mmol, 1.3 equiv.) were added and the mixture was stirred at 70 °C for 4 h. Next, the reaction mixture was allowed to cool down to room temperature and concentrated under vacuum. The crude material was purified by flash column chromatography on silica gel using a Biotage flash chromatography machine to afford the corresponding product.

D.4. Characterization of the 3-pyrroline products

Methyl 4-(2-(2-hydroxyethyl)-2-(*p*-tolyl)-1-(4-(trifluoromethyl)phenyl)-2,5-dihydro-1*H*-pyrrol-3-yl)benzoate (7a)

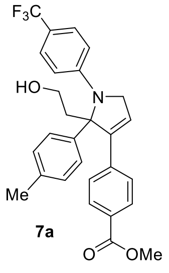

Prepared according to the general procedure D3 using **4aqa** (243.2 mg, 700.0 µmol, 1.00 equiv.) and **3b** (258.6 mg, 910.0 µmol, 1.30 equiv.). The crude material was purified by column chromatography (15 – 50 % (v/v) hexanes in EtOAc) to give **7a** (252.0 mg, 523.4 µmol, 75% yield) as an amorphous yellow solid.

R_f(50% hexane/EtOAc) = 0.57.

^{1}H NMR (400 MHz, $CDCl_3$) δ 7.84 (d, *J* = 8.3 Hz, 2H, Ar*H*), 7.31 (d, *J* = 8.8 Hz, 2H, Ar*H*), 7.24 – 7.11 (m, 4H, Ar*H*), 6.88 (d, *J* = 8.3 Hz, 2H, Ar*H*), 6.53 (d, *J* = 8.8 Hz, 2H, Ar*H*), 6.22 (t, *J* = 2.2 Hz, 1H, C=C*H*), 4.48 (qd, *J* = 15.3, 2.0 Hz, 2H, NCH_2), 3.88 (s, 3H, CO_2CH_3), 3.74 – 3.62 (m, 1H, $HOCH_2$), 3.58 (dq, *J* = 10.5, 5.9 Hz, 1H, $HOCH_2$), 3.00 (ddd, *J* = 14.6, 9.1, 6.0 Hz, 1H, $HOCH_2CH_2$), 2.42 (ddt, *J* = 12.2, 8.9, 4.5 Hz, 1H, $HOCH_2CH_2$), 2.35 (s, 3H, $ArCH_3$), 1.55 (s, 1H, O*H*).

$^{13}C\{^1H\}$ NMR (101 MHz, $CDCl_3$) δ 166.8, 147.1, 146.8, 140.5, 139.1, 137.8, 129.9, 129.5, 128.2, 126.8, 126.5, 126.4 (q, J_{C-F} = 3.9 Hz), 125.2 (q, J_{C-F} = 270.2 Hz), 122.9, 118.0 (q, J_{C-F} = 32.5 Hz), 112.4, 75.1, 59.2, 56.4, 52.3, 34.5, 21.3.

^{19}F NMR (376 MHz, $CDCl_3$) δ -61.0.

IR (cm^{-1}) 3434 (w), 2943 (w), 2824 (w), 1720 (m), 1612 (m), 1529 (w), 1324 (s).

HRMS (ESI/QTOF) m/z: $[M + H]^+$ Calcd for $C_{28}H_{27}F_3NO_3^+$ 482.1938; Found 482.1939.

2-(3-(4-(*tert*-Butyl)phenyl)-2-(*p*-tolyl)-1-(4-(trifluoromethyl)phenyl)-2,5-dihydro-1H-pyrrol-2-yl)ethan-1-ol (7c)

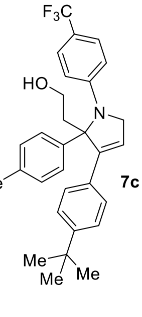

Prepared according to the general procedure D3 using **4aqa** (104.2 mg, 300 µmol, 1.00 equiv.) and **3d** (110 mg, 390 µmol, 1.30 equiv.). The crude material was purified by column chromatography (15 – 50 % (v/v) hexanes in EtOAc) to give **7c** (66.9 mg, 140 µmol, 46% yield) as a yellow oil.

R_f = 0.62 (30% EtOAc in pentane).

^{1}H NMR (400 MHz, $CDCl_3$) δ 7.31 – 7.22 (m, 4H, Ar*H*), 7.21 – 7.11 (m, 4H, Ar*H*), 6.79 – 6.73 (m, 2H, Ar*H*), 6.58 – 6.46 (m, 2H, Ar*H*), 6.13 (t, *J* = 2.1 Hz, 1H, C=C*H*), 4.54 – 4.35 (m, 2H, NCH_2), 3.67 (ddd, *J* = 10.5, 9.2, 5.4 Hz, 1H, $HOCH_2$), 3.56 (ddd, *J* = 10.6, 8.9, 5.8 Hz, 1H, $HOCH_2$), 3.00 (ddd, *J* = 13.6, 9.2, 5.9 Hz, 1H, $HOCH_2CH_2$), 2.49 (ddd, *J* = 14.0, 8.9, 5.4 Hz, 1H, $HOCH_2CH_2$), 2.36 (s, 3H, $ArCH_3$), 1.26 (s, 9H, $C(CH_3)_3$). O*H* was not resolved.

$^{13}C\{^1H\}$ NMR (101 MHz, $CDCl_3$) δ 151.2, 147.6, 147.0, 141.0, 137.5, 131.4, 129.8, 127.7, 126.9, 126.3 (q, J_{C-F} = 3.8 Hz), 125.3 (q, J_{C-F} = 270.5 Hz), 125.3, 123.9, 117.7 (q, J_{C-F} = 32.6 Hz), 112.2, 74.9, 59.4, 56.3, 34.7, 34.4, 31.3, 21.3.

^{19}F NMR (376 MHz, $CDCl_3$) δ -60.9.

IR (cm^{-1}) 3670 (w), 2968 (s), 2901 (s), 2360 (w), 2335 (w), 2249 (w), 1909 (w), 1706 (w), 1614 (m), 1517 (m), 1321 (s), 1226 (m), 1164 (m), 1068 (s), 910 (m), 826 (m).

HRMS (nanochip-ESI/LTQ-Orbitrap) m/z: $[M + H_{-1}]^+$ Calcd for $C_{30}H_{31}F_3NO^+$ 478.2352; Found 478.2363.

2-(3-(3-Methoxyphenyl)-2-(*p*-tolyl)-1-(4-(trifluoromethyl)phenyl)-2,5-dihydro-1H-pyrrol-2-yl)ethan-1-ol (7d)

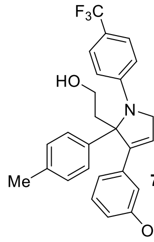

7d

Prepared according to the general procedure D3 using **4aqa** (104.2 mg, 300 µmol, 1.00 equiv.) and **3j** (99.9 mg, 390 µmol, 1.30 equiv.). The crude material was purified by column chromatography (15 – 50 % (v/v) hexanes in EtOAc) to give **7d** (86.8 mg, 191 µmol, 64% yield) as a yellow oil.

R_f = 0.45 (30% EtOAc in pentane).

^{1}H NMR (400 MHz, $CDCl_3$) δ 7.31 (d, *J* = 8.7 Hz, 2H, Ar*H*), 7.27 – 7.22 (m, 2H, Ar*H*), 7.16 (d, *J* = 8.0 Hz, 2H, Ar*H*), 7.11 (t, *J* = 8.0 Hz, 1H, Ar*H*), 6.78 (ddd, *J* = 8.3, 2.7, 1.0 Hz, 1H, Ar*H*), 6.57 – 6.48 (m, 3H, Ar*H*), 6.21 (dd, *J* = 2.6, 1.6 Hz, 1H, Ar*H*), 6.14 (t, *J* = 2.1 Hz, 1H, C=C*H*), 4.55 – 4.35 (m, 2H, NCH_2), 3.73 – 3.61 (m, 1H, HOCH_2), 3.59 – 3.52 (m, 4H, HOCH_2, ArOCH_3), 2.99 (ddd, *J* = 13.7, 9.2, 5.9 Hz, 1H, HOCH$_2$CH_2), 2.47 (ddd, *J* = 14.0, 8.9, 5.4 Hz, 1H, HOCH$_2$CH_2), 2.35 (s, 3H, ArCH_3). O*H* was not resolved.

^{13}C{^{1}H} NMR (101 MHz, $CDCl_3$) δ 159.2, 147.6, 146.9, 141.0, 137.6, 135.8, 129.8, 129.3, 126.9, 126.3 (q, $J_{C\text{-}F}$ = 3.7 Hz), 125.2 (q, $J_{C\text{-}F}$ = 270.1 Hz), 121.7, 120.8, 117.7 (q, $J_{C\text{-}F}$ = 32.5 Hz), 114.2, 113.2, 112.3, 75.0, 59.3, 56.2, 55.1, 34.5, 21.2.

^{19}F NMR (376 MHz, $CDCl_3$) δ -60.9.

IR (cm^{-1}) 3435 (w), 2972 (m), 2900 (m), 2360 (w), 2250 (w), 1613 (m), 1526 (m), 1368 (m), 1322 (s), 1162 (m), 1108 (s), 1067 (s), 909 (m).

HRMS (nanochip-ESI/LTQ-Orbitrap) m/z: $[M + H]^+$ Calcd for $C_{27}H_{27}F_3NO_2^+$ 454.1988; Found 454.1996.

2-(3-(4-Chlorophenyl)-2-(*p*-tolyl)-1-(4-(trifluoromethyl)phenyl)-2,5-dihydro-1H-pyrrol-2-yl)ethan-1-ol (7e)

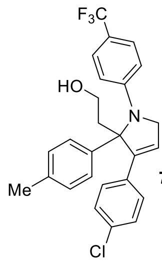

7e

Prepared according to the general procedure D3 using **4aqa** (104.2 mg, 300 µmol, 1.00 equiv.) and **3e** (102 mg, 390 µmol, 1.30 equiv.). The crude material was purified by column chromatography (15 – 50 % (v/v) hexanes in EtOAc) to give **7e** (20.8 mg, 45.4 µmol, 16% yield) as a yellow oil.

R_f = 0.76 (30% EtOAc in pentane).

^{1}H NMR (400 MHz, $CDCl_3$) δ 7.30 (d, *J* = 8.7 Hz, 2H, Ar*H*), 7.19 (d, *J* = 7.9 Hz, 2H, Ar*H*), 7.17 – 7.11 (m, 4H, Ar*H*), 6.75 – 6.67 (m, 2H, Ar*H*), 6.51 (d, *J* = 8.7 Hz, 2H, Ar*H*), 6.12 (t, *J* = 2.1 Hz, 1H, C=C*H*), 4.55 – 4.33 (m, 2H, NCH_2), 3.71 – 3.52 (m, 2H, HOCH_2), 2.98 (ddd, *J* = 13.8, 9.2, 5.9 Hz, 1H, HOCH$_2$CH_2), 2.41 (ddd, *J* = 14.0, 8.7, 5.4 Hz, 1H, HOCH$_2$CH_2), 2.35 (s, 3H, ArCH_3). O*H* was not resolved.

^{13}C{^{1}H} NMR (101 MHz, $CDCl_3$) δ 158.1, 146.8, 146.8, 140.5, 137.7, 134.2, 132.9, 129.9, 129.6, 128.5, 126.3 (q, $J_{C\text{-}F}$ = 3.7 Hz), 125.2 (q, $J_{C\text{-}F}$ = 270.2 Hz), 122.1, 117.9 (q, $J_{C\text{-}F}$ = 32.7 Hz), 112.3, 75.0, 59.2, 56.3, 34.4, 21.3.

^{19}F NMR (376 MHz, $CDCl_3$) δ -61.0.

IR (cm^{-1}) 3413 (w), 2969 (m), 2898 (m), 2360 (w), 2248 (w), 1904 (w), 1614 (m), 1527 (m), 1367 (m), 1322 (s), 1161 (m), 1107 (s), 1066 (s), 908 (m).

HRMS (nanochip-ESI/LTQ-Orbitrap) m/z: $[M + H]^+$ Calcd for $C_{26}H_{24}ClF_3NO^+$ 458.1493; Found 458.1504.

2-(3-(4-Fluorophenyl)-2-(*p*-tolyl)-1-(4-(trifluoromethyl)phenyl)-2,5-dihydro-1H-pyrrol-2-yl)ethan-1-ol (7f)

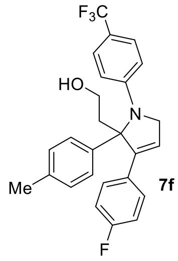

7f

Prepared according to the general procedure D3 using **4aqa** (104.2 mg, 300 µmol, 1.00 equiv.) and **3f** (102 mg, 390 µmol, 1.30 equiv.). The crude material was purified by column chromatography (15 – 50 % (v/v) hexanes in EtOAc) to give **7f** (101 mg, 228 µmol, 76% yield) as a yellow oil.

R_f = 0.59 (30% EtOAc in pentane).

^{1}H NMR (400 MHz, $CDCl_3$) δ 7.31 (d, *J* = 8.8 Hz, 2H, Ar*H*), 7.24 – 7.12 (m, 4H, Ar*H*), 6.90 – 6.82 (m, 2H, Ar*H*), 6.79 – 6.72 (m, 2H, Ar*H*), 6.57 – 6.47 (m, 2H, Ar*H*), 6.10 – 6.07 (m, 1H, C=C*H*), 4.55 – 4.35 (m, 2H, NCH_2), 3.66 (ddd, *J* = 10.6, 9.2, 5.3 Hz, 1H, HOCH_2), 3.55 (ddd, *J* = 10.6, 8.9, 5.8 Hz, 1H, HOCH_2), 2.97 (ddd, *J* = 13.8, 9.3, 5.9 Hz, 1H, HOCH$_2$CH_2), 2.41 (ddd, *J* = 14.1, 8.9, 5.4 Hz, 1H, HOCH$_2$CH_2), 2.36 (s, 3H, ArCH_3). O*H* was not resolved.

^{13}C{^{1}H} NMR (101 MHz, $CDCl_3$) δ 162.6 (d, $J_{C\text{-}F}$ = 247.7 Hz), 146.8, 146.7, 140.5, 137.6, 130.4 (d, $J_{C\text{-}F}$ = 3.4 Hz), 129.9 (d, $J_{C\text{-}F}$ = 8.0 Hz), 129.7, 126.7, 126.2 (q, $J_{C\text{-}F}$ = 3.8 Hz), 125.1 (q, $J_{C\text{-}F}$ = 270.4 Hz), 121.7, 117.7 (q, $J_{C\text{-}F}$ = 32.6 Hz), 115.1 (d, $J_{C\text{-}F}$ = 21.3 Hz), 112.2, 74.9, 59.1, 56.1, 34.3, 21.1.

^{19}F NMR (376 MHz, $CDCl_3$) δ -60.9, -113.6 – -113.7 (m).

IR (cm^{-1}) 3430 (w), 2975 (m), 2898 (m), 2360 (w), 2250 (w), 1896 (w), 1614 (m), 1509 (m), 1369 (m), 1322 (s), 1228 (m), 1161 (m), 1108 (s), 1067 (s), 908 (m), 814 (m).

HRMS (nanochip-ESI/LTQ-Orbitrap) m/z: $[M + H]^+$ Calcd for $C_{26}H_{24}F_4NO^+$ 442.1789; Found 442.1800.

2-(3-(4-Nitrophenyl)-2-(*p*-tolyl)-1-(4-(trifluoromethyl)phenyl)-2,5-dihydro-1H-pyrrol-2-yl)ethan-1-ol (7g)

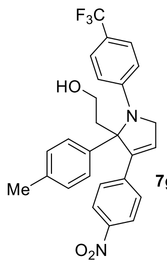

7g

Prepared according to the general procedure D3 using **4aqa** (34.7 mg, 100.0 µmol, 1.00 equiv.) and **S9** (35.2 mg, 130 µmol, 1.30 equiv.). The crude material was purified by column chromatography (15 – 50 % (v/v) hexanes in EtOAc) to give **7g** (46.8 mg, 90.7 µmol, 91% yield) as a yellow solid.

R_f(50% hexane/EtOAc) = 0.53.

^{1}H NMR (400 MHz, $CDCl_3$) δ 8.03 (d, *J* = 8.9 Hz, 2H, Ar*H*), 7.31 (d, *J* = 8.7 Hz, 2H, Ar*H*), 7.19 (q, *J* = 7.9 Hz, 4H, Ar*H*), 6.97 (d, *J* = 8.9 Hz, 2H, Ar*H*), 6.52 (d, *J* = 8.7 Hz, 2H, Ar*H*), 6.28 (t, *J* = 2.1 Hz, 1H, C=C*H*), 4.60 – 4.43 (m, 2H, NCH_2), 3.63 (tdd, *J* = 13.4, 10.5, 5.1 Hz, 2H, HOCH_2), 3.03 (ddd, *J* = 14.4, 8.8, 6.0 Hz, 1H, HOCH$_2$CH_2), 2.47 – 2.37 (m, 1H, HOCH$_2$CH_2), 2.36 (s, 3H, ArCH_3), 1.21 (t, *J* = 5.5 Hz, 1H, O*H*).

^{13}C{^{1}H} NMR (101 MHz, $CDCl_3$) δ 147.5, 146.6, 146.1, 141.1, 138.2, 130.1, 129.1, 126.4 (q, J_{C-F} = 3.8 Hz), 124.3, 123.8, 123.5, 123.0 (d, J_{C-F} = 270.4 Hz), 118.3 (q, J_{C-F} = 32.5 Hz), 112.4, 75.1, 59.1, 56.4, 34.5, 21.3.

^{19}F NMR (376 MHz, $CDCl_3$) δ -61.0.

IR (cm^{-1}) 3410 (w), 2956 (w), 2826 (w), 1614 (m), 1518 (m), 1321 (s).

HRMS (ESI/QTOF) m/z: [M + H]+ Calcd for $C_{26}H_{24}F_3N_2O_3^+$ 469.1734; Found 469.1729.

D.5. Unsuccessful substrates

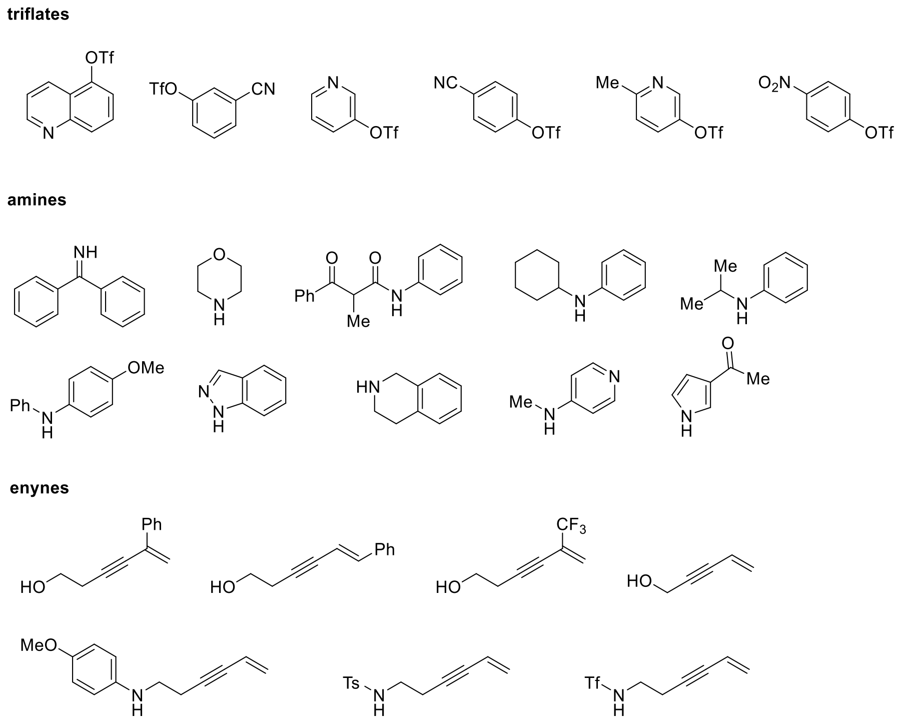

Amine directing group side-reactivity

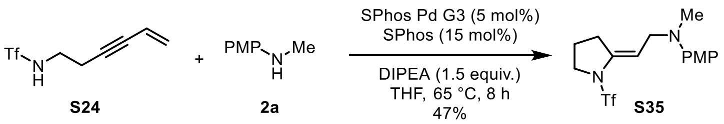

An oven-dried 8 mL microwave tube equipped with a Teflon coated stirring bar was charged with SPhos Pd G3 (3.9 mg, 15 µmol, 5 mol%), SPhos (6.2 mg, 5.0 µmol, 15 mol%) and 4-methoxy-*N*-methylaniline **2a** (17.8 mg, 0.130 mmol, 1.30 equiv.). The tube was evacuated and back-filled with N_2 three times. Then, THF (0.2 mL), DIPEA (26.0 µL, 150 µmol, 1.5 equiv.) and 1,1,1-trifluoro-*N*-(hex-5-en-3-yn-1-yl)methanesulfonamide **S24** (22.7 mg, 100 µmol, 1.00 equiv.) were added and the mixture was stirred at 65 °C for 8 h. Next, the reaction mixture was allowed to cool down to room temperature and concentrated under vacuum. The crude material was purified by preparative TLC on silica gel (20% (v/v) EtOAc/hexane) to afford **S35** (17.1 mg, 0.470 µmol, 47% yield).

^{1}H NMR (400 MHz, $CDCl_3$) δ 6.88 – 6.71 (m, 4H, Ar*H*), 5.71 (tt, *J* = 7.0, 2.2 Hz, 1H, =C*H*), 3.84 – 3.71 (m, 7H, CH_2N and OCH_3), 2.78 (s, 3H, NCH_3), 2.62 (td, *J* = 7.5, 2.1 Hz, 2H, CH_2C=), 1.96 (p, *J* = 7.2 Hz, 2H, CH_2).

^{13}C NMR (101 MHz, $CDCl_3$) δ 152.6, 144.6, 137.9, 120.4 (q, J_{C-F} = 326.2 Hz), 116.5, 114.6, 106.7, 55.7, 53.0, 51.9, 38.7, 28.5, 22.4.

^{19}F NMR (376 MHz, $CDCl_3$) δ -73.6.

HRMS (ESI/QTOF) m/z: $[M + H]^+$ Calcd for $C_{15}H_{20}F_3N_2O_3S^+$ 365.1141; Found 365.1124.

E. Product modifications

E.1. Alcohol cyclization

4-Methoxy-*N*-methyl-*N*-((4-(*p*-tolyl)-5,6-dihydro-2*H*-pyran-2-yl)methyl)aniline (8)

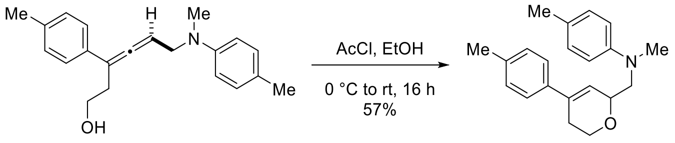

4ada **8**

Acetyl chloride (0.85 mL, 12 mmol, 15 equiv.) was added dropwise *via* an addition funnel to a flask containing anhydrous EtOH (6 mL) at 0 °C. The mixture was stirred for 30 minutes then a solution of **4ada** (246 mg, 800 µmol, 1.0 equiv.) in anhydrous EtOH (3 mL) was added dropwise, and the mixture was stirred for an additional 16 hours. The reaction was then quenched with sat. aq. $NaHCO_3$ (10 mL) and extracted with EtOAc (3 × 10 mL). The combined organics were dried over anhydrous Na_2SO_4, filtered, and the solvents were removed in vacuum. The crude residue was purified by column chromatography (9:1 hexane/EtOAc) on silica gel to give **8** (141 mg, 459 µmol, 57% yield) as a red oil.

R_f (20% EtOAc/hexane) = 0.62.

^1H NMR (400 MHz, $CDCl_3$) δ 7.28 (dd, J = 8.1, 2.9 Hz, 2H, Ar*H*), 7.15 (dd, J = 8.0, 2.2 Hz, 2H, Ar*H*), 7.06 (dd, J = 8.4, 2.2 Hz, 2H, Ar*H*), 6.72 (d, J = 8.1 Hz, 2H, Ar*H*), 6.06 (s, 1H, C=C*H*), 4.55 (hept, J = 2.8 Hz, 1H, C=CHC*H*), 4.15 (dtd, J = 12.4, 6.3, 3.0 Hz, 1H, OCH_aH_b), 3.81 – 3.70 (m, 1H, OCH_aH_b), 3.55 – 3.39 (m, 2H, NCH_2), 3.02 (d, J = 2.1 Hz, 3H, CH_3), 2.73 – 2.59 (m, 1H, $OCH_2CH_aH_b$), 2.39 (t, J = 2.7 Hz, 1H, $OCH_2CH_aH_b$), 2.35 (d, J = 2.7 Hz, 3H, CH_3), 2.27 (d, J = 2.9 Hz, 3H, CH_3).

$^{13}C\{^1H\}$ NMR (101 MHz, $CDCl_3$) δ 147.6, 137.40, 137.36, 135.4, 129.8, 129.3, 125.8, 124.8, 123.4, 112.8, 73.0, 63.8, 58.1, 39.8, 27.4, 21.2, 20.4.

IR (cm^{-1}) 3396 (m), 2863 (w), 2360 (s), 1682 (m), 1622 (m), 1514 (s).

HRMS (ESI/QTOF) m/z: $[M + H]^+$ Calcd for $C_{21}H_{26}NO^+$ 308.2009; Found 308.2008.

***N*,4-Dimethyl-*N*-((4-(*p*-tolyl)tetrahydro-2*H*-pyran-2-yl)methyl)aniline (9)**

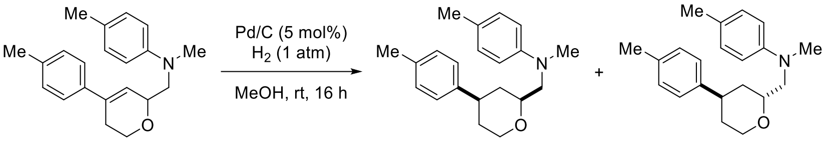

8 **9a**, 71% **9b**, 21%

An oven-dried 25 mL round-bottom flask equipped with a Teflon coated stirring bar was charged with Pd/C (5.3 mg, 5.0 µmol, 5 mol%) and **8** (30.7 mg, 0.100 mmol, 1.00 equiv.). The flask was sealed and evacuated and back-filled with N_2 three times. MeOH (2 mL) was added and the suspension was stirred at room temperature for 10 minutes under a nitrogen flow. Then, a hydrogen balloon was connected to the flask through a needle and the mixture was vigorously stirred at room temperature for 16 hours. Then, the reaction mixture was degassed by bubbling nitrogen for 10 minutes and filtered through a plug of celite eluting with 10 mL of EtOAc and concentrated in vacuum. The crude material was purified by flash column chromatography (8:2 hexane/EtOAc) on silica gel to afford **9a** (22 mg, 71 µmol, 71% yield) and **9b** (6.5 mg, 21 µmol, 21% yield). Diastereoselectivity was assigned by 1D NOE.

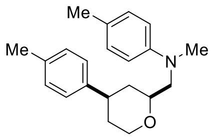

9a

R_f (20 % EtOAc/hexane) = 0.68.

^1H NMR (400 MHz, $CDCl_3$) δ 7.19 – 7.09 (m, 4H, Ar*H*), 7.06 (d, J = 8.4 Hz, 2H, Ar*H*), 6.66 (d, J = 8.5 Hz, 2H, Ar*H*), 4.14 (ddd, J = 11.4, 4.1, 2.0 Hz, 1H, OCH_aCH_b), 3.78 – 3.67 (m, 1H, OCH_aCH_b), 3.56 (td, J = 11.4, 3.3 Hz, 1H, NCH_2CH), 3.45 (dd, J = 15.0, 6.5 Hz, 1H, NCH_aH_b), 3.30 (dd, J = 15.0, 5.3 Hz, 1H, NCH_aH_b), 2.98 (s, 3H, CH_3), 2.82 – 2.68 (m, 1H, ArC*H*), 2.34 (s, 3H, CH_3), 2.27 (s, 3H, CH_3), 1.88 (dt, J = 12.9, 1.8 Hz, 1H, $ArCHCH_2$), 1.84 – 1.68 (m, 2H, $ArCHCH_2$), 1.54 – 1.40 (m, 1H, $ArCHCH_2$).

$^{13}C\{^1H\}$ NMR (101 MHz, $CDCl_3$) δ 147.6, 142.9, 136.0, 129.8, 129.4, 126.8, 125.5, 112.5, 76.1, 68.4, 58.9, 41.2, 40.1, 38.0, 33.7, 21.1, 20.3.

IR (cm^{-1}) 3427 (w), 3042 (w), 2921 (w), 2856 (w), 1618 (m), 1519 (s).

HRMS (nanochip-ESI/LTQ-Orbitrap) m/z: $[M + H]^+$ Calcd for $C_{21}H_{28}NO^+$ 310.2165; Found 310.2170.

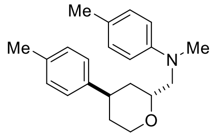

9b

$\underline{R_f}$(20 % EtOAc/hexane) = 0.64.

<u>1H NMR</u> (400 MHz, $CDCl_3$) δ 7.12 (s, 4H, Ar*H*), 7.04 (d, *J* = 8.4 Hz, 2H, Ar*H*), 6.66 (d, *J* = 8.0 Hz, 2H, Ar*H*), 4.08 (p, *J* = 6.1 Hz, 1H, NCH_2CH), 3.84 (ddd, *J* = 11.4, 7.0, 4.2 Hz, 1H, OCH_aCH_b), 3.74 (ddd, *J* = 11.6, 6.2, 4.1 Hz, 1H, OCH_aCH_b), 3.49 (qd, *J* = 14.8, 6.4 Hz, 2H, NCH_2), 3.08 (tt, *J* = 8.0, 4.7 Hz, 1H, ArC*H*), 2.97 (s, 3H, CH_3), 2.32 (s, 3H, CH_3), 2.25 (s, 3H, CH_3), 2.01 (ddd, *J* = 12.5, 8.2, 4.2 Hz, 1H, $ArCHCH_2$), 1.92 (ddt, *J* = 9.4, 6.3, 3.3 Hz, 2H, $ArCHCH_2$), 1.81 (dt, *J* = 13.7, 5.1 Hz, 1H, $ArCHCH_2$).

<u>$^{13}C\{^1H\}$ NMR</u> (101 MHz, $CDCl_3$) δ 147.7, 141.7, 135.8, 129.9, 129.3, 127.2, 125.8, 112.8, 71.1, 62.9, 55.8, 39.6, 35.0, 34.3, 32.2, 21.1, 20.4.

<u>IR</u> (cm^{-1}) 3393 (w), 3039 (w), 2923 (m), 2855 (m), 1732 (w), 1618 (m), 1519 (s).

<u>HRMS</u> (nanochip-ESI/LTQ-Orbitrap) m/z: $[M + H]^+$ Calcd for $C_{21}H_{28}NO^+$ 310.2165; Found 310.2172.

E.2. Mukaiyama hydration

Methyl 4-(3-hydroxy-2-(2-hydroxyethyl)-2-(*p*-tolyl)-1-(4-(trifluoromethyl)phenyl)pyrrolidin-3-yl)benzoate (10)

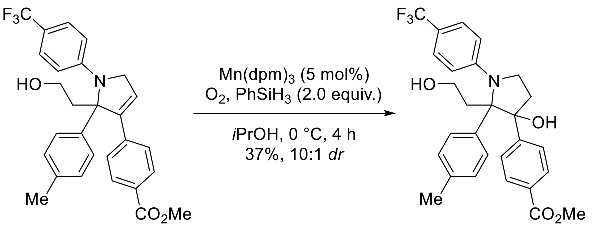

Following a reported procedure,[30] to a stirred solution of **7a** (99.2 mg, 0.206 mmol, 1.0 equiv.) and $Mn(dpm)_3$ (6.2 mg, 10 μmol, 5 mol%) in 2-propanol (10 mL) at 0 °C was added phenylsilane (51 μL, 0.41 mmol, 2.0 equiv.). The reaction mixture was stirred at 0 °C under O_2 atmosphere for 4 h. After being quenched with sat. $Na_2S_2O_3$ (10 mL), the reaction mixture was diluted with water, extracted with EtOAc (3×50 mL). The combined organic layers were washed with brine and dried over Na_2SO_4. The crude material was purified by column chromatography on silica gel (10 – 50% (v/v) EtOAc/hexane) to afford **10** (37.8 mg, 757 μmol, 37% yield) as a pale-yellow solid in 10:1 *dr*.

Major diastereomer:

$\underline{R_f}$(50 % EtOAc/hexane) = 0.48.

<u>1H NMR</u> (400 MHz, $CDCl_3$) δ 7.99 (d, *J* = 8.5 Hz, 2H, Ar*H*), 7.26 (d, *J* = 8.7 Hz, 2H, Ar*H*), 7.15 (dd, *J* = 14.6, 8.4 Hz, 4H, Ar*H*), 6.87 (d, *J* = 8.3 Hz, 2H, Ar*H*), 6.35 (d, *J* = 8.5 Hz, 2H, Ar*H*), 4.08 – 3.96 (m, 1H, CH_2OH), 3.92 (s, 3H, CO_2CH_3), 3.79 – 3.71 (m, 1H, CH_2OH), 3.67 (td, *J* = 10.2, 6.2 Hz, 1H, NCH_2), 3.49 (td, *J* = 9.9, 5.1 Hz, 1H, NCH_2), 3.04 (dt, *J* = 12.7, 9.6 Hz, 1H, CH_2CH_2OH), 2.52 – 2.40 (m, 1H, NCH_2CH_2), 2.38 (s, 3H, CH_3), 2.36 – 2.31 (m, 1H, CH_2CH_2OH), 2.14 – 2.01 (m, 1H, NCH_2CH_2). O*H* were not resolved.

<u>$^{13}C\{^1H\}$ NMR</u> (101 MHz, $CDCl_3$) δ 166.9, 148.1, 145.9, 138.3, 134.4, 129.8, 129.3 (2C), 128.8, 127.1, 125.9 (q, J_{C-F} = 3.7 Hz), 125.2 (q, J_{C-F} = 270.1 Hz), 117.6 (q, J_{C-F} = 32.6 Hz), 113.4, 84.9, 74.8, 59.5, 52.4, 48.3, 39.2, 34.0, 21.2.

<u>^{19}F NMR</u> (376 MHz, $CDCl_3$) δ -60.9.

<u>IR</u> (cm^{-1}) 3457 (w), 2965 (w), 2863 (w), 2252 (w), 1720 (m), 1612 (m), 1525 (m), 1322 (s).

<u>HRMS</u> (ESI/QTOF) m/z: $[M + Na]^+$ Calcd for $C_{28}H_{28}F_3NNaO_4^+$ 522.1863; Found 522.1870.

E.3. Hydroamination and hydrogenation

2-(2-(*p*-Tolyl)-1-(4-(trifluoromethyl)phenyl)-2,5-dihydro-1*H*-pyrrol-2-yl)ethan-1-ol (11)

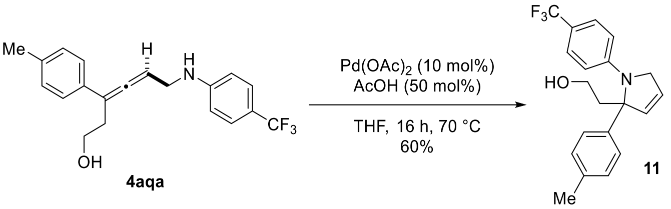

To a microwave tube equipped with a magnetic stir bar were added **4aqa** (347 mg, 1.00 mmol, 1.00 equiv.), $Pd(OAc)_2$ (21 mg, 0.10 mmol, 10 mol%), and acetic acid (29 μL, 0.50 mmol, 50 mol%). The tube was evacuated and backfilled with nitrogen three times. Subsequently, THF (2 mL) was added. The mixture was stirred at 70 °C for 16 h. After 16 h, the reaction was cooled to room temperature, diluted with EtOAc and concentrated in vacuo. Crude material was purified by column chromatography ins silica gel (20% (v/v) EtOAc/hexane) to afford **11** (208 mg, 599 μmol, 60% yield).

Rf = 0.53 (30% EtOAc in pentane).

^{1}H NMR (400 MHz, $CDCl_3$) δ 7.31 (d, J = 8.7 Hz, 2H, ArH), 7.13 (s, 4H, ArH), 6.45 (d, J = 8.7 Hz, 2H, ArH), 5.95 (dt, J = 6.2, 2.0 Hz, 1H, CH=), 5.70 (dt, J = 6.2, 2.1 Hz, 1H, CH=), 4.39 (s, 2H, CH_2N), 3.65 (ddd, J = 10.7, 8.5, 5.9 Hz, 1H, CH_2OH), 3.58 – 3.45 (m, 1H, CH_2OH), 2.95 (ddd, J = 14.2, 8.5, 5.9 Hz, 1H, CH_2), 2.55 (ddd, J = 14.0, 8.1, 5.9 Hz, 1H, CH_2), 2.32 (s, 3H, ArCH_3), 1.58 (s, 1H, OH).

^{13}C{^{1}H} NMR (101 MHz, $CDCl_3$) δ 146.8, 140.8, 137.9, 137.0, 129.7, 126.2 (q, $J_{C\text{-}F}$ = 3.8 Hz), 125.6, 125.1 (q, $J_{C\text{-}F}$ = 271.4 Hz), 122.0, 117.6 (q, $J_{C\text{-}F}$ = 32.6 Hz), 112.4, 73.5, 59.2, 57.3, 36.4, 21.0.

^{19}F NMR (376 MHz, $CDCl_3$) δ -60.9.

IR (cm^{-1}) 3393 (w), 2933 (w), 2855 (w), 2363 (w), 1612 (m), 1528 (m), 1371 (m), 1329 (s).

HRMS (ESI/QTOF) m/z: $[M + H]^+$ Calcd for $C_{20}H_{21}F_3NO^+$ 348.1570; Found 348.1574.

2-(2-(*p*-Tolyl)-1-(4-(trifluoromethyl)phenyl)pyrrolidin-2-yl)ethan-1-ol (12)

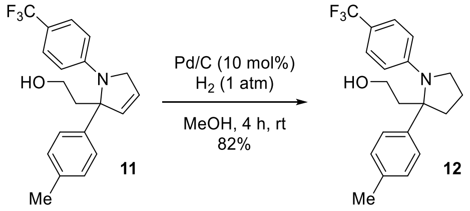

An oven-dried 25 mL round-bottom flask equipped with a Teflon coated stirring bar was charged with Pd/C (7.4 mg, 6.9 µmol, 5 mol%) and **11** (48.1 mg, 0.14 mmol, 1.00 equiv.). The flask was sealed and evacuated and back-filled with N_2 three times. MeOH (2 mL) was added and the suspension was stirred at room temperature for 10 minutes under a nitrogen flow. Then, a hydrogen balloon was connected to the flask through a needle and the mixture was vigorously stirred at room temperature for 4 hours. Then, the reaction mixture was degassed by bubbling nitrogen for 10 minutes and filtered through a plug of celite eluting with 10 mL of EtOAc and concentrated in vacuum. The crude material was purified by flash column chromatography (8:2 hexane/EtOAc) on silica gel to afford **12** (39 mg, 0.11 mmol, 82% yield).

Rf (20% EtOAc/hexane) = 0.43.

^{1}H NMR (400 MHz, $CDCl_3$) δ 7.37 (d, J = 8.5 Hz, 2H, ArH), 7.12 (d, J = 7.9 Hz, 2H, ArH), 7.06 (d, J = 8.1 Hz, 2H, ArH), 6.54 (d, J = 8.5 Hz, 2H, ArH), 3.55 (ddd, J = 10.5, 6.5, 5.6 Hz, 1H, NCH_2), 3.46 (ddd, J = 10.6, 7.6, 6.2 Hz, 1H, NCH_2), 3.06 (hept, J = 6.3 Hz, 2H, HOCH_2), 2.71 (tt, J = 10.0, 5.1 Hz, 1H, $NCH_2CH_2CH_2$), 2.33 (s, 3H, CH_3), 1.98 – 1.87 (m, 1H, $NCH_2CH_2CH_2$), 1.86 – 1.72 (m, 2H, NCH_2CH_2), 1.72 – 1.61 (m, 1H, $NCH_2CH_2CH_2$), 1.60 – 1.49 (m, 1H, $HOCH_2CH_2$), 1.49 – 1.38 (m, 1H, $HOCH_2CH_2$). OH was not resolved.

^{13}C{^{1}H} NMR (101 MHz, $CDCl_3$) δ 149.9, 141.3, 136.1, 129.5, 127.6, 126.7 (q, J = 4.0 Hz), 125.0 (q, J = 270.5 Hz), 112.7, 67.8, 61.1, 41.9, 39.9, 34.3, 27.1, 21.2.

^{19}F NMR (376 MHz, $CDCl_3$) δ -61.0.

IR (cm^{-1}) 3401 (w), 2928 (w), 2868 (w), 1731 (w), 1616 (m), 1535 (w), 1325 (s).

HRMS (ESI/QTOF) m/z: $[M + H]^+$ Calcd for $C_{20}H_{23}F_3NO^+$ 350.1726; Found 350.1726.

E.4. Macrocyclization

6-(Methyl(*p*-tolyl)amino)-3-(*p*-tolyl)hexa-3,4-dien-1-yl sulfamate (13)

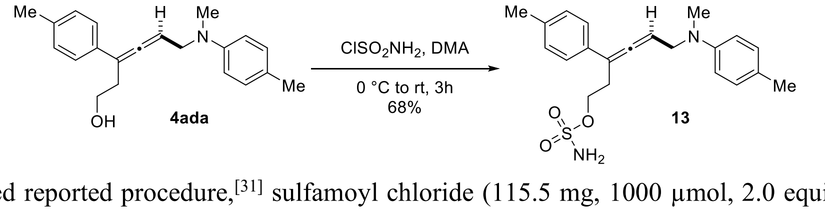

Following a modified reported procedure,[31] sulfamoyl chloride (115.5 mg, 1000 µmol, 2.0 equiv.) was added to a stirred solution of **4ada** (153.7 mg, 500.0 µmol, 1.0 equiv.) in dry DMA (0.85 mL) at 0 °C. The mixture was stirred at room temperature for 3 h and then poured into 50 mL of cold aqueous brine. The resulting solution was extracted with chloroform (3×10 mL), the combined organic layers were washed with cold brine (3×50 mL) and dried over Na_2SO_4. After concentration under reduced pressure, the crude product was purified by a short plug of silica gel (hexane/EtOAc = 8:2) affording **13** (131 mg, 0.340 mmol, 68% yield) as a yellow oil.

Rf (10% EtOAc/hexane) = 0.11.

^{1}H NMR (400 MHz, $CDCl_3$) δ 7.18 (d, J = 8.2 Hz, 2H, ArH), 7.11 (d, J = 8.1 Hz, 2H, ArH), 7.05 (d, J = 8.3 Hz, 2H, ArH), 6.73 (d, J = 8.1 Hz, 2H, ArH), 5.60 (tt, J = 5.7, 3.1 Hz, 1H, C=CH), 4.76 (s, 2H, NH_2), 4.26 – 4.13 (m, 2H, OCH_2), 4.04 (d, J = 5.8 Hz, 2H, NCH_2), 2.92 (s, 3H, NCH_3), 2.76 (td, J = 7.1, 3.0 Hz, 2H, OCH_2CH_2), 2.33 (s, 3H, ArCH_3), 2.26 (s, 3H, ArCH_3).

$^{13}C\{^{1}H\}$ NMR (101 MHz, $CDCl_3$) δ 203.9, 146.7, 137.0, 132.4, 129.9, 129.4, 126.9, 125.8, 114.1, 102.4, 91.7, 69.2, 52.3, 39.1, 29.2, 21.2, 20.4.

IR (cm^{-1}) 3268 (w), 3031 (w), 2919 (m), 2859 (w), 1947 (w), 1619 (w), 1518 (s), 1366 (s).

HRMS (ESI/QTOF) m/z: $[M + H]^+$ Calcd for $C_{21}H_{27}N_2O_3S^+$ 387.1737; Found 387.1737.

5,9-Bis(4-methylphenyl)-1-oxa-3,5-diaza-2,2-dioxo-2-thiacycloundeca-3,7,8-triene (14)

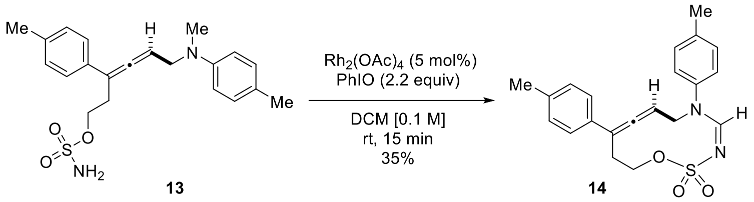

Following a modified reported procedure,[31] **13** (38.7 mg, 100 µmol, 1.0 equiv.) and $Rh_2(OAc)_4$ (2.2 mg, 5.0 µmol, 0.05 equiv.) were placed in a dry round bottom flask. The solids were dissolved in CH_2Cl_2 (0.1 M), and the resulting solution was stirred for 5 min. PhIO (48.4 mg, 220 µmol, 2.2 equiv.) was added in a single portion and the reaction was stirred for 15 min. Then, the solvent was removed via rotary evaporation and the crude reaction mixture was immediately purified via column chromatography to afford **14** (13.7 mg, 346 µmol, 35% yield) as a pale-yellow solid.

R_f(20% EtOAc/hexane) = 0.12.

^1H NMR (400 MHz, $CDCl_3$) δ 8.35 (s, 1H, N=C*H*), 7.14 (dd, *J* = 8.3, 4.0 Hz, 4H, Ar*H*), 7.07 (d, *J* = 8.1 Hz, 2H, Ar*H*), 7.01 (d, *J* = 8.4 Hz, 2H, Ar*H*), 5.65 (h, *J* = 2.5 Hz, 1H, C=C*H*), 5.04 (dd, *J* = 16.3, 2.5 Hz, 1H, NCH_2), 4.78 (ddd, *J* = 10.9, 9.2, 3.2 Hz, 1H, OCH_2), 4.40 (ddd, *J* = 10.9, 3.9, 2.8 Hz, 1H, OCH_2), 4.19 (dd, *J* = 16.2, 4.8 Hz, 1H, NCH_2), 2.92 – 2.75 (m, 2H, OCH_2CH_2), 2.35 (s, 3H, CH_3), 2.32 (s, 3H, CH_3).

$^{13}C\{^{1}H\}$ NMR (101 MHz, $CDCl_3$) δ 202.9, 160.0, 139.9, 138.1, 137.5, 133.1, 130.5, 129.3, 126.3, 122.5, 107.7, 89.7, 68.1, 49.4, 30.6, 21.1.

IR (cm^{-1}) 2964 (w), 2923 (w), 2853 (w), 2255 (w), 1589 (s), 1514 (m), 1330 (m), 1158 (s).

HRMS (ESI/QTOF) m/z: $[M + Na]^+$ Calcd for $C_{21}H_{22}N_2NaO_3S^+$ 405.1243; Found 405.1245.

F. Mechanistic studies

HRMS Study

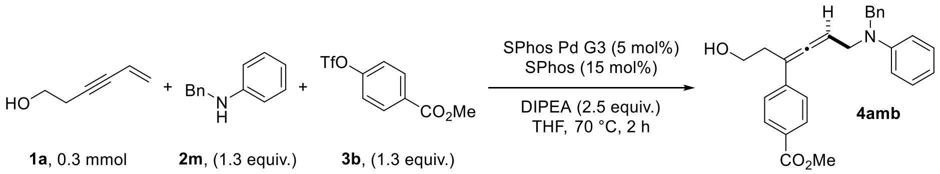

An oven-dried 8 mL microwave tube equipped with a Teflon coated stirring bar was charged with SPhos Pd G3 (11.7 mg, 15.0 µmol, 5 mol%), SPhos (18.5 mg, 45.0 µmol, 15 mol%) and amine **2m** (72 mg, 0.39 mmol, 1.3 equiv.). The tube was evacuated and back-filled with N_2 three times. Then, THF (0.6 mL) and DIPEA (131 µL, 750 µmol, 2.5 equiv.) were added and the mixture was stirred at 70 °C for 5 minutes. Afterwards, the corresponding enyne **1a** (28.8 mg, 300 µmol, 1.0 equiv.) and methyl 4-(trifluoromethylsulfonyloxy)benzoate **3b** (111 mg, 390 µmol, 1.30 equiv.). were added. The resulting solution was then stirred at 70 °C for 2 h. Then, an aliquot of 10 µL was diluted with 1 mL of THF and submitted for MS analysis.

HRMS (nanochip-ESI/LTQ-Orbitrap) m/z: $[M]^+$ Calcd for $C_{40}H_{50}O_5PPd^+$ 747.2425; Found 747.2460.

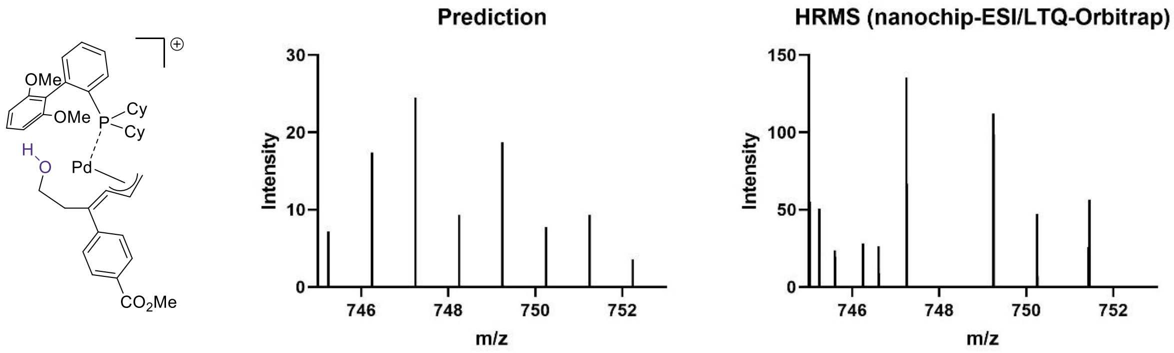

Cross nucleophile experiment

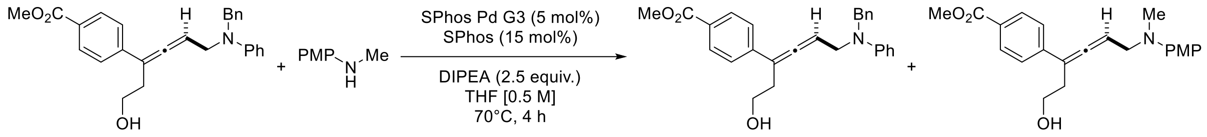

4amb **2a**, (1.3 equiv) **4amb**, 90% **4aab**, 10%

An oven-dried 8 mL microwave tube equipped with a Teflon coated stirring bar was charged with SPhos Pd G3 (1.1 mg, 1.5 µmol, 5 mol%), SPhos (1.8 mg, 4.4 µmol, 15 mol%), amine **2a** (5.2 mg, 38 µmol, 1.3 equiv.) and allene **4amb** (12 mg, 29 µmol, 1.0 equiv.). The tube was evacuated and back-filled with N_2 three times. Then, THF (0.1 mL) and DIPEA (7.3 µL, 73 µmol, 2.5 equiv.) were added and the mixture was stirred at 70 °C for 4 hours. Then, the solvent was evaporated under reduced pressure, and the mixture was analyzed by ^{1}H NMR using TCE as internal standard.

G. X-ray crystallography

3-(*p*-Tolyl)-6-((4-(trifluoromethyl)phenyl)amino)hexa-3,4-dien-1-ol (4aqa)

CCDC deposition Number 2498438

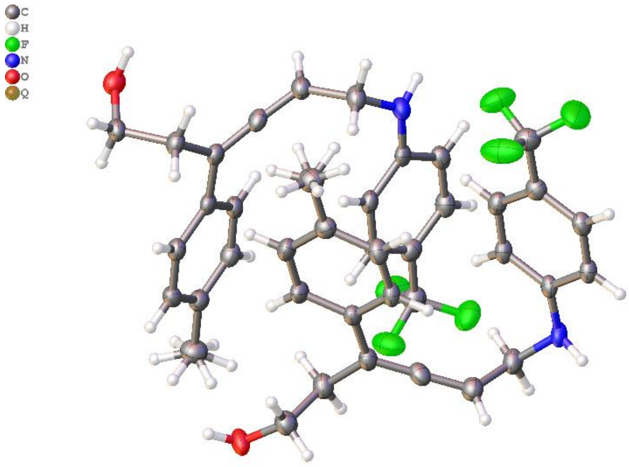

Crystals of compound **4aqa** were collected upon crystallization *via* vapour diffusion with Et_2O/pentane at 5 °C.

Experimental. Single clear pale colourless plate-shaped crystals of **4aqa** were used as supplied. A suitable crystal with dimensions 0.12 × 0.08 × 0.06 mm^3 was selected and mounted on a XtaLAB Synergy R, DW system, HyPix-Arc 150 diffractometer. The crystal was kept at a steady T = 100.01(11) K during data collection. The structure was solved with the ShelXT (Sheldrick, 2015) solution program using dual methods and by using Olex2 1.5 (Dolomanov et al., 2009) as the graphical interface. The model was refined with ShelXL 2019/3 (Sheldrick, 2015) using full matrix least squares minimisation on $\boldsymbol{F}^2$.

Crystal Data. $C_{20}H_{20}F_3NO$, M_r = 347.37, monoclinic, $P2_1/c$ (No. 14), a = 10.3869(3) Å, b = 20.2769(6) Å, c = 16.3577(6) Å, β = 91.230(3)°, $\alpha = \gamma = 90°$, V = 3444.37(19) Å^3, T = 100.01(11) K, Z = 8, Z' = 2, μ(Cu K_α) = 0.878, 27782 reflections measured, 6731 unique (R_{int} = 0.0436) which were used in all calculations. The final wR_2 was 0.1693 (all data) and R_1 was 0.0580 (I≥2 σ(I)).

**Compound**	**4aqa**
Formula	$C_{20}H_{20}F_3NO$
$D_{calc.}$/ g cm^{-3}	1.340
μ/mm^{-1}	0.878
Formula Weight	347.37
Colour	clear pale colourless
Shape	plate
Size/mm^3	0.12×0.08×0.06
T/K	100.01(11)
Crystal System	monoclinic
Space Group	$P2_1/c$
a/Å	10.3869(3)
b/Å	20.2769(6)
c/Å	16.3577(6)
α/°	90
β/°	91.230(3)
γ/°	90
V/Å^3	3444.37(19)
Z	8
Z'	2
Wavelength/Å	1.54184
Radiation type	Cu K_α
Θ_{min}/°	3.472
Θ_{max}/°	74.889
Measured Refl's.	27782
Indep't Refl's	6731
Refl's I≥2 σ(I)	4549
R_{int}	0.0436
Parameters	591
Restraints	0
Largest Peak	0.469
Deepest Hole	-0.301
GooF	1.043
wR_2 (all data)	0.1693
wR_2	0.1478
R_1 (all data)	0.0920
R_1	0.0580

Structure Quality Indicators

**Reflections:**	d min (CuKα) 2Θ=149.8° 0.80	I/σ(I) 22.7	Rint m=4.21 4.36%	Full 135.4° 95% to 149.8° 99.9
**Refinement:**	Shift 0.000	Max Peak 0.5	Min Peak -0.3	GooF 1.043

A clear pale colourless plate-shaped crystal with dimensions 0.12 × 0.08 × 0.06 mm^3 was mounted. Data were collected using a XtaLAB Synergy R, DW system, HyPix-Arc 150 diffractometer operating at T = 100.01(11) K.

Data were measured using ω scans with Cu K_α radiation. The diffraction pattern was indexed and the total number of runs and images was based on the strategy calculation from the program CrysAlisPro system (CCD 44.115a 64-bit (release 23-06-2025)). The maximum resolution that was achieved was Θ = 74.889° (0.80 Å).

The unit cell was refined using CrysAlisPro 1.171.44.115a (Rigaku OD, 2025) on 7903 reflections, 28% of the observed reflections.

Data reduction, scaling and absorption corrections were performed using CrysAlisPro 1.171.44.115a (Rigaku OD, 2025). The final completeness is 99.90 % out to 74.889° in Θ. An analytical absorption correction was performed

using CrysAlisPro 1.171.44.115a (Rigaku Oxford Diffraction, 2025). The analytical numeric absorption correction was done using a multifaceted crystal model based on expressions derived by R.C. Clark & J.S. Reid. (Clark, R. C. & Reid, J. S. (1995). Acta Cryst. A51, 887-897). The empirical absorption correction was done using spherical harmonics, implemented in SCALE3 ABSPACK scaling algorithm. The absorption coefficient μ of this crystal is 0.878 mm^{-1} at this wavelength (λ = 1.54184Å) and the minimum and maximum transmissions are 0.960 and 0.979.

The structure was solved and the space group $P2_1/c$ (# 14) determined by the ShelXT (Sheldrick, 2015) structure solution program using dual methods and refined by full matrix least squares minimisation on $\boldsymbol{F}^2$ using version 2019/3 of ShelXL (Sheldrick, 2015). All non-hydrogen atoms were refined anisotropically. Most hydrogen atom positions were calculated geometrically and refined using the riding model, but some hydrogen atoms were refined freely.

The value of Z' is 2. This means that there are two independent molecules in the asymmetric unit. The moiety formula is C20 H20 F3 N O.

Data Plots: Diffraction Data

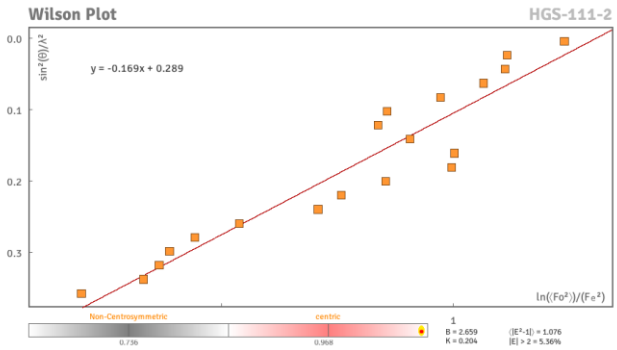

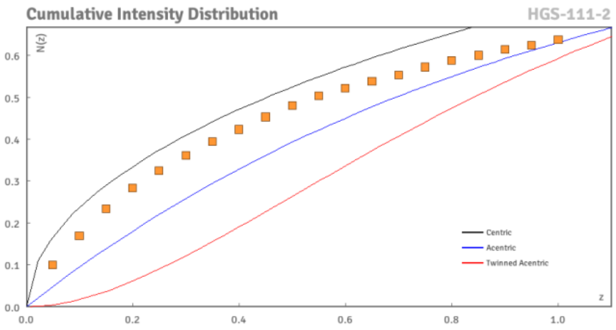

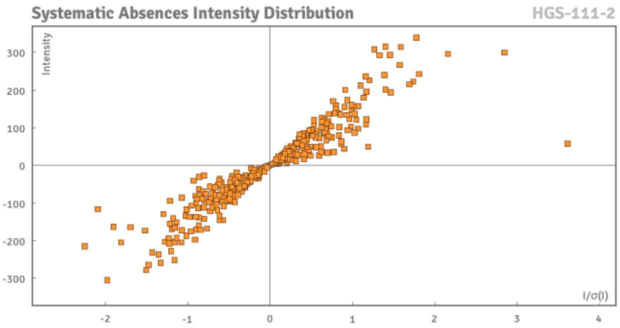

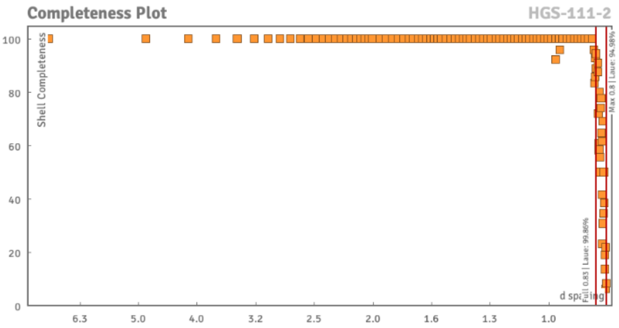

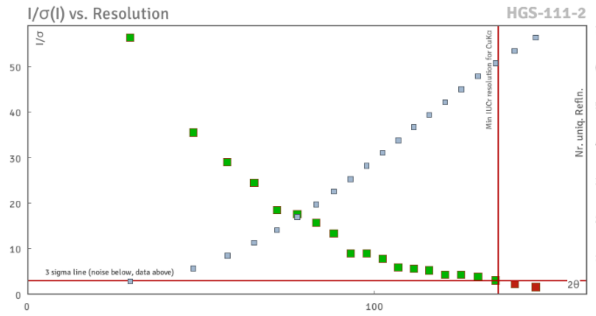

Data Plots: Refinement and Data

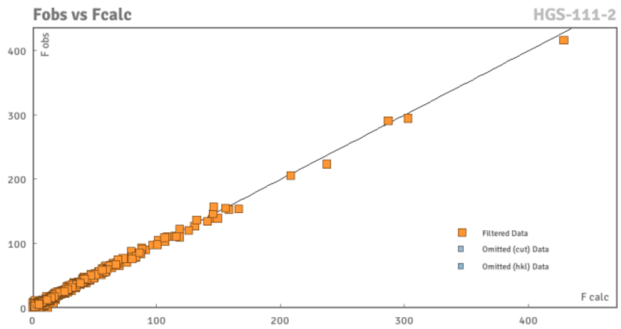

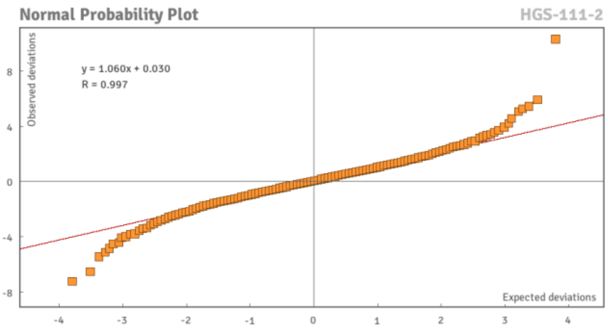

Reflection Statistics

Total reflections (after filtering)	28323	Unique reflections	6731
Completeness	0.95	Mean I/σ	14.2
hkl_{max} collected	(12, 20, 19)	hkl_{min} collected	(-12, -25, -19)
hkl_{max} used	(12, 25, 19)	hkl_{min} used	(-12, 0, 0)
Lim d_{max} collected	100.0	Lim d_{min} collected	0.77
d_{max} used	20.28	d_{min} used	0.8
Friedel pairs	4738	Friedel pairs merged	1
Inconsistent equivalents	13	R_{int}	0.0436
R_{sigma}	0.044	Intensity transformed	0
Omitted reflections	0	Omitted by user (OMIT hkl)	0
Multiplicity	(9484, 4811, 1742, 403, 203, 110, 42, 23, 9, 9, 5)	Maximum multiplicity	17
Removed systematic absences	541	Filtered off (Shel/OMIT)	0

Table 1: Fractional Atomic Coordinates (×10^4) and Equivalent Isotropic Displacement Parameters (Å^2×10^3) for **4aqa.** U_{eq} is defined as 1/3 of the trace of the orthogonalised U_{ij}.

Atom	x	y	z	U_{eq}
F1	9806.0(19)	5433.9(8)	1044.0(11)	58.2(5)
F2	9270.8(17)	5408.7(8)	2303.8(12)	55.6(5)
F3	11261.7(16)	5370.3(8)	1978.8(11)	52.1(5)
O1	4026.0(17)	9209.0(9)	903.4(11)	35.7(4)
N1	10412.6(19)	8451.9(11)	1851.4(15)	33.0(5)
C1	3758(2)	8993.4(13)	1709.4(17)	32.3(6)
C2	4977(2)	8922.6(13)	2242.8(17)	31.1(6)
C3	5942(2)	8437.2(12)	1903.5(15)	27.6(5)
C4	7076(2)	8654.1(12)	1672.5(16)	30.2(6)
C5	8228(2)	8858.5(12)	1481.0(17)	31.7(6)
C6	9355(2)	8883.2(13)	2078.3(18)	34.3(6)
C7	5622(2)	7723.8(12)	1845.3(15)	27.2(5)
C8	4645(2)	7444.0(13)	2306.4(16)	30.0(6)
C9	4398(2)	6772.4(13)	2277.5(17)	31.6(6)
C10	5107(2)	6352.2(12)	1788.2(16)	31.0(6)
C11	4839(3)	5623.9(13)	1752.7(19)	44.3(7)
C12	6080(2)	6632.0(13)	1323.8(17)	32.5(6)
C13	6323(2)	7302.5(12)	1343.4(16)	28.4(5)
C14	10304(2)	7773.0(12)	1833.4(15)	27.9(5)
C15	9303(2)	7440.5(13)	2215.1(16)	29.4(6)
C16	9253(2)	6758.4(13)	2204.0(16)	30.1(6)
C17	10198(2)	6392.3(12)	1824.3(15)	31.4(6)
C18	11210(2)	6719.5(13)	1450.3(16)	33.0(6)
C19	11256(2)	7399.9(13)	1452.9(16)	32.0(6)
C20	10127(3)	5664.8(14)	1786.7(17)	37.9(6)
F4	12009(2)	9228.5(8)	4829.5(12)	67.9(6)
F5	12331(2)	9233.6(8)	3548.9(11)	58.8(5)

Atom	x	y	z	U_{eq}
F6	13924.0(18)	9247.4(8)	4382.7(14)	69.1(6)
O2	5160.7(17)	5466.8(9)	3987.1(13)	38.8(5)
N2	12832.2(18)	6175.2(10)	4373.7(14)	31.2(5)
C21	6197(3)	5826.2(14)	4405.9(18)	37.7(6)
C22	7302(2)	5900.2(14)	3827.5(18)	36.5(6)
C23	8352(2)	6342.6(12)	4189.4(16)	30.4(6)
C24	9469(2)	6076.6(12)	4392.8(16)	32.6(6)
C25	10591(2)	5820.6(13)	4611.5(18)	36.1(6)
C26	11742(2)	5772.6(14)	4075.4(18)	36.8(6)
C27	8120(2)	7061.8(12)	4270.2(15)	27.6(5)
C28	7033(2)	7359.8(13)	3915.5(17)	33.9(6)
C29	6861(2)	8035.6(13)	3962.2(17)	34.9(6)
C30	7753(2)	8445.1(13)	4356.7(16)	33.2(6)
C31	8829(2)	8142.4(13)	4721.1(17)	34.1(6)
C32	9007(2)	7468.4(13)	4685.8(16)	30.0(6)
C33	7565(3)	9180.1(13)	4379.5(19)	42.7(7)
C34	12766(2)	6866.9(12)	4329.2(15)	26.6(5)
C35	11935(2)	7203.9(13)	3792.4(16)	30.1(6)
C36	11948(2)	7886.9(13)	3760.3(16)	31.5(6)
C37	12794(2)	8247.0(12)	4257.3(16)	30.6(6)
C38	13634(2)	7911.0(13)	4786.7(16)	31.0(6)
C39	13627(2)	7238.2(12)	4821.2(15)	29.0(5)
C40	12757(3)	8979.1(14)	4253.2(17)	39.0(6)

Table 2: Anisotropic Displacement Parameters (×10[4]) for **4aqa.** The anisotropic displacement factor exponent takes the form: $-2\pi^2[h^2a^{*2} \times U_{11} + ... + 2hka^* \times b^* \times U_{12}]$

Atom	U_{11}	U_{22}	U_{33}	U_{23}	U_{13}	U_{12}
F1	84.9(14)	41.1(10)	47.7(11)	-8.2(8)	-15.7(10)	-9.6(9)
F2	61.7(11)	34.6(9)	71.4(13)	5.8(8)	21.3(10)	-1.5(8)
F3	49.0(10)	38.2(9)	68.9(12)	-5.2(8)	-7.4(8)	12.8(7)
O1	35.0(10)	34.3(10)	37.6(11)	-0.2(8)	-6.7(8)	0.0(8)
N1	20.7(10)	30.7(12)	47.5(14)	0.3(10)	0.1(10)	-0.9(9)
C1	22.0(12)	31.5(14)	43.3(16)	1.6(12)	-0.4(11)	2.0(10)
C2	24.8(12)	31.8(14)	36.5(15)	-0.9(11)	-3.3(11)	4.9(10)
C3	21.9(11)	29.7(13)	31.0(14)	1.8(10)	-5.6(10)	1.7(10)
C4	27.2(12)	25.6(12)	37.6(14)	-1.8(11)	-6.1(11)	5.5(10)
C5	24.7(12)	29.8(13)	40.7(16)	-0.1(11)	-1.6(11)	-0.2(10)
C6	24.8(12)	31.5(14)	46.4(17)	-4.7(12)	-2.2(11)	0.9(11)
C7	20.3(11)	29.8(13)	31.1(13)	1.5(10)	-7.3(10)	1.3(9)
C8	23.4(12)	33.4(14)	33.2(14)	1.3(11)	-0.7(10)	2.9(10)
C9	23.9(12)	34.9(14)	35.9(15)	6.0(11)	-2.7(11)	-4.1(10)
C10	28.8(12)	29.3(13)	34.7(14)	0.9(11)	-5.8(11)	-0.3(10)
C11	47.9(17)	35.3(15)	49.6(18)	2.1(13)	0.2(14)	-5.4(13)
C12	28.5(12)	32.2(14)	36.5(15)	-3.3(11)	-5.3(11)	5.3(11)
C13	19.7(11)	34.3(14)	31.0(14)	0.5(11)	-2.8(10)	-0.5(10)
C14	19.9(11)	33.1(14)	30.2(13)	1.4(10)	-7.2(10)	-0.4(9)
C15	20.7(11)	34.3(14)	33.0(14)	0.9(11)	-2.9(10)	3.7(10)
C16	22.8(12)	35.4(14)	31.9(14)	6.6(11)	-3.9(10)	-2.9(10)
C17	32.1(13)	31.7(14)	30.2(14)	-0.8(11)	-6.8(11)	2.2(10)
C18	27.2(12)	39.3(15)	32.3(14)	-5.9(11)	-0.1(11)	3.9(11)
C19	24.0(12)	39.9(15)	32.2(14)	0.8(11)	0.1(10)	-2.5(11)
C20	36.8(14)	41.4(15)	35.4(15)	-2.3(12)	-2.8(12)	1.2(12)
F4	106.2(16)	33.9(9)	65.4(13)	0.0(9)	43.5(12)	9.8(9)
F5	86.1(14)	40.3(10)	49.8(11)	9.6(8)	-5.5(10)	5.8(9)
F6	57.3(12)	40.6(10)	108.8(17)	6.6(10)	-13.9(11)	-15.9(9)
O2	20.5(9)	32.7(10)	63.2(14)	-4.6(9)	-1.0(8)	-0.2(7)
N2	20.0(10)	30.8(11)	42.5(13)	-5.2(10)	-8.8(9)	1.9(8)
C21	32.1(14)	37.0(15)	44.0(16)	-4.3(13)	-0.3(12)	-4.0(11)
C22	26.8(13)	38.1(15)	44.5(17)	-5.2(13)	-2.0(12)	-0.9(11)

Atom	U_{11}	U_{22}	U_{33}	U_{23}	U_{13}	U_{12}
C23	23.3(12)	34.8(14)	32.9(14)	0.8(11)	-3.1(10)	-1.5(10)
C24	27.7(12)	29.0(13)	41.3(15)	-5.0(11)	2.5(11)	-5.6(10)
C25	29.8(13)	31.6(14)	46.8(17)	-1.7(12)	-1.7(12)	1.6(11)
C26	26.6(13)	37.3(15)	46.0(17)	-3.5(13)	-7.2(12)	0.2(11)
C27	22.0(11)	32.7(13)	28.1(13)	3.8(10)	-1.6(10)	-2.0(10)
C28	26.2(12)	35.0(14)	40.0(15)	5.6(12)	-9.5(11)	-3.4(11)
C29	25.5(12)	40.2(15)	38.8(15)	8.0(12)	-5.2(11)	2.0(11)
C30	31.2(13)	32.9(14)	35.7(15)	1.3(11)	2.6(11)	0.7(10)
C31	26.7(13)	38.2(15)	37.1(15)	-7.4(12)	-3.2(11)	-1.1(11)
C32	20.3(11)	37.8(14)	31.6(14)	-2.6(11)	-3.2(10)	0.9(10)
C33	42.4(16)	34.9(15)	50.9(18)	1.5(13)	4.7(14)	1.7(12)
C34	17.6(10)	30.2(13)	31.9(13)	-3.5(10)	0.1(9)	1.3(9)
C35	21.0(11)	37.4(14)	31.8(14)	-3.1(11)	-3.9(10)	0.6(10)
C36	25.6(12)	36.2(14)	32.5(14)	1.3(11)	-1.6(10)	4.0(10)
C37	28.3(12)	32.7(14)	30.9(14)	-1.0(11)	3.4(10)	-0.2(10)
C38	24.3(12)	35.3(14)	33.2(14)	-4.0(11)	-2.4(10)	-2.9(10)
C39	21.8(11)	35.8(14)	29.4(13)	-2.9(11)	-3.2(10)	1.3(10)
C40	42.4(15)	38.1(15)	36.6(15)	3.5(12)	4.0(12)	-2.2(12)

Table 3: Bond Lengths in Å for **4aqa**.

Atom	Atom	Length/Å
F1	C20	1.338(3)
F2	C20	1.345(3)
F3	C20	1.352(3)
O1	C1	1.422(3)
N1	C6	1.459(3)
N1	C14	1.381(3)
C1	C2	1.529(3)
C2	C3	1.518(3)
C3	C4	1.320(3)
C3	C7	1.487(3)
C4	C5	1.311(3)
C5	C6	1.509(3)
C7	C8	1.398(3)
C7	C13	1.400(3)
C8	C9	1.386(4)
C9	C10	1.390(4)
C10	C11	1.503(4)
C10	C12	1.398(4)
C12	C13	1.383(4)
C14	C15	1.398(3)
C14	C19	1.401(3)
C15	C16	1.384(4)
C16	C17	1.388(4)
C17	C18	1.395(4)
C17	C20	1.478(4)
C18	C19	1.380(4)

Atom	Atom	Length/Å
F4	C40	1.334(3)
F5	C40	1.329(3)
F6	C40	1.341(3)
O2	C21	1.459(3)
N2	C26	1.471(3)
N2	C34	1.406(3)
C21	C22	1.511(4)
C22	C23	1.522(3)
C23	C24	1.315(3)
C23	C27	1.484(3)
C24	C25	1.319(4)
C25	C26	1.501(4)
C27	C28	1.396(3)
C27	C32	1.401(3)
C28	C29	1.384(4)
C29	C30	1.392(4)
C30	C31	1.397(4)
C30	C33	1.504(4)
C31	C32	1.380(4)
C34	C35	1.397(3)
C34	C39	1.408(3)
C35	C36	1.386(4)
C36	C37	1.391(3)
C37	C38	1.394(3)
C37	C40	1.485(4)
C38	C39	1.366(4)

Table 4: Bond Angles in ° for **4aqa**.

Atom	Atom	Atom	Angle/°
C14	N1	C6	122.8(2)
O1	C1	C2	112.4(2)
C3	C2	C1	113.4(2)
C4	C3	C2	119.3(2)
C4	C3	C7	120.4(2)
C7	C3	C2	120.4(2)

Atom	Atom	Atom	Angle/°
C5	C4	C3	176.9(3)
C4	C5	C6	123.7(3)
N1	C6	C5	113.2(2)
C8	C7	C3	121.6(2)
C8	C7	C13	117.5(2)
C13	C7	C3	120.9(2)

Atom	Atom	Atom	Angle/˚
C9	C8	C7	121.1(2)
C8	C9	C10	121.5(2)
C9	C10	C11	121.7(2)
C9	C10	C12	117.5(2)
C12	C10	C11	120.8(2)
C13	C12	C10	121.3(2)
C12	C13	C7	121.1(2)
N1	C14	C15	122.1(2)
N1	C14	C19	119.3(2)
C15	C14	C19	118.5(2)
C16	C15	C14	120.3(2)
C15	C16	C17	120.9(2)
C16	C17	C18	119.2(2)
C16	C17	C20	121.2(2)
C18	C17	C20	119.6(2)
C19	C18	C17	120.0(2)
C18	C19	C14	121.1(2)
F1	C20	F2	106.3(2)
F1	C20	F3	104.8(2)
F1	C20	C17	113.5(2)
F2	C20	F3	105.6(2)
F2	C20	C17	113.1(2)
F3	C20	C17	112.9(2)
C34	N2	C26	120.0(2)
O2	C21	C22	108.6(2)
C21	C22	C23	111.3(2)
C24	C23	C22	118.5(2)
C24	C23	C27	121.6(2)
C27	C23	C22	119.8(2)

Atom	Atom	Atom	Angle/˚
C23	C24	C25	178.6(3)
C24	C25	C26	125.3(3)
N2	C26	C25	112.8(2)
C28	C27	C23	121.3(2)
C28	C27	C32	117.7(2)
C32	C27	C23	121.0(2)
C29	C28	C27	120.6(2)
C28	C29	C30	122.1(2)
C29	C30	C31	117.0(2)
C29	C30	C33	121.2(2)
C31	C30	C33	121.9(2)
C32	C31	C30	121.6(2)
C31	C32	C27	121.0(2)
N2	C34	C39	118.3(2)
C35	C34	N2	123.3(2)
C35	C34	C39	118.3(2)
C36	C35	C34	120.4(2)
C35	C36	C37	120.6(2)
C36	C37	C38	119.0(2)
C36	C37	C40	120.4(2)
C38	C37	C40	120.5(2)
C39	C38	C37	120.7(2)
C38	C39	C34	120.9(2)
F4	C40	F6	105.8(2)
F4	C40	C37	113.0(2)
F5	C40	F4	106.1(2)
F5	C40	F6	105.2(2)
F5	C40	C37	113.6(2)
F6	C40	C37	112.4(2)

Table 5: Torsion Angles in ˚ for **4aqa**.

Atom	Atom	Atom	Atom	Angle/˚
O1	C1	C2	C3	-59.1(3)
N1	C14	C15	C16	-178.0(2)
N1	C14	C19	C18	177.4(2)
C1	C2	C3	C4	115.5(3)
C1	C2	C3	C7	-65.6(3)
C2	C3	C7	C8	-19.6(3)
C2	C3	C7	C13	162.5(2)
C3	C7	C8	C9	-176.7(2)
C3	C7	C13	C12	176.1(2)
C4	C3	C7	C8	159.3(2)
C4	C3	C7	C13	-18.5(3)
C4	C5	C6	N1	117.2(3)
C6	N1	C14	C15	-15.9(4)
C6	N1	C14	C19	166.9(2)
C7	C8	C9	C10	-0.3(4)
C8	C7	C13	C12	-1.8(3)
C8	C9	C10	C11	-179.8(2)
C8	C9	C10	C12	0.0(4)
C9	C10	C12	C13	-0.6(4)
C10	C12	C13	C7	1.6(4)
C11	C10	C12	C13	179.1(2)
C13	C7	C8	C9	1.2(4)
C14	N1	C6	C5	-65.4(3)
C14	C15	C16	C17	0.7(4)
C15	C14	C19	C18	0.2(4)
C15	C16	C17	C18	0.0(4)
C15	C16	C17	C20	-178.0(2)

Atom	Atom	Atom	Atom	Angle/˚
C16	C17	C18	C19	-0.7(4)
C16	C17	C20	F1	106.5(3)
C16	C17	C20	F2	-14.7(3)
C16	C17	C20	F3	-134.5(2)
C17	C18	C19	C14	0.6(4)
C18	C17	C20	F1	-71.5(3)
C18	C17	C20	F2	167.3(2)
C18	C17	C20	F3	47.5(3)
C19	C14	C15	C16	-0.8(3)
C20	C17	C18	C19	177.3(2)
O2	C21	C22	C23	173.1(2)
N2	C34	C35	C36	-177.8(2)
N2	C34	C39	C38	178.1(2)
C21	C22	C23	C24	111.9(3)
C21	C22	C23	C27	-70.8(3)
C22	C23	C27	C28	-9.4(4)
C22	C23	C27	C32	172.5(2)
C23	C27	C28	C29	-176.8(2)
C23	C27	C32	C31	176.2(2)
C24	C23	C27	C28	167.8(3)
C24	C23	C27	C32	-10.3(4)
C24	C25	C26	N2	115.0(3)
C26	N2	C34	C35	-21.6(4)
C26	N2	C34	C39	161.8(2)
C27	C28	C29	C30	0.2(4)
C28	C27	C32	C31	-1.9(4)
C28	C29	C30	C31	-1.2(4)
C28	C29	C30	C33	178.2(3)
C29	C30	C31	C32	0.6(4)
C30	C31	C32	C27	1.0(4)
C32	C27	C28	C29	1.3(4)
C33	C30	C31	C32	-178.8(3)
C34	N2	C26	C25	-70.5(3)
C34	C35	C36	C37	0.4(4)
C35	C34	C39	C38	1.2(4)
C35	C36	C37	C38	0.3(4)
C35	C36	C37	C40	-176.7(2)
C36	C37	C38	C39	-0.2(4)
C36	C37	C40	F4	93.2(3)
C36	C37	C40	F5	-27.8(4)
C36	C37	C40	F6	-147.1(3)
C37	C38	C39	C34	-0.5(4)
C38	C37	C40	F4	-83.8(3)
C38	C37	C40	F5	155.3(2)
C38	C37	C40	F6	36.0(4)
C39	C34	C35	C36	-1.2(4)
C40	C37	C38	C39	176.8(2)

Table 6: Hydrogen Fractional Atomic Coordinates (×10[4]) and Equivalent Isotropic Displacement Parameters (Å^2×10^3) for **4aqa.** U_{eq} is defined as 1/3 of the trace of the orthogonalised U_{ij}.

Atom	x	y	z	U_{eq}
H1	4450(40)	9610(20)	920(20)	93(14)
H1A	10950(30)	8623(15)	1490(20)	50(9)
H1B	3160(20)	9297(12)	1990(15)	27(7)
H1C	3320(30)	8542(14)	1637(17)	38(7)
H2A	5430(30)	9385(14)	2304(16)	36(7)
H2B	4680(30)	8778(14)	2821(19)	46(8)
H5	8450(30)	8995(14)	907(18)	46(8)
H6A	9020(30)	8772(13)	2646(18)	38(8)

Atom	x	y	z	U_{eq}
H6B	9730(30)	9340(15)	2084(17)	43(8)
H8	4160(30)	7701(13)	2660(17)	35(7)
H9	3730(30)	6604(13)	2586(17)	35(7)
H11A	5023.44	5426.9	2289.64	53
H11B	5387.93	5418.94	1344.08	53
H11C	3932.55	5551.08	1601.1	53
H11D	4539.17	5504.38	1200.24	53
H11E	4174.68	5512.34	2145.8	53
H11F	5630.07	5380.2	1888.78	53
H12	6590(30)	6331(14)	981(18)	44(8)
H13	6980(20)	7484(12)	1018(16)	27(7)
H15	8690(20)	7689(12)	2503(16)	28(7)
H16	8540(30)	6535(13)	2456(17)	39(8)
H18	11850(30)	6456(14)	1186(18)	44(8)
H19	11930(30)	7611(13)	1180(16)	33(7)
H2	4440(40)	5710(19)	4040(20)	82(13)
H2C	13160(30)	6044(15)	4906(19)	54(9)
H21A	6550(30)	5540(13)	4939(17)	39(8)
H21B	5890(20)	6280(13)	4619(15)	26(6)
H22A	7660(30)	5435(15)	3689(18)	46(8)
H22B	6790(30)	6022(17)	3270(20)	71(11)
H25	10770(30)	5643(17)	5210(20)	73(11)
H26A	12050(30)	5275(14)	4059(17)	42(8)
H26B	11390(30)	5903(14)	3499(18)	44(8)
H28	6390(30)	7087(15)	3630(19)	51(9)
H29	6110(30)	8225(14)	3719(18)	45(8)
H31	9450(30)	8400(15)	4982(19)	50(9)
H32	9740(30)	7285(12)	4970(16)	30(7)
H33A	7560.13	9329.54	4949.16	51
H33B	6743.39	9293.47	4109.59	51
H33C	8270.25	9396.43	4094.52	51
H33D	7489.04	9350.09	3819.69	51
H33E	8305.79	9386.15	4659.25	51
H33F	6778.93	9283.2	4674.33	51
H35	11350(30)	6973(12)	3451(16)	30(7)
H36	11410(30)	8106(16)	3380(20)	60(10)
H38	14220(30)	8125(14)	5119(18)	43(8)
H39	14160(20)	7005(12)	5187(16)	30(7)

Table 7: Hydrogen Bond information for **4aqa.**

D	H	A	d(D-H)/Å	d(H-A)/Å	d(D-A)/Å	D-H-A/deg
O1	H1	O2[1]	0.92(4)	1.79(4)	2.691(3)	165(4)
C6	H6B	F2[2]	1.00(3)	2.60(3)	3.545(3)	158(2)
O2	H2	N2[3]	0.90(4)	2.01(4)	2.895(3)	169(4)
N2	H2C	O1[4]	0.97(3)	1.92(3)	2.876(3)	173(3)

----

[1]1-x,1/2+y,1/2-z; [2]2-x,1/2+y,1/2-z; [3]-1+x,+y,+z; [4]1+x,3/2-y,1/2+z

Table 8: Atomic Occupancies for all atoms that are not fully occupied in **4aqa**.

Atom	Occupancy	Atom	Occupancy	Atom	Occupancy
H11A	0.36(3)	H11F	0.64(3)	H33E	0.65(3)
H11B	0.36(3)	H33A	0.35(3)	H33F	0.65(3)
H11C	0.36(3)	H33B	0.35(3)		
H11D	0.64(3)	H33C	0.35(3)		
H11E	0.64(3)	H33D	0.65(3)		

5,9-Bis(4-methylphenyl)-1-oxa-3,5-diaza-2,2-dioxo-2-thiacycloundeca-3,7,8-triene (14)

CCDC deposition Number 2519617

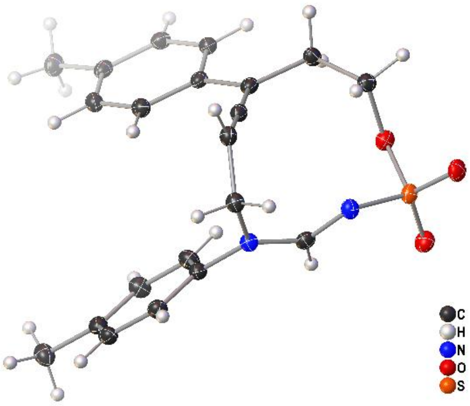

Crystals of compound **14** were collected upon crystallization *via* vapour diffusion with hexane/DCM at rt.

Experimental. Single colourless prism-shaped crystals of **14** were used as supplied. A suitable crystal with dimensions 0.18 × 0.04 × 0.03 mm was selected and mounted on a XtaLAB Synergy R, DW system, HyPix-Arc 150 diffractometer. The crystal was kept at a steady T = 100.00(10) K during data collection. The structure was solved with the ShelXT 2018/2 (Sheldrick, 2015) solution program using dual methods and by using Olex2 1.5 (Dolomanov et al., 2009) as the graphical interface. The model was refined with ShelXL 2019/3 (Sheldrick, 2015) using full matrix least squares minimisation on $|F|^2$.

Crystal Data. $C_{21}H_{22}N_2O_3S$, M_r = 382.46, monoclinic, $P2_1/c$ (No. 14), a = 11.1667(3) Å, b = 7.8757(3) Å, c = 21.2865(6) Å, β = 96.947(3)°, $\alpha = \gamma$ = 90°, V = 1858.31(11) Å^3, T = 100.00(10) K, Z = 4, Z' = 1, μ(Cu K$_\alpha$) = 1.750, 21688 reflections measured, 3763 unique (R_{int} = 0.0347) which were used in all calculations. The final wR_2 was 0.0896 (all data) and R_1 was 0.0349 (I≥2σ(I)).

**Compound**	**14**
Formula	$C_{21}H_{22}N_2O_3S$
$D_{calc.}$/ g cm^{-3}	1.367
μ/mm^{-1}	1.750
Formula Weight	382.46
Colour	colourless
Shape	prism-shaped
Size/mm	0.18×0.04×0.03
T/K	100.00(10)
Crystal System	monoclinic
Space Group	$P2_1/c$
a/Å	11.1667(3)
b/Å	7.8757(3)
c/Å	21.2865(6)
α/°	90
β/°	96.947(3)
γ/°	90
V/Å^3	1858.31(11)
Z	4
Z'	1
Wavelength/Å	1.54184
Radiation type	Cu K_α
Θ_{min}/°	3.988
Θ_{max}/°	75.728
Index range h	-9 ≥ h ≥ 13
Index range k	-9 ≥ k ≥ 9
Index range l	-26 ≥ l ≥ 26
Measured Refl's.	21688
Indep't Refl's	3763
Refl's I≥2σ(I)	3132
R_{int}	0.0347
Parameters	333
Restraints	0
Largest Peak/eÅ^3	0.275
Deepest Hole/eÅ^3	-0.413
GooF	1.053
R_1 (I≥2σ(I) / all)	0.0349 / 0.0452
wR_2 (I≥2σ(I) / all)	0.0850 / 0.0896
CCDC number	2519617

Structure Quality Indicators

**Reflections:**	d min (CuKα) 2Θ=151.5° 0.80	I/σ(I) 33.2	Rint m=6.19 3.47%	Full 135.4° 98% to 151.5° 99.9
**Refinement:**	Shift 0.000	Max Peak 0.3	Min Peak -0.4	GooF 1.053

A colourless prism-shaped crystal with dimensions 0.18 × 0.04 × 0.03 mm was mounted. Data were collected using a XtaLAB Synergy R, DW system, HyPix-Arc 150 diffractometer operating at T = 100.00(10) K.

Data were measured using ω scans with Cu K_α radiation. The diffraction pattern was indexed and the total number of runs and images was based on the strategy calculation from the program CrysAlis^{Pro} system (CCD 44.125a 64-bit (release 17-10-2025)). The maximum resolution achieved was Θ = 75.728°.

The unit cell was refined using CrysAlis^{Pro} on 4797 reflections, 22% of the observed reflections.

Data reduction, scaling and absorption corrections were performed using CrysAlis^{Pro}. The final completeness is 99.90 % out to 75.728° in Θ. A Gaussian absorption correction was performed using

CrysAlis^{Pro} 1.171.44.128a (Rigaku Oxford Diffraction, 2025) Numerical absorption correction based on Gaussian integration over a multifaceted crystal model. Empirical absorption correction using spherical harmonics as implemented in SCALE3 ABSPACK scaling algorithm. The absorption coefficient μ of this material is 1.750 mm^{-1} at this wavelength (λ = 1.54184Å) and the minimum and maximum transmissions are 0.674 and 1.000.

The structure was solved in the space group $P2_1/c$ (# 14) by ShelXT 2018/2 (Sheldrick, 2015) using dual methods. It was refined by full matrix least squares minimisation on $|F|^2$ using version 2019/3 of ShelXL 2019/3 (Sheldrick, 2015). All non-hydrogen atoms were refined anisotropically.

All hydrogen atoms were freely refined.

There is a single formula unit in the asymmetric unit, which is represented by the reported sum formula. In other words: Z is 4 and Z' is 1. The moiety formula is C21 H22 N2 O3 S.

Data Plots: Diffraction Data

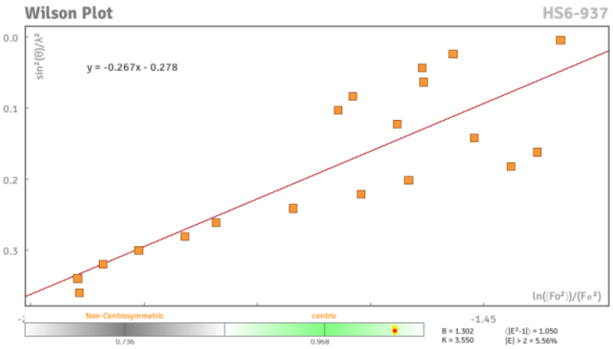

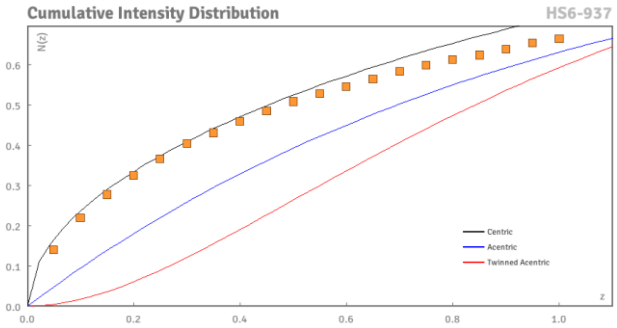

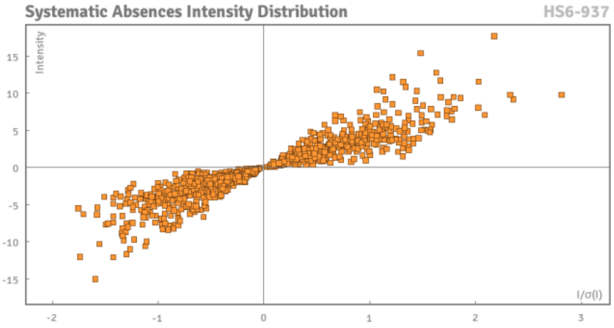

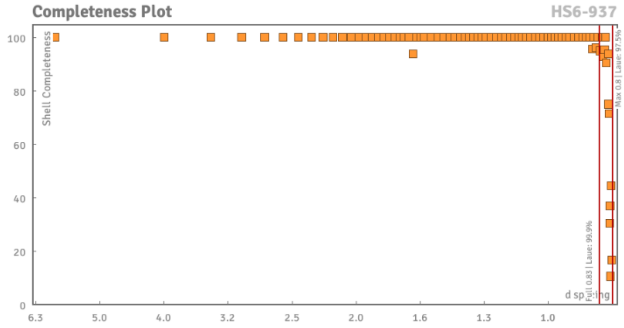

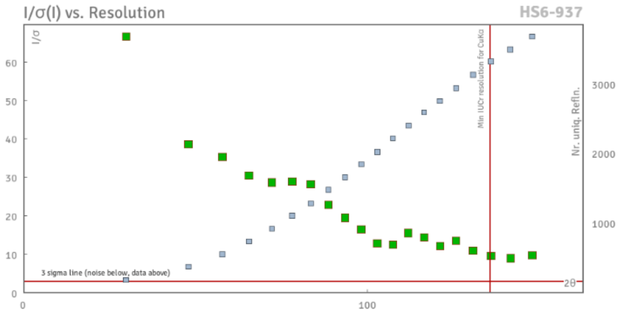

Data Plots: Refinement and Data

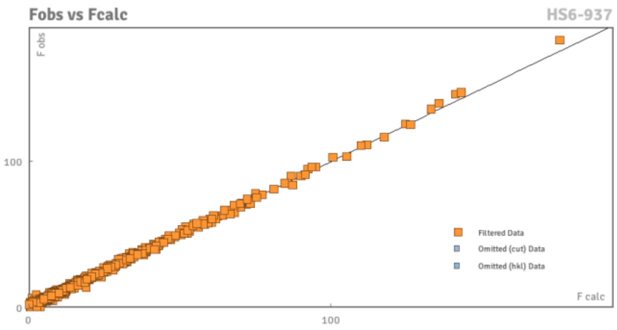

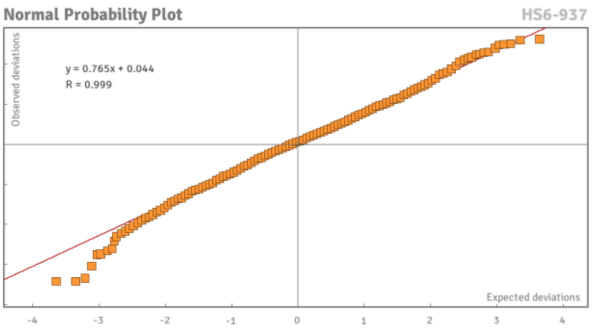

Reflection Statistics

Total reflections (after filtering)	23293	Unique reflections	3763
Completeness	0.975	Mean I/σ	22.07
hkl_{max} collected	(13, 9, 26)	hkl_{min} collected	(-9, -9, -26)
hkl_{max} used	(13, 9, 26)	hkl_{min} used	(-13, 0, 0)
Lim d_{max} collected	100.0	Lim d_{min} collected	0.77
d_{max} used	21.13	d_{min} used	0.8
Friedel pairs	1909	Friedel pairs merged	1
Inconsistent equivalents	4	R_{int}	0.0347
R_{sigma}	0.0301	Intensity transformed	0
Omitted reflections	0	Omitted by user (OMIT hkl)	0
Multiplicity	(3778, 1873, 1054, 564, 356, 299, 174, 157, 95, 80, 53, 41, 36, 24, 16, 13, 9, 5, 2, 2)	Maximum multiplicity	32
Removed systematic absences	1605	Filtered off (Shel/OMIT)	0

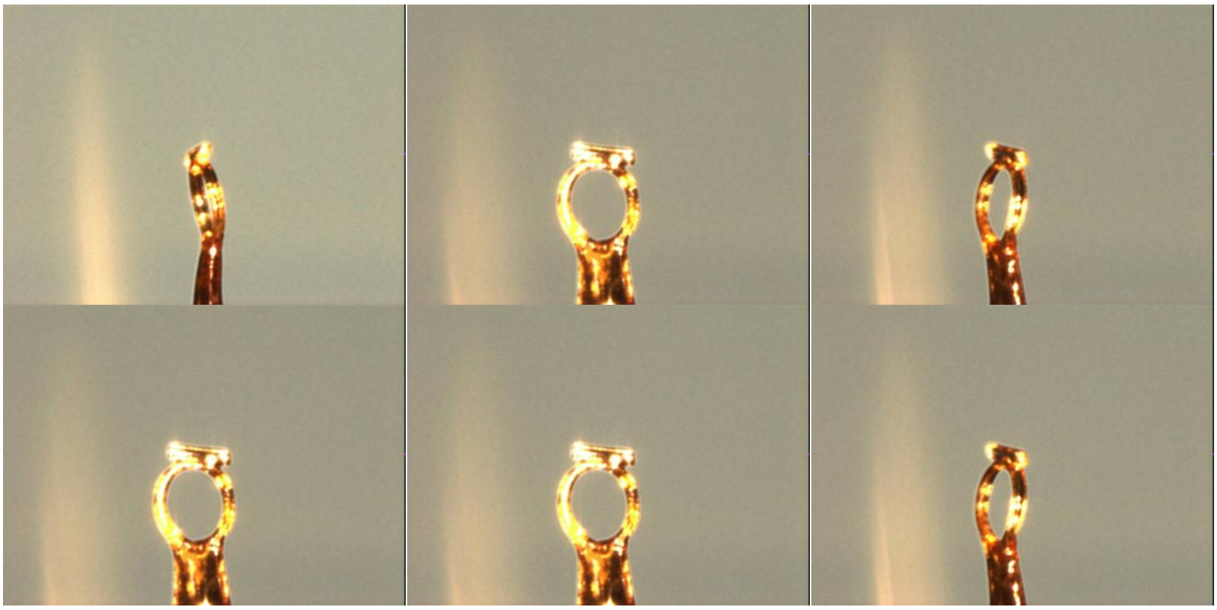

Table 9: Fractional Atomic Coordinates (×10[4]) and Equivalent Isotropic Displacement Parameters (Å[2]×10[3]) for **14**. U_{eq} is defined as 1/3 of the trace of the orthogonalised U_{ij}.

Atom	x	y	z	U_{eq}
S1	2525.3(3)	6420.4(5)	4827.3(2)	17.01(12)
O1	1547.6(9)	6431.7(15)	4224.7(5)	18.3(2)
O2	1927.9(10)	6306.6(16)	5381.5(5)	21.7(3)
O3	3247.3(10)	7889.7(15)	4739.7(5)	21.2(3)
N1	3216.6(11)	4652.6(18)	4755.2(6)	17.5(3)
N2	4363.8(11)	3169.2(17)	4121.0(6)	16.5(3)

Atom	x	y	z	U_{eq}
C1	3979.2(13)	4627(2)	4331.9(7)	17.0(3)
C2	5096.7(13)	3123(2)	3608.0(7)	17.1(3)
C3	6093.8(13)	2060(2)	3646.6(7)	18.9(3)
C4	6778.7(14)	2000(2)	3145.5(8)	19.5(3)
C5	6493.7(13)	2989(2)	2605.5(7)	19.0(3)
C6	5491.1(14)	4051(2)	2578.2(8)	21.4(4)
C7	4787.0(14)	4107(2)	3073.3(8)	20.4(3)
C8	7249.2(16)	2911(3)	2067.8(8)	24.3(4)
C9	3811.0(14)	1586(2)	4308.9(7)	17.3(3)
C10	2549.9(14)	1342(2)	3968.2(7)	17.4(3)
C11	1935.9(13)	2432(2)	3596.2(7)	16.9(3)
C12	1251.2(13)	3469(2)	3222.6(7)	16.2(3)
C13	342.7(14)	4628(2)	3489.5(7)	18.0(3)
C14	628.4(14)	5084(2)	4181.0(7)	18.2(3)
C15	1287.7(13)	3470(2)	2522.2(7)	15.9(3)
C16	2138.4(14)	2492(2)	2248.7(7)	18.4(3)
C17	2175.4(14)	2490(2)	1600.0(7)	19.9(3)
C18	1379.9(14)	3478(2)	1196.5(7)	18.6(3)
C19	526.0(13)	4433(2)	1466.5(7)	18.6(3)
C20	477.5(13)	4437(2)	2116.4(7)	17.6(3)
C21	1445.3(17)	3552(3)	493.1(8)	24.2(4)

Table 10: Anisotropic Displacement Parameters (×10[4]) for **14**. The anisotropic displacement factor exponent takes the form: $-2\pi^2[h^2a^{*2} \times U_{11} + ... +2hka^* \times b^* \times U_{12}]$

Atom	U_{11}	U_{22}	U_{33}	U_{23}	U_{13}	U_{12}
S1	21.1(2)	16.3(2)	13.83(18)	-1.07(14)	2.71(13)	0.46(15)
O1	21.8(5)	16.8(6)	16.0(5)	1.0(4)	1.2(4)	0.0(5)
O2	26.4(6)	24.4(7)	15.3(5)	-2.6(5)	6.1(4)	-0.6(5)
O3	26.2(6)	15.2(6)	22.6(6)	-0.3(4)	4.3(4)	-2.2(5)
N1	20.3(6)	17.3(7)	15.0(6)	1.0(5)	2.5(5)	1.9(5)
N2	17.8(6)	15.0(7)	16.9(6)	1.2(5)	2.7(5)	0.7(5)
C1	17.3(7)	17.4(8)	15.6(7)	0.3(6)	-1.3(5)	-1.0(6)
C2	15.9(7)	18.1(8)	17.3(7)	-1.2(6)	1.9(6)	-1.2(6)
C3	18.0(7)	19.9(9)	18.4(7)	0.8(6)	0.3(6)	1.0(6)
C4	16.2(7)	18.3(8)	23.5(8)	-1.5(6)	0.9(6)	1.5(7)
C5	18.3(7)	17.6(8)	21.5(8)	-2.1(6)	4.0(6)	-3.2(6)
C6	22.0(8)	21.9(9)	20.4(8)	5.0(7)	2.9(6)	1.0(7)
C7	18.3(7)	19.8(9)	23.4(8)	3.3(6)	3.2(6)	3.0(7)
C8	25.7(9)	20.6(9)	28.1(9)	0.8(7)	9.6(7)	-0.3(8)
C9	20.1(7)	14.2(8)	17.9(7)	2.7(6)	3.2(6)	1.3(6)
C10	20.8(7)	15.2(8)	16.7(7)	-0.3(6)	4.3(6)	0.2(7)
C11	17.6(7)	17.4(8)	16.1(7)	-2.2(6)	4.1(5)	-2.3(6)
C12	16.1(7)	15.3(8)	17.0(7)	-1.3(6)	1.6(5)	-2.2(6)
C13	17.1(7)	21.4(9)	15.3(7)	-0.6(6)	1.6(6)	2.2(7)
C14	17.6(7)	21.2(9)	16.1(7)	-0.2(6)	3.6(6)	-0.7(7)
C15	16.7(7)	15.3(8)	15.8(7)	-1.9(6)	2.4(5)	-3.5(6)
C16	19.7(7)	16.4(8)	19.1(7)	0.1(6)	2.6(6)	0.7(7)
C17	19.8(7)	18.9(9)	21.7(8)	-3.0(6)	5.6(6)	-0.1(7)
C18	20.5(7)	19.1(8)	16.4(7)	-1.6(6)	3.5(6)	-6.0(7)
C19	17.3(7)	20.8(9)	17.2(7)	-0.6(6)	0.5(6)	-2.0(7)
C20	16.0(7)	18.4(8)	18.5(7)	-2.6(6)	2.4(6)	-0.9(6)
C21	29.4(9)	25.7(10)	18.5(8)	-2.5(7)	6.9(7)	-2.6(8)

Table 11: Bond Lengths in Å for **14**.

Atom	Atom	Length/Å
S1	O1	1.5806(10)
S1	O2	1.4267(11)
S1	O3	1.4354(12)
S1	N1	1.6083(14)
O1	C14	1.4714(19)
N1	C1	1.3127(19)
N2	C1	1.323(2)
N2	C2	1.4422(18)
N2	C9	1.468(2)
C2	C3	1.388(2)
C2	C7	1.386(2)
C3	C4	1.386(2)
C4	C5	1.393(2)
C5	C6	1.393(2)
C5	C8	1.503(2)
C6	C7	1.390(2)
C9	C10	1.516(2)
C10	C11	1.305(2)
C11	C12	1.318(2)
C12	C13	1.525(2)
C12	C15	1.496(2)
C13	C14	1.511(2)
C15	C16	1.403(2)
C15	C20	1.398(2)
C16	C17	1.387(2)
C17	C18	1.395(2)
C18	C19	1.392(2)
C18	C21	1.509(2)
C19	C20	1.391(2)

Table 12: Bond Angles in ° for **14**.

Atom	Atom	Atom	Angle/°
O1	S1	N1	102.70(6)
O2	S1	O1	108.98(6)
O2	S1	O3	118.96(7)
O2	S1	N1	107.60(7)
O3	S1	O1	103.52(6)
O3	S1	N1	113.70(7)
C14	O1	S1	117.08(9)
C1	N1	S1	115.94(12)
C1	N2	C2	121.05(14)
C1	N2	C9	118.69(13)
C2	N2	C9	118.74(13)
N1	C1	N2	120.68(15)
C3	C2	N2	119.67(14)
C7	C2	N2	119.99(14)
C7	C2	C3	120.32(14)
C4	C3	C2	119.23(15)
C3	C4	C5	121.57(15)
C4	C5	C8	120.81(15)
C6	C5	C4	118.19(14)
C6	C5	C8	121.00(15)
C7	C6	C5	120.86(15)
C2	C7	C6	119.82(15)
N2	C9	C10	111.98(13)
C11	C10	C9	126.59(15)
C10	C11	C12	176.16(16)
C11	C12	C13	120.65(14)
C11	C12	C15	121.16(14)
C15	C12	C13	118.08(13)
C14	C13	C12	115.70(13)
O1	C14	C13	107.25(12)
C16	C15	C12	120.99(14)
C20	C15	C12	121.46(14)
C20	C15	C16	117.55(14)
C17	C16	C15	121.15(15)
C16	C17	C18	121.26(15)
C17	C18	C21	121.92(15)
C19	C18	C17	117.64(14)
C19	C18	C21	120.43(15)
C20	C19	C18	121.52(15)
C19	C20	C15	120.87(15)

Table 13: Torsion Angles in ° for **14**.

Atom	Atom	Atom	Atom	Angle/°
S1	O1	C14	C13	-145.15(11)
S1	N1	C1	N2	-163.00(11)
O1	S1	N1	C1	76.05(12)
O2	S1	O1	C14	-50.68(12)
O2	S1	N1	C1	-169.03(11)
O3	S1	O1	C14	-178.22(10)
O3	S1	N1	C1	-35.08(13)
N1	S1	O1	C14	63.23(11)
N2	C2	C3	C4	178.73(14)
N2	C2	C7	C6	-179.59(15)
N2	C9	C10	C11	-6.4(2)
C1	N2	C2	C3	136.43(16)
C1	N2	C2	C7	-45.1(2)

Atom	Atom	Atom	Atom	Angle/°
C1	N2	C9	C10	73.72(17)
C2	N2	C1	N1	173.23(13)
C2	N2	C9	C10	-92.39(16)
C2	C3	C4	C5	0.4(2)
C3	C2	C7	C6	-1.2(3)
C3	C4	C5	C6	-0.1(2)
C3	C4	C5	C8	179.81(16)
C4	C5	C6	C7	-0.7(2)
C5	C6	C7	C2	1.4(3)
C7	C2	C3	C4	0.3(2)
C8	C5	C6	C7	179.31(16)
C9	N2	C1	N1	7.5(2)
C9	N2	C2	C3	-57.80(19)
C9	N2	C2	C7	120.63(17)
C11	C12	C13	C14	25.4(2)
C11	C12	C15	C16	-6.6(2)
C11	C12	C15	C20	173.26(15)
C12	C13	C14	O1	79.02(17)
C12	C15	C16	C17	-179.97(15)
C12	C15	C20	C19	179.63(15)
C13	C12	C15	C16	177.08(14)
C13	C12	C15	C20	-3.1(2)
C15	C12	C13	C14	-158.21(14)
C15	C16	C17	C18	0.9(2)
C16	C15	C20	C19	-0.5(2)
C16	C17	C18	C19	-1.6(2)
C16	C17	C18	C21	177.14(16)
C17	C18	C19	C20	1.3(2)
C18	C19	C20	C15	-0.2(2)
C20	C15	C16	C17	0.2(2)
C21	C18	C19	C20	-177.50(16)

Table 14: Hydrogen Fractional Atomic Coordinates (×10[4]) and Equivalent Isotropic Displacement Parameters (Å^2×10^3) for **14**. U_{eq} is defined as 1/3 of the trace of the orthogonalised U_{ij}.

Atom	x	y	z	U_{eq}
H1	4305(16)	5640(30)	4162(9)	24(5)
H3	6305(15)	1330(20)	4012(9)	19(5)
H4	7459(17)	1250(30)	3185(9)	25(5)
H6	5243(15)	4730(30)	2209(8)	21(5)
H7	4077(16)	4820(30)	3034(8)	22(5)
H8A	7640(20)	1820(40)	2048(11)	50(7)
H8B	6742(19)	3120(30)	1651(10)	40(6)
H8C	7880(20)	3820(30)	2112(11)	45(6)
H9A	3776(15)	1610(20)	4768(9)	21(5)
H9B	4309(16)	670(30)	4209(9)	22(5)
H10	2207(16)	250(30)	4051(9)	25(5)
H13A	266(15)	5690(30)	3254(9)	21(5)
H13B	-463(16)	4070(30)	3425(9)	22(5)
H14A	-85(16)	5550(20)	4350(8)	20(5)
H14B	939(16)	4100(30)	4436(9)	23(5)
H16	2712(16)	1800(30)	2513(9)	26(5)
H17	2783(16)	1780(30)	1429(9)	23(5)
H19	-26(16)	5190(30)	1192(9)	25(5)
H20	-136(15)	5130(20)	2277(8)	18(4)
H21A	1932(17)	2650(30)	363(10)	32(5)
H21B	658(19)	3480(30)	254(10)	36(6)
H21C	1782(18)	4630(30)	380(10)	40(6)

H. References

[1] P. Yu, B. Morandi, "Nickel-Catalyzed Cyanation of Aryl Chlorides and Triflates Using Butyronitrile: Merging Retro-hydrocyanation with Cross-Coupling" *Angew. Chem. Int. Ed.* **2017**, *56*, 15693–15697.

[2] T. Taeufer, J. Pospech, "Palladium-Catalyzed Synthesis of N,N-Dimethylanilines via Buchwald–Hartwig Amination of (Hetero)aryl Triflates" *J. Org. Chem.* **2020**, *85*, 7097–7111.

[3] S. D. Schimler, R. D. J. Froese, D. C. Bland, M. S. Sanford, "Reactions of Arylsulfonate Electrophiles with NMe4F: Mechanistic Insight, Reactivity, and Scope" *J. Org. Chem.* **2018**, *83*, 11178–11190.

[4] C. A. Quesnelle, V. Snieckus, "Directed ortho Metalation (DoM)-Linked Corriu–Kumada, Negishi, and Suzuki–Miyaura Cross-Coupling Protocols: A Comparative Study" *Synthesis* **2018**, *50*, 4395–4412.

[5] N. Machinaga, T. Yoshino, J. Chiba, J. Watanabe, T. Suzuki, Y. Kimura, *Heteroarylamide Lower Carboxylic Acid Derivative*, **2009**, EP2017263A1.

[6] T. Taeufer, J. Pospech, "Palladium-Catalyzed Synthesis of N,N-Dimethylanilines via Buchwald–Hartwig Amination of (Hetero)aryl Triflates" *J. Org. Chem.* **2020**, *85*, 7097–7111.

[7] T. Bunlaksananusorn, P. Knochel, "t-BuOK-Mediated Hydrophosphination of Functionalized Alkenes: A Novel Synthesis of Chiral P,N- and P,P-Ligands" *J. Org. Chem.* **2004**, *69*, 4595–4601.

[8] S. Iimura, W. Wu, "Palladium-catalyzed borylation of l-tyrosine triflate derivative with pinacolborane: practical route to 4-borono-l-phenylalanine (l-BPA) derivatives" *Tetrahedron Lett.* **2010**, *51*, 1353–1355.

[9] K. Muto, J. Yamaguchi, K. Itami, "Nickel-Catalyzed C–H/C–O Coupling of Azoles with Phenol Derivatives" *J. Am. Chem. Soc.* **2012**, *134*, 169–172.

[10] W. M. Seganish, P. DeShong, "Preparation and Palladium-Catalyzed Cross-Coupling of Aryl Triethylammonium Bis(catechol) Silicates with Aryl Triflates" *J. Org. Chem.* **2004**, *69*, 1137–1143.

[11] T. Kawasuji, T. Yoshinaga, A. Sato, M. Yodo, T. Fujiwara, R. Kiyama, "A platform for designing HIV integrase inhibitors. Part 1: 2-Hydroxy-3-heteroaryl acrylic acid derivatives as novel HIV integrase inhibitor and modeling of hydrophilic and hydrophobic pharmacophores" *Bioorg. Med. Chem.* **2006**, *14*, 8430–8445.

[12] H. Yu, B. Yu, H. Zhang, H. Huang, "Palladium-Catalyzed Aminomethylation and Cyclization of Enynol to O-Heterocycle Confined 1,3-Dienes" *Org. Lett.* **2021**, *23*, 3891–3896.

[13] N. J. Adamson, H. Jeddi, S. J. Malcolmson, "Preparation of Chiral Allenes through Pd-Catalyzed Intermolecular Hydroamination of Conjugated Enynes: Enantioselective Synthesis Enabled by Catalyst Design" *J. Am. Chem. Soc.* **2019**, *141*, 8574–8583.

[14] H. Solé-Àvila, M. Puriņš, L. Eichenberger, J. Waser, "Enamine Synthesis via Regiocontrolled 6-endo-dig and 5-exo-dig Tethered Carboamination of Propargylic Alcohols" *Angew. Chem. Int. Ed.* **2024**, *63*, e202411383.

[15] J. J. Pflueger, L. C. Morrill, J. N. deGruyter, M. A. Perea, R. Sarpong, "Magnesiate Addition/Ring-Expansion Strategy To Access the 6–7–6 Tricyclic Core of Hetisine-Type C20-Diterpenoid Alkaloids" *Org. Lett.* **2017**, *19*, 4632–4635.

[16] K. Guo, Q. Zeng, A. Villar-Yanez, C. Bo, A. W. Kleij, "Ni-Catalyzed Decarboxylative Silylation of Alkynyl Carbonates: Access to Chiral Allenes via Enantiospecific Conversions" *Org. Lett.* **2022**, *24*, 637–641.

[17] J. Waser, J. C. González-Gómez, H. Nambu, P. Huber, E. M. Carreira, "Cobalt-Catalyzed Hydrohydrazination of Dienes and Enynes: Access to Allylic and Propargylic Hydrazides" *Org. Lett.* **2005**, *7*, 4249–4252.

[18] F. Pünner, G. Hilt, "Regioselective solvent-dependent benzannulation of conjugated enynes" *Chem. Commun.* **2012**, *48*, 3617–3619.

[19] C. Deng, L. Jiang, J. Yao, Q. Liang, L. Miao, C. Li, M. Miao, H. Zhou, "Rhodium(III)-Catalyzed Sequential Cyclization of N-Boc Hydrazones with Propargylic Monofluoroalkynes via C–H Activation/C–F Cleavage for the Synthesis of Spiro[cyclobutane-1,9′-indeno[1,2-a]indenes]" *J. Org. Chem.* **2022**, *87*, 6105–6114.

[20] T. Kawasaki, Y. Yamamoto, "Synthesis of 6H-Dibenzo[b,d]pyran-6-ones from Aryl 3-Bromopropenoates via a Sequential One-Pot Procedure Using the Sonogashira Coupling−Benzannulation Reaction" *J. Org. Chem.* **2002**, *67*, 5138–5141.

[21] N. Kobayashi, K. Kaneko, S. Amemiya, K. Noguchi, M. Yamanaka, A. Saito, "Alkyne aza-Prins cyclization of N-(hexa-3,5-diynyl)tosylamides with aldehydes using triflic acid and a binuclear aluminum complex" *Chem. Commun.* **2019**, *55*, 8619–8622.

[22] P. J. Smith, Y. Jiang, Z. Tong, H. D. Pickford, K. E. Christensen, J. Nugent, E. A. Anderson, "Synthesis of Polysubstituted Fused Pyrroles by Gold-Catalyzed Cycloisomerization/1,2-Sulfonyl Migration of Yndiamides" *Org. Lett.* **2021**, *23*, 6547–6552.

[23] Y. Bai, Z. Lin, Z. Ye, D. Dong, J. Wang, L. Chen, F. Xie, Y. Li, P. H. Dixneuf, M. Zhang, "Ruthenium-Catalyzed Regioselective Hydrohalogenation of Alkynes Mediated by Trimethylsilyl Triflate" *Org. Lett.* **2022**, *24*, 7988–7992.

[24] C. Yang, Z.-L. Liu, D.-T. Dai, Q. Li, W.-W. Ma, M. Zhao, Y.-H. Xu, "Catalytic Asymmetric Conjugate Protosilylation and Protoborylation of 2-Trifluoromethyl Enynes for Synthesis of Functionalized Allenes" *Org. Lett.* **2020**, *22*, 1360–1367.

[25] H. Yu, B. Yu, H. Zhang, H. Huang, "Palladium-Catalyzed Aminomethylation and Cyclization of Enynol to O-Heterocycle Confined 1,3-Dienes" *Org. Lett.* **2021**, *23*, 3891–3896.

[26] H. Cheng, T.-L. Lam, Y. Liu, Z. Tang, C.-M. Che, "Photoinduced Hydroarylation and Cyclization of Alkenes with Luminescent Platinum(II) Complexes" *Angew. Chem. Int. Ed.* **2021**, *60*, 1383–1389.

[27] D. Jaspers, S. Doye, "Potassium Hydroxide Catalyzed Addition of Arylamines to Styrenes" *Synlett* **2011**, *2011*, 1444–1448.

[28] N. Sakai, H. Hori, Y. Ogiwara, "Copper(II)-Catalyzed [4+1] Annulation of Propargylamines with N,O-Acetals: Entry to the Synthesis of Polysubstituted Pyrrole Derivatives" *Eur. J. Org. Chem.* **2015**, *2015*, 1905–1909.

[29] D. Zhu, Z. Wu, B. Luo, Y. Du, P. Liu, Y. Chen, Y. Hu, P. Huang, S. Wen, "Heterocyclic Iodoniums for the Assembly of Oxygen-Bridged Polycyclic Heteroarenes with Water as the Oxygen Source" *Org. Lett.* **2018**, *20*, 4815–4818.

[30] Z. Xu, Q. Wang, J. Zhu, "Total Syntheses of (−)-Mersicarpine, (−)-Scholarisine G, (+)-Melodinine E, (−)-Leuconoxine, (−)-Leuconolam, (−)-Leuconodine A, (+)-Leuconodine F, and (−)-Leuconodine C: Self-Induced Diastereomeric Anisochronism (SIDA) Phenomenon for Scholarisine G and Leuconodines A and C" *J. Am. Chem. Soc.* **2015**, *137*, 6712–6724.

[31] E. G. Burke, J. M. Schomaker, "Oxidative Allene Amination for the Synthesis of Azetidin-3-ones" *Angew. Chem. Int. Ed.* **2015**, *54*, 12097–12101.

I. NMR Spectra

I.1. Spectra of starting materials

Hept-6-en-4-yn-2-ol (1j)

^{1}H NMR (400 MHz, $CDCl_3$)

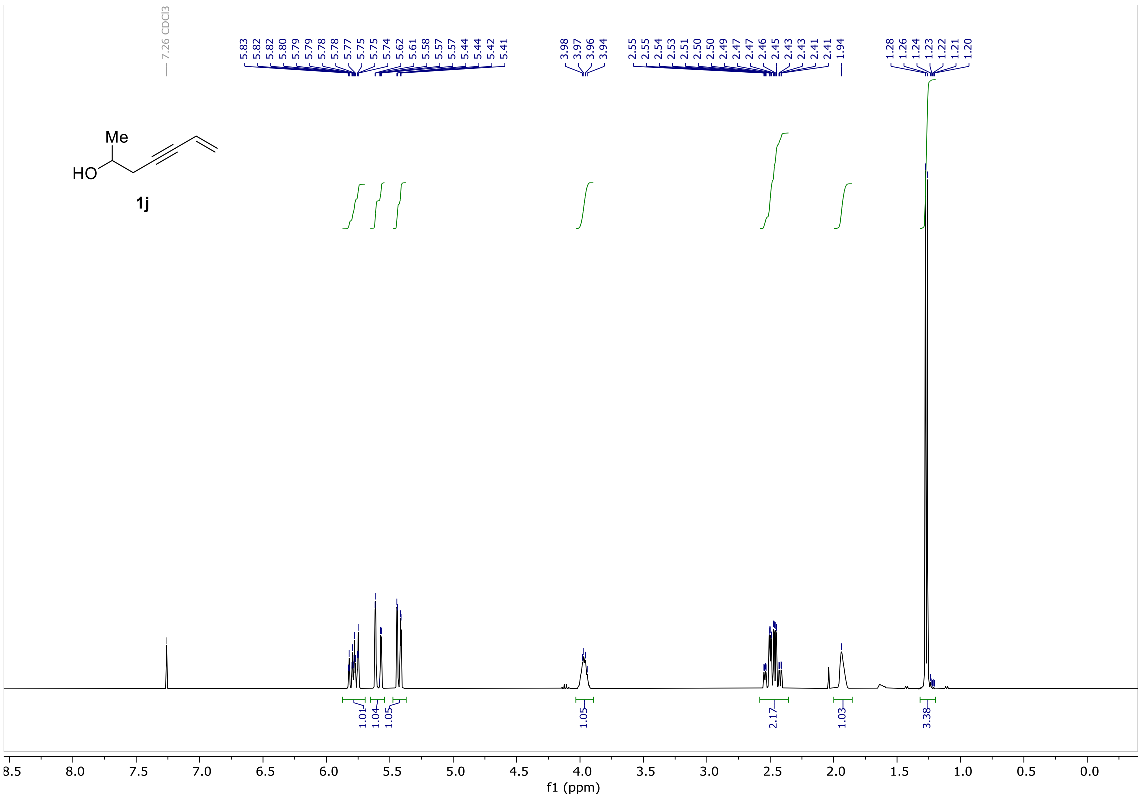

^{13}C NMR (101 MHz, $CDCl_3$)

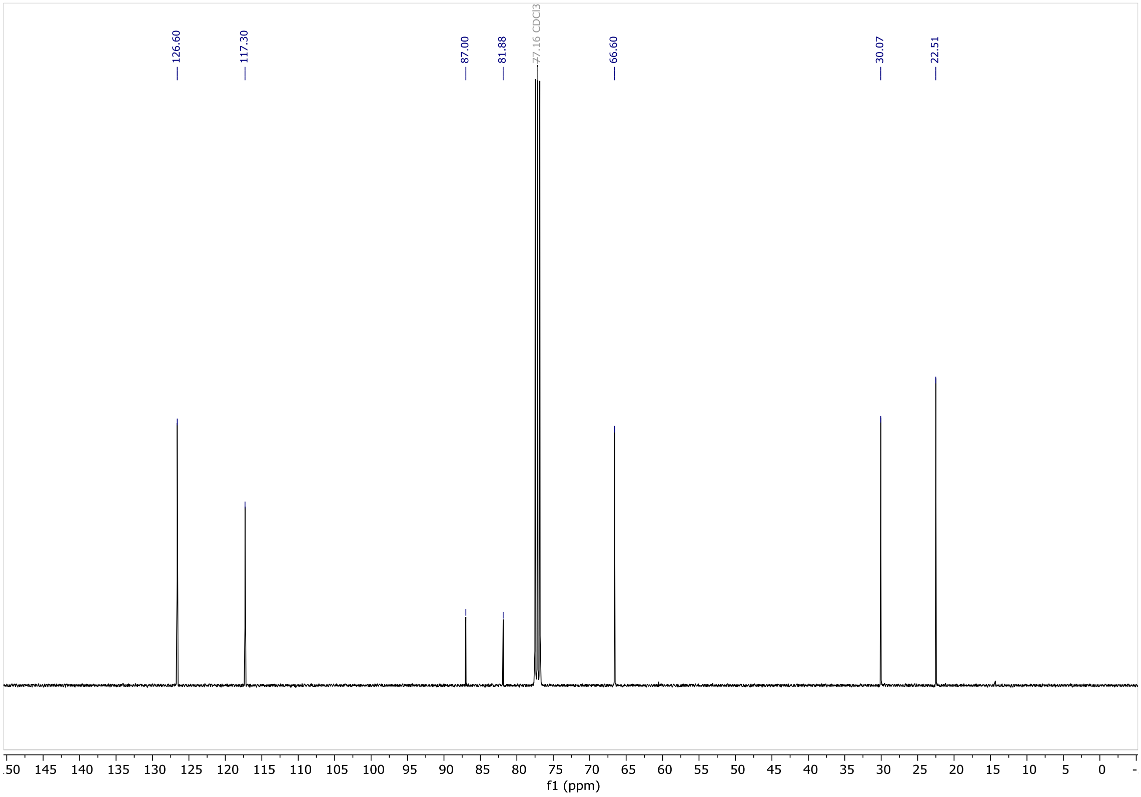

***N*-(Hex-5-en-3-yn-1-yl)-4-methylbenzenesulfonamide (1i)**

^{1}H NMR (400 MHz, $CDCl_3$)

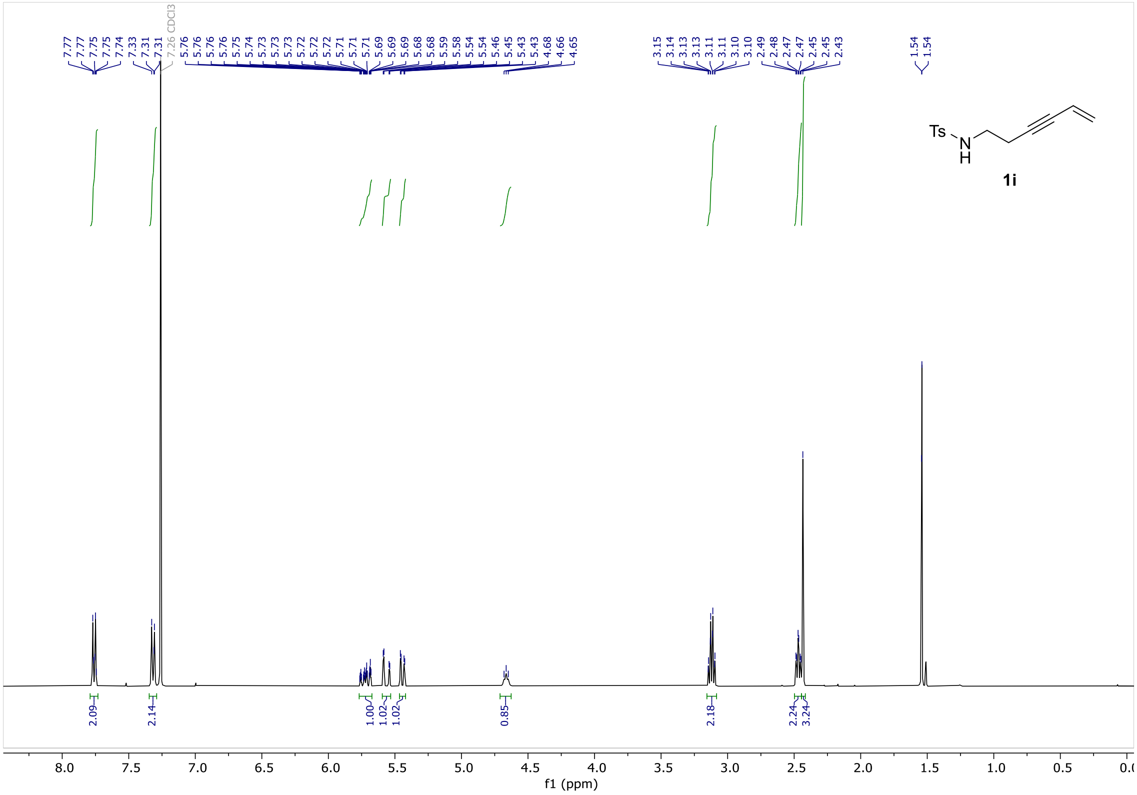

^{13}C NMR (101 MHz, $CDCl_3$)

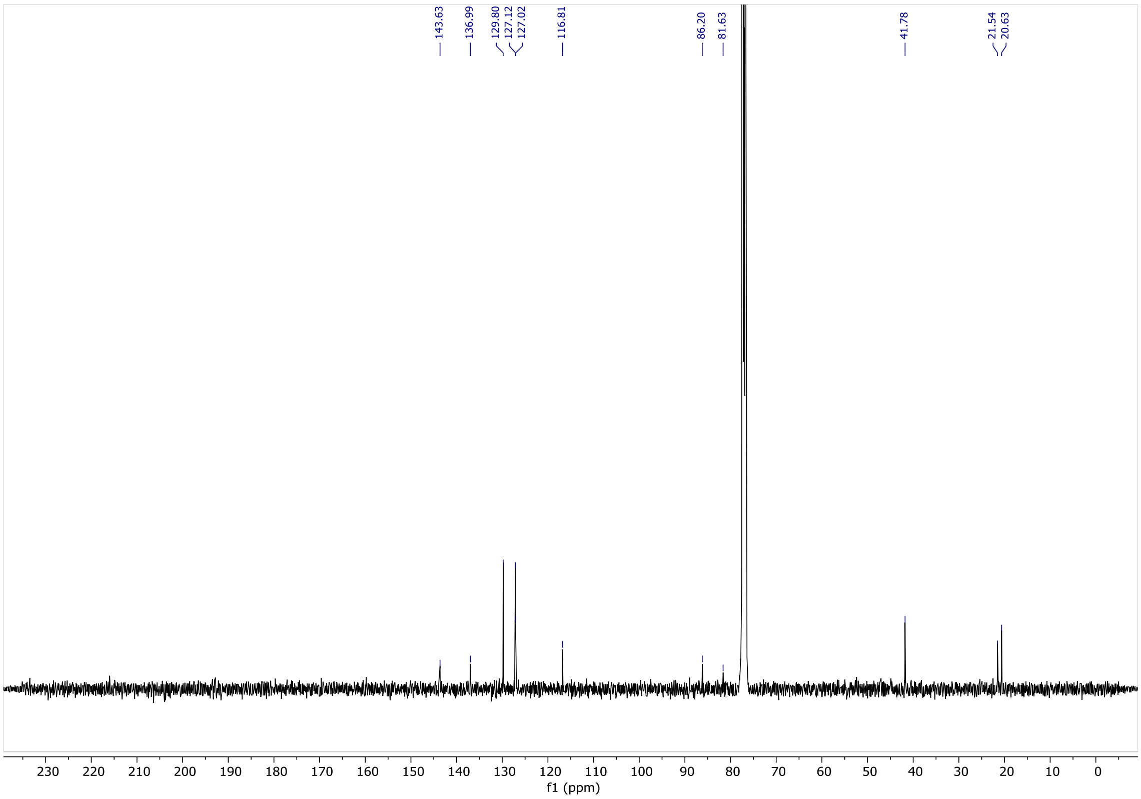

1,1,1-Trifluoro-*N*-(hex-5-en-3-yn-1-yl)methanesulfonamide (S24)

^{1}H NMR (400 MHz, $CDCl_3$)

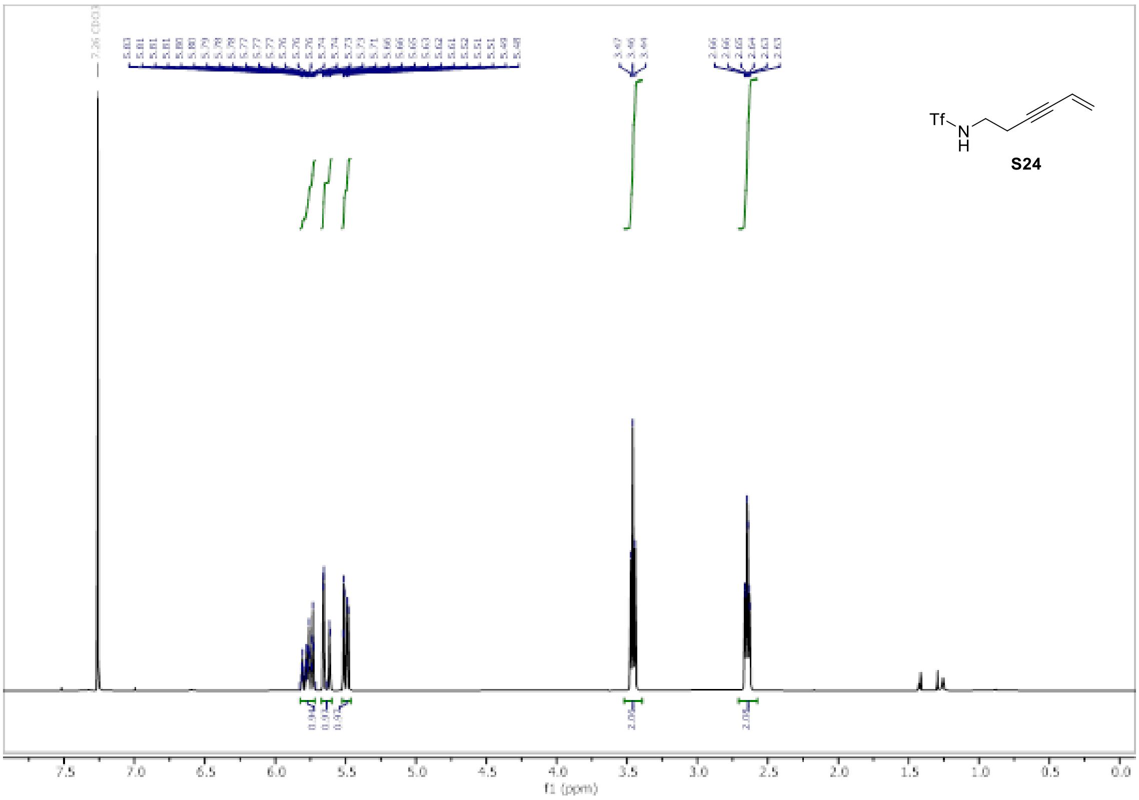

^{13}C NMR (101 MHz, $CDCl_3$)

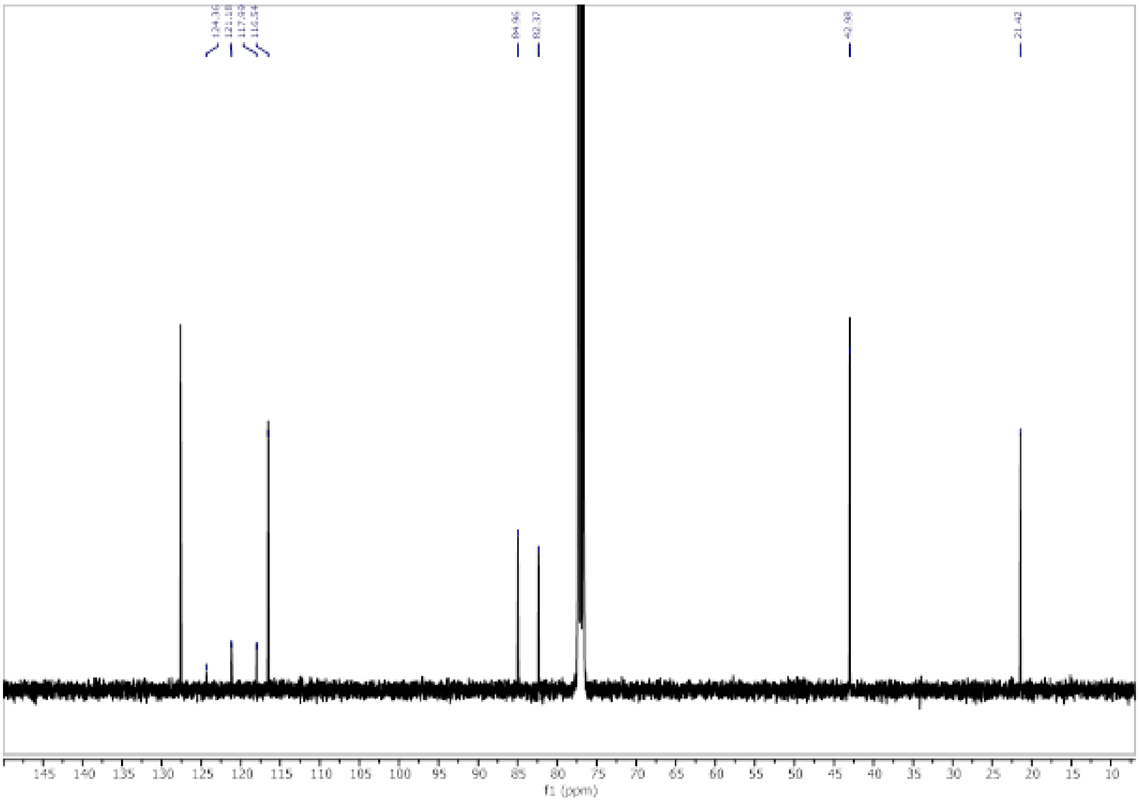

N-(hex-5-en-3-yn-1-yl)-4-methoxyaniline (S27)

^{1}H NMR (400 MHz, $CDCl_3$)

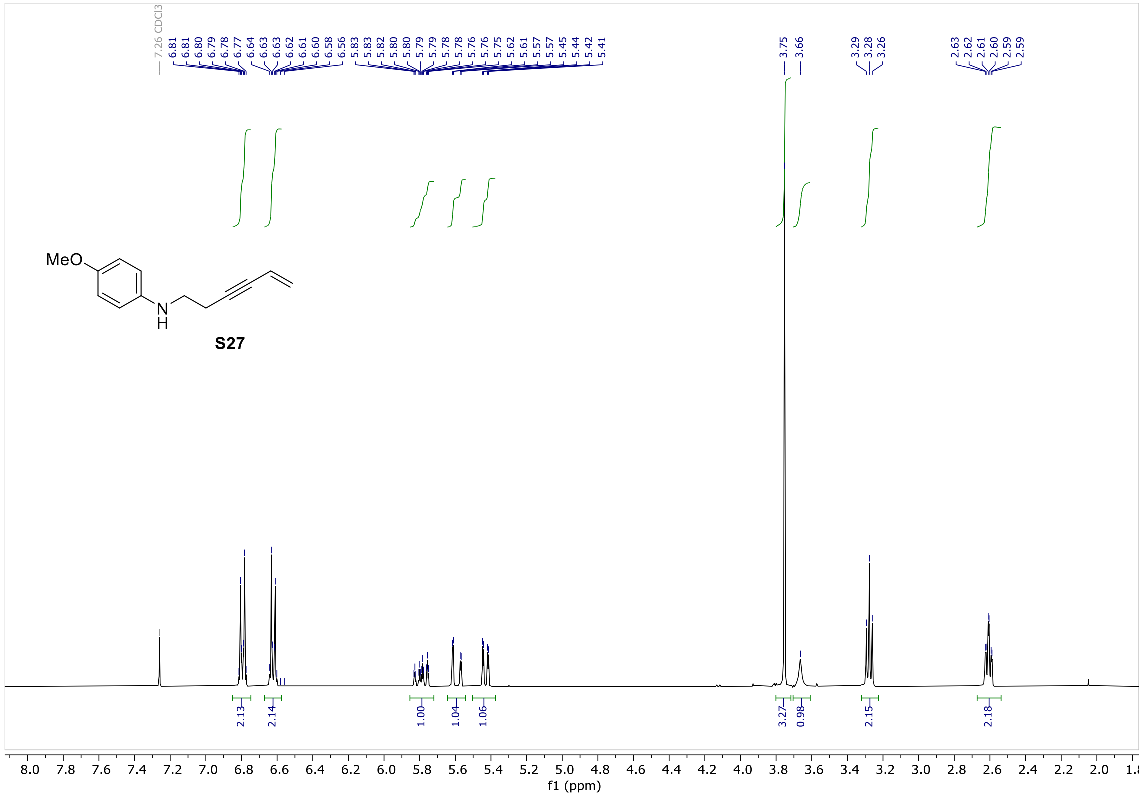

^{13}C NMR (101 MHz, $CDCl_3$)

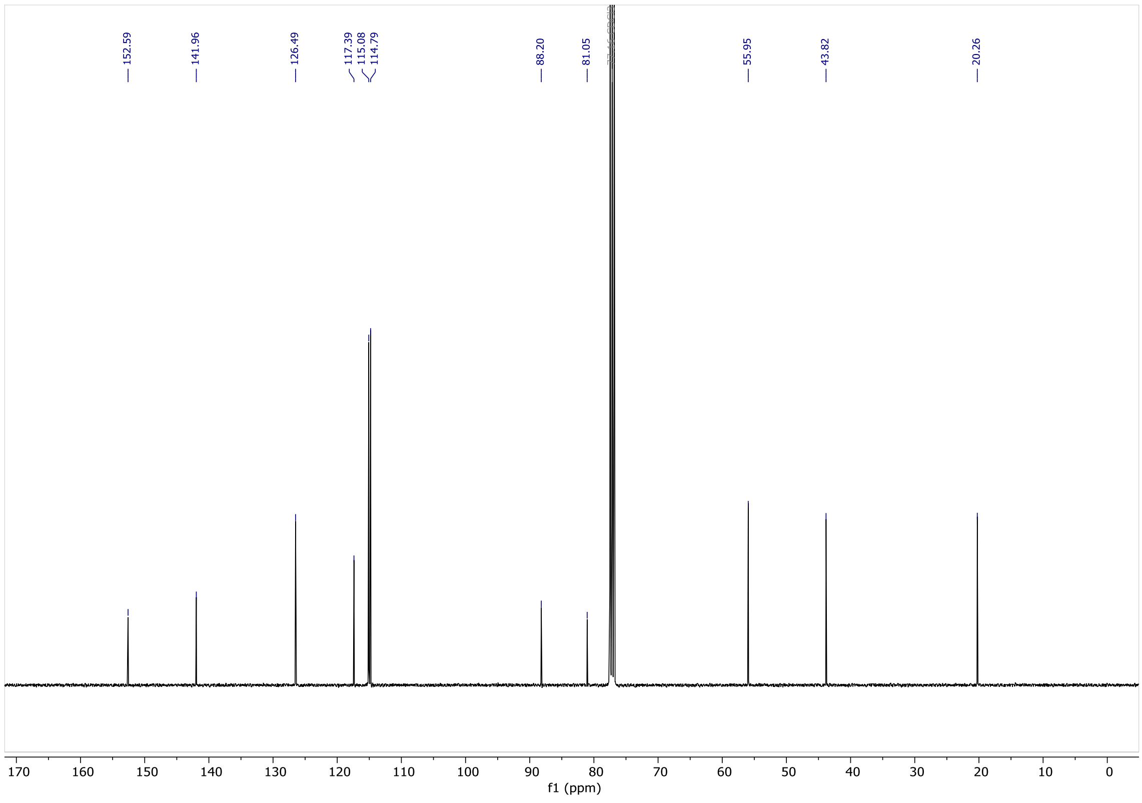

4-Methoxy-*N*-methyl-*N*-(2-(1-((trifluoromethyl)sulfonyl)pyrrolidin-2-ylidene)ethyl)aniline (S35)

^{1}H NMR (400 MHz, $CDCl_3$)

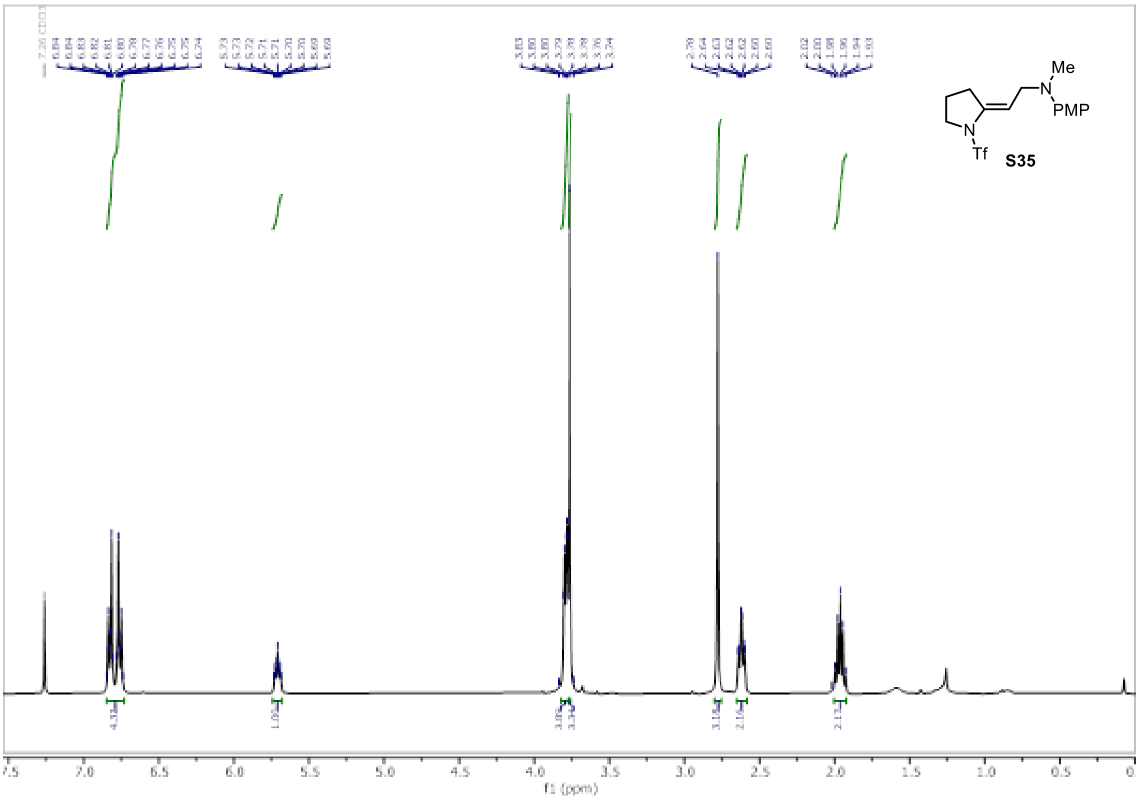

^{13}C NMR (101 MHz, $CDCl_3$)

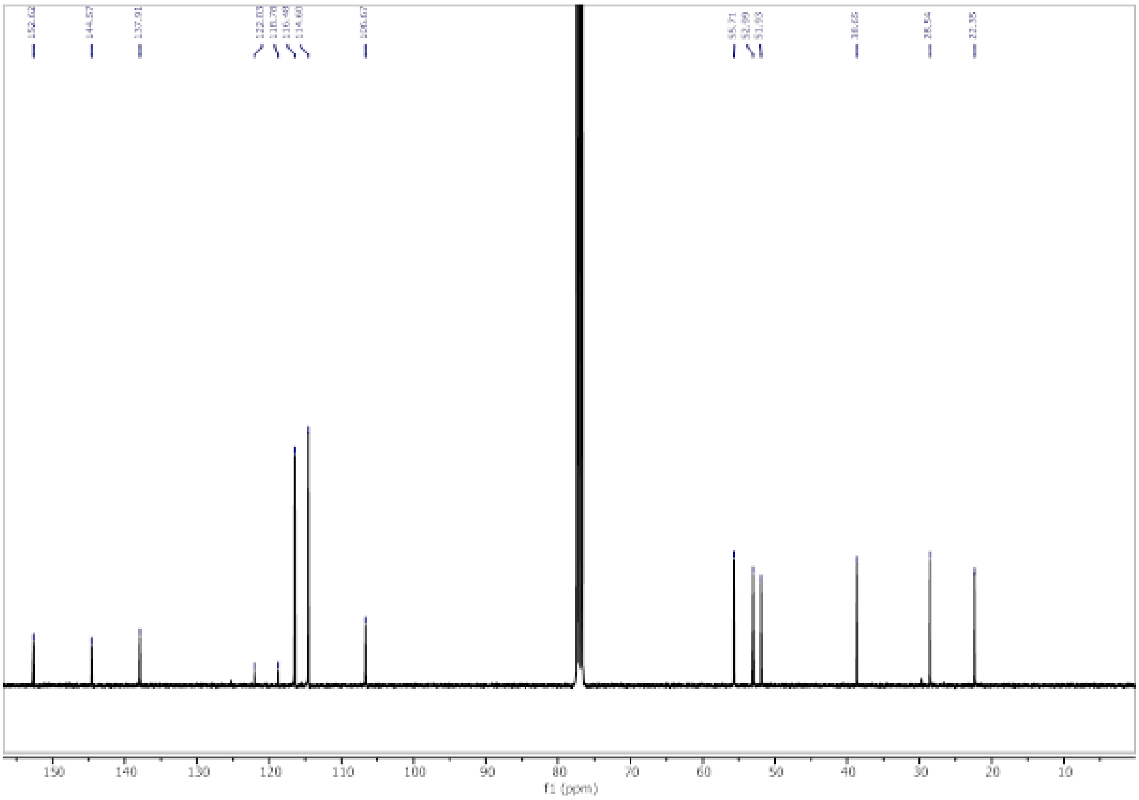

***N*-Methyl-3,4-dihydro-2*H*-benzo[b][1,4]dioxepin-7-amine (2h)**

^{1}H NMR (400 MHz, $CDCl_3$)

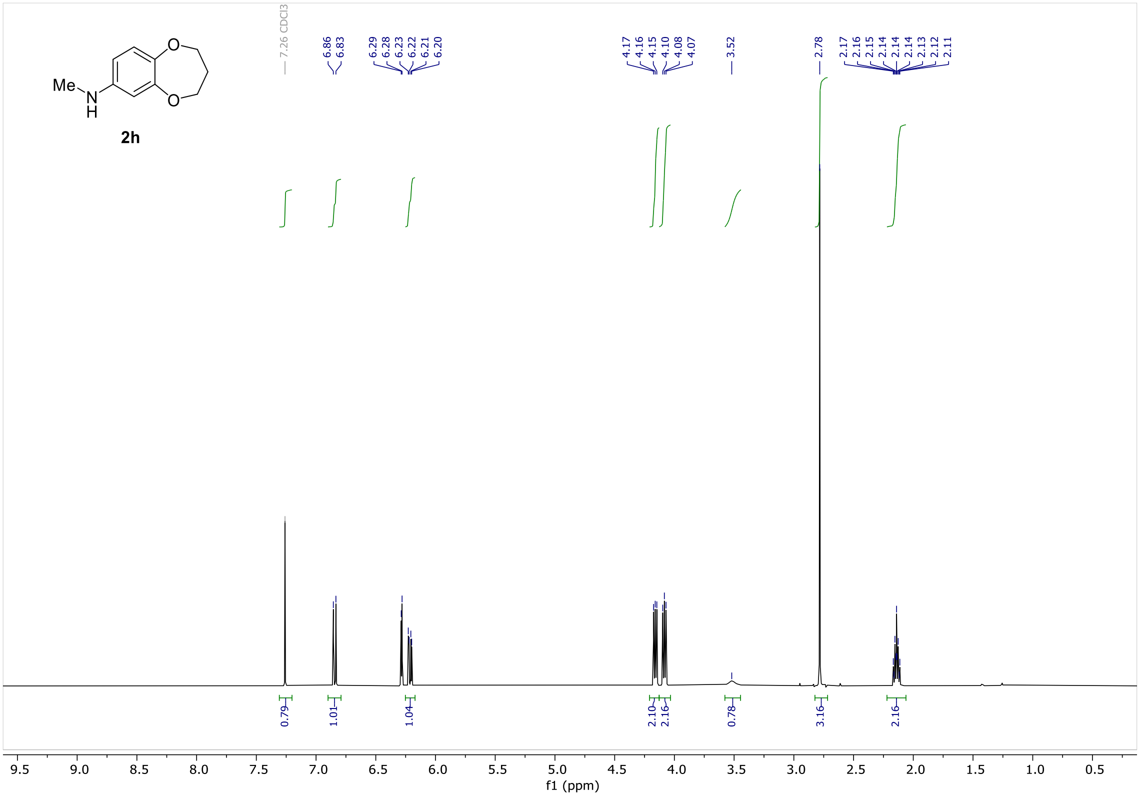

^{13}C NMR (101 MHz, $CDCl_3$)

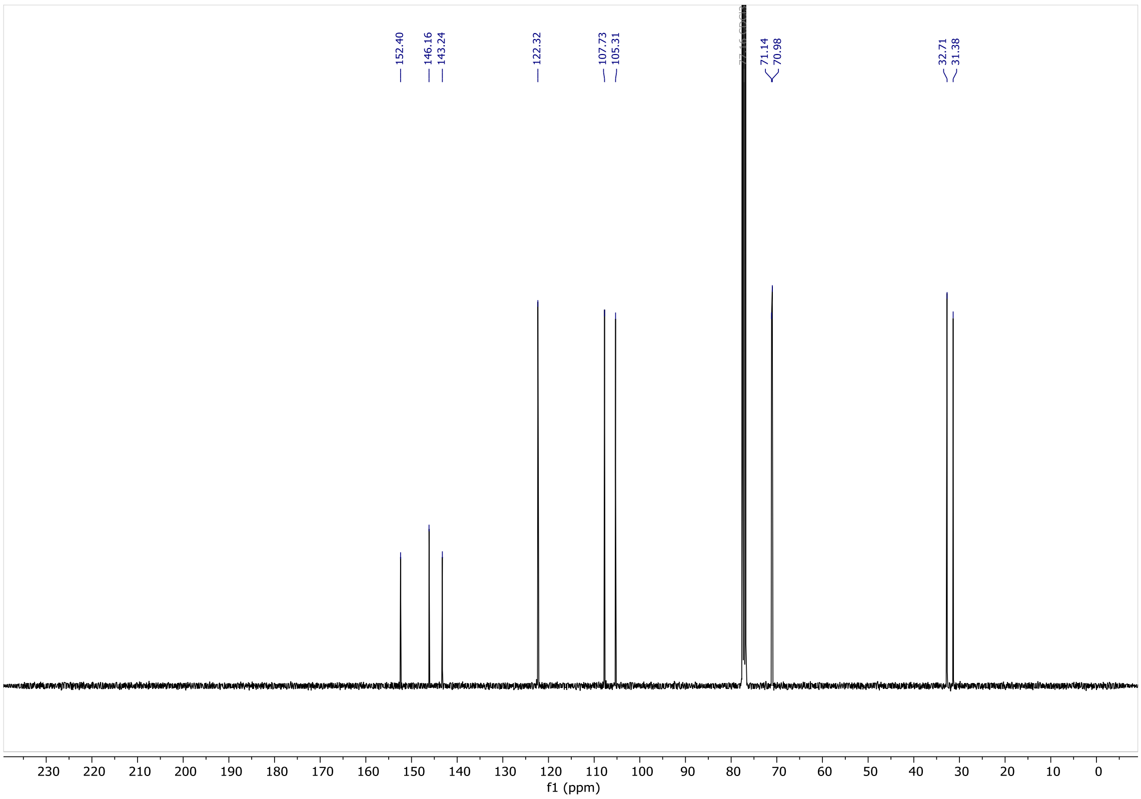

I.2. Spectra of allenes

6-((4-Methoxyphenyl)(methyl)amino)-3-(p-tolyl)hexa-3,4-dien-1-ol (4aaa)

^{1}H NMR (400 MHz, $CDCl_3$)

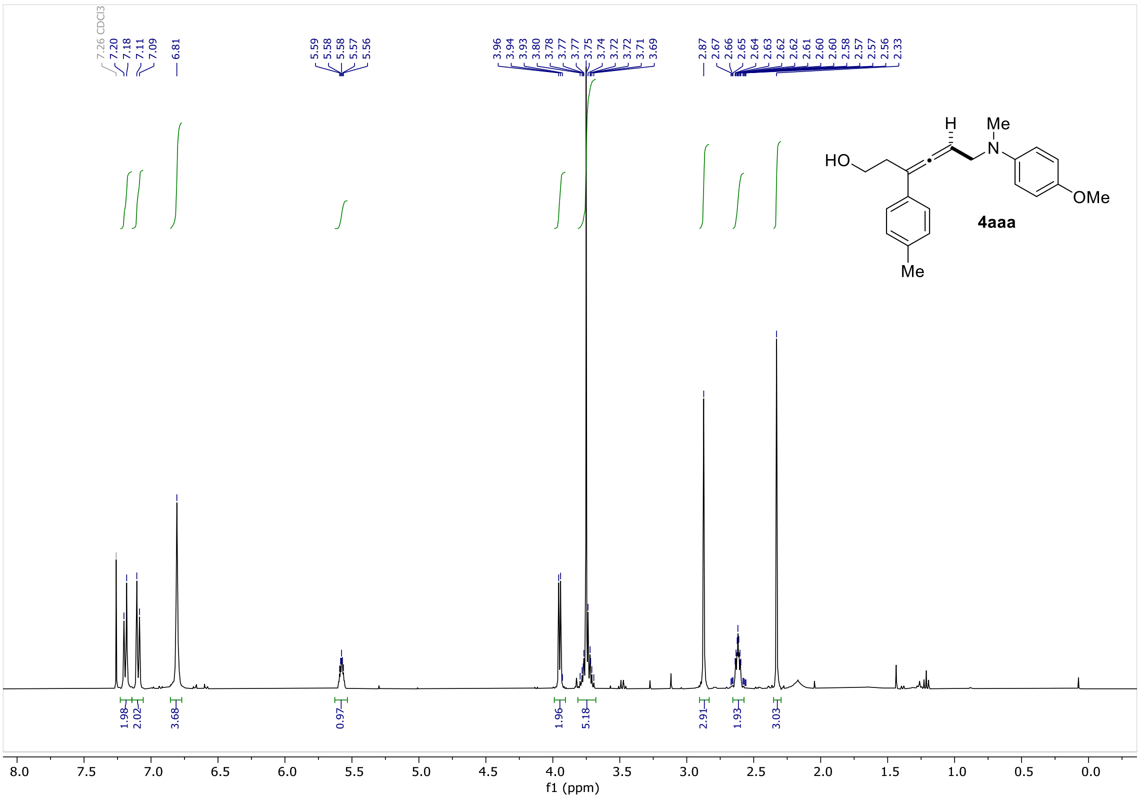

^{13}C NMR (101 MHz, $CDCl_3$)

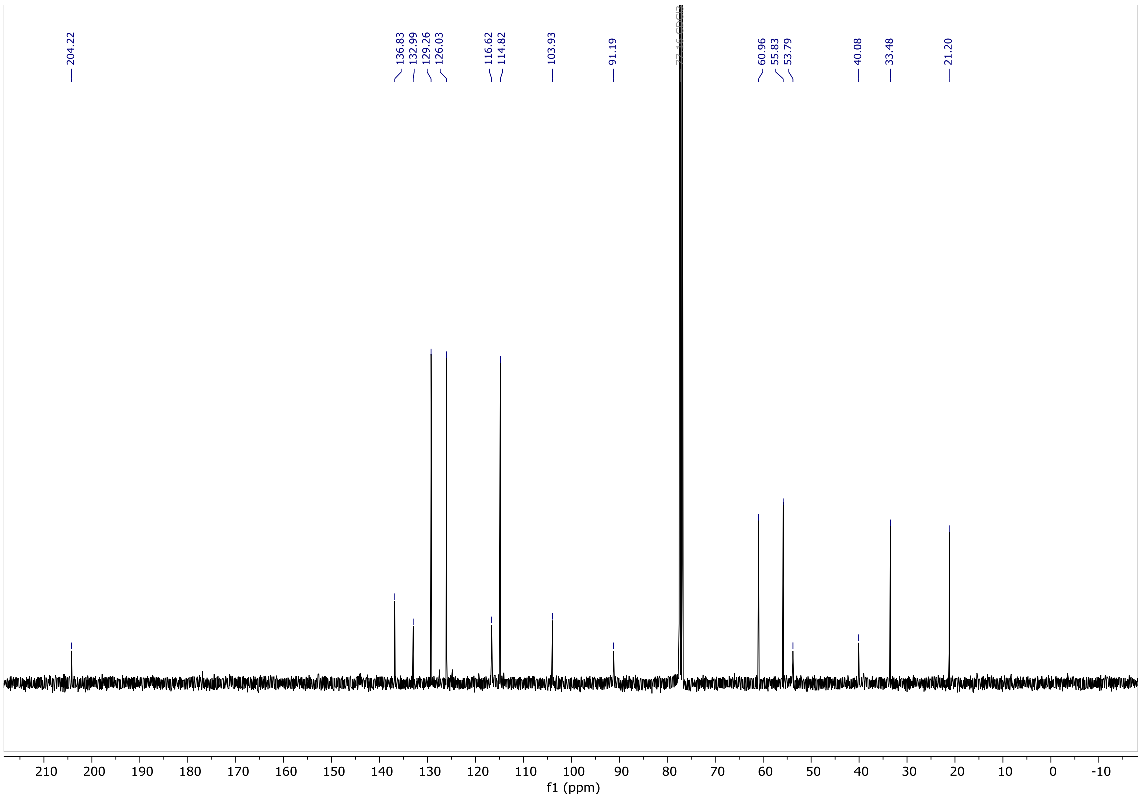

6-((4-Chlorophenyl)(methyl)amino)-3-(*p*-tolyl)hexa-3,4-dien-1-ol (4aba)

^{1}H NMR (400 MHz, $CDCl_3$)

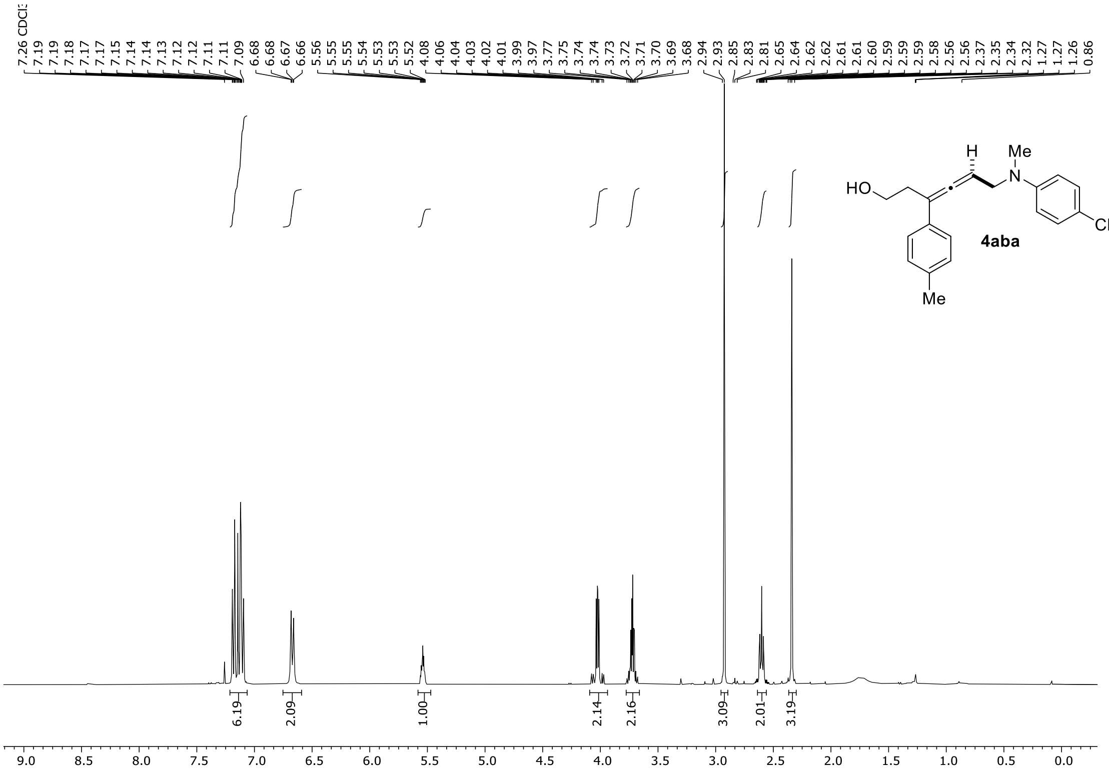

^{13}C NMR (101 MHz, $CDCl_3$)

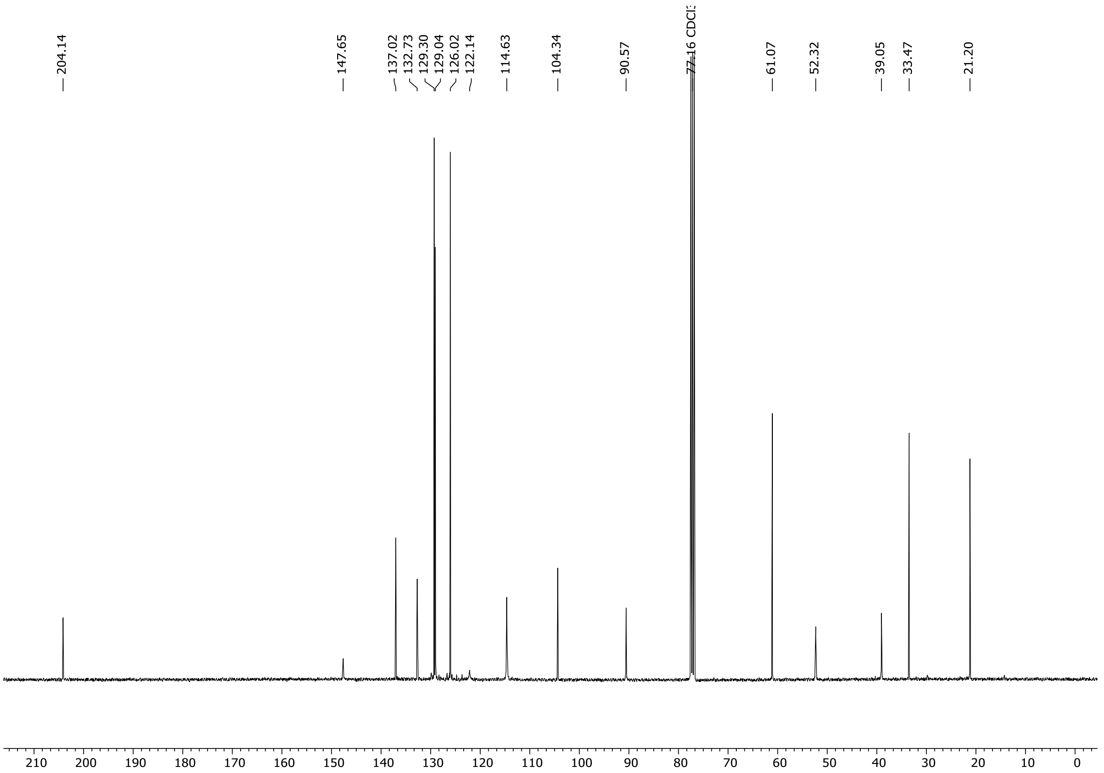

Methyl 4-((6-hydroxy-4-(*p*-tolyl)hexa-2,3-dien-1-yl)(methyl)amino)benzoate (4aca)

^{1}H NMR (400 MHz, CDCl$_3$)

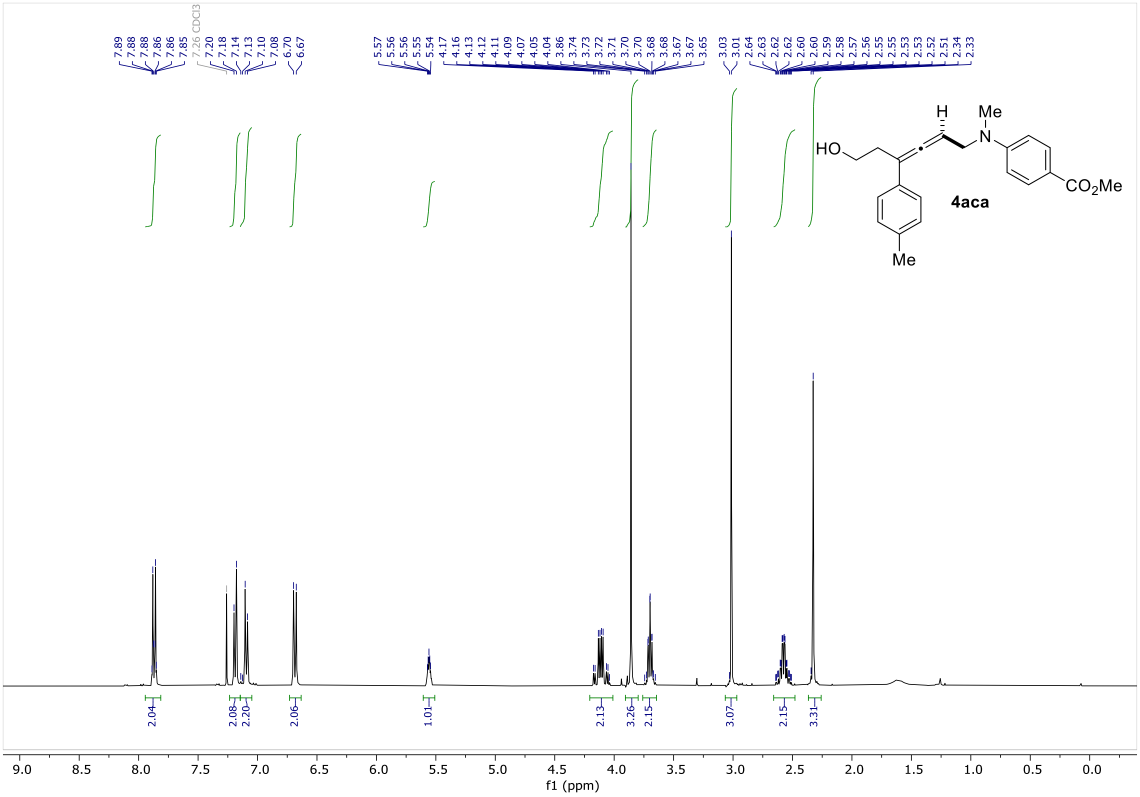

^{13}C NMR (101 MHz, CDCl$_3$)

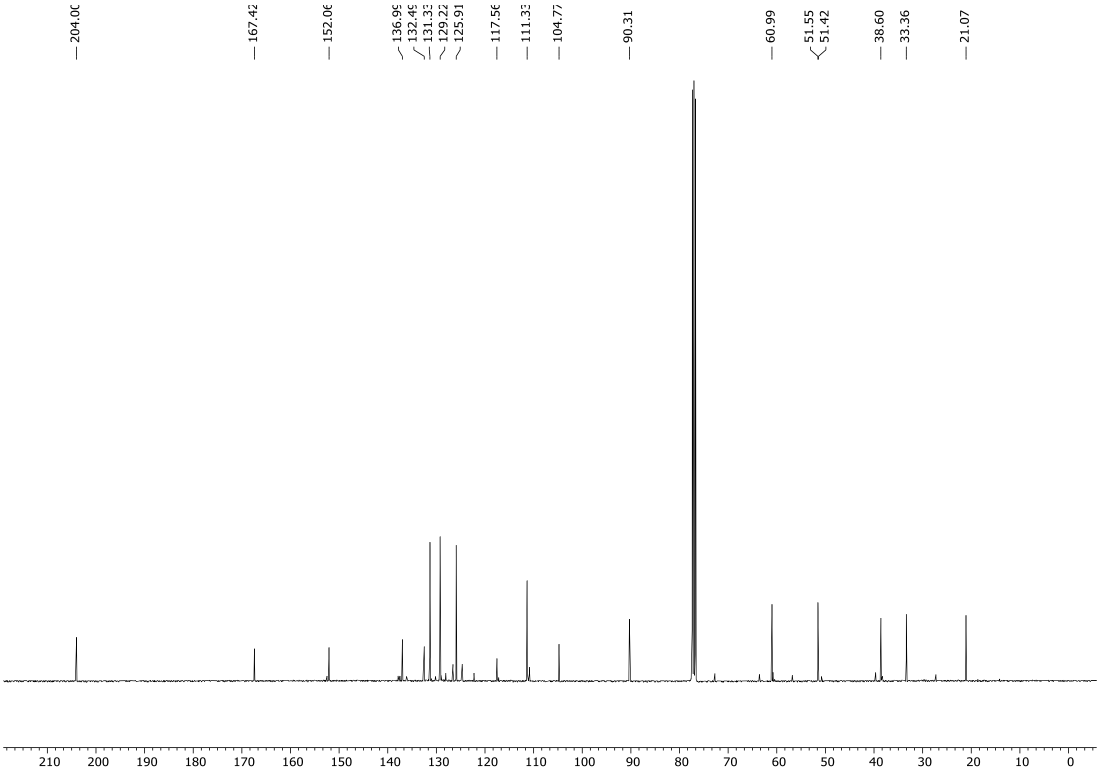

6-(Methyl(*p*-tolyl)amino)-3-(*p*-tolyl)hexa-3,4-dien-1-ol (4ada)

^{1}H NMR (400 MHz, $CDCl_3$)

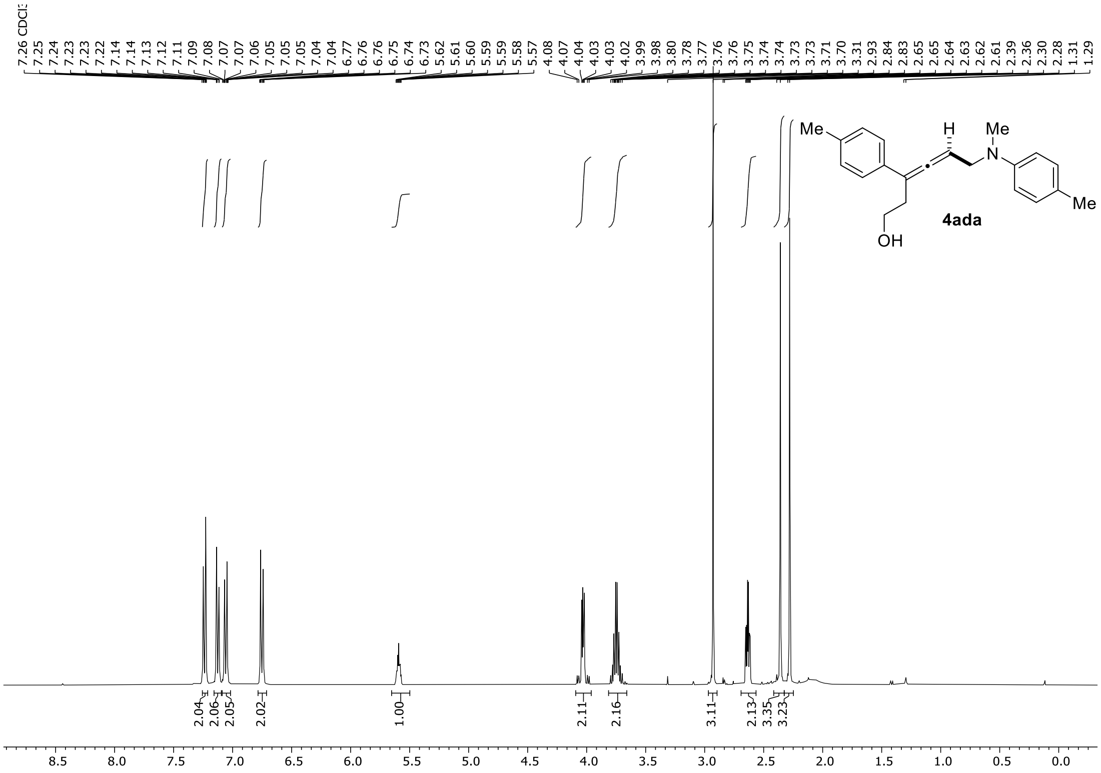

^{13}C NMR (101 MHz, $CDCl_3$)

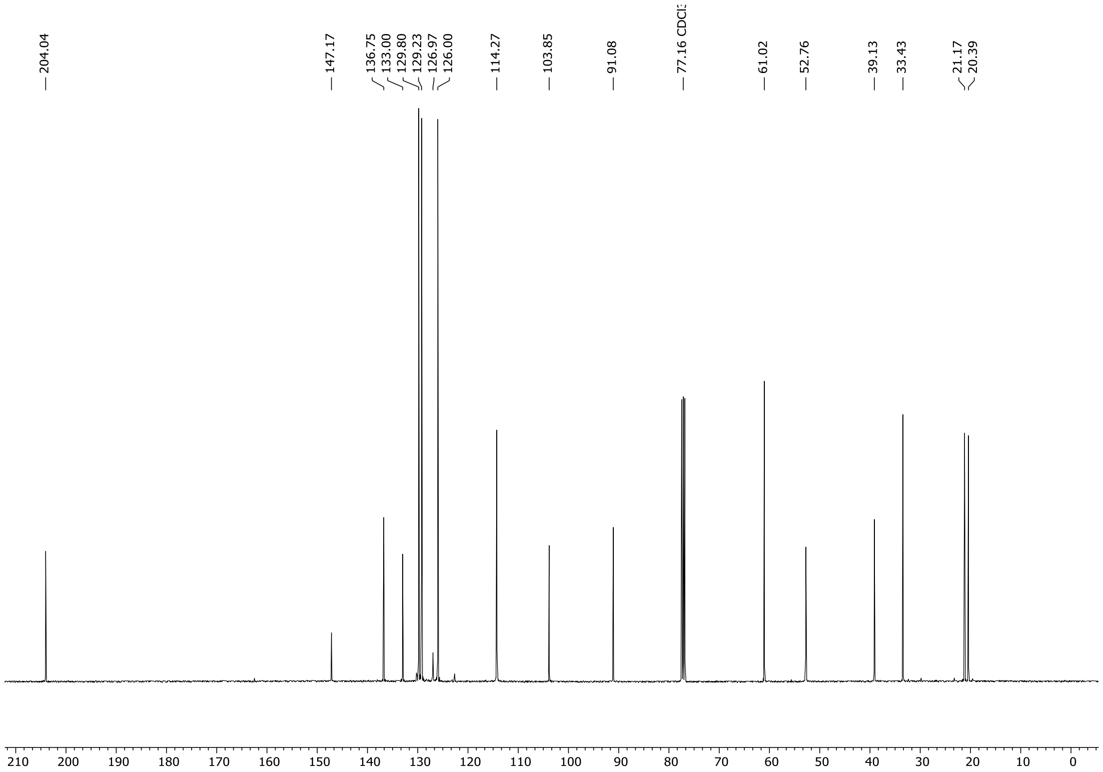

Methyl 4-(1-hydroxy-6-((4-methoxyphenyl)(methyl)amino)hexa-3,4-dien-3-yl)benzoate (4aab)

^{1}H NMR (400 MHz, $CDCl_3$)

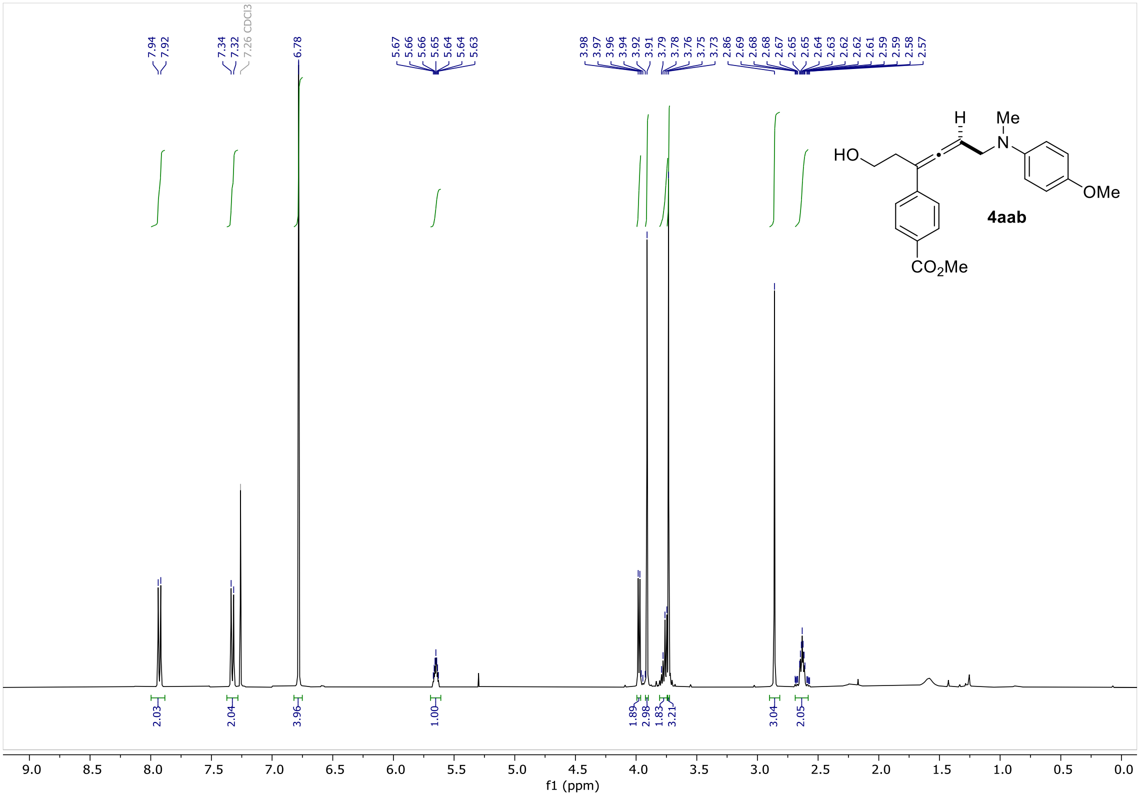

^{13}C NMR (101 MHz, $CDCl_3$)

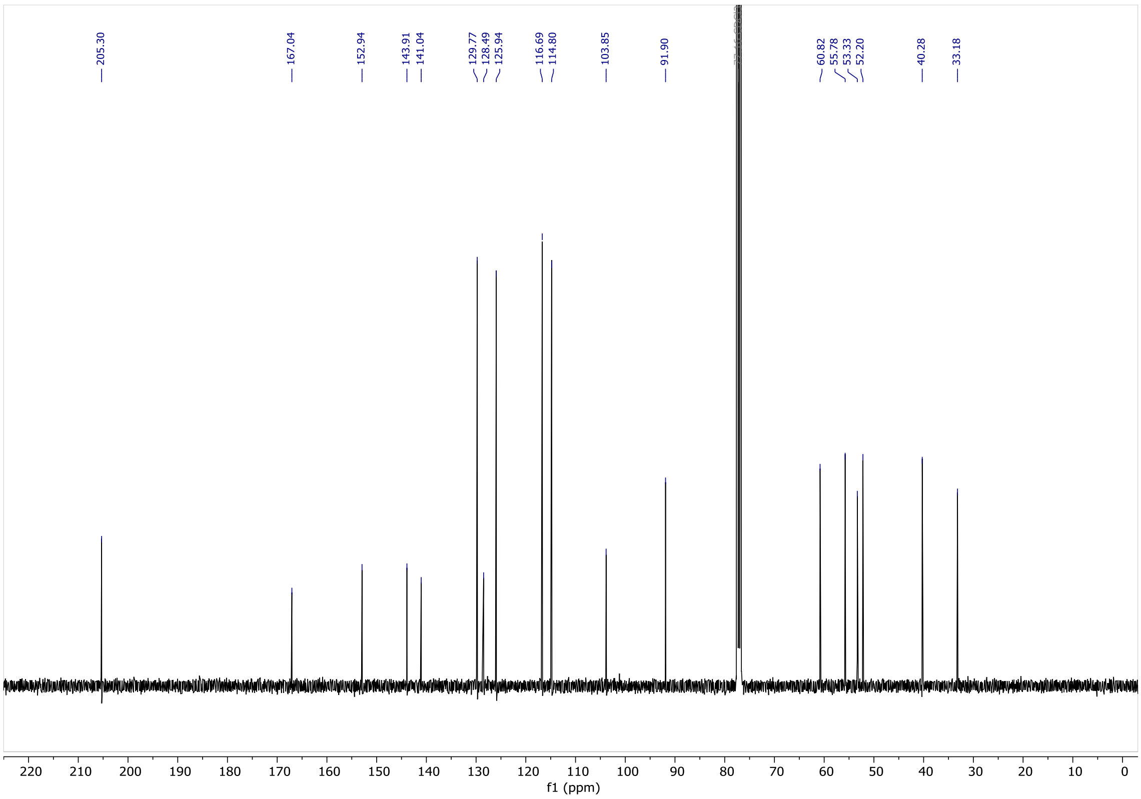

Methyl 4-(1-hydroxy-6-(methyl(4-(trifluoromethyl)phenyl)amino)hexa-3,4-dien-3-yl)benzoate (4aeb)

^{1}H NMR (400 MHz, $CDCl_3$)

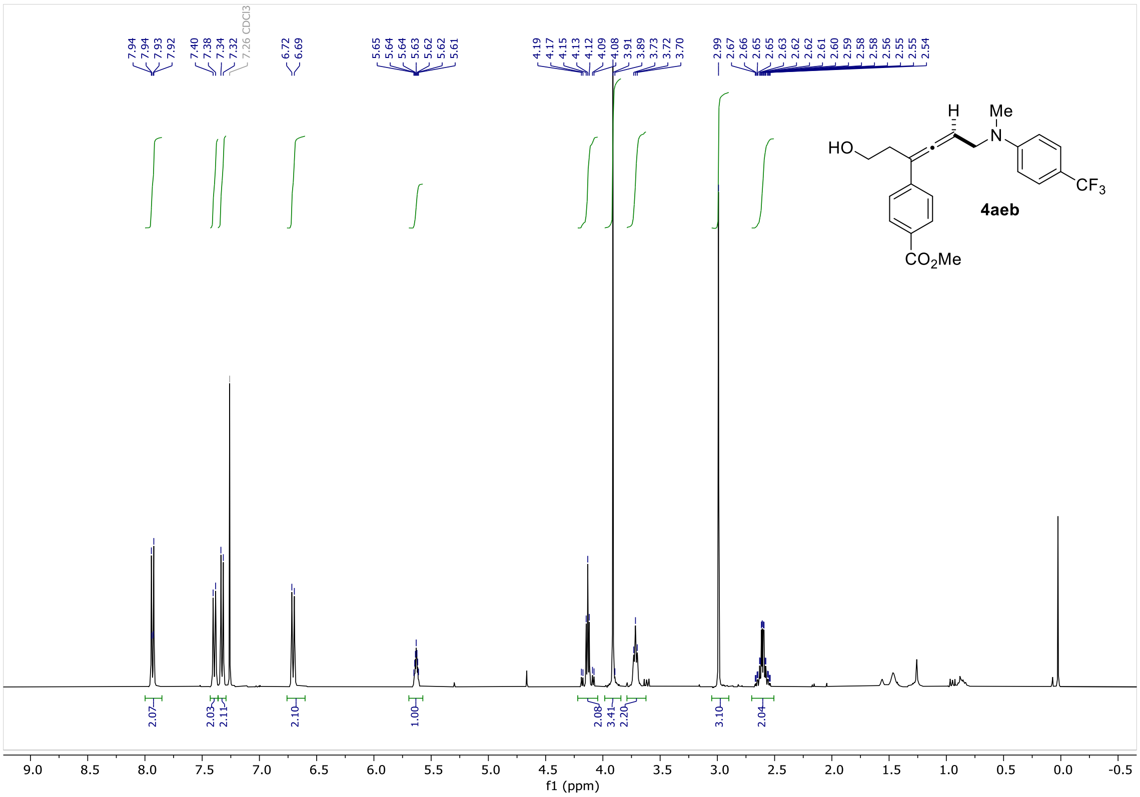

^{13}C NMR (101 MHz, $CDCl_3$)

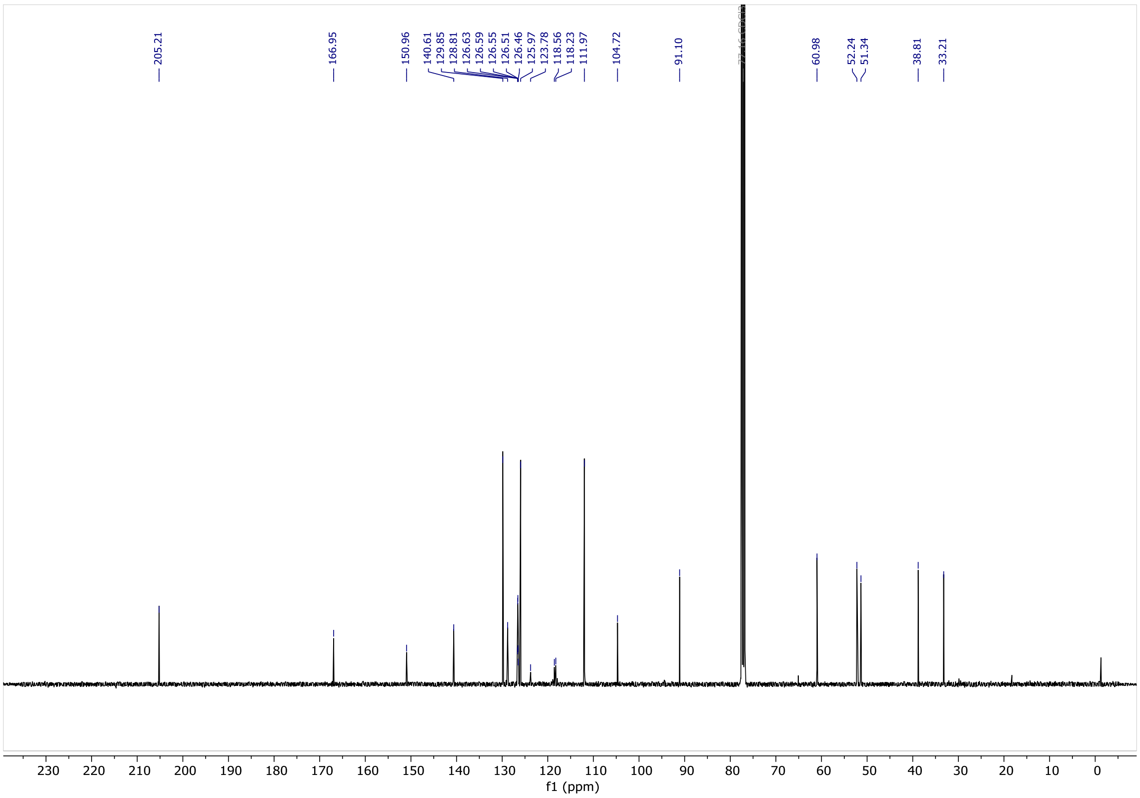

Methyl 4-(1-hydroxy-6-(methyl(naphthalen-2-yl)amino)hexa-3,4-dien-3-yl)benzoate (4afb)

^{1}H NMR (400 MHz, $CDCl_3$)

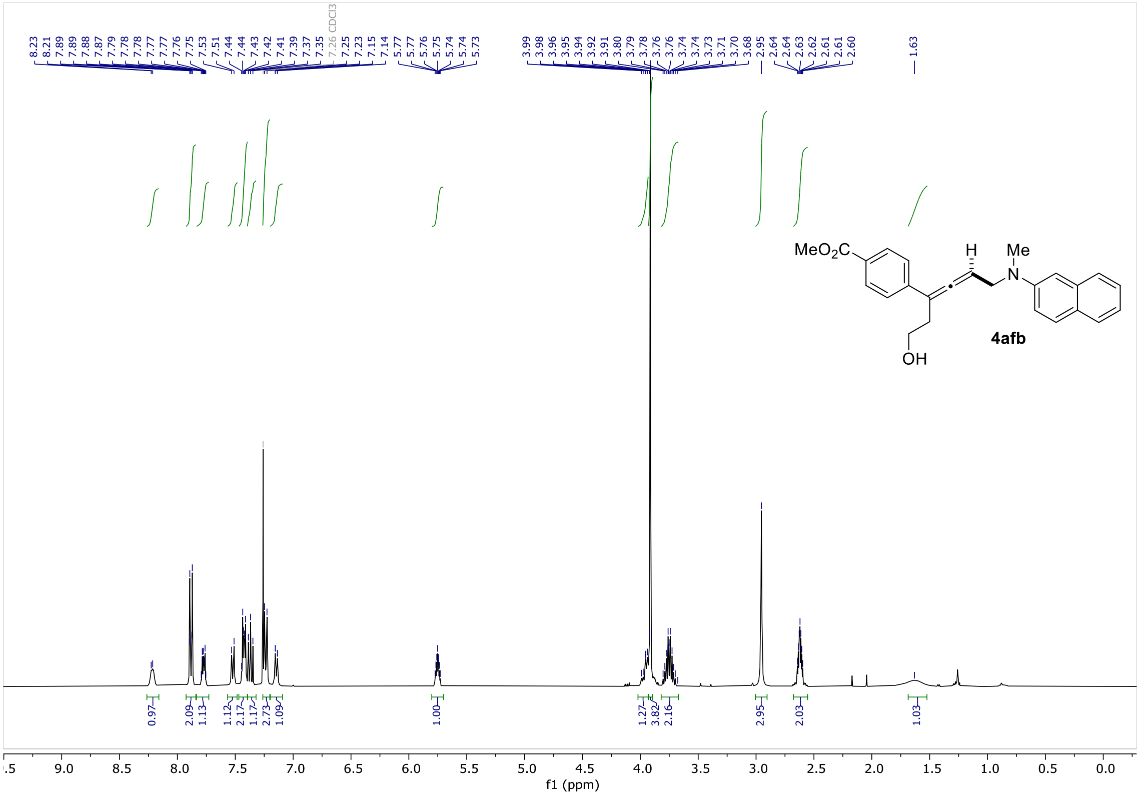

^{13}C NMR (101 MHz, $CDCl_3$)

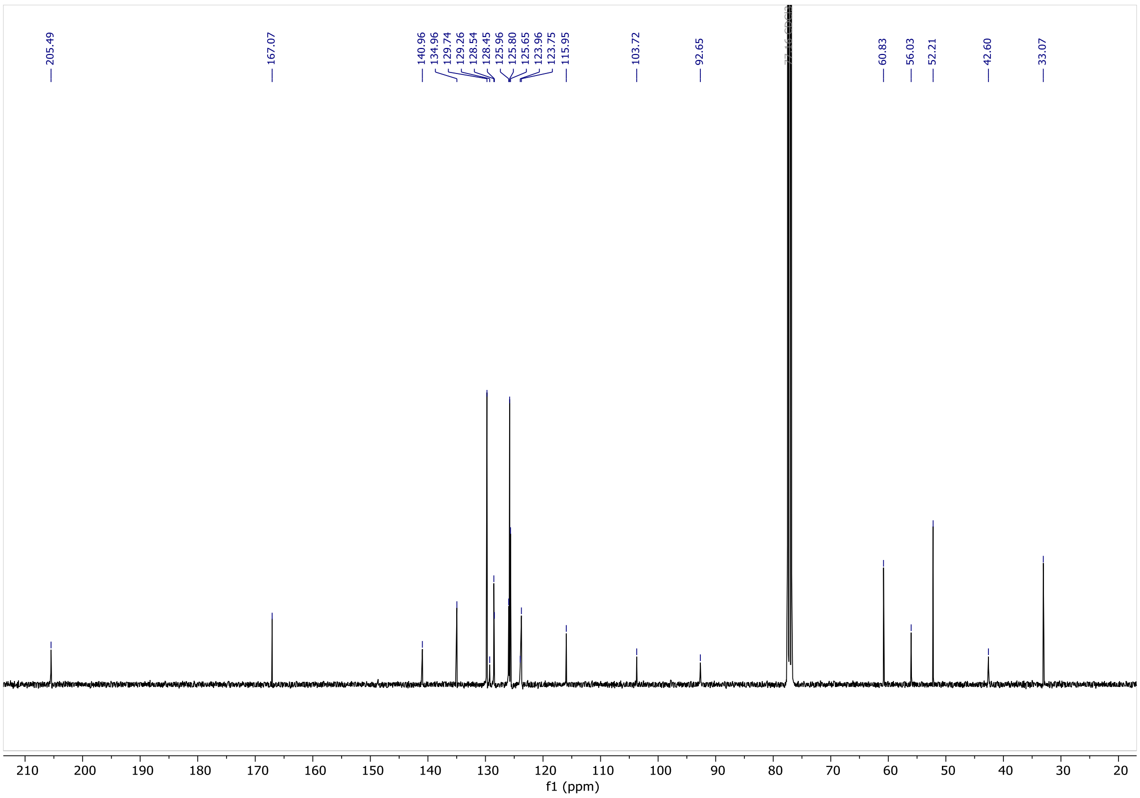

Methyl 4-(6-((3-(*tert*-butyl)phenyl)(methyl)amino)-1-hydroxyhexa-3,4-dien-3-yl)benzoate (4agb)

^{1}H NMR (400 MHz, $CDCl_3$)

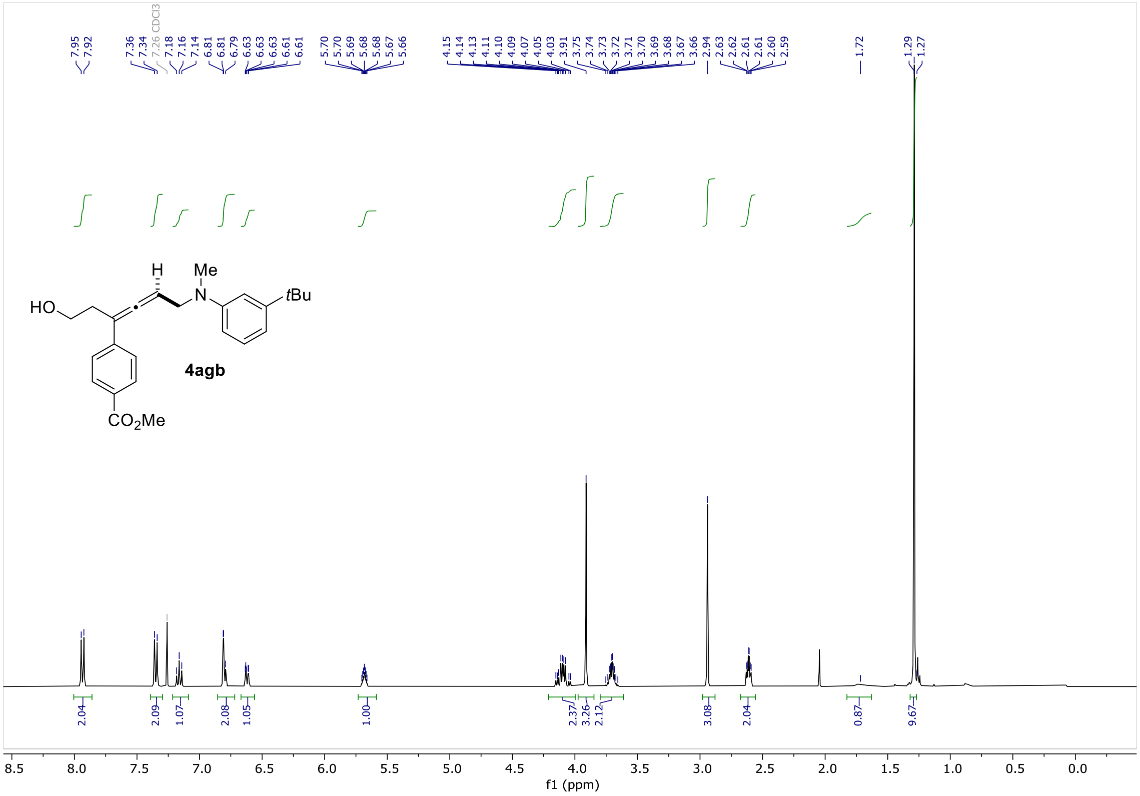

^{13}C NMR (101 MHz, $CDCl_3$)

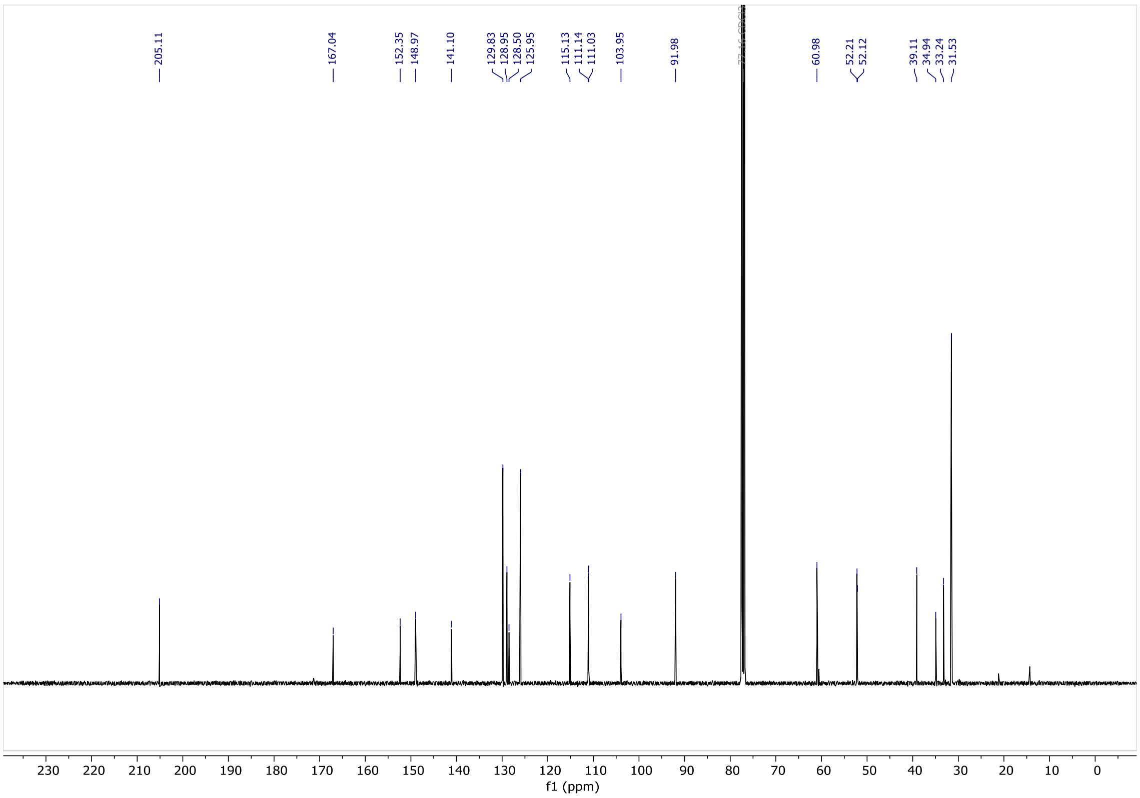

Methyl 4-(6-((3,4-dihydro-2H-benzo[b][1,4]dioxepin-7-yl)(methyl)amino)-1-hydroxyhexa-3,4-dien-3-yl)benzoate (4ahb)

^{1}H NMR (400 MHz, $CDCl_3$)

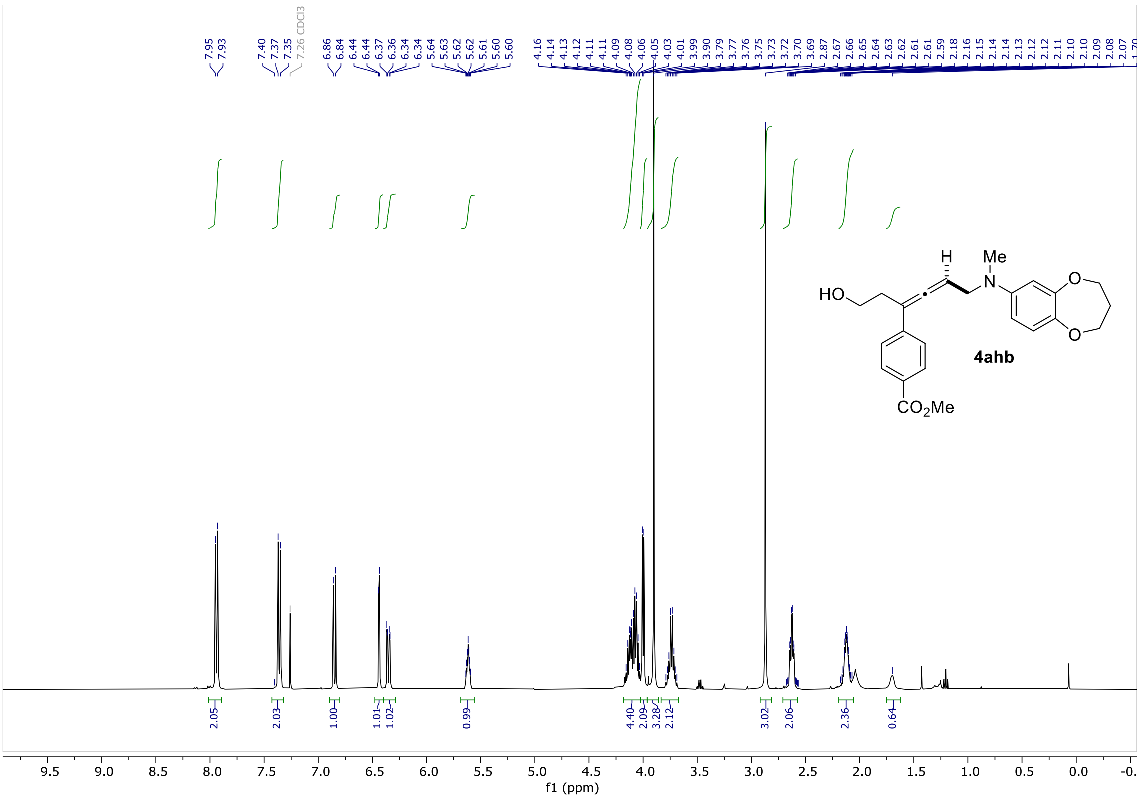

^{13}C NMR (101 MHz, $CDCl_3$)

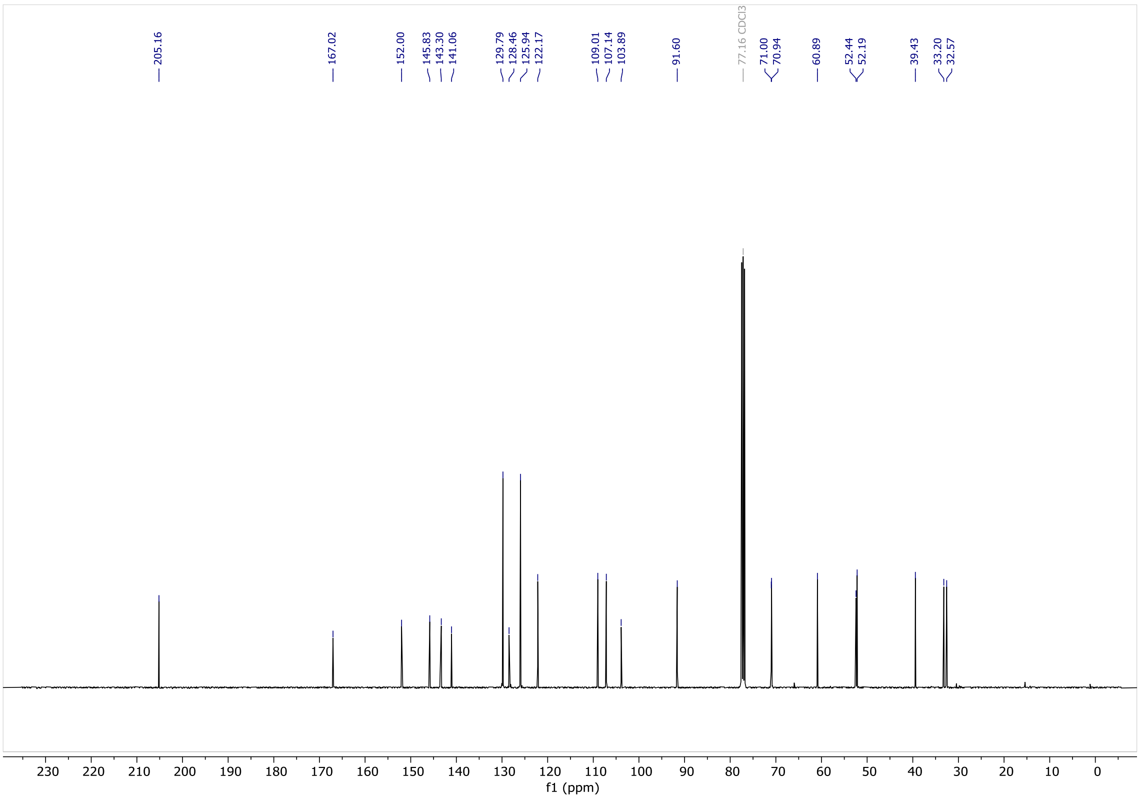

Methyl 4-(1-hydroxy-6-(methyl(o-tolyl)amino)hexa-3,4-dien-3-yl)benzoate (4aib)

^{1}H NMR (400 MHz, $CDCl_3$)

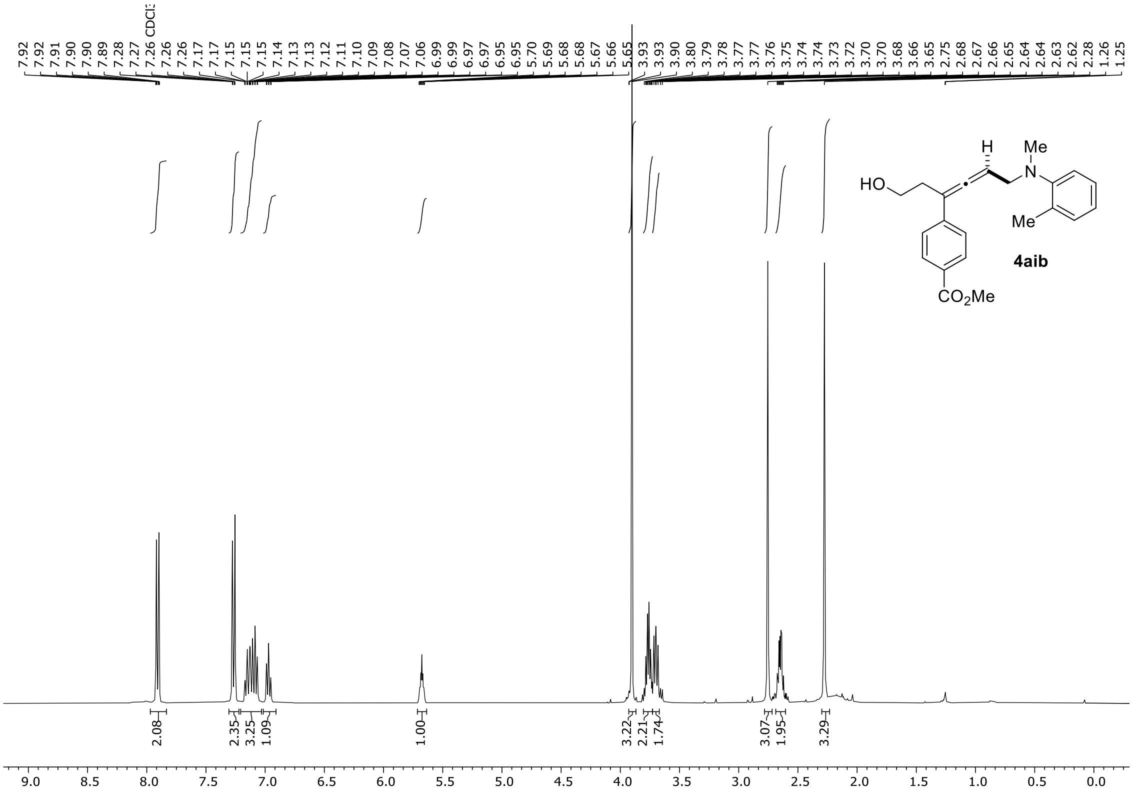

^{13}C NMR (101 MHz, $CDCl_3$)

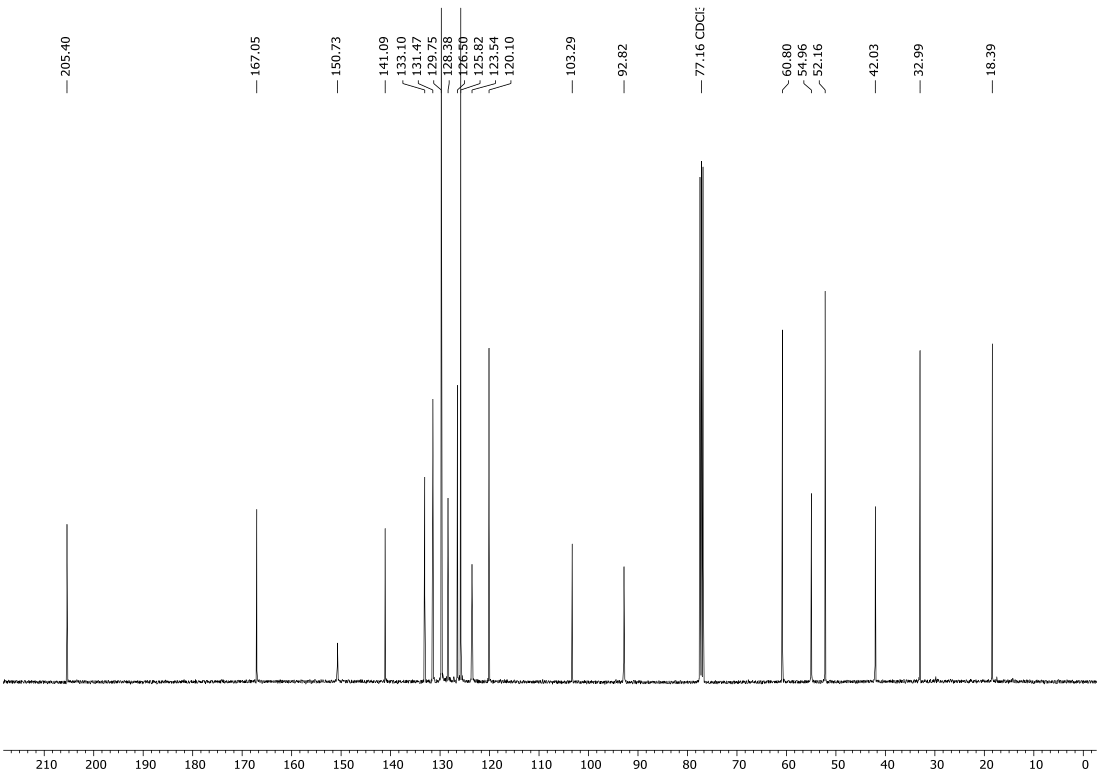

Methyl 4-(1-hydroxy-6-((4-methoxyphenyl)(phenethyl)amino)hexa-3,4-dien-3-yl)benzoate (4ajb)

^{1}H NMR (400 MHz, $CDCl_3$)

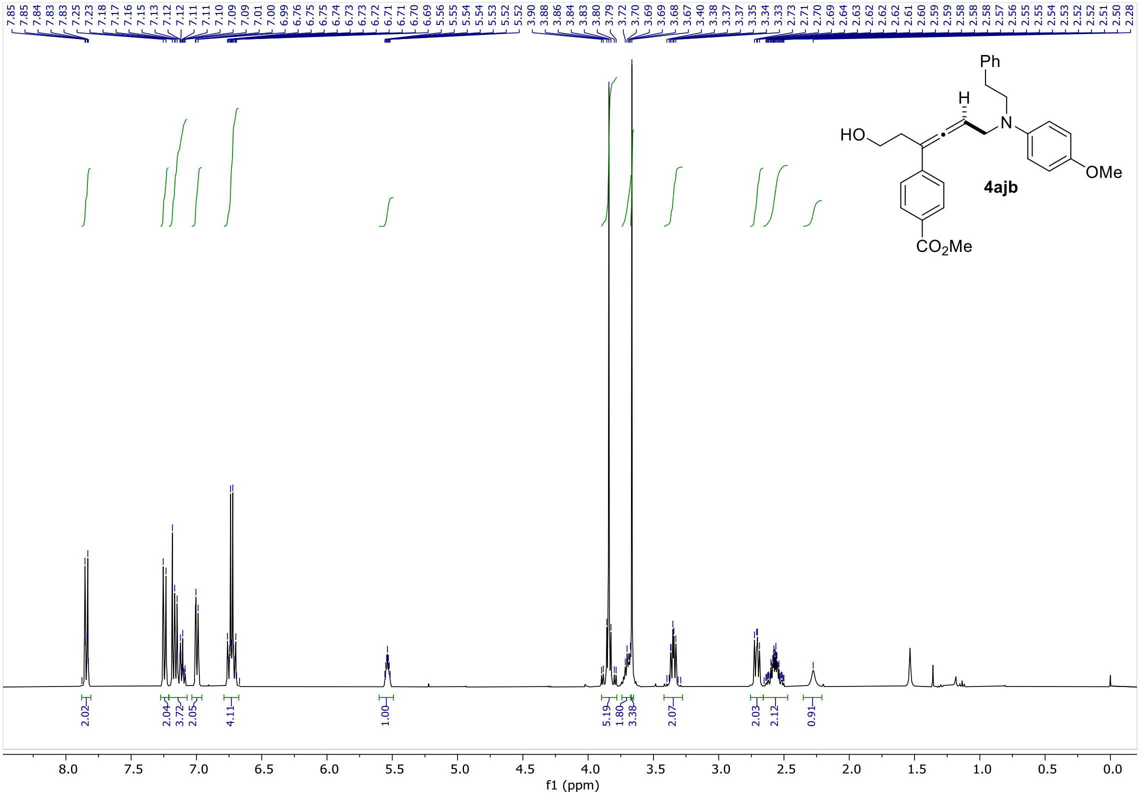

^{13}C NMR (101 MHz, $CDCl_3$)

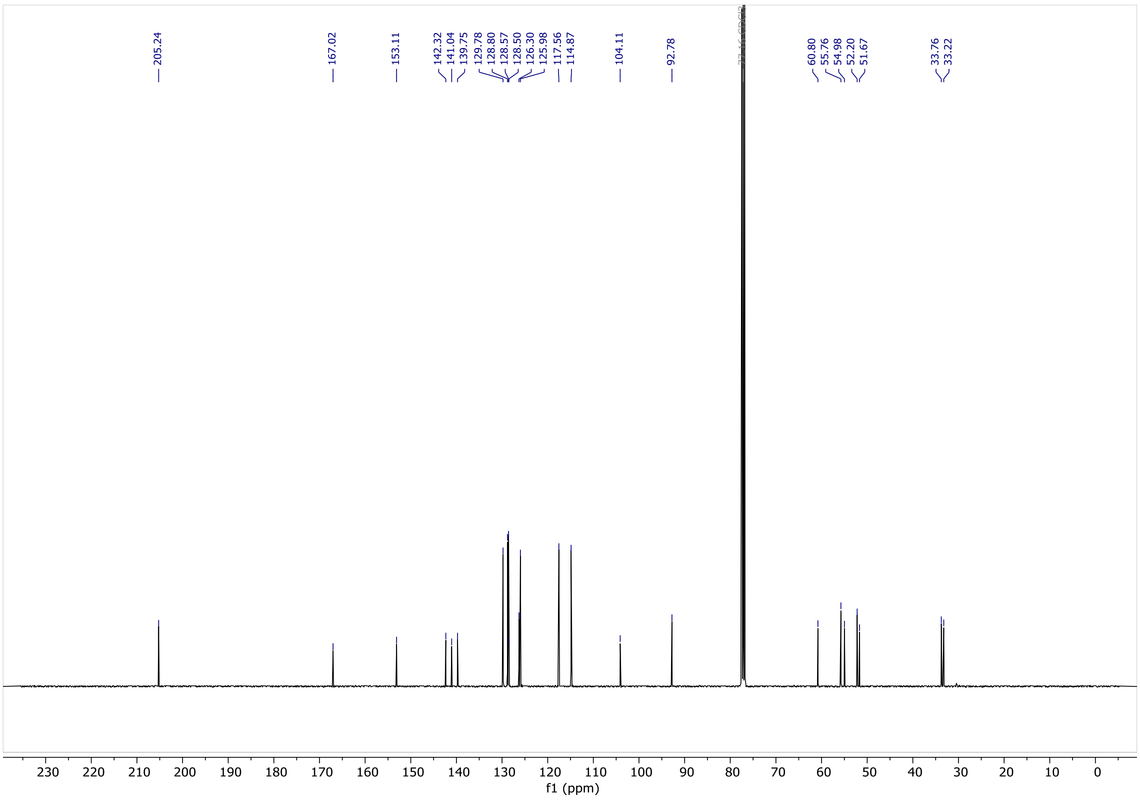

Methyl 4-(1-hydroxy-6-((2-hydroxyethyl)(phenyl)amino)hexa-3,4-dien-3-yl)benzoate (4akb)

^{1}H NMR (400 MHz, $CDCl_3$)

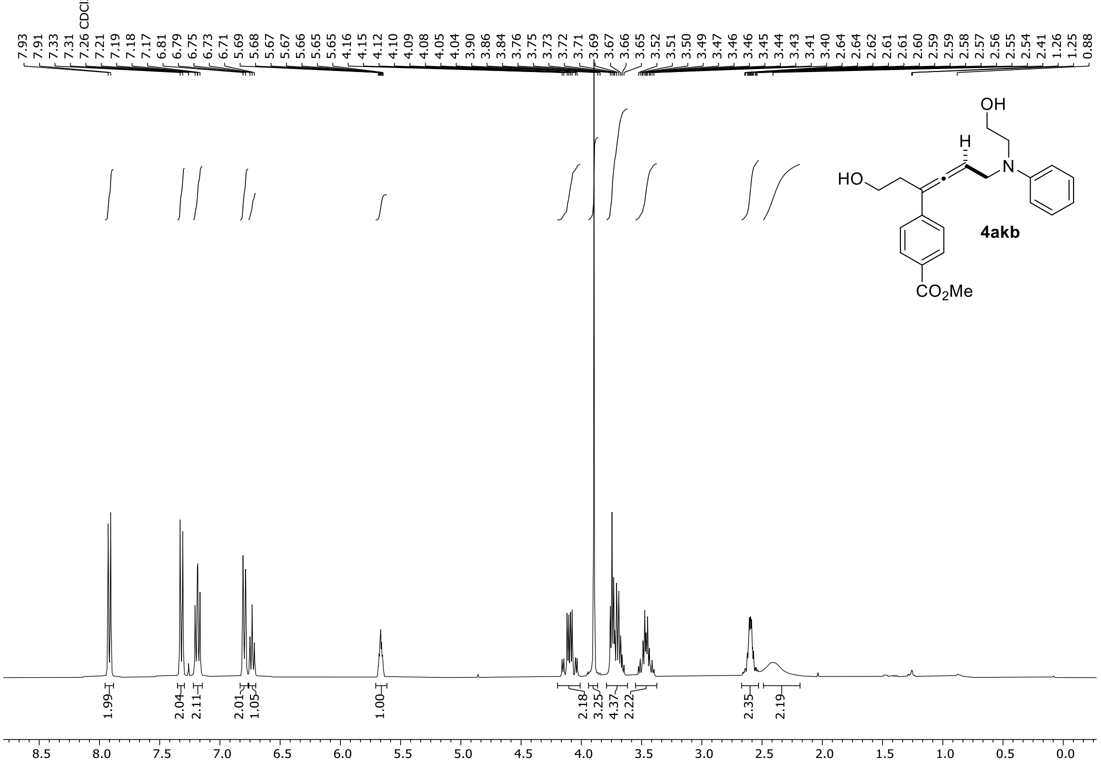

^{13}C NMR (101 MHz, $CDCl_3$)

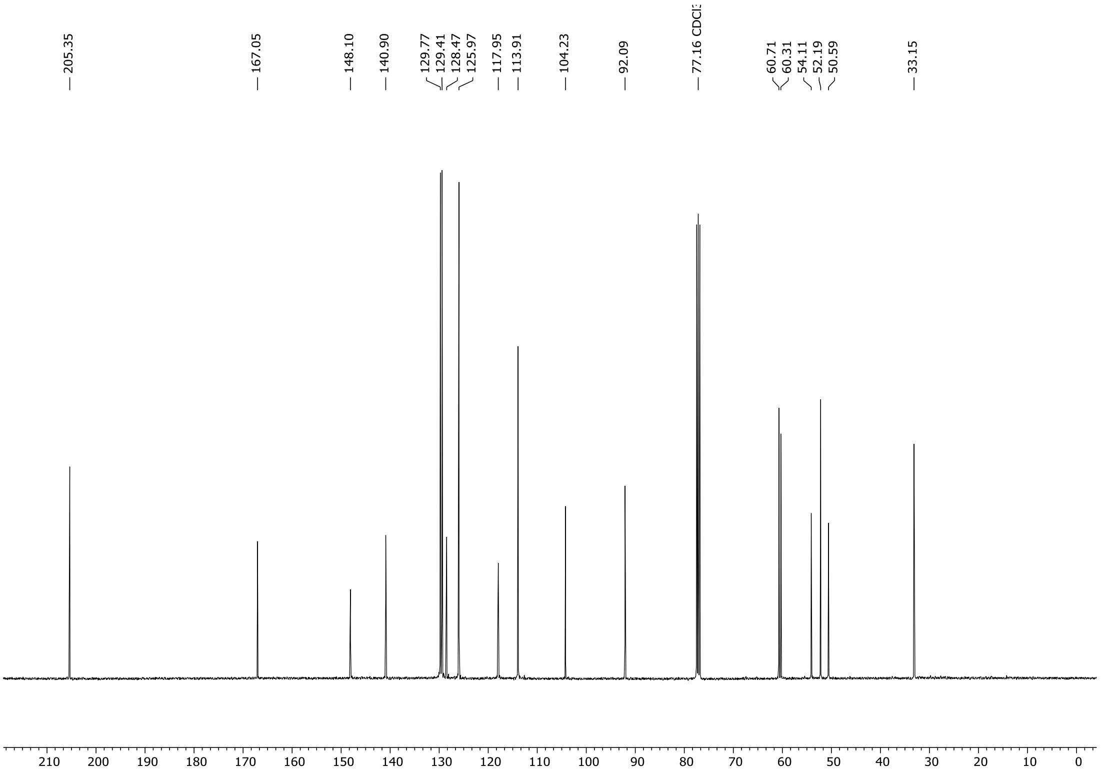

Methyl 4-(1-hydroxy-6-(phenyl(3-phenylprop-2-yn-1-yl)amino)hexa-3,4-dien-3-yl)benzoate (4alb)

^{1}H NMR (400 MHz, $CDCl_3$)

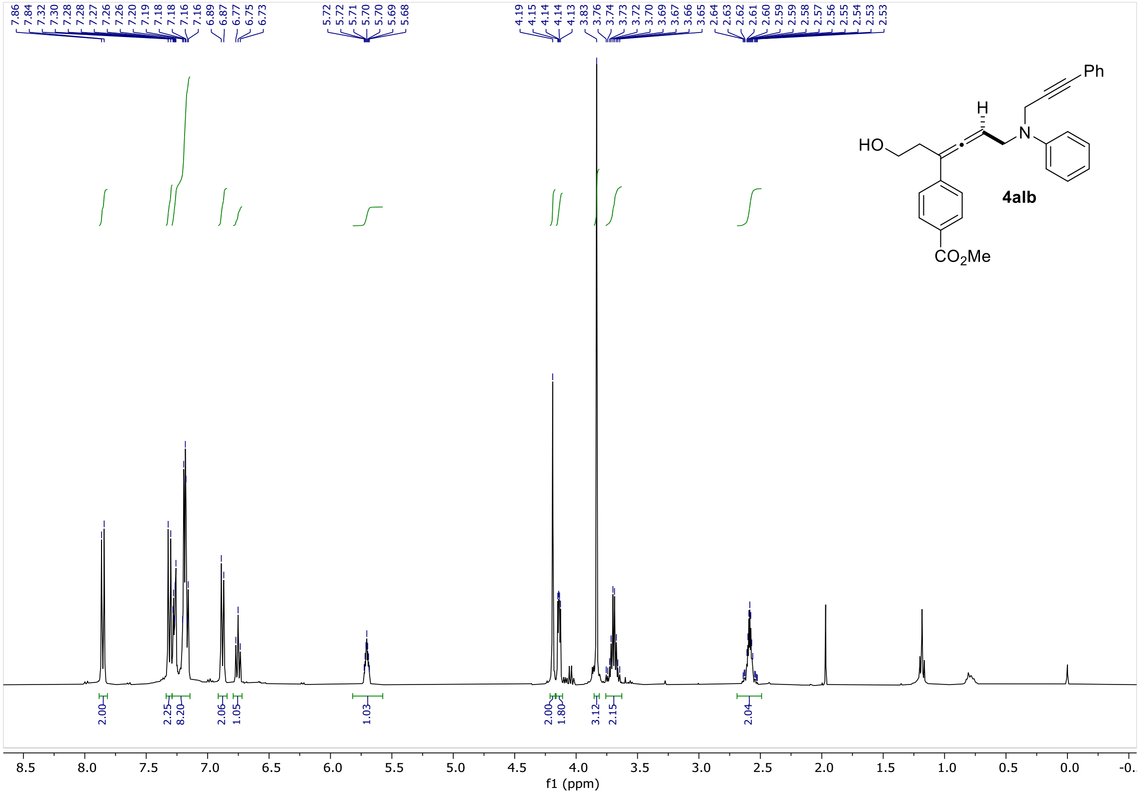

^{13}C NMR (101 MHz, $CDCl_3$)

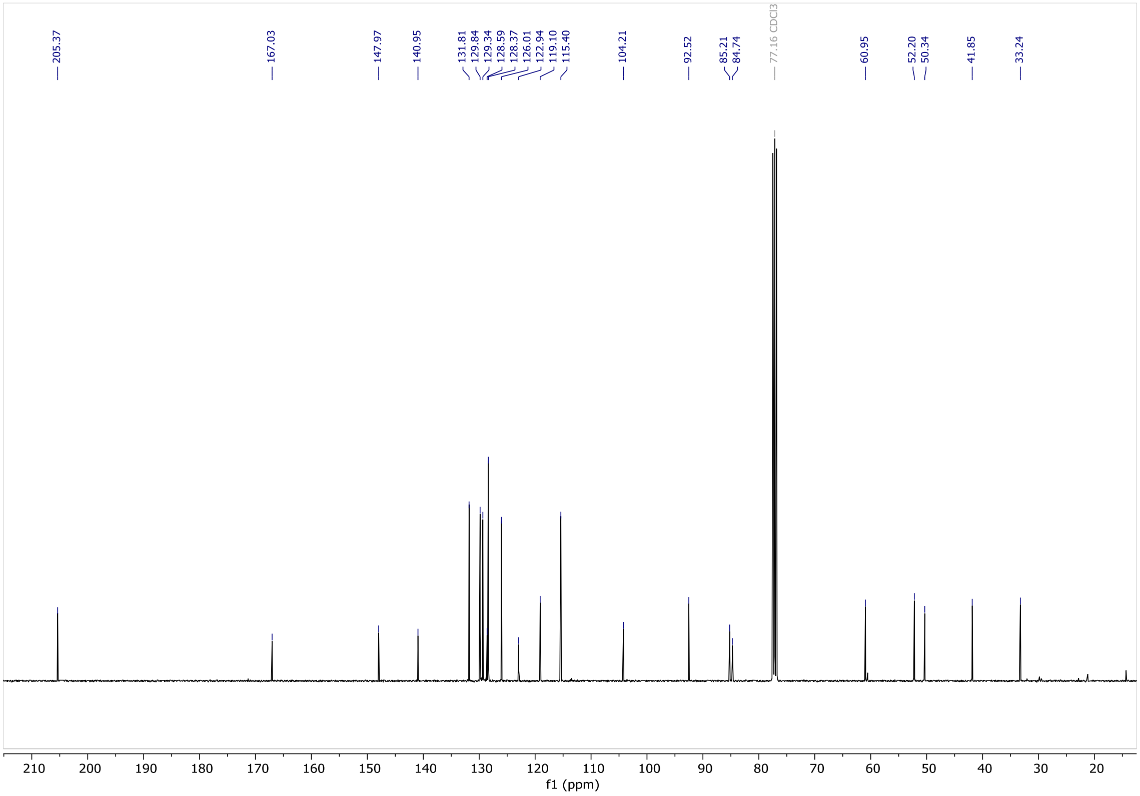

Methyl 4-(6-(benzyl(phenyl)amino)-1-hydroxyhexa-3,4-dien-3-yl)benzoate (4amb)

^{1}H NMR (400 MHz, $CDCl_3$)

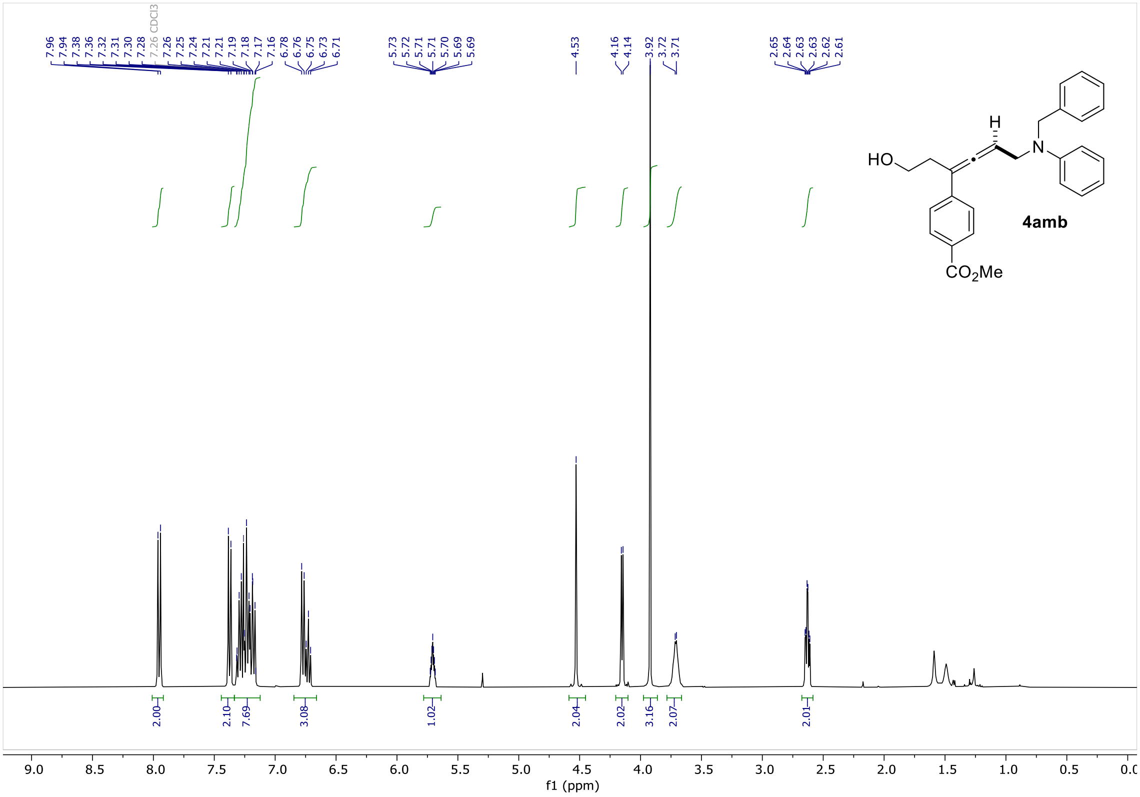

^{13}C NMR (101 MHz, $CDCl_3$)

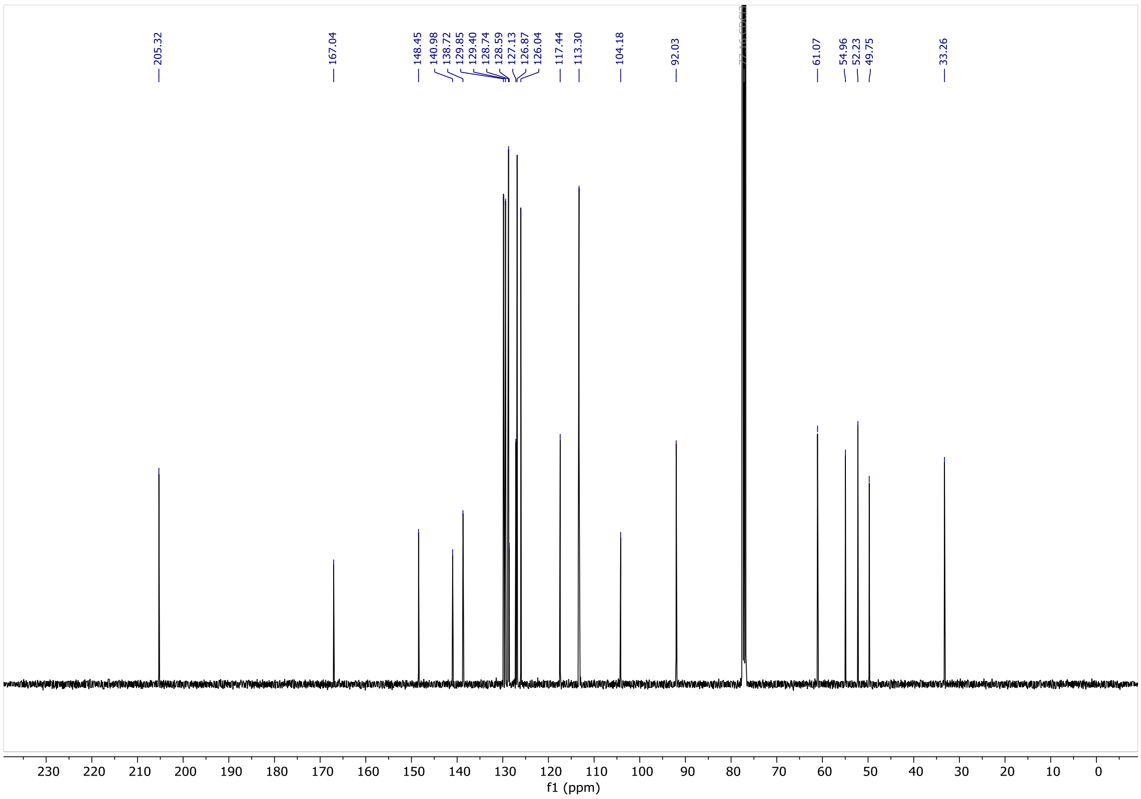

Methyl 4-(1-hydroxy-6-((4-methoxybenzyl)(phenyl)amino)hexa-3,4-dien-3-yl)benzoate (4anb)

^{1}H NMR (400 MHz, $CDCl_3$)

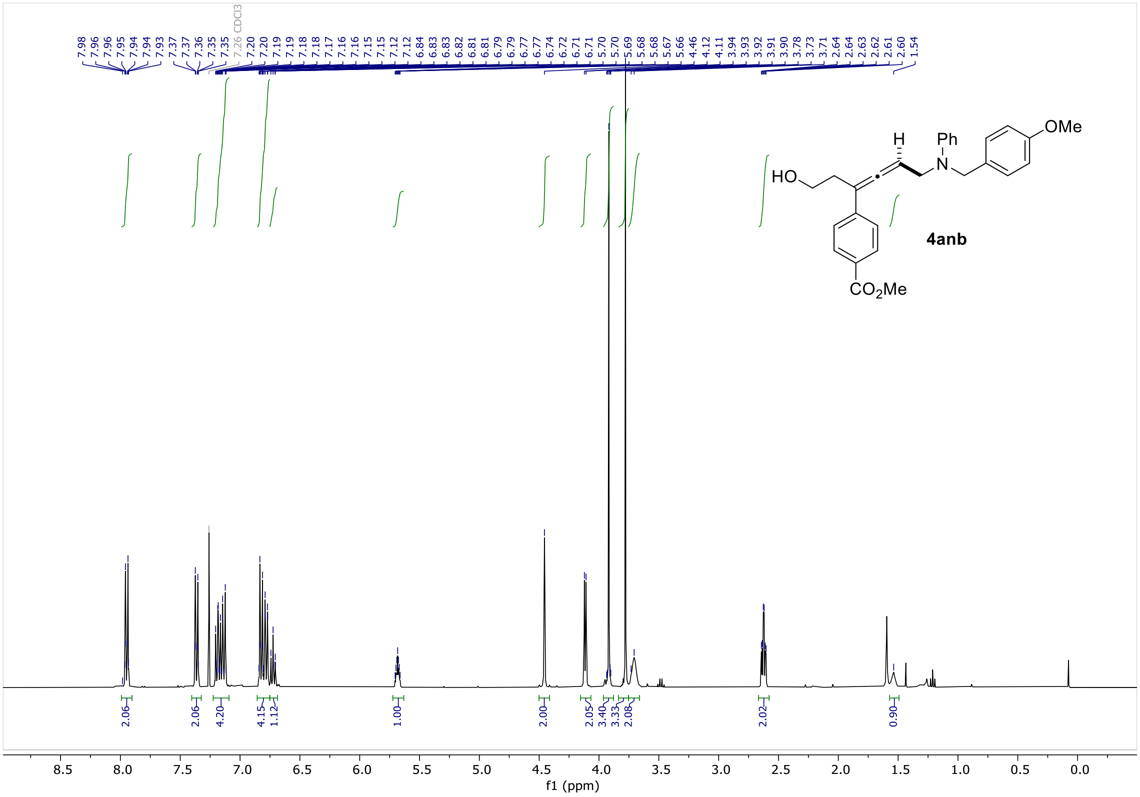

^{13}C NMR (101 MHz, $CDCl_3$)

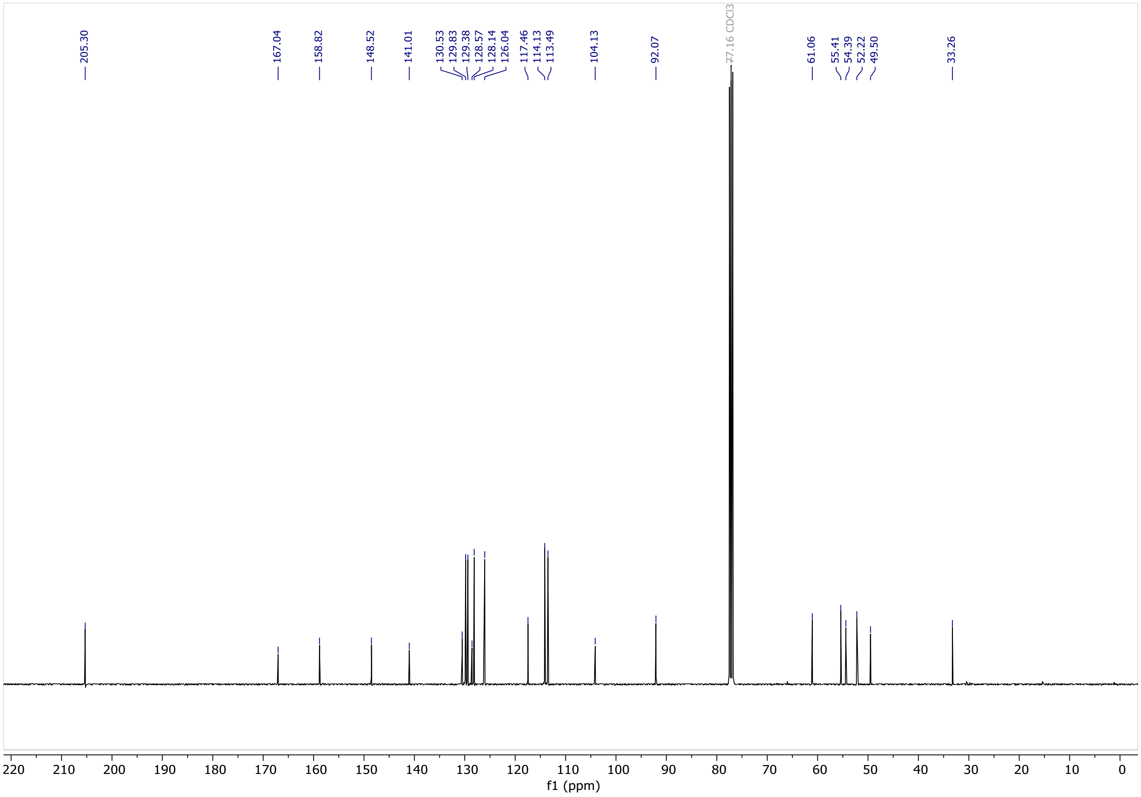

Methyl 4-(6-(3,4-dihydroquinolin-1(2*H*)-yl)-1-hydroxyhexa-3,4-dien-3-yl)benzoate (4aob)

^{1}H NMR (400 MHz, $CDCl_3$)

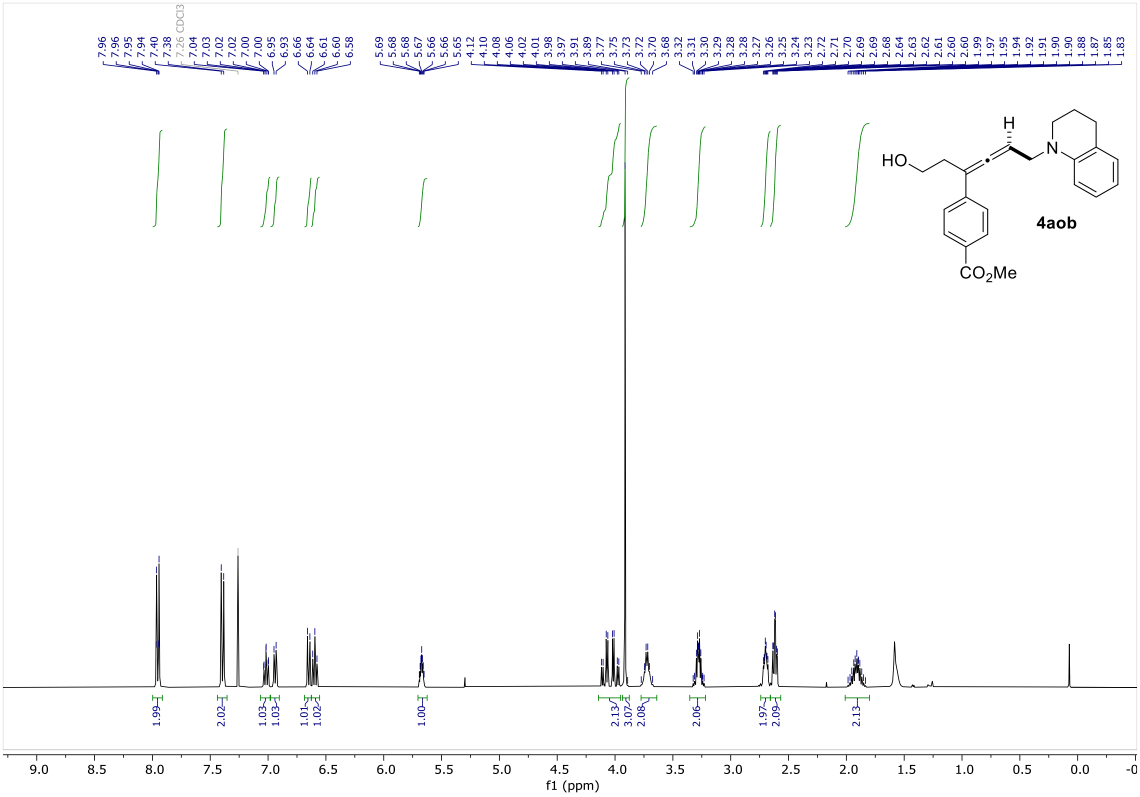

^{13}C NMR (101 MHz, $CDCl_3$)

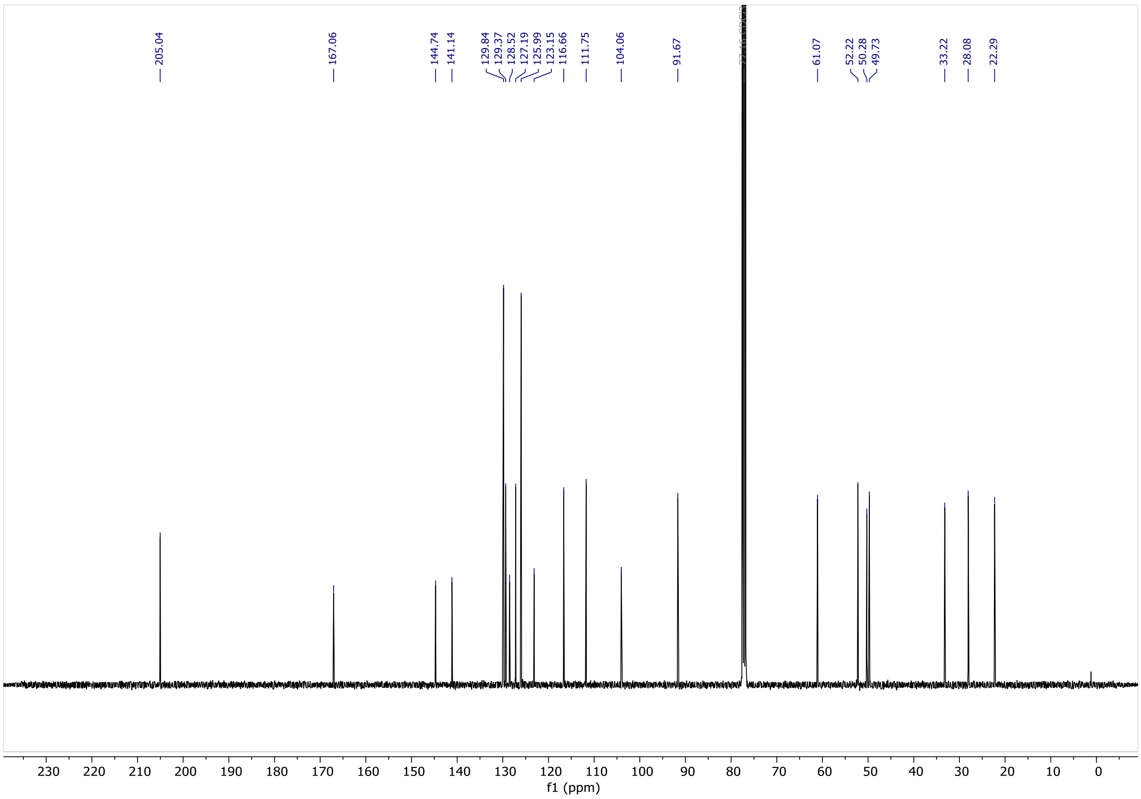

Methyl 4-(1-hydroxy-6-(indolin-1-yl)hexa-3,4-dien-3-yl)benzoate (4apb)

^{1}H NMR (400 MHz, $CDCl_3$)

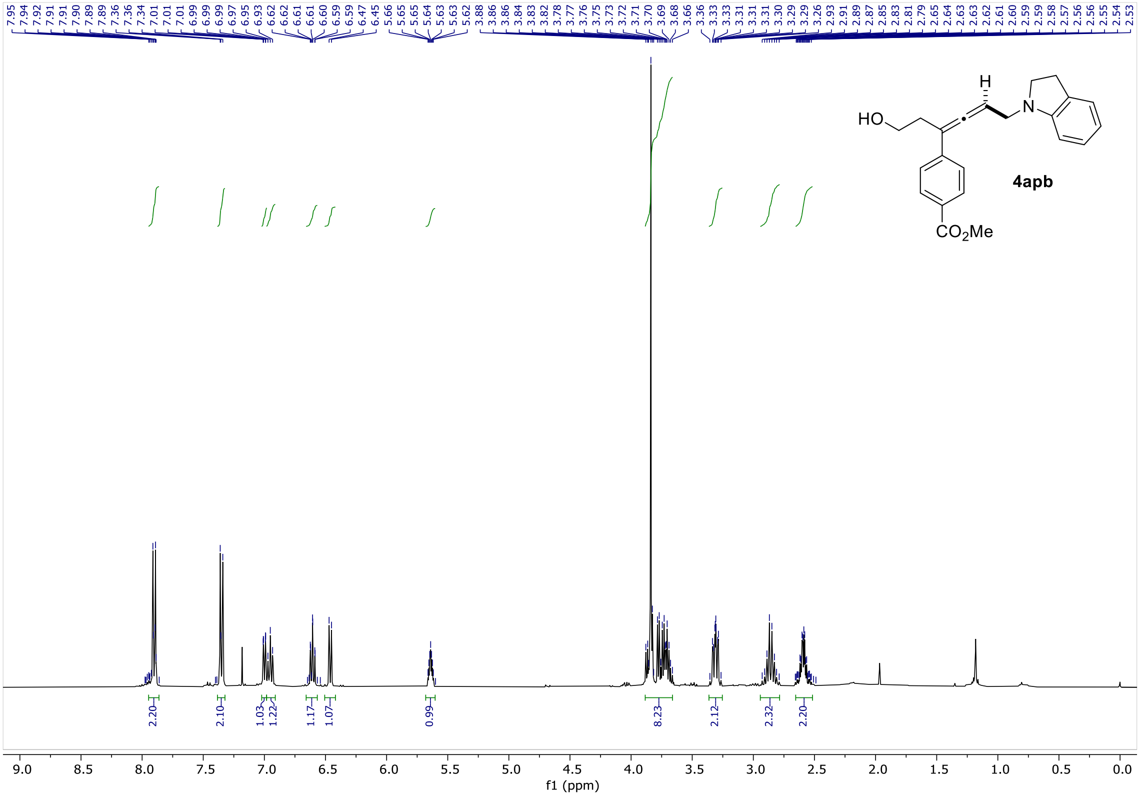

^{13}C NMR (101 MHz, $CDCl_3$)

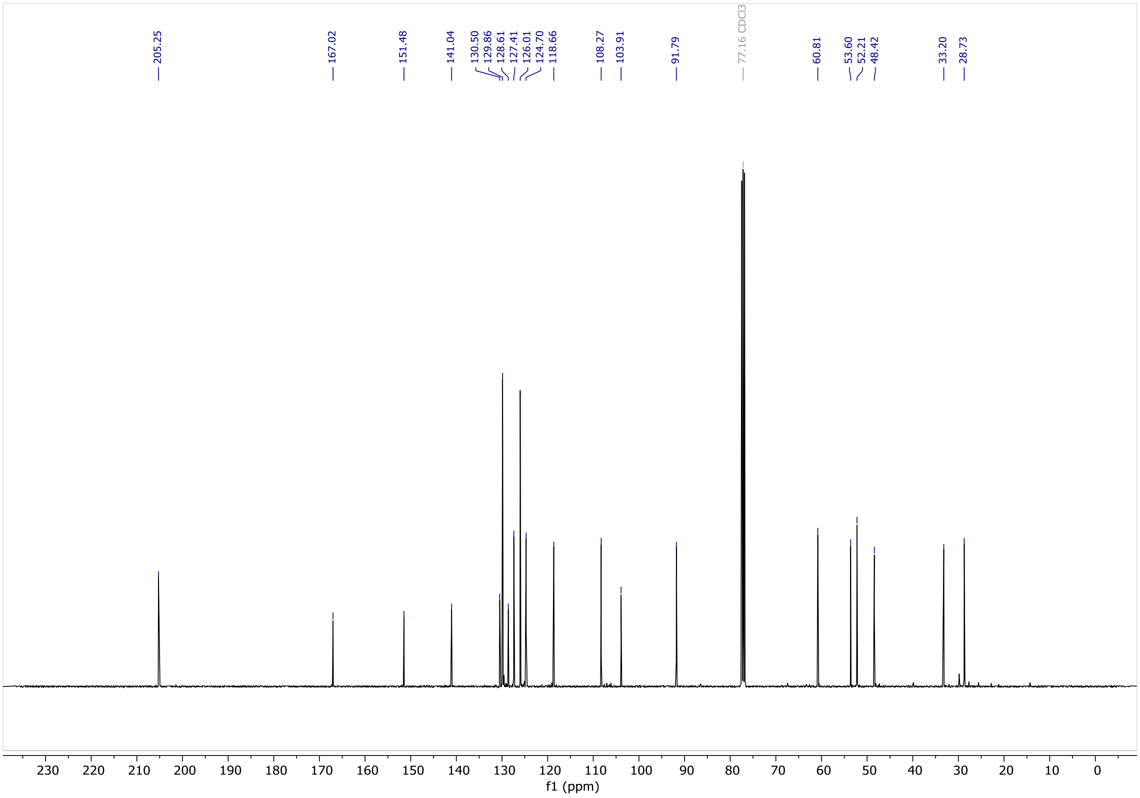

3-(*p*-Tolyl)-6-((4-(trifluoromethyl)phenyl)amino)hexa-3,4-dien-1-ol (4aqa)

^{1}H NMR (400 MHz, $CDCl_3$)

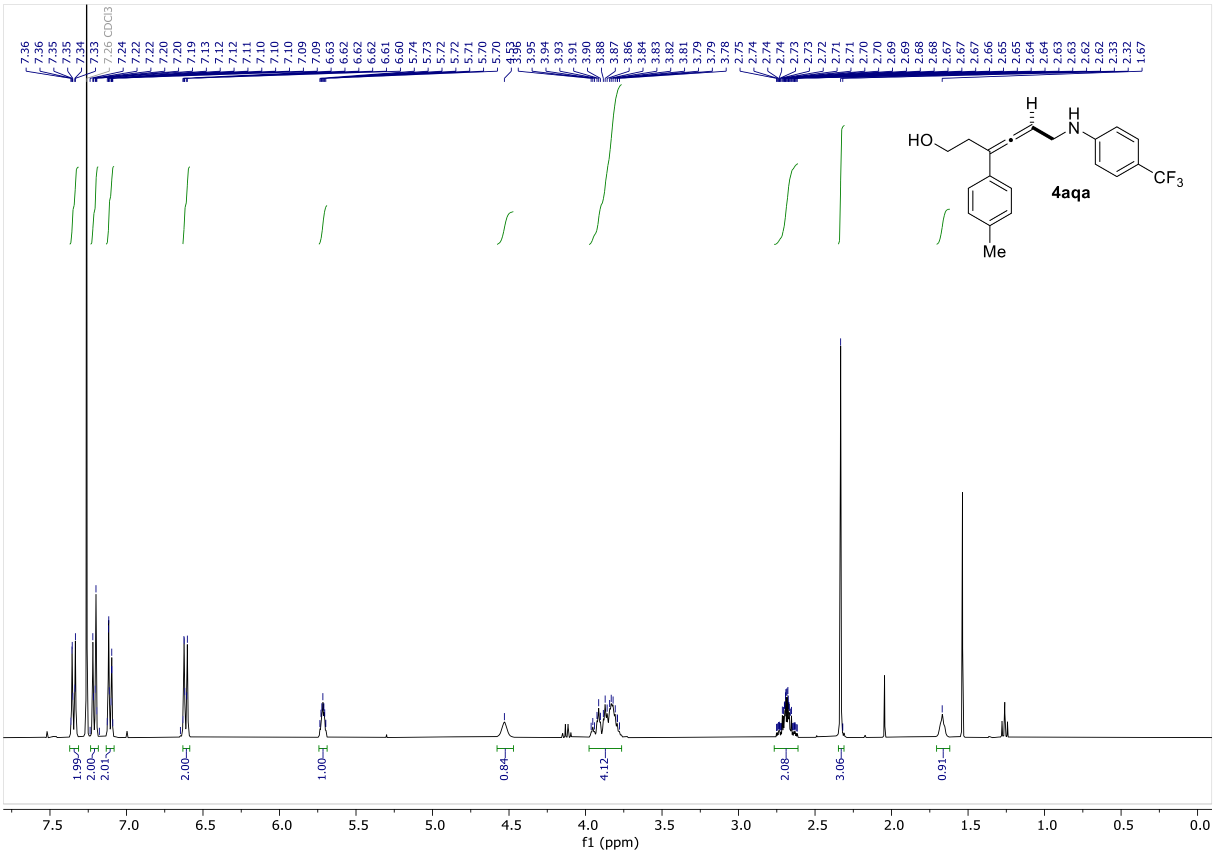

^{13}C NMR (101 MHz, $CDCl_3$)

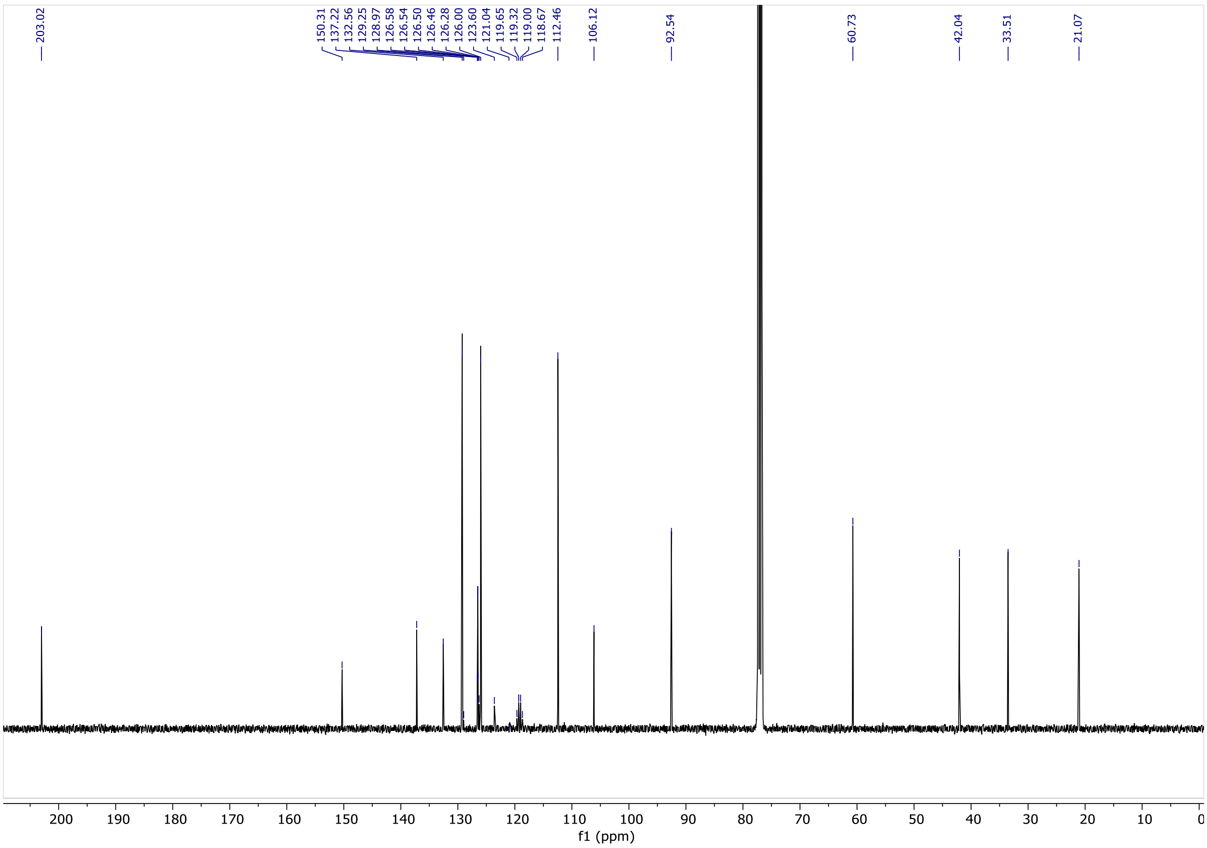

6-((4-Fluorophenyl)amino)-3-(*p*-tolyl)hexa-3,4-dien-1-ol (4ara)

^{1}H NMR (400 MHz, $CDCl_3$)

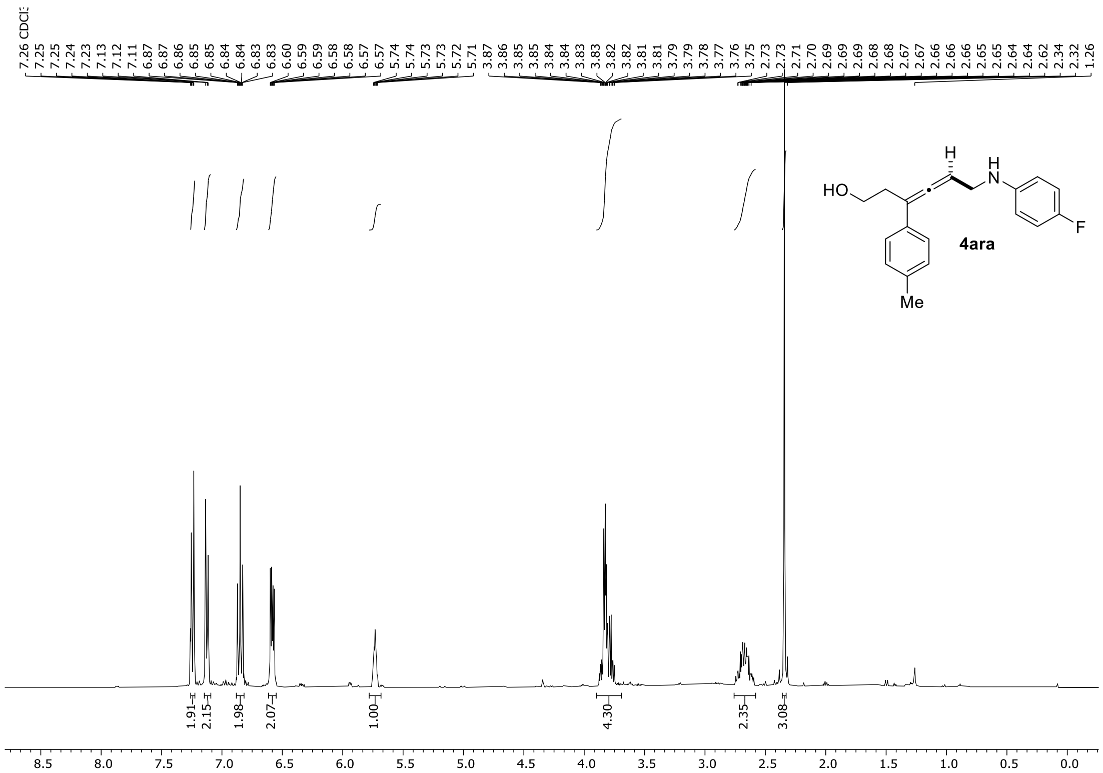

^{13}C NMR (101 MHz, $CDCl_3$)

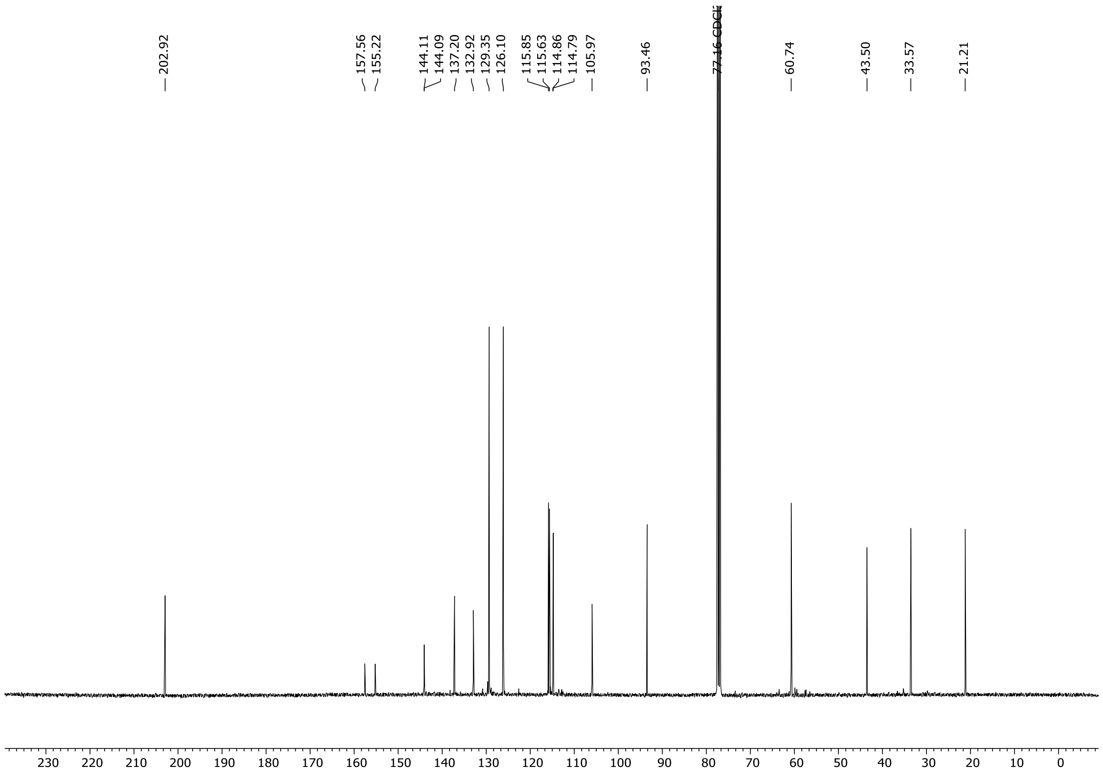

6-((3-Chlorophenyl)amino)-3-(*p*-tolyl)hexa-3,4-dien-1-ol (4asa)

^1H NMR (400 MHz, $CDCl_3$)

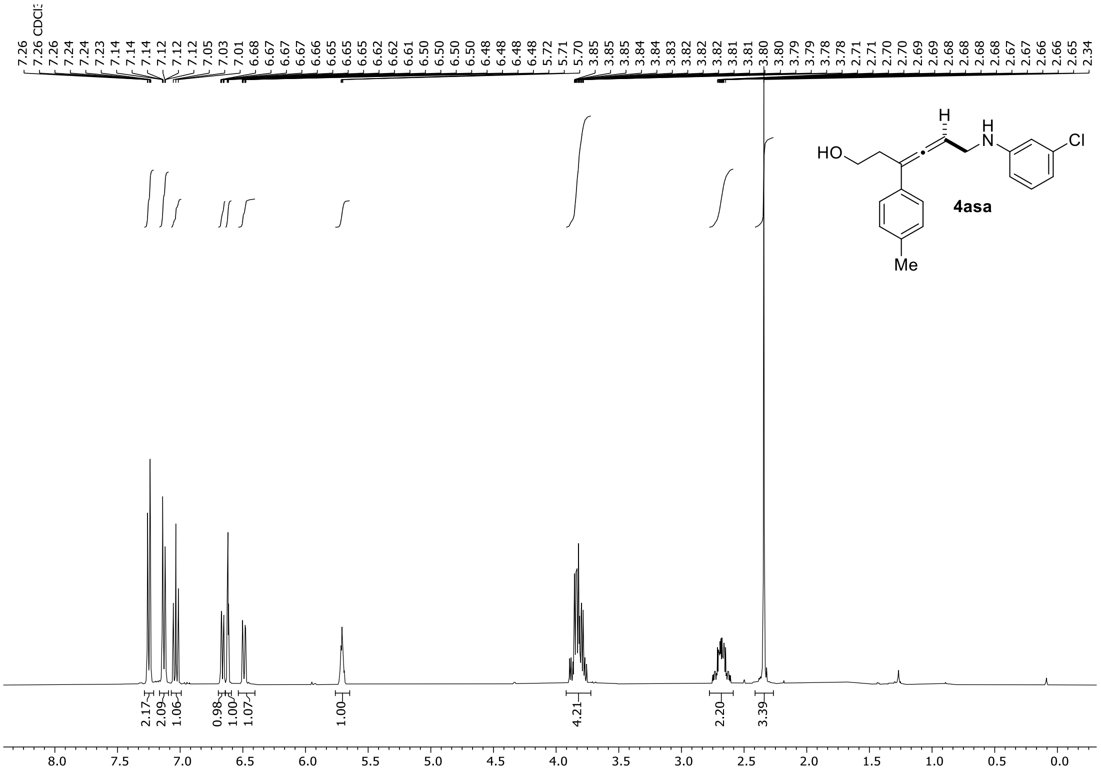

^{13}C NMR (101 MHz, $CDCl_3$)

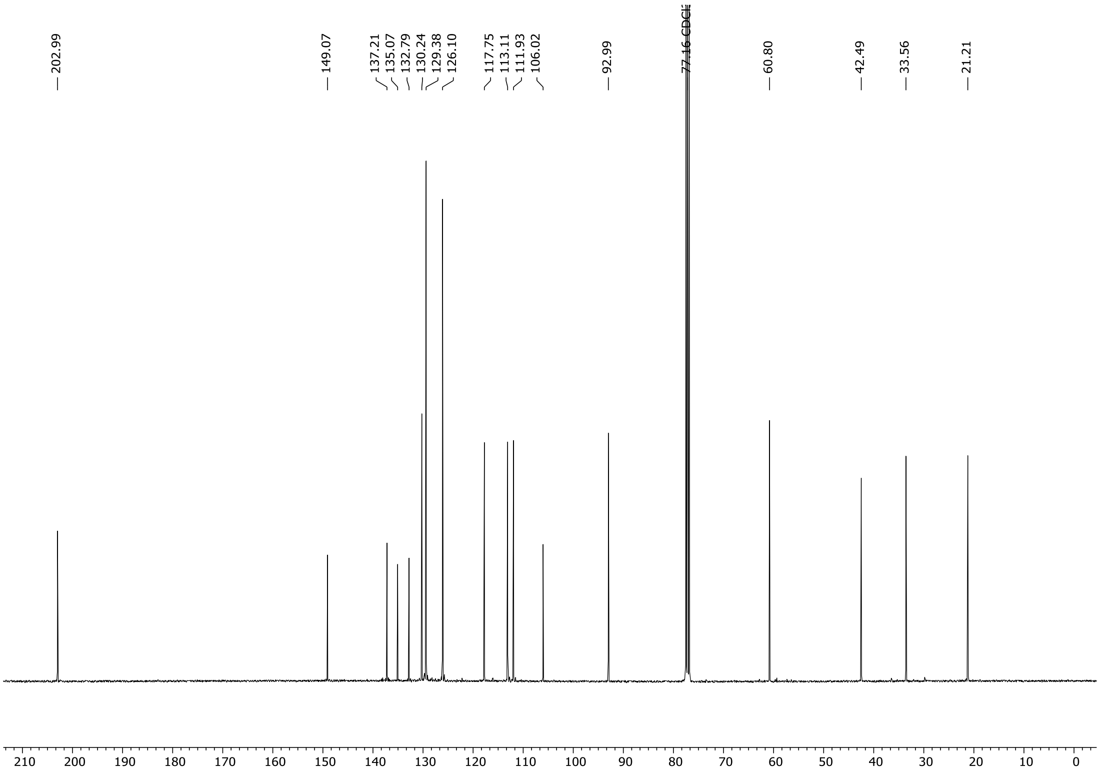

6-((3-Methoxyphenyl)amino)-3-(*p*-tolyl)hexa-3,4-dien-1-ol (4ata)

^{1}H NMR (400 MHz, $CDCl_3$)

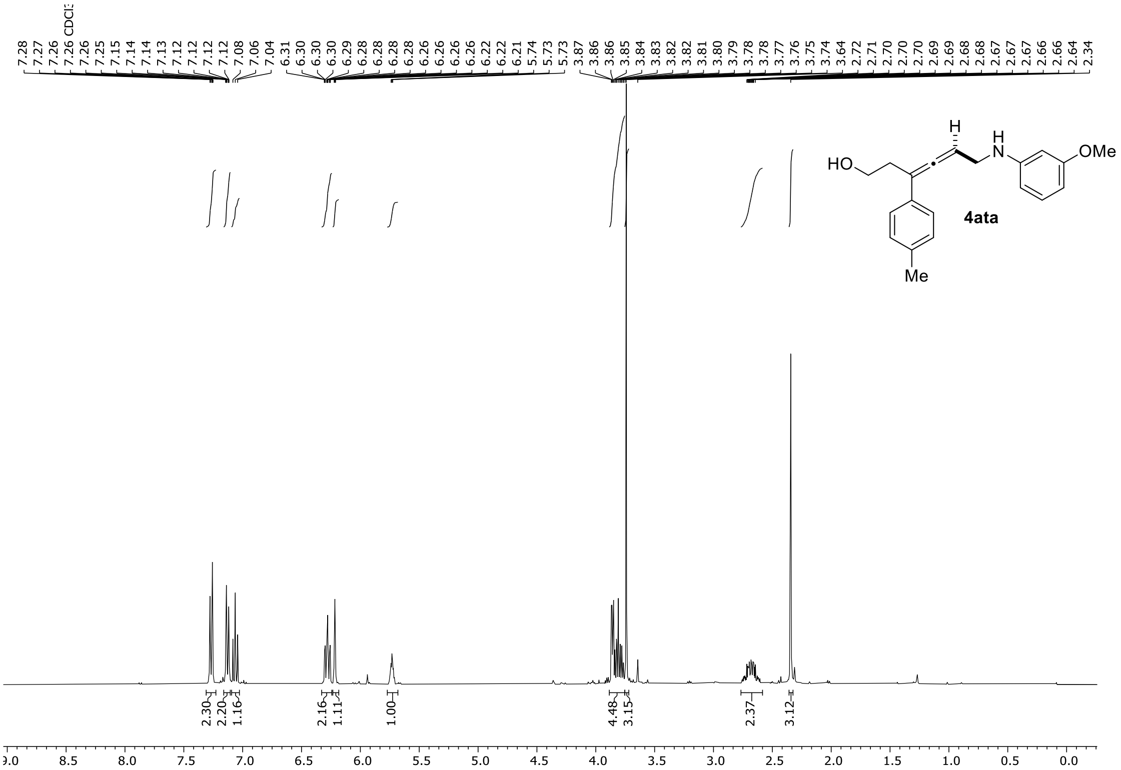

^{13}C NMR (101 MHz, $CDCl_3$)

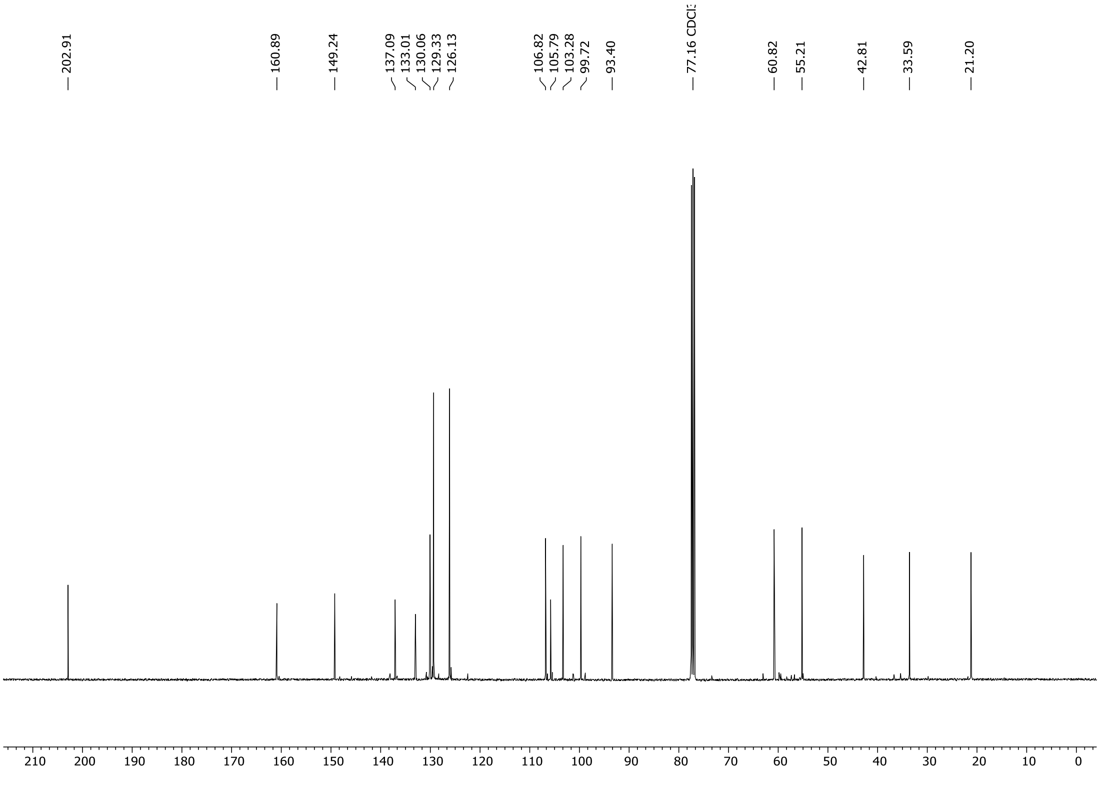

6-((2-Fluorophenyl)amino)-3-(*p*-tolyl)hexa-3,4-dien-1-ol (4aua)

^{1}H NMR (400 MHz, $CDCl_3$)

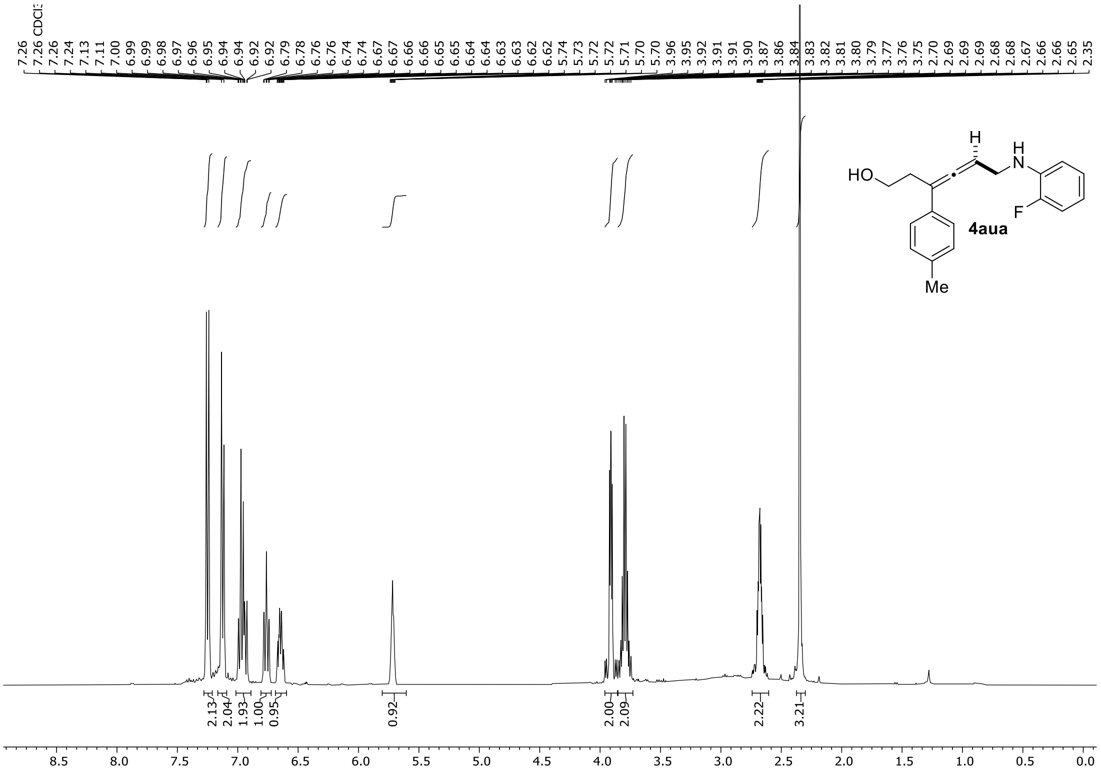

^{13}C NMR (101 MHz, $CDCl_3$)

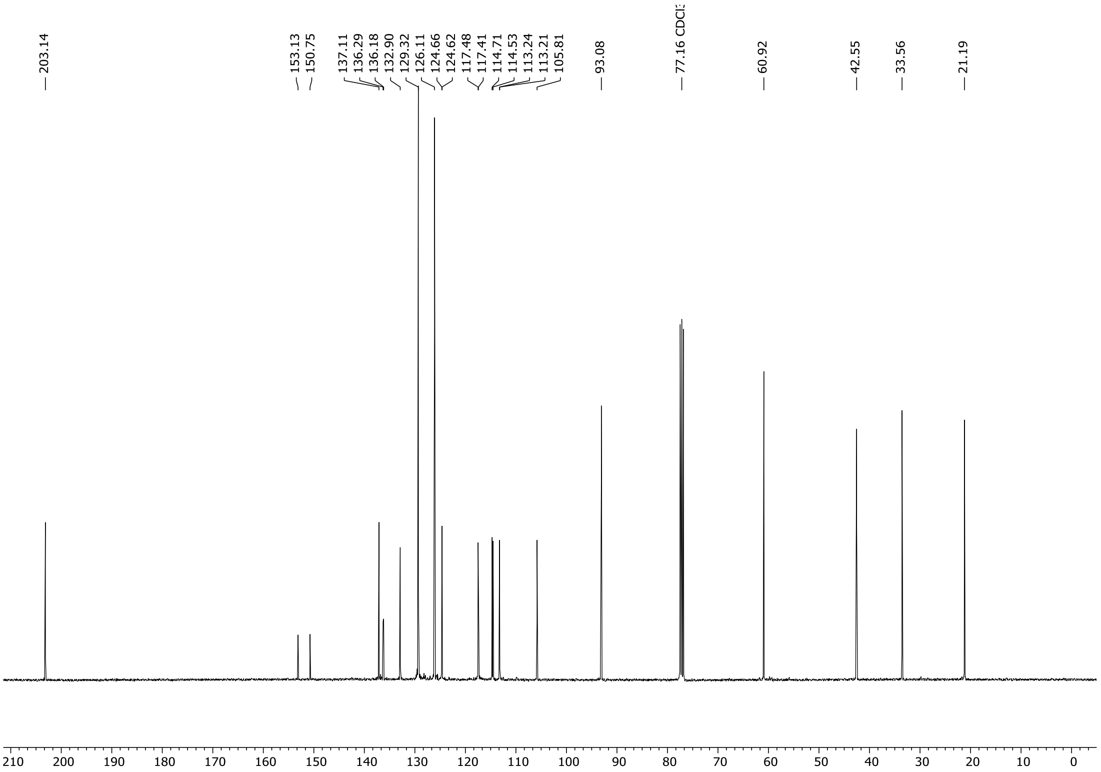

^{19}F NMR (376 MHz, $CDCl_3$)

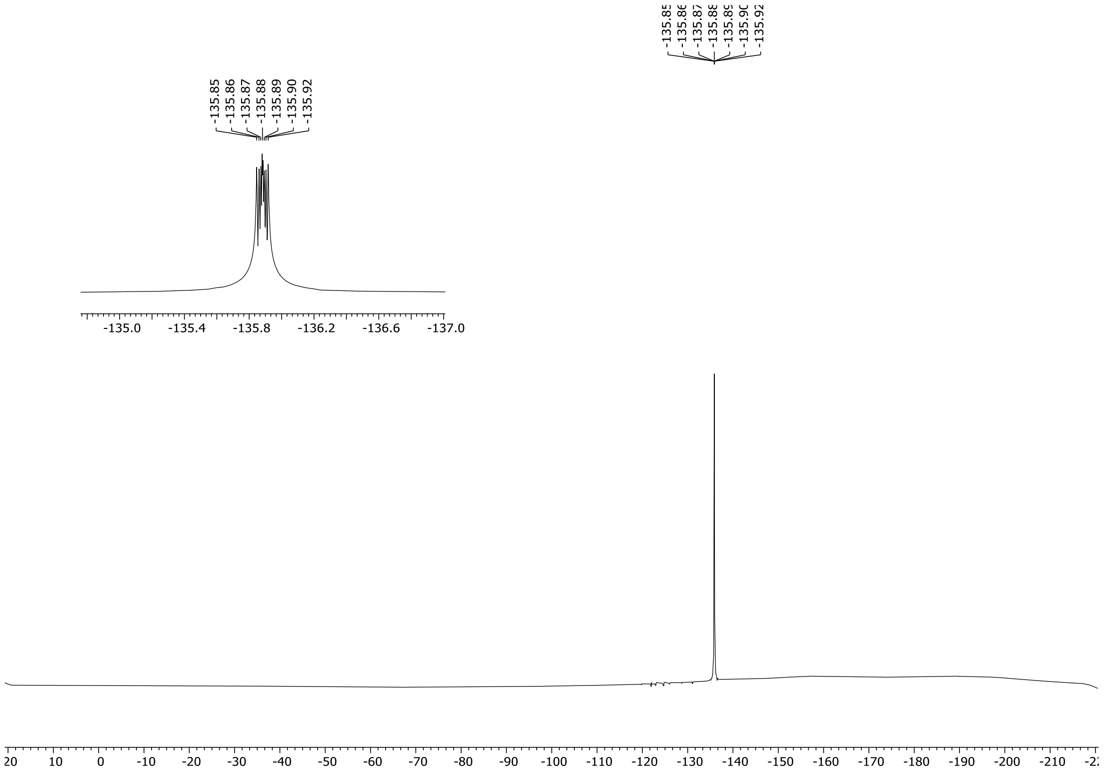

6-((2-(*tert*-Butyl)phenyl)amino)-3-(*p*-tolyl)hexa-3,4-dien-1-ol (4ava)

^{1}H NMR (400 MHz, $CDCl_3$)

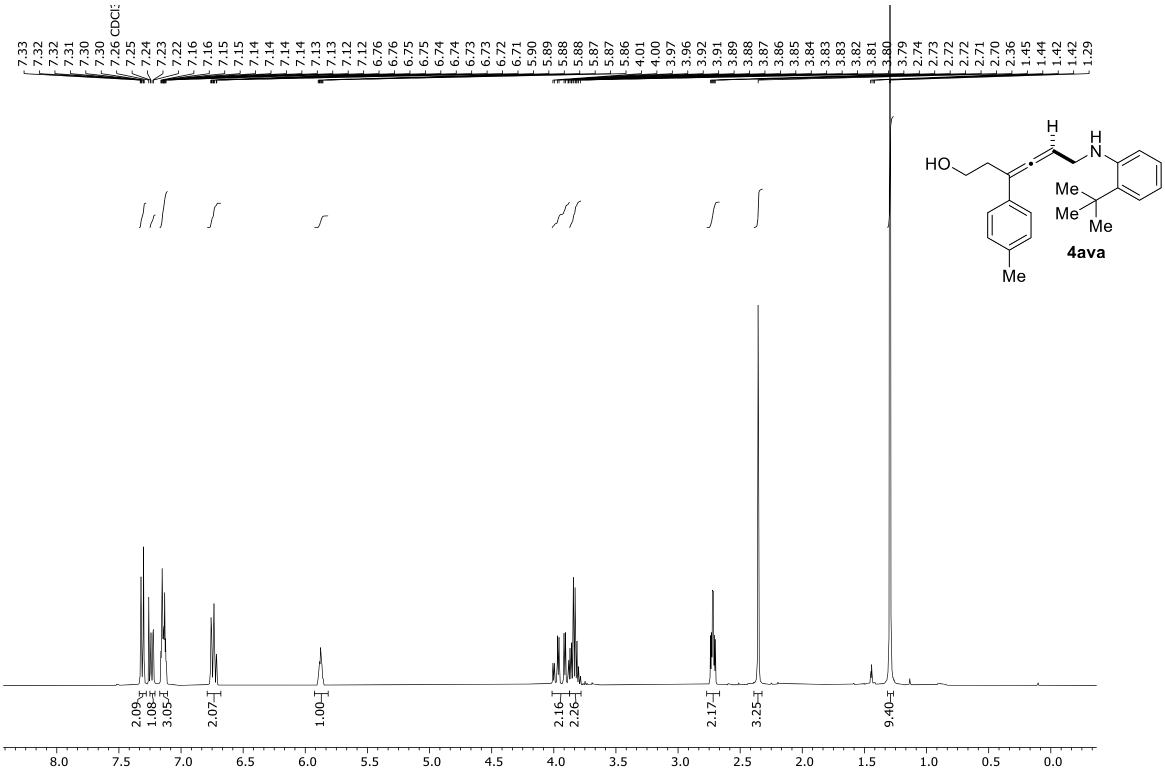

^{13}C NMR (101 MHz, $CDCl_3$)

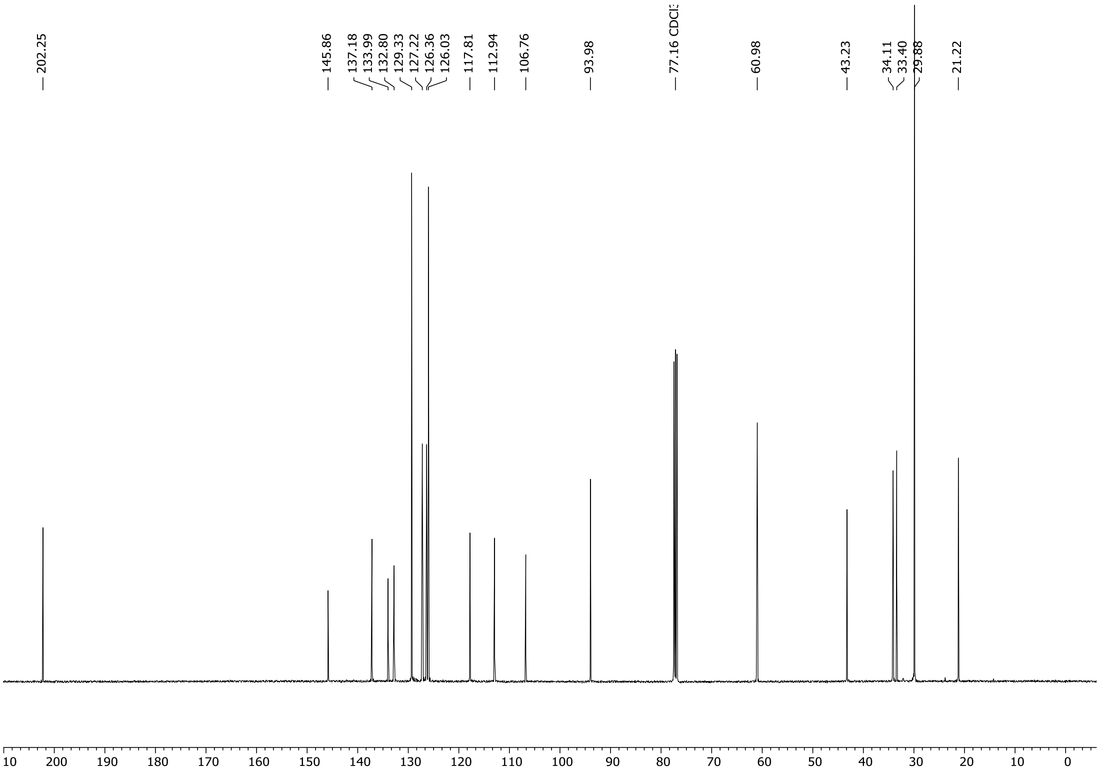

6-((3,5-Bis(trifluoromethyl)phenyl)amino)-3-(*p*-tolyl)hexa-3,4-dien-1-ol (4awa)

^{1}H NMR (400 MHz, CDCl$_3$)

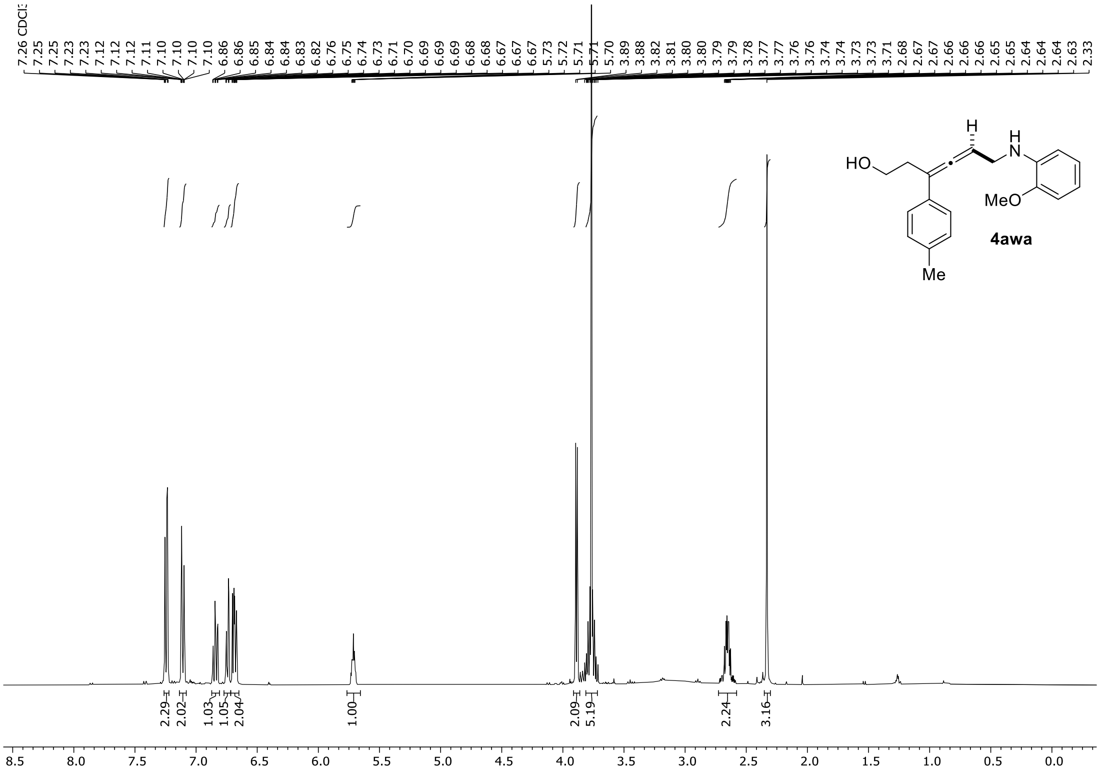

^{13}C NMR (101 MHz, CDCl$_3$)

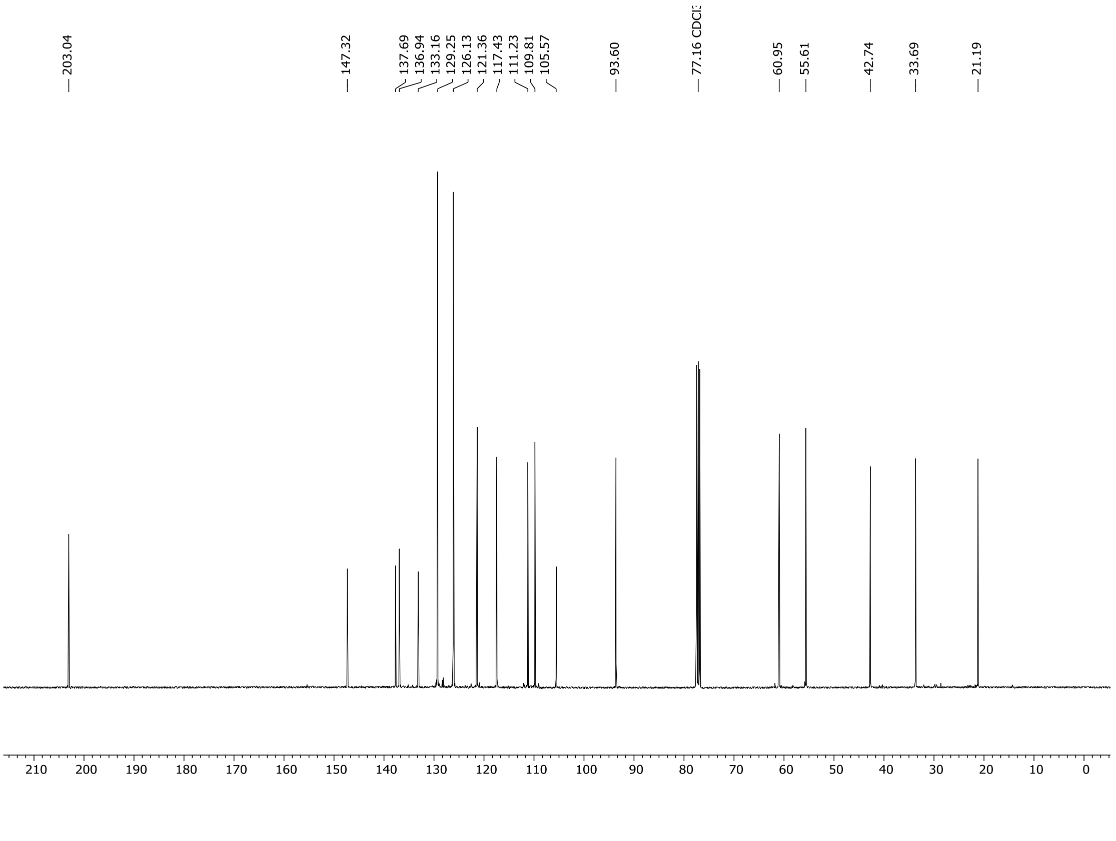

Methyl 4-(6-((2,6-dimethylphenyl)amino)-1-hydroxyhexa-3,4-dien-3-yl)benzoate (4axb)

^{1}H NMR (400 MHz, $CDCl_3$)

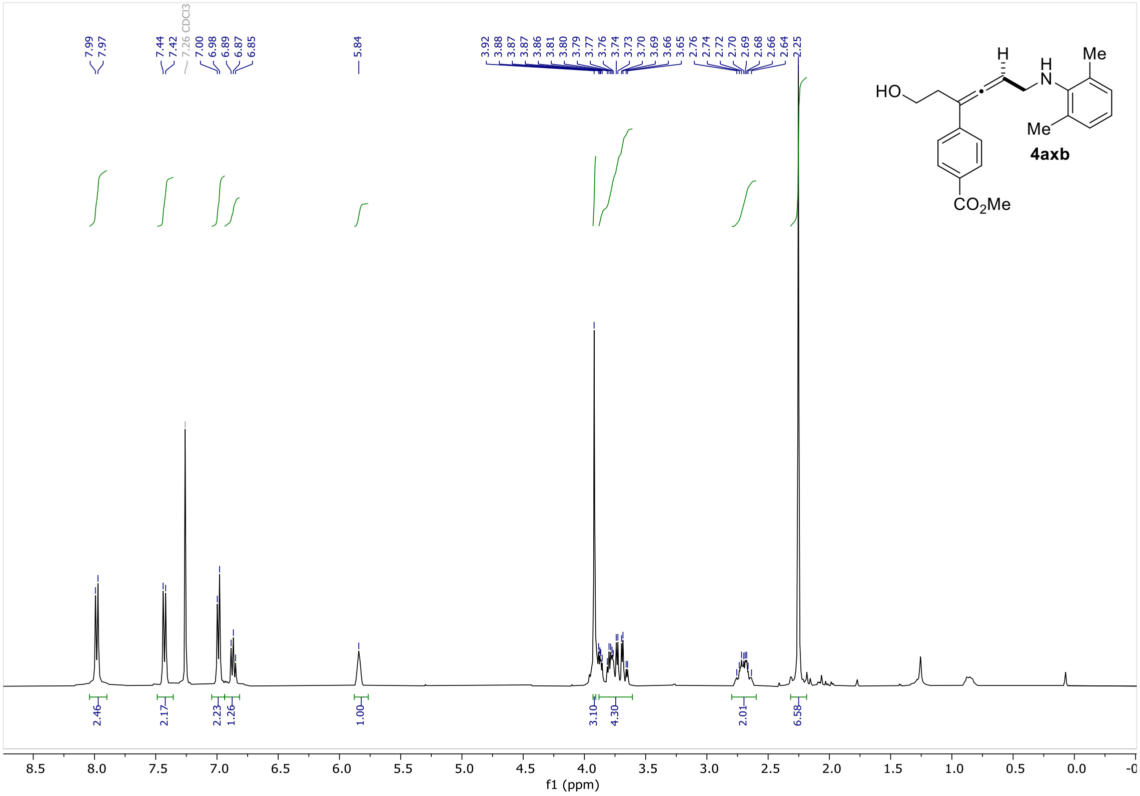

^{13}C NMR (101 MHz, $CDCl_3$)

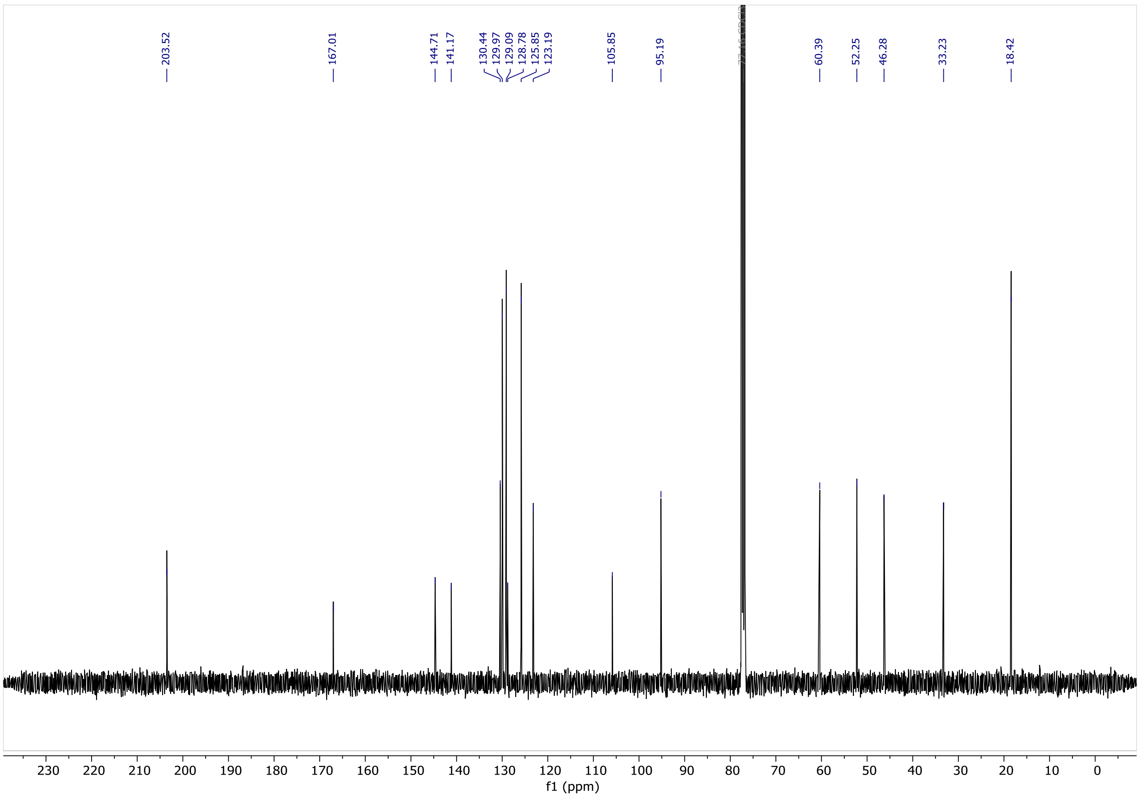

Methyl 4-(1-hydroxy-6-((2-methylnaphthalen-1-yl)amino)hexa-3,4-dien-3-yl)benzoate (4ayb)

^{1}H NMR (400 MHz, $CDCl_3$)

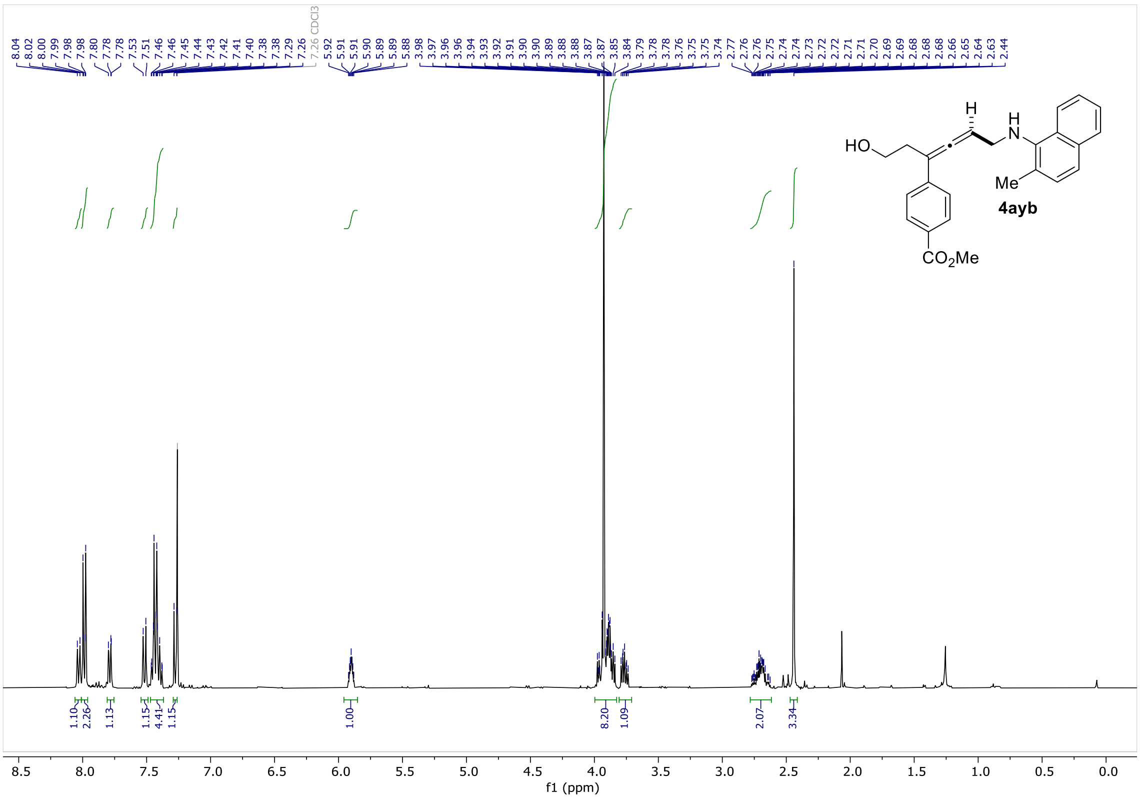

^{13}C NMR (101 MHz, $CDCl_3$)

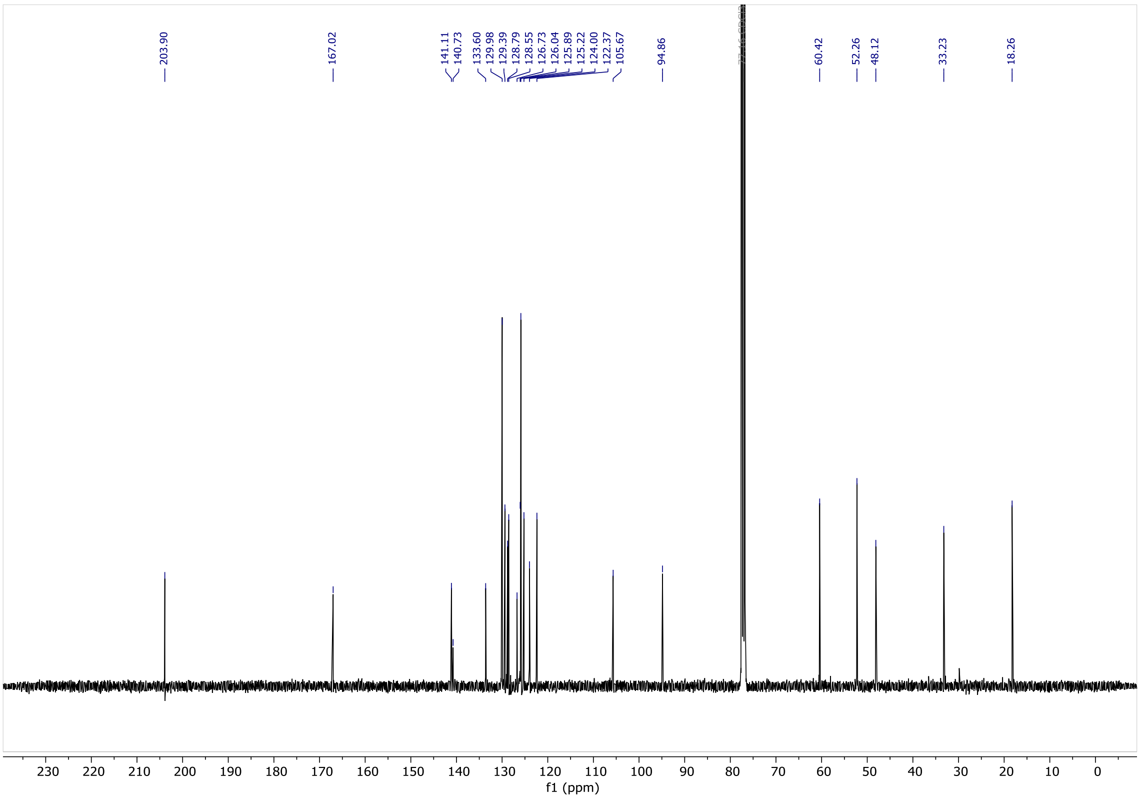

1-(4-(1-Hydroxy-6-((4-methoxyphenyl)(methyl)amino)hexa-3,4-dien-3-yl)phenyl)ethan-1-one (4aac)

^{1}H NMR (400 MHz, $CDCl_3$)

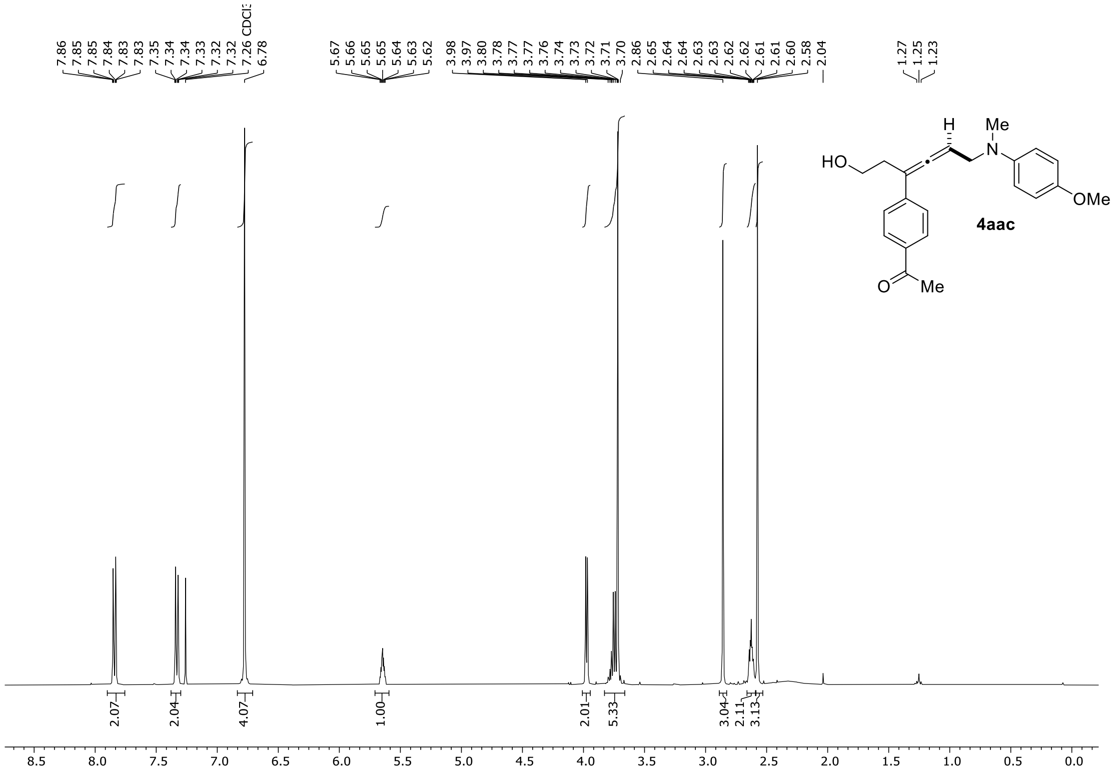

^{13}C NMR (101 MHz, $CDCl_3$)

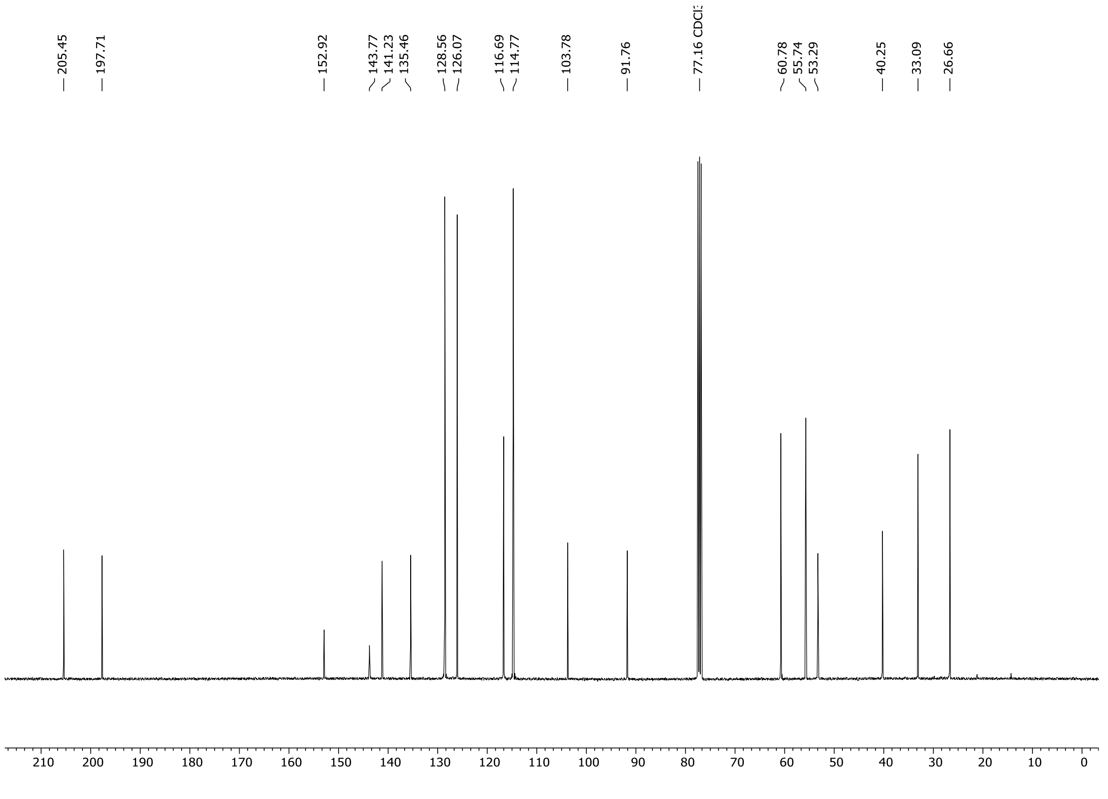

3-(4-(*tert*-Butyl)phenyl)-6-((4-methoxyphenyl)(methyl)amino)hexa-3,4-dien-1-ol (4aad)

^{1}H NMR (400 MHz, $CDCl_3$)

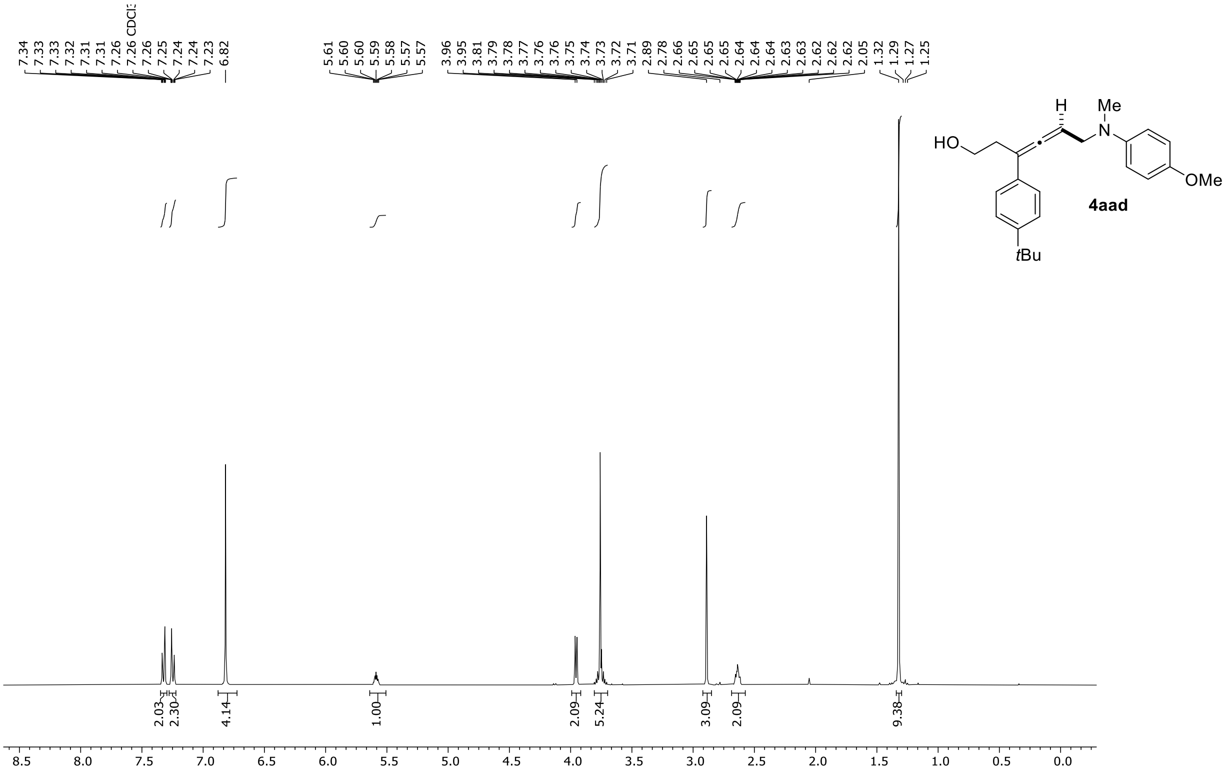

^{13}C NMR (101 MHz, $CDCl_3$)

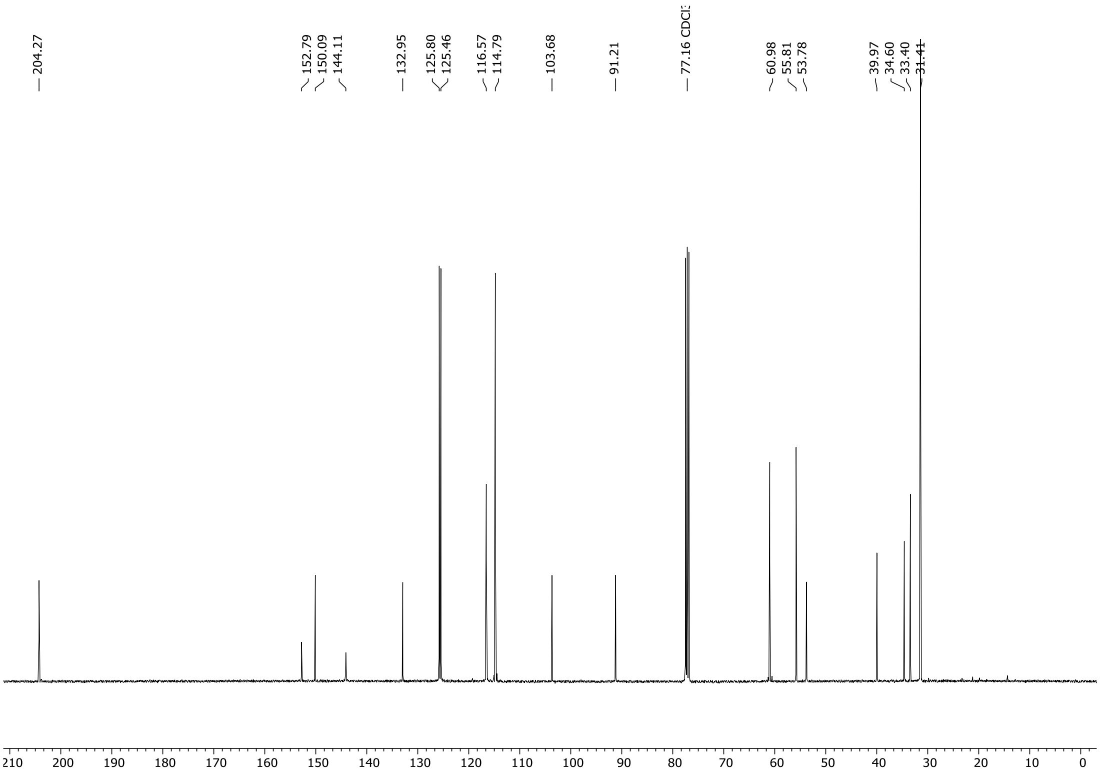

3-(4-Chlorophenyl)-6-((4-methoxyphenyl)(methyl)amino)hexa-3,4-dien-1-ol (4aae)

^{1}H NMR (400 MHz, $CDCl_3$)

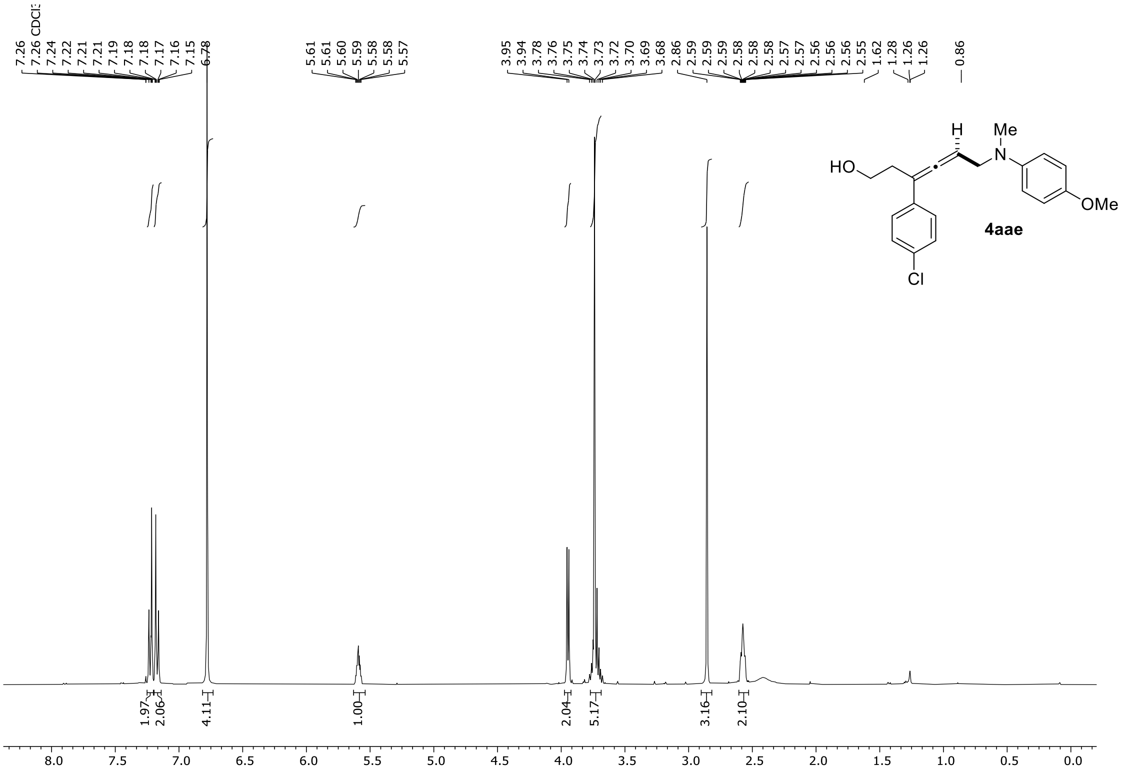

^{13}C NMR (101 MHz, $CDCl_3$)

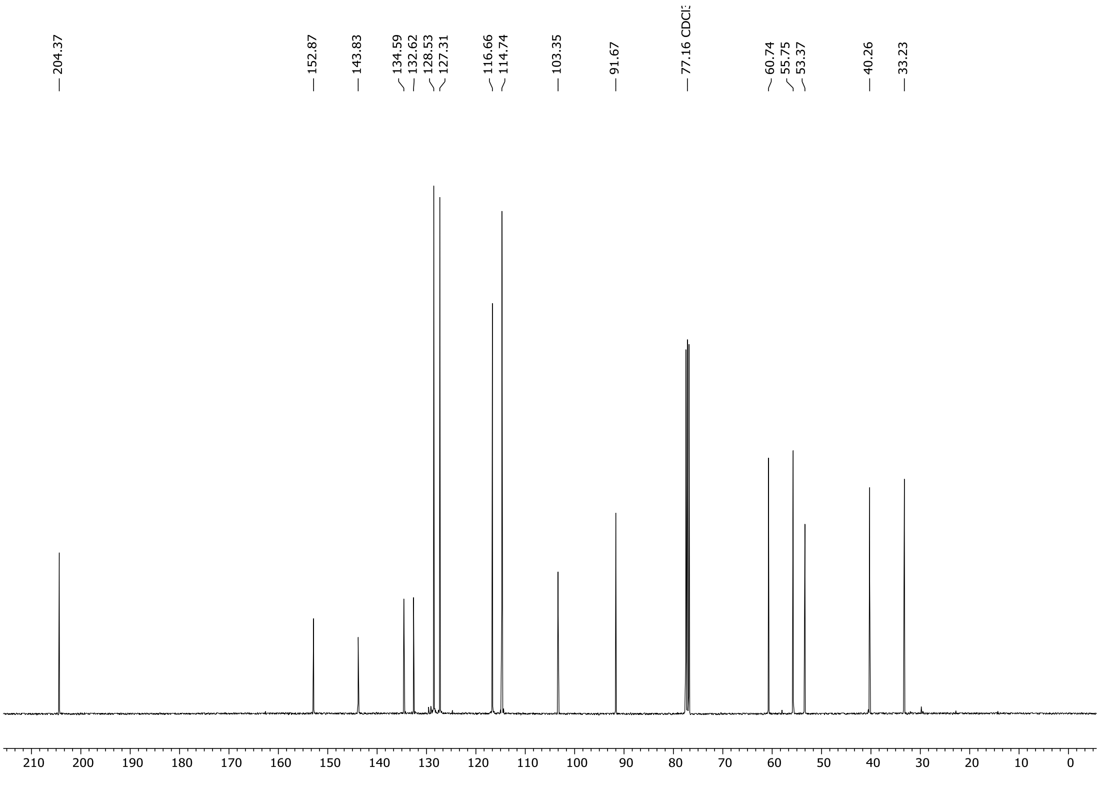

3-(4-Fluorophenyl)-6-((4-methoxyphenyl)(methyl)amino)hexa-3,4-dien-1-ol (4aaf)

^{1}H NMR (400 MHz, $CDCl_3$)

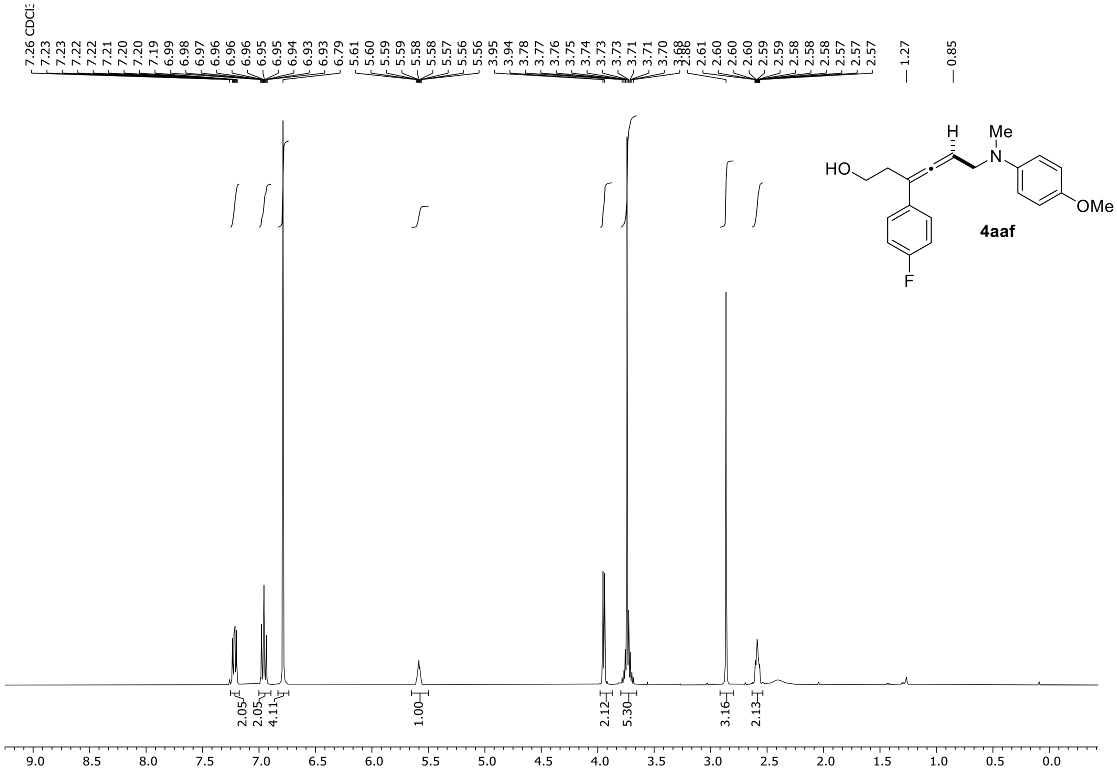

^{13}C NMR (101 MHz, $CDCl_3$)

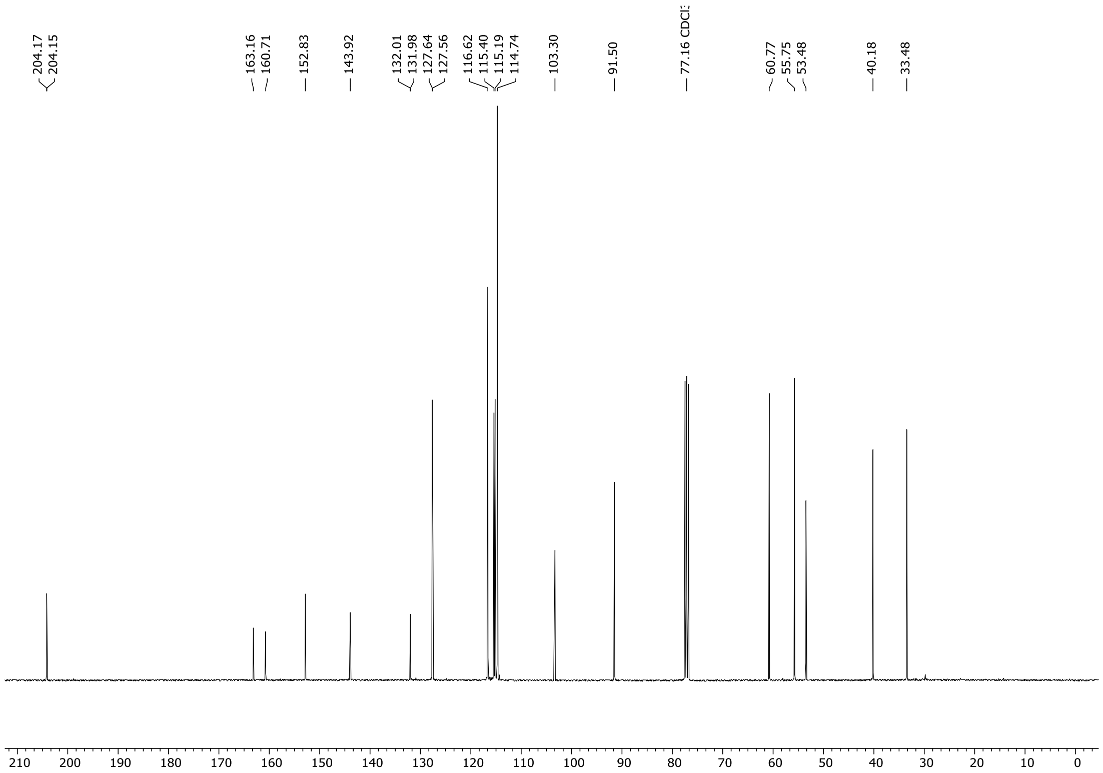

^{19}F NMR (376 MHz, $CDCl_3$)

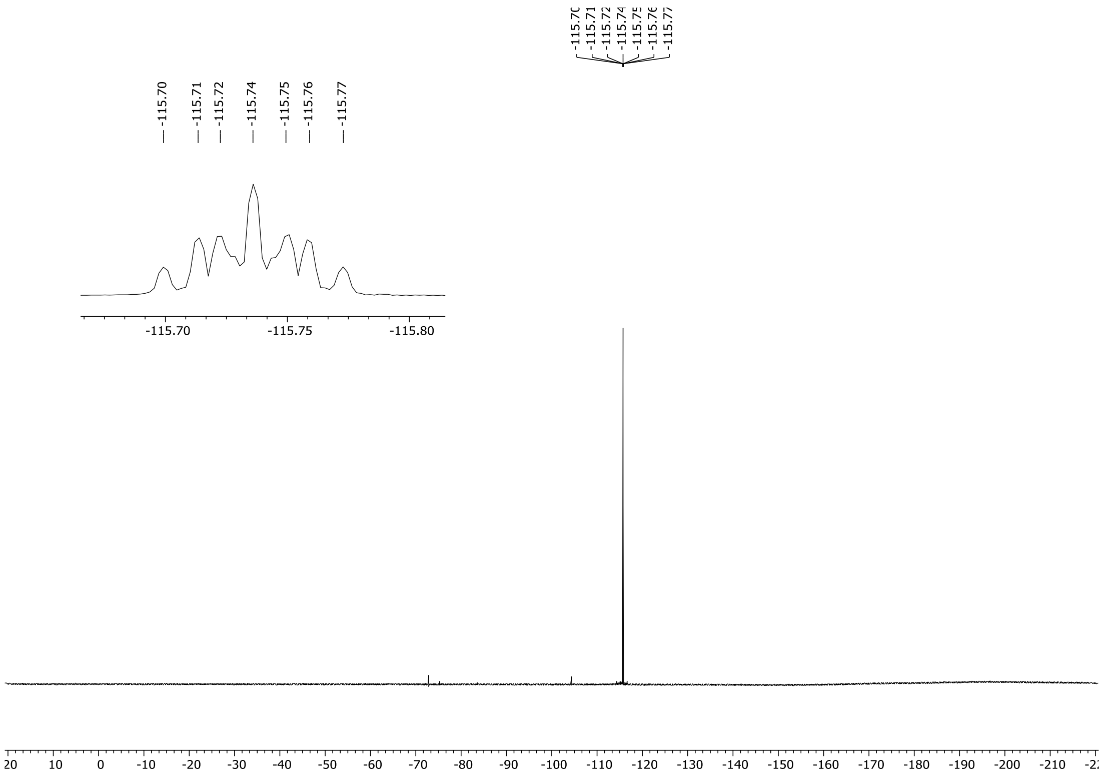

3-(4-Methoxyphenyl)-6-((4-methoxyphenyl)(methyl)amino)hexa-3,4-dien-1-ol (4aag)

^{1}H NMR (400 MHz, $CDCl_3$)

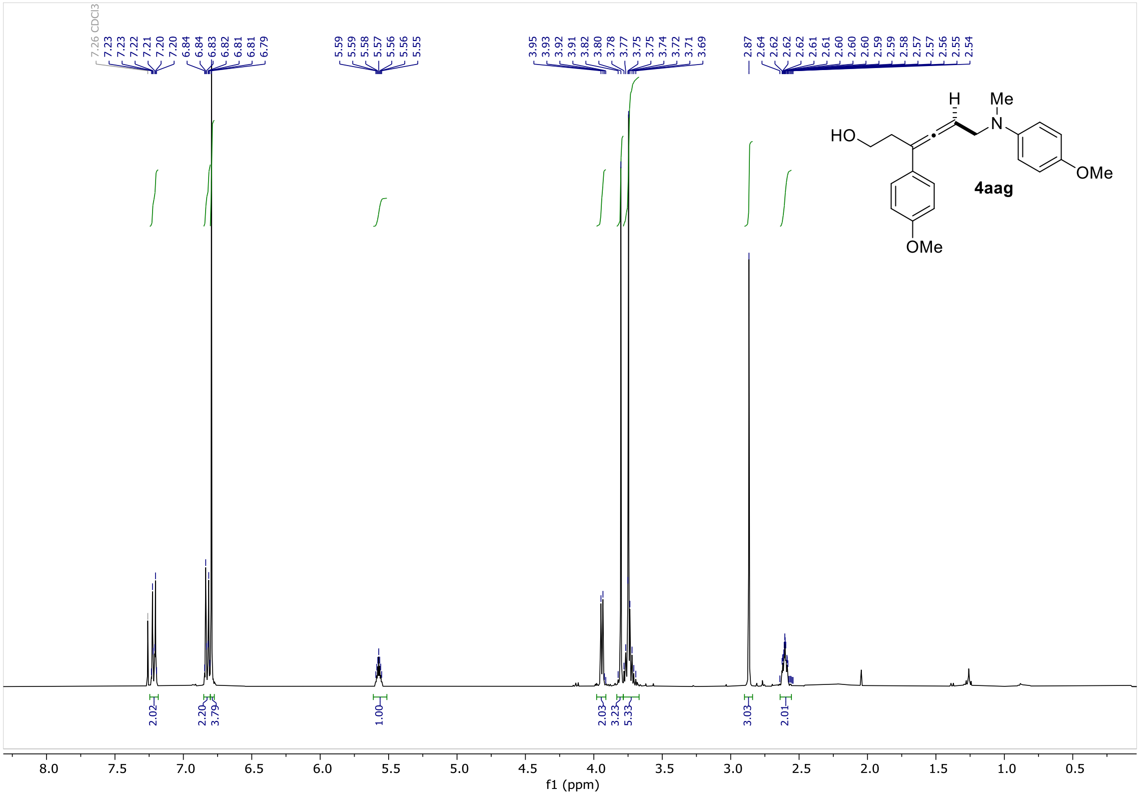

^{13}C NMR (101 MHz, $CDCl_3$)

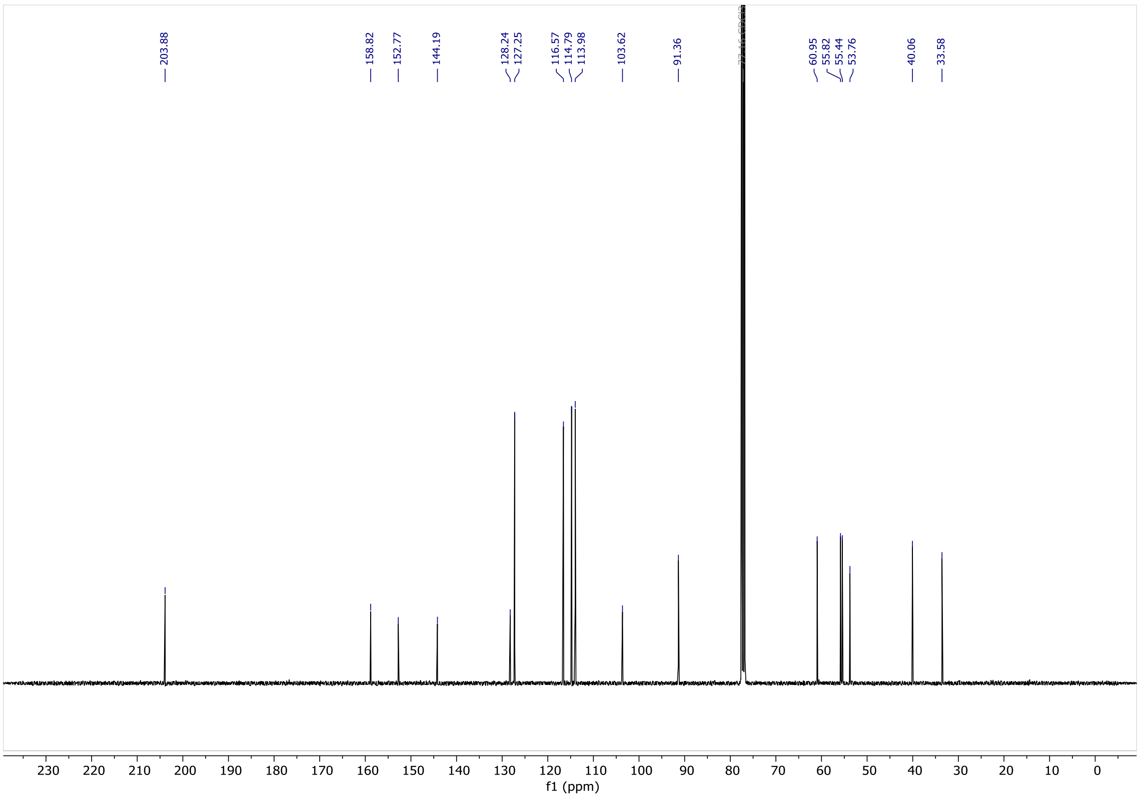

6-((4-Methoxyphenyl)(methyl)amino)-3-(4-(trifluoromethyl)phenyl)hexa-3,4-dien-1-ol (4aah)

^{1}H NMR (400 MHz, $CDCl_3$)

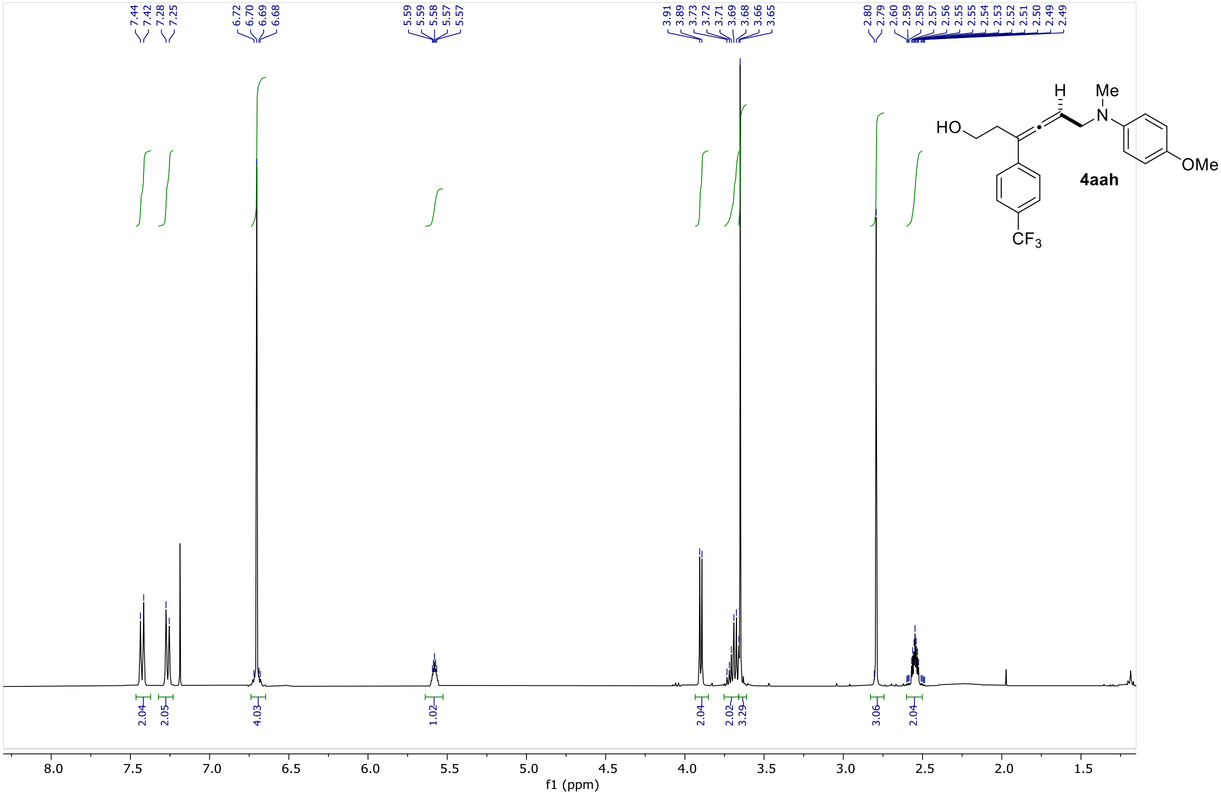

^{13}C NMR (101 MHz, $CDCl_3$)

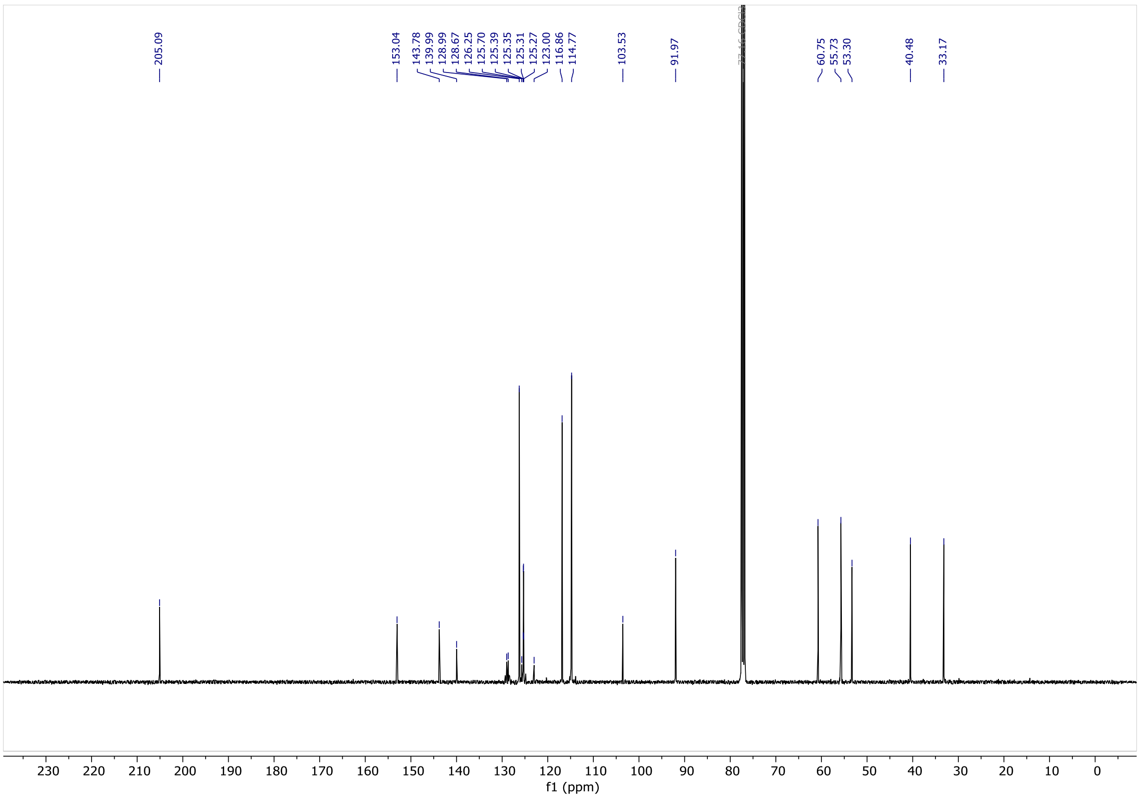

4-(1-Hydroxy-6-((4-methoxyphenyl)(methyl)amino)hexa-3,4-dien-3-yl)benzaldehyde (4aai)

^{1}H NMR (400 MHz, $CDCl_3$)

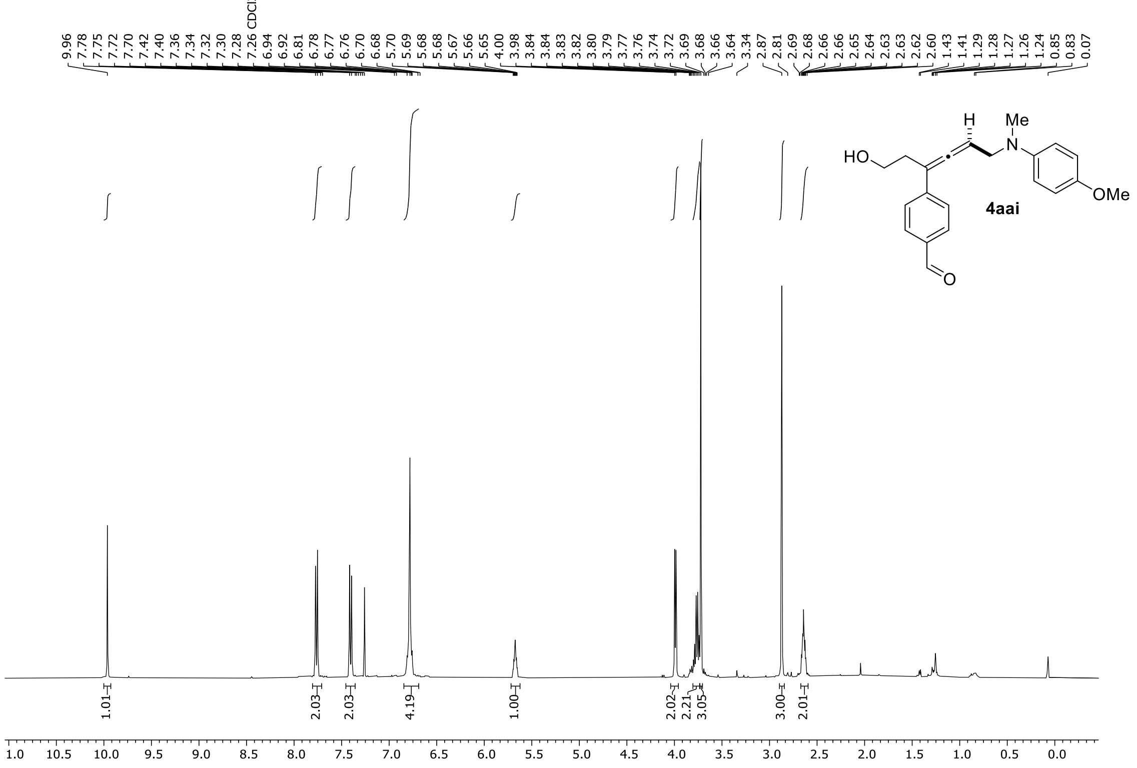

^{13}C NMR (101 MHz, $CDCl_3$)

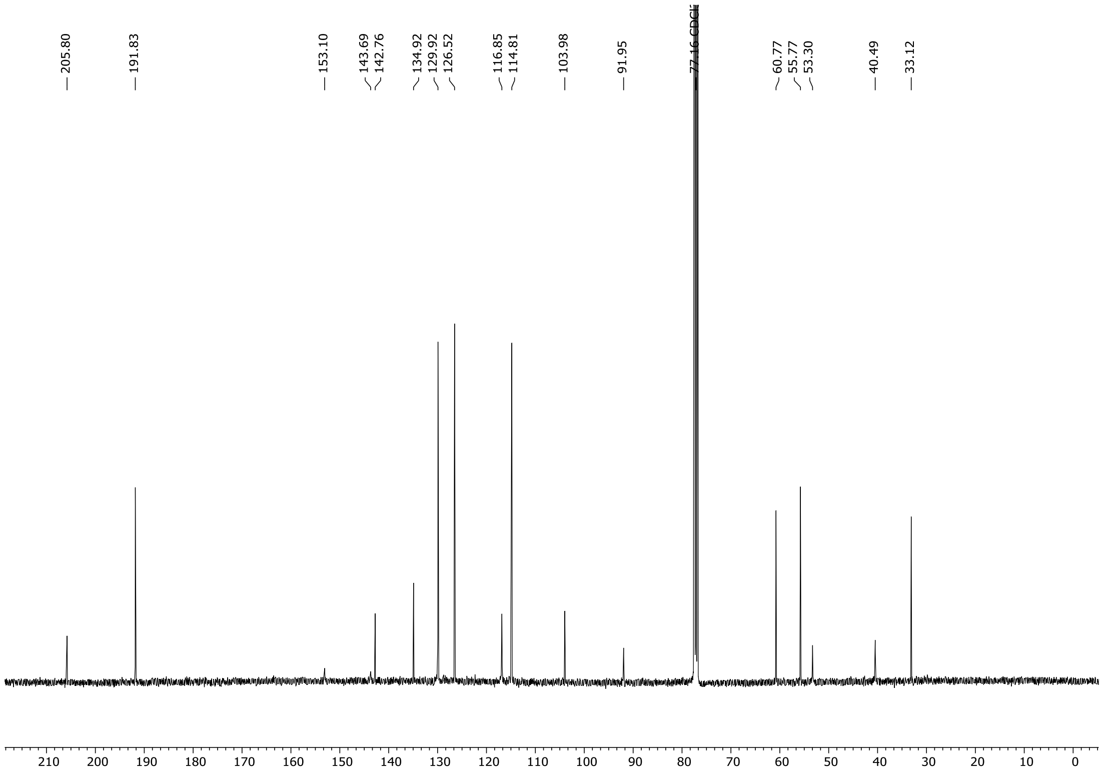

Methyl 4-(1-hydroxy-6-((4-methoxyphenyl)(methyl)amino)hexa-3,4-dien-3-yl)benzoate (4aaj)

^{1}H NMR (400 MHz, $CDCl_3$)

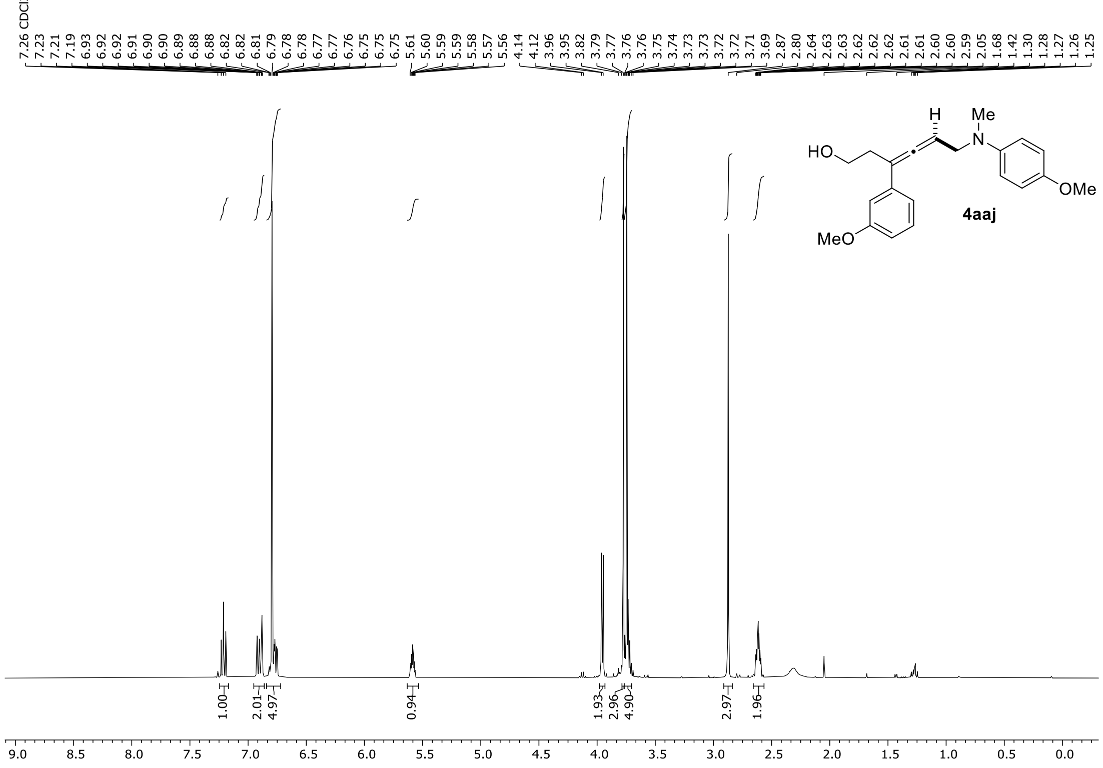

^{13}C NMR (101 MHz, $CDCl_3$)

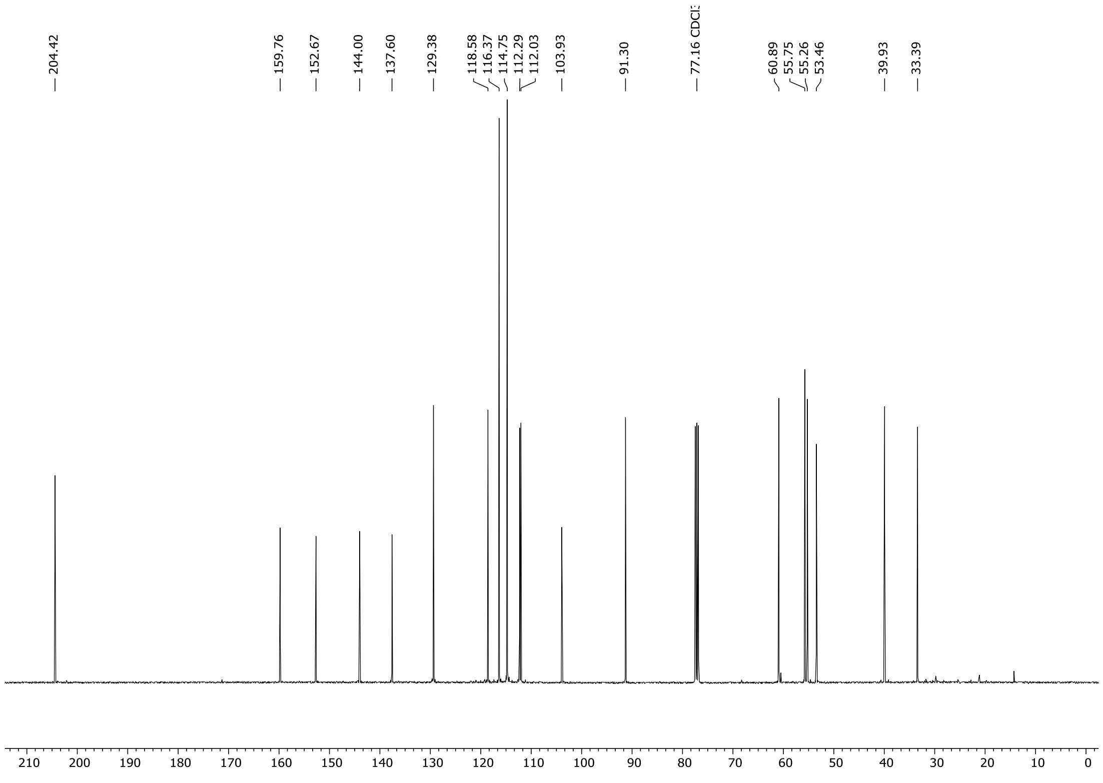

6-((4-Methoxyphenyl)(methyl)amino)-3-(naphthalen-2-yl)hexa-3,4-dien-1-ol (4aak)

^{1}H NMR (400 MHz, $CDCl_3$)

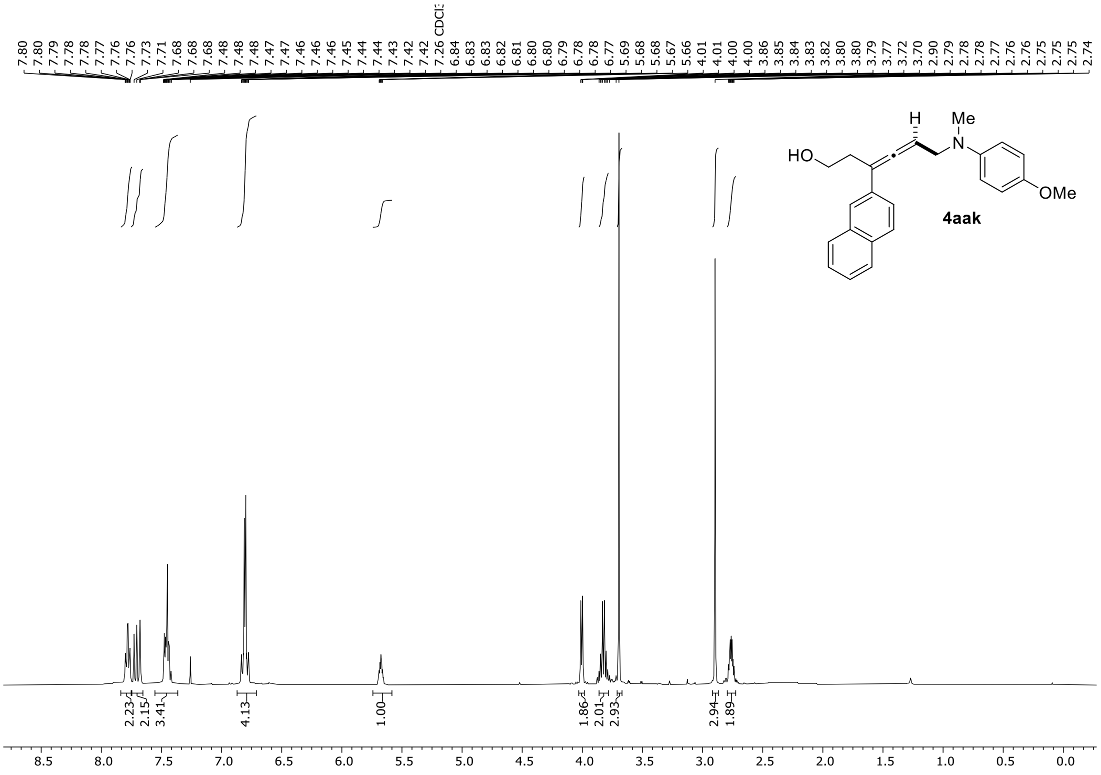

^{13}C NMR (101 MHz, $CDCl_3$)

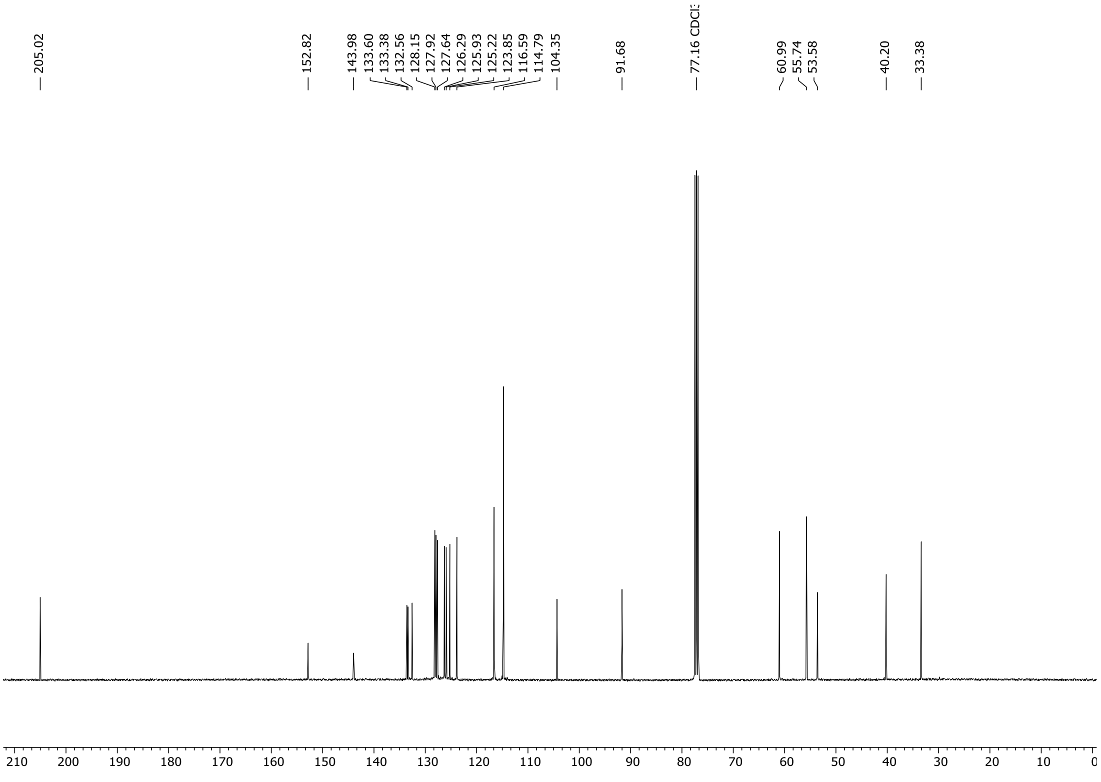

6-((4-Methoxyphenyl)(methyl)amino)-3-(*o*-tolyl)hexa-3,4-dien-1-ol (4aal)

^{1}H NMR (400 MHz, $CDCl_3$)

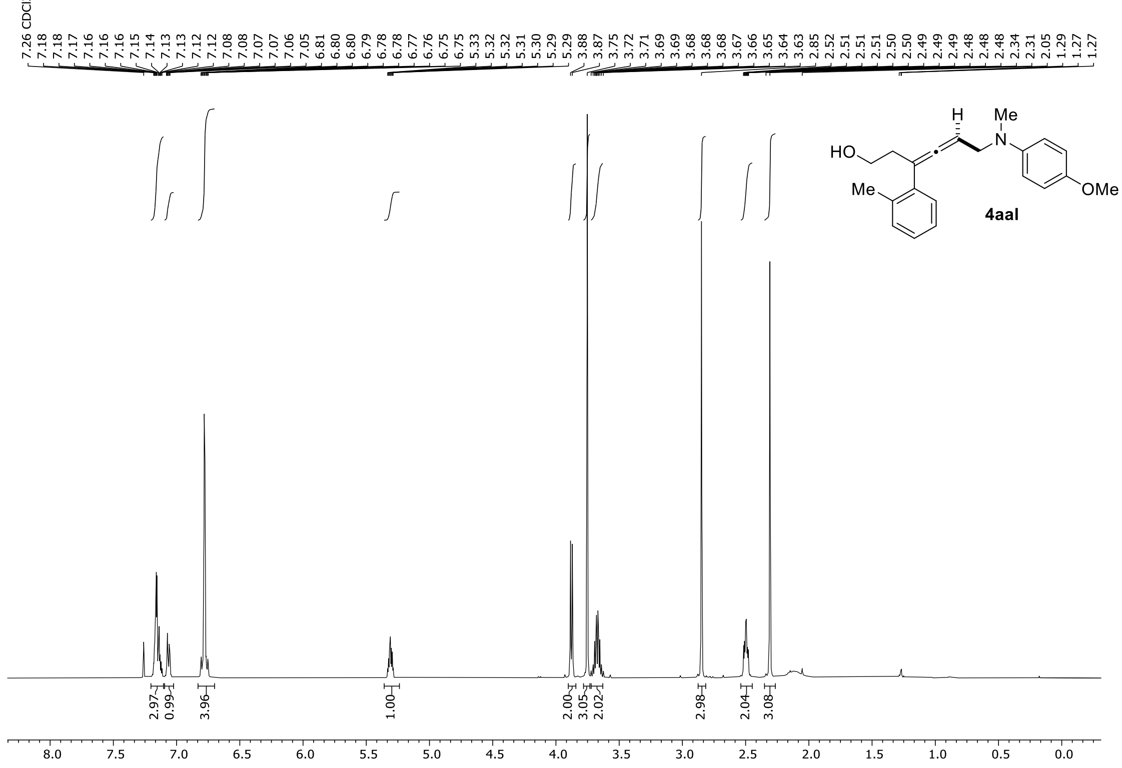

^{13}C NMR (101 MHz, $CDCl_3$)

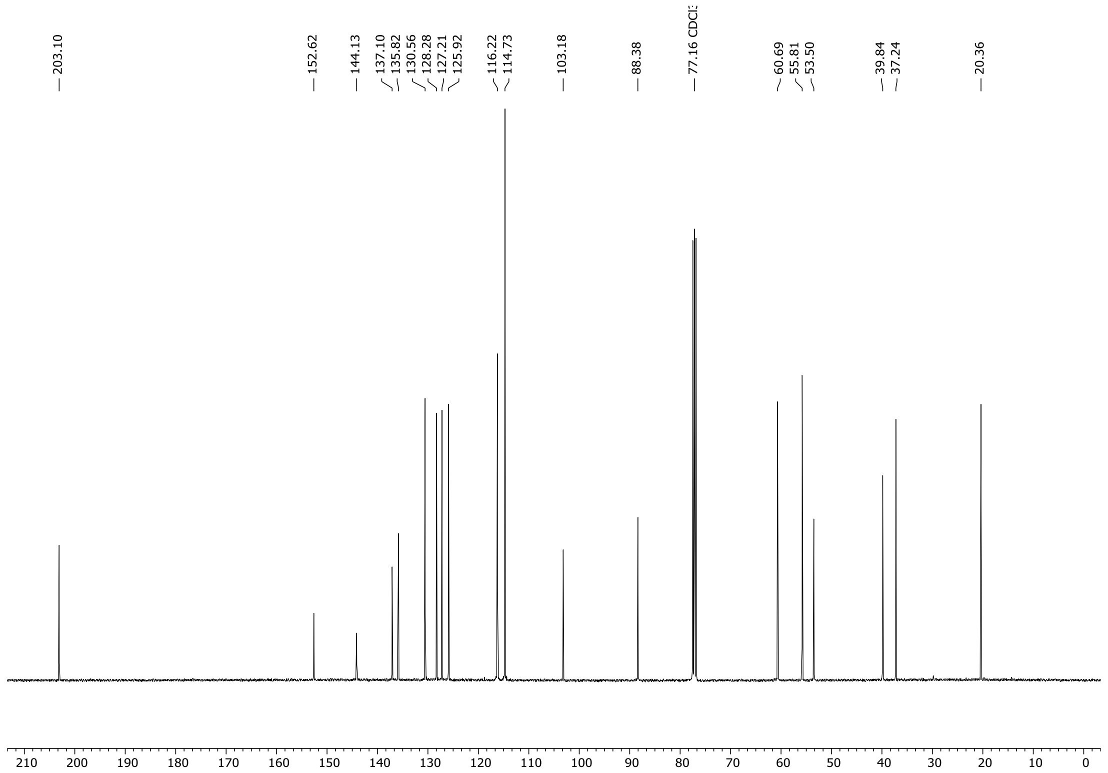

3-(2-Fluorophenyl)-6-((4-methoxyphenyl)(methyl)amino)hexa-3,4-dien-1-ol (4aam)

^{1}H NMR (400 MHz, $CDCl_3$)

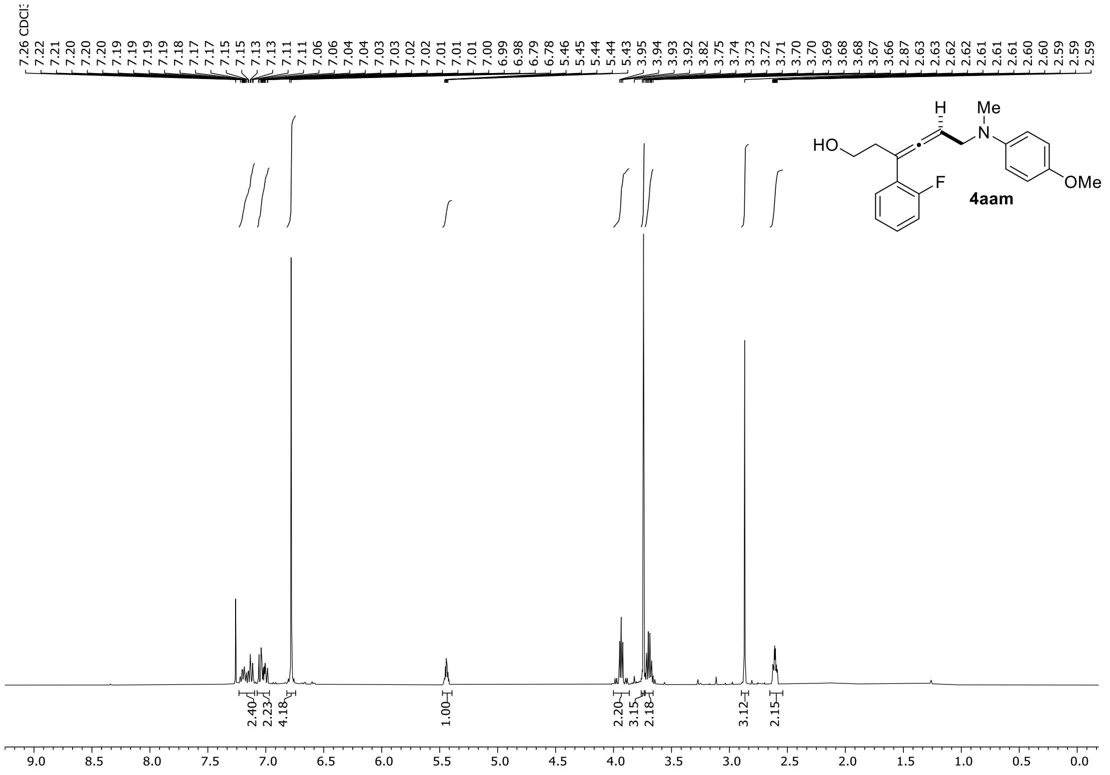

^{13}C NMR (101 MHz, $CDCl_3$)

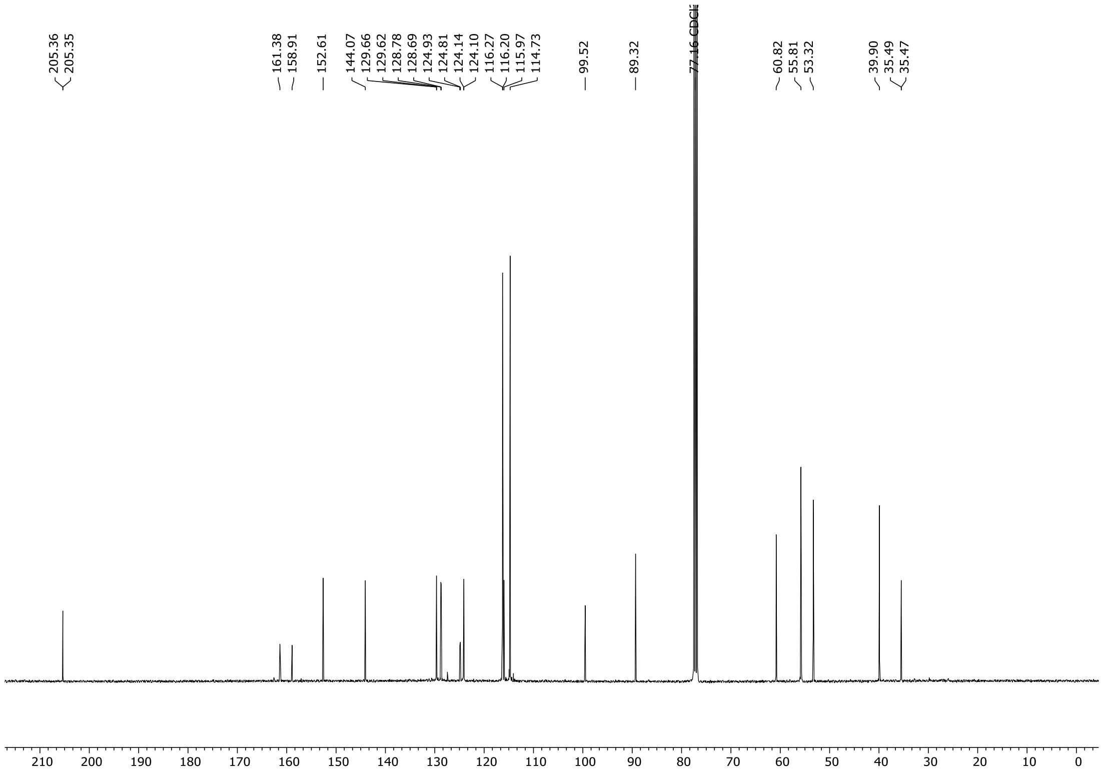

^{19}F NMR (376 MHz, $CDCl_3$)

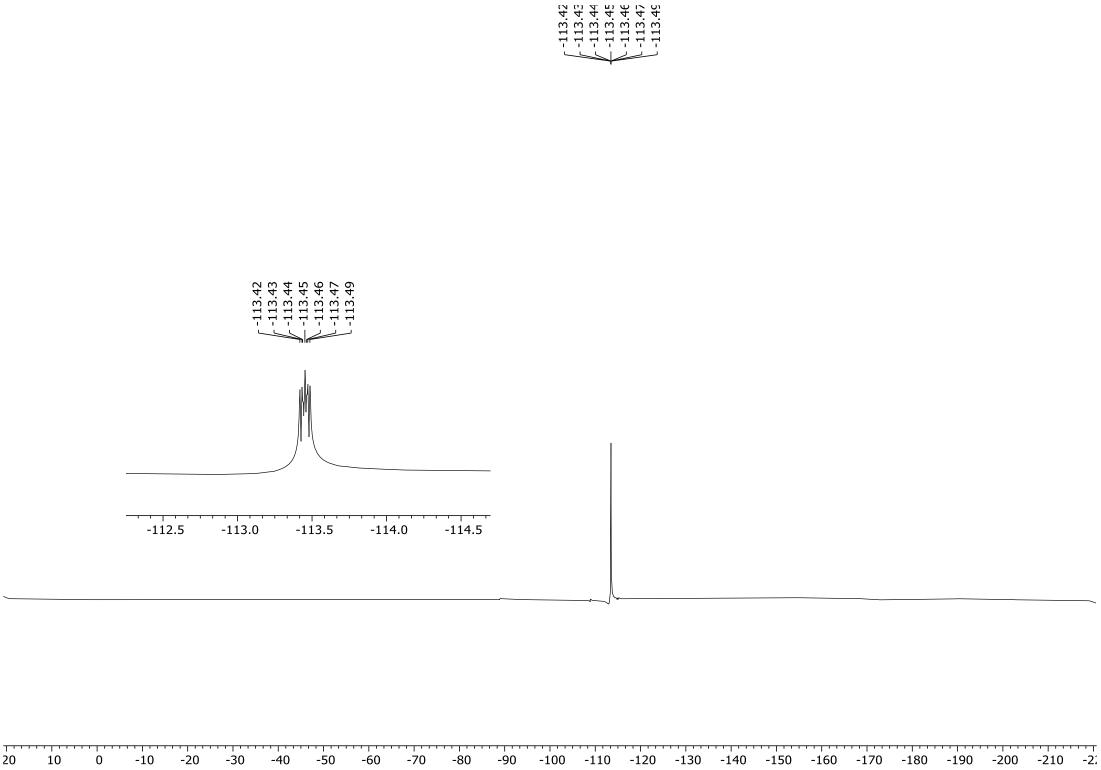

Methyl 2-fluoro-4-(1-hydroxy-6-((4-methoxyphenyl)(methyl)amino)hexa-3,4-dien-3-yl)benzoate (4aan)

^{1}H NMR (400 MHz, $CDCl_3$)

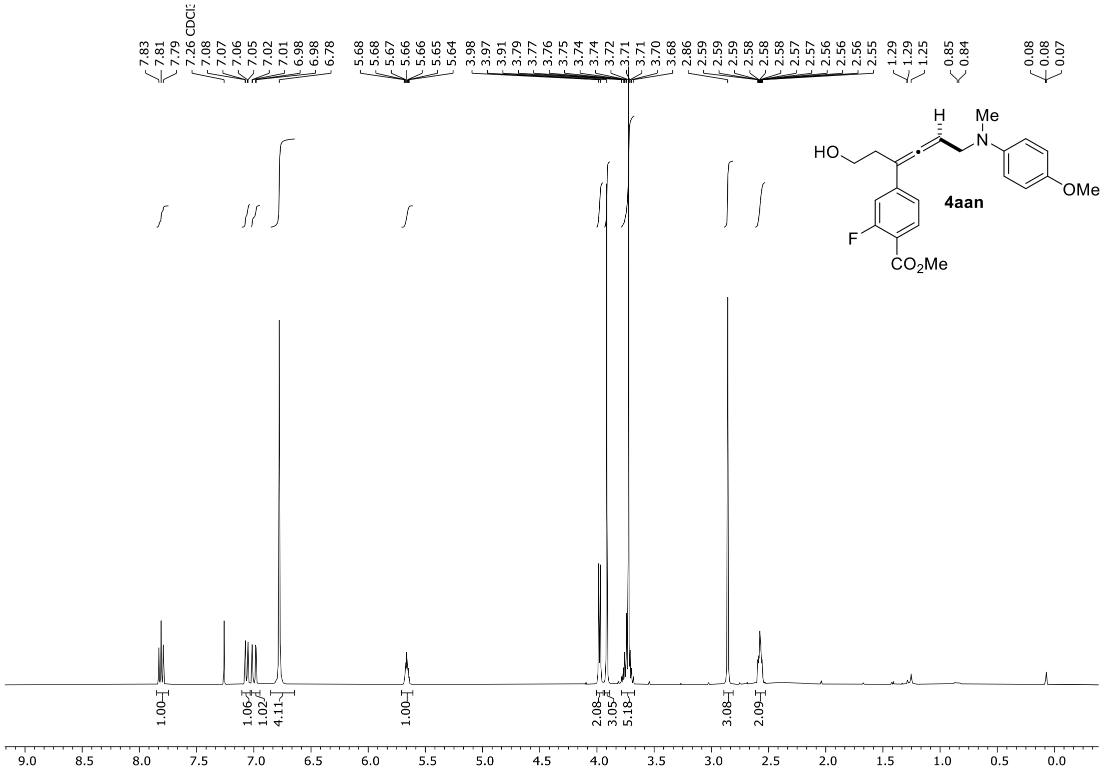

^{13}C NMR (101 MHz, $CDCl_3$)

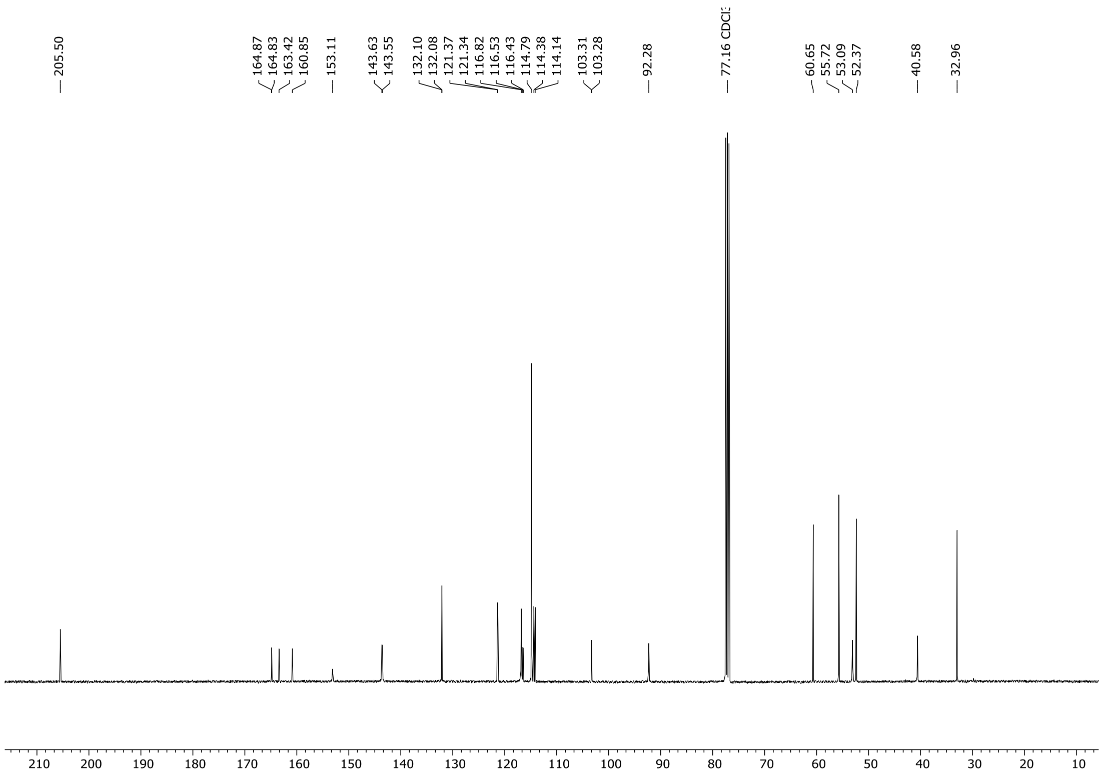

^{19}F NMR (376 MHz, $CDCl_3$)

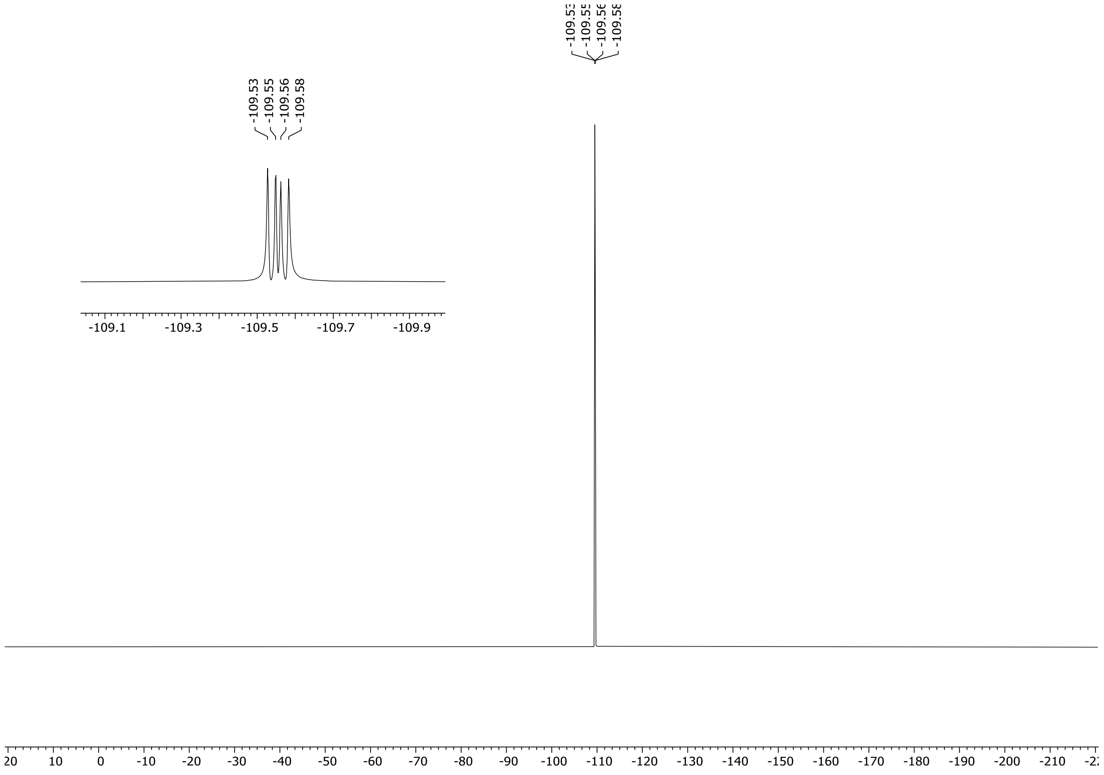

3-(4-Allyl-2-methoxyphenyl)-6-((4-methoxyphenyl)(methyl)amino)hexa-3,4-dien-1-ol (4aao)

^{1}H NMR (400 MHz, $CDCl_3$)

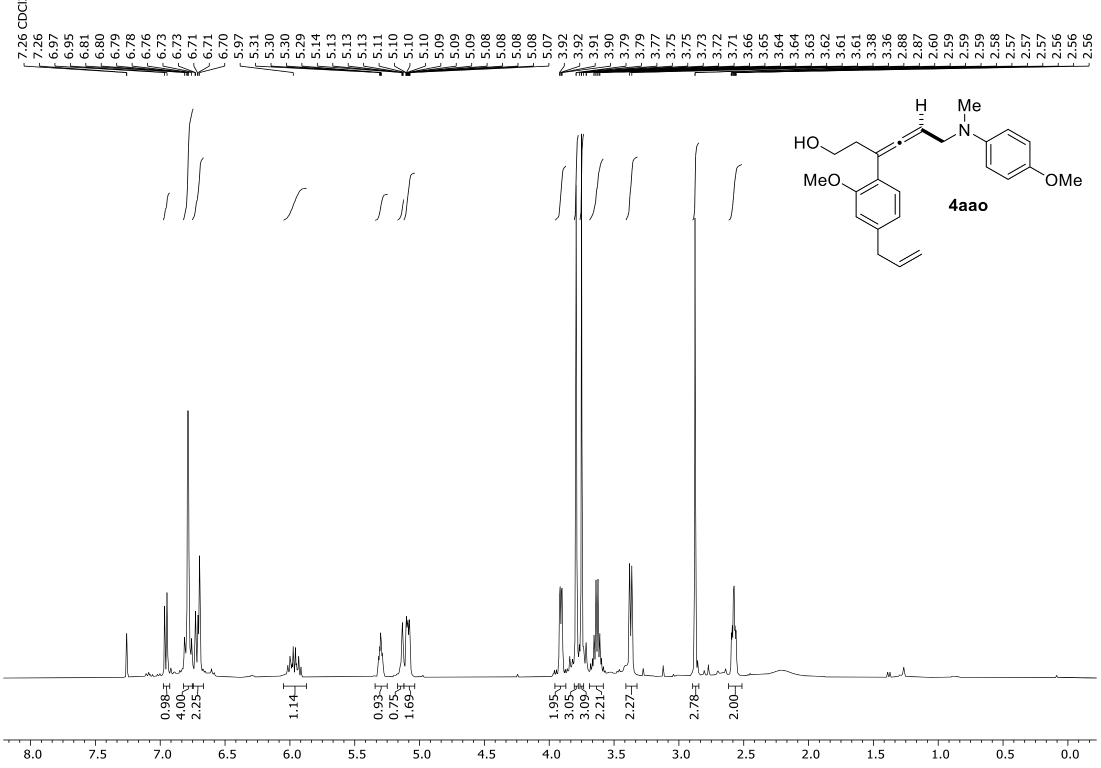

^{13}C NMR (101 MHz, $CDCl_3$)

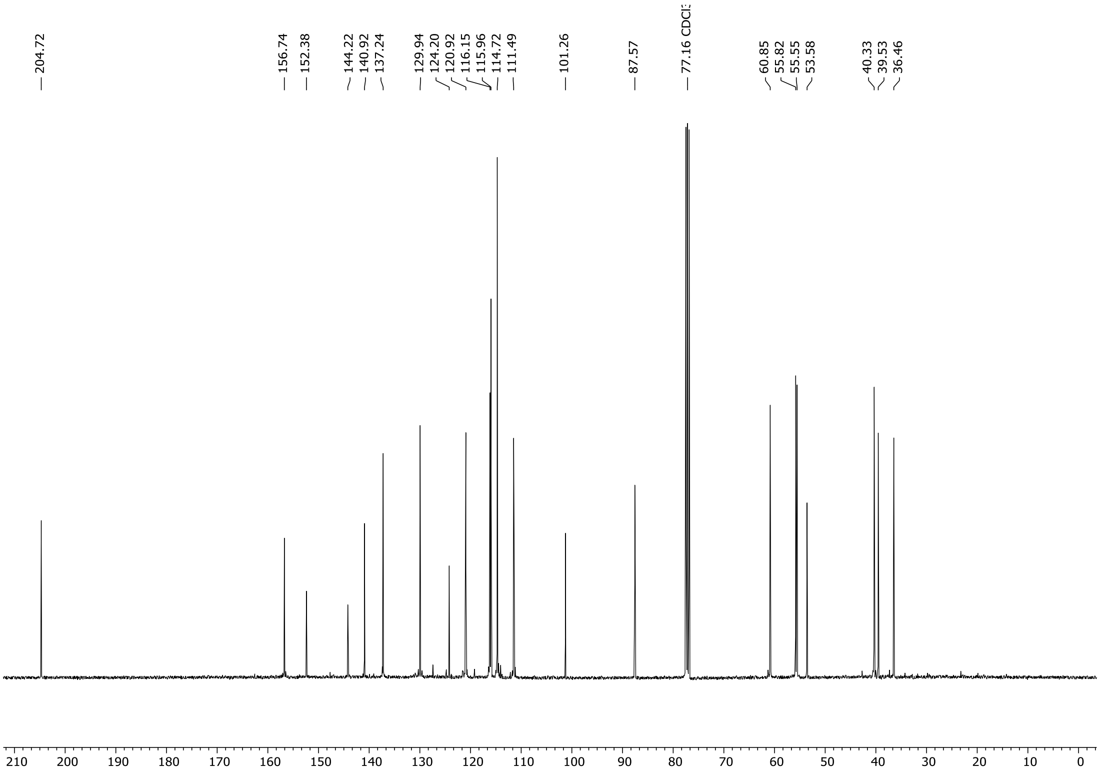

3-(Benzo[*d*][1,3]dioxol-5-yl)-6-((4-methoxyphenyl)(methyl)amino)hexa-3,4-dien-1-ol (4aap)

^{1}H NMR (400 MHz, $CDCl_3$)

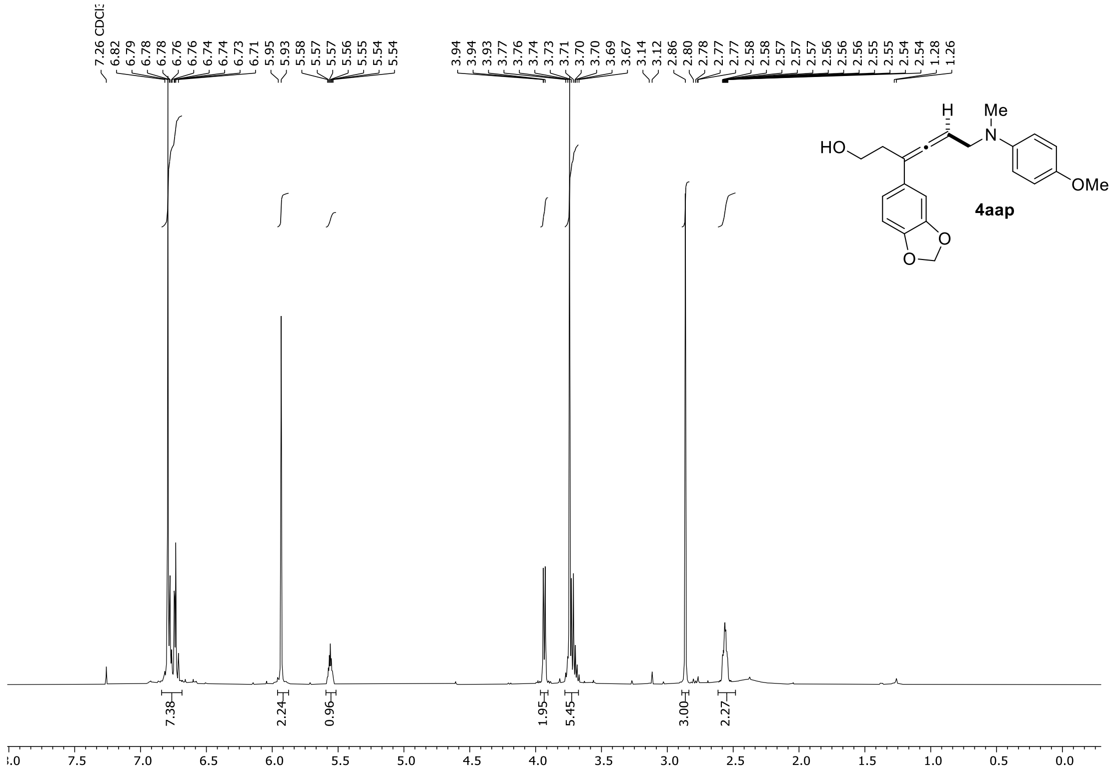

^{13}C NMR (101 MHz, $CDCl_3$)

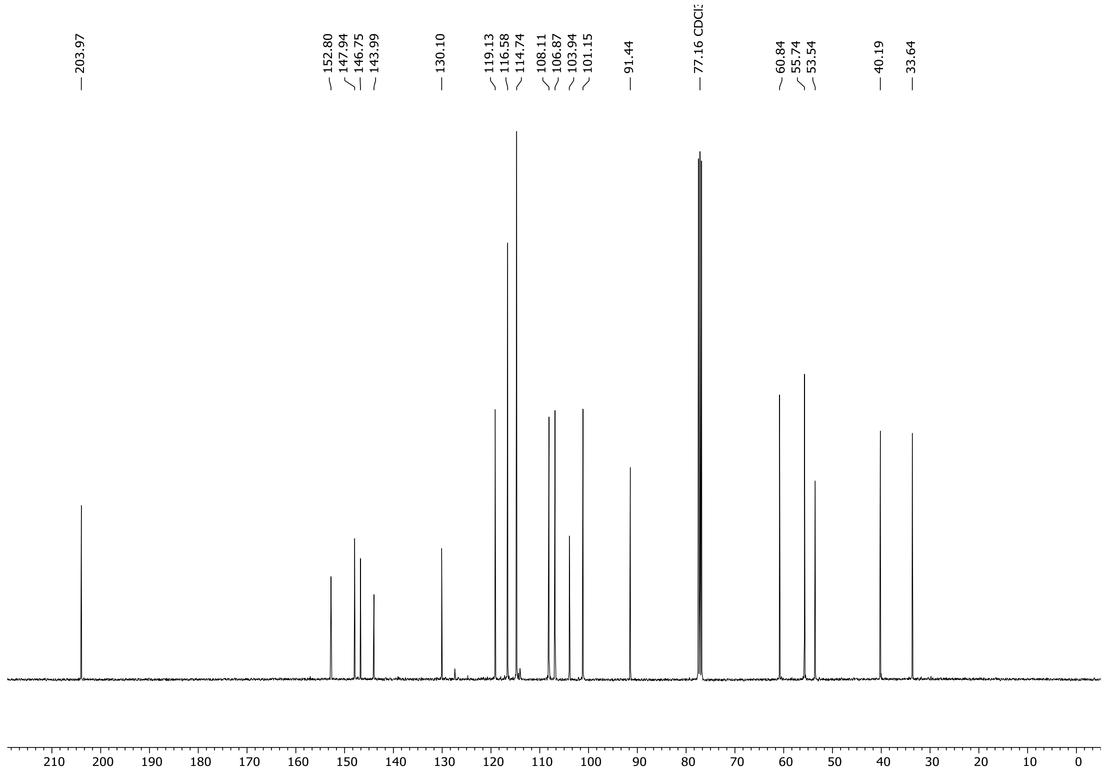

***tert*-Butyl 5-(1-hydroxy-6-((4-methoxyphenyl)(methyl)amino)hexa-3,4-dien-3-yl)-1H-indole-1-carboxylate (4aaq)**

^{1}H NMR (400 MHz, $CDCl_3$)

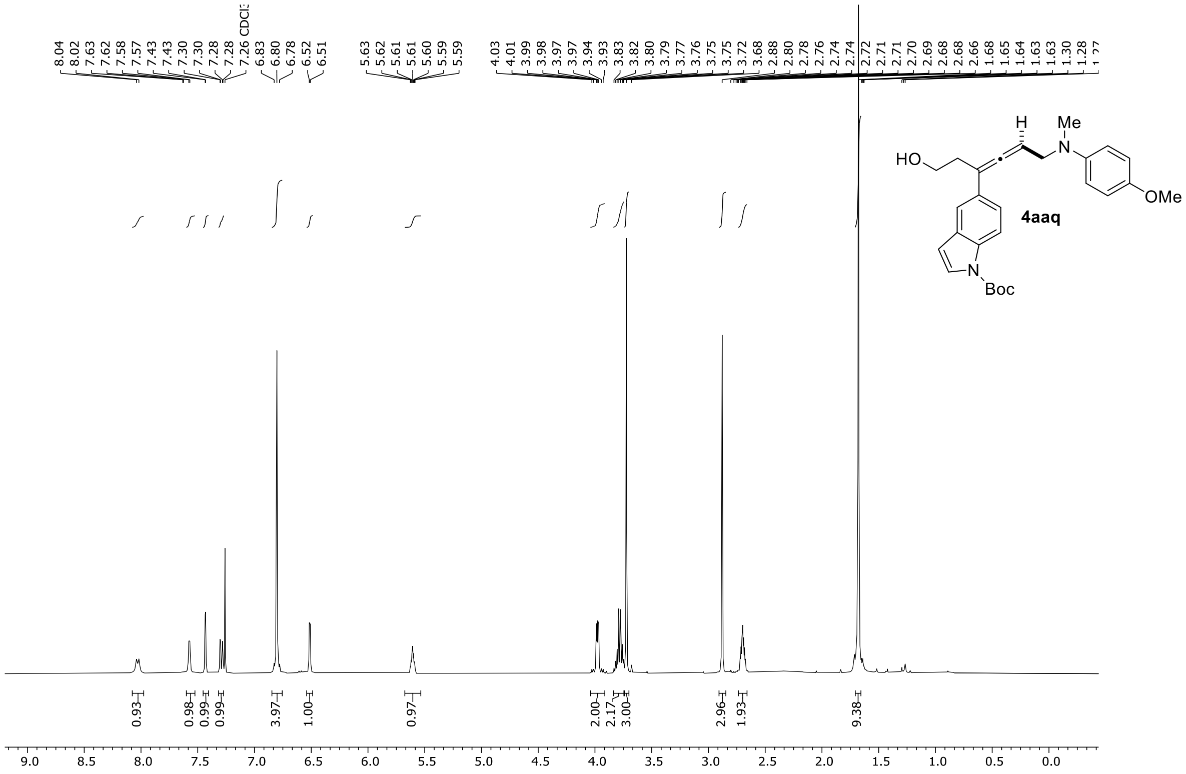

^{13}C NMR (101 MHz, $CDCl_3$)

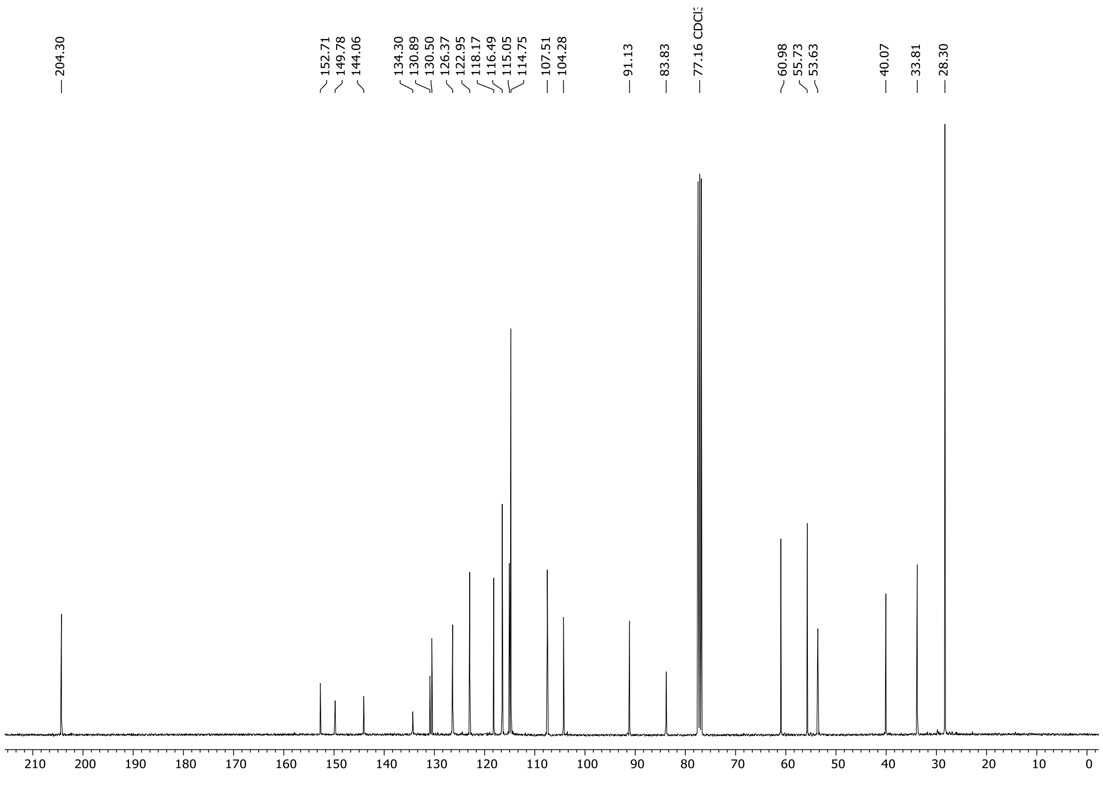

6-((4-Methoxyphenyl)(methyl)amino)-3-(1,7,7-trimethylbicyclo[2.2.1]hept-2-en-2-yl)hexa-3,4-dien-1-ol (4aar)

^{1}H NMR (400 MHz, $CDCl_3$)

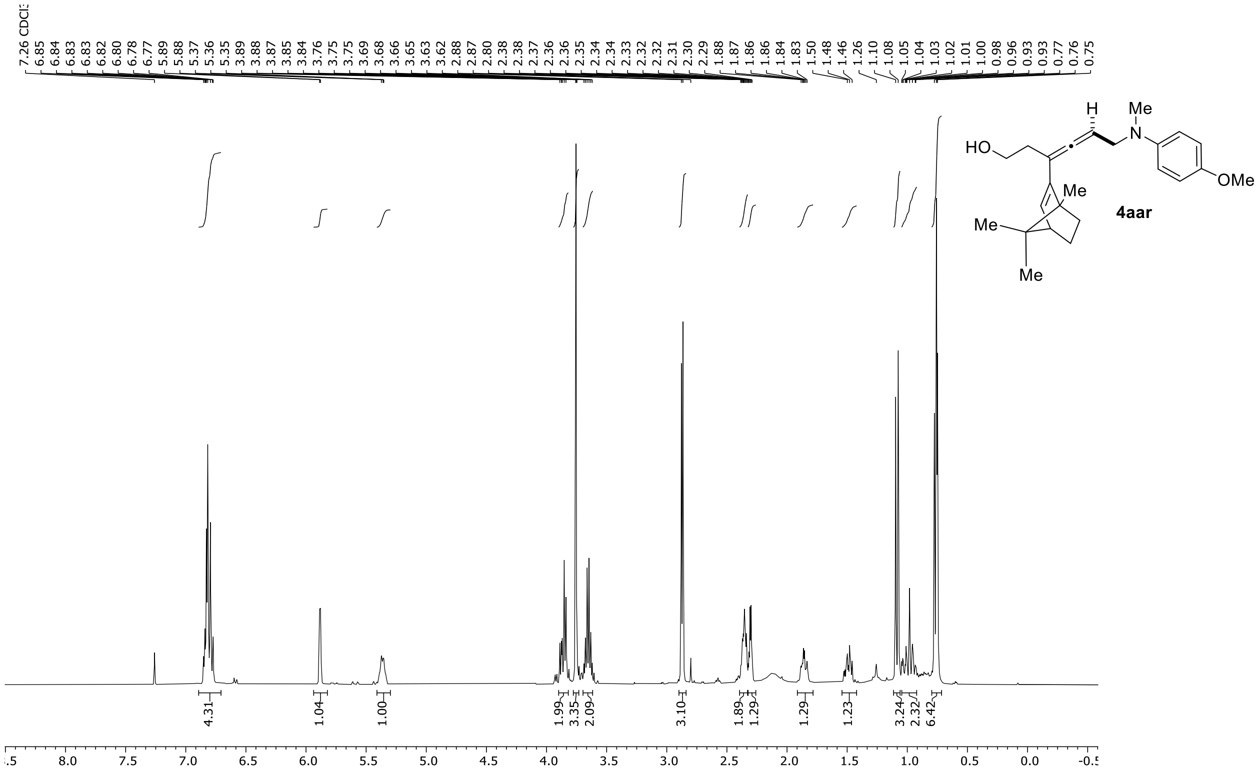

^{13}C NMR (101 MHz, $CDCl_3$)

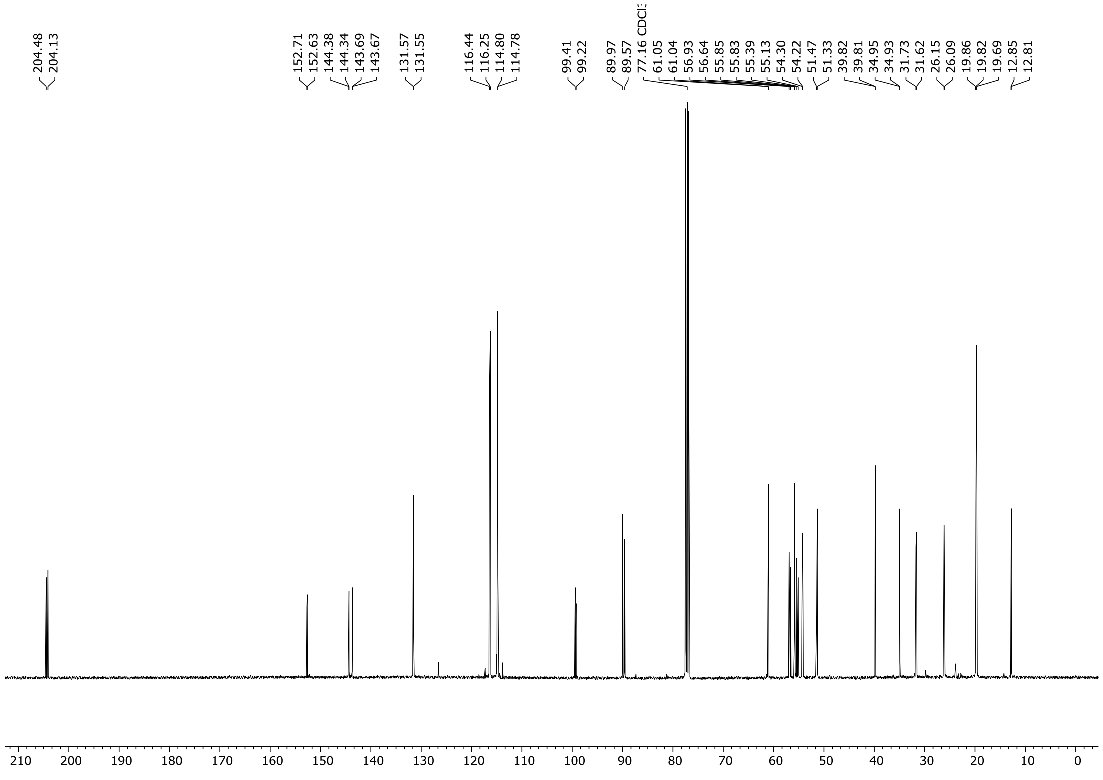

Methyl (2*S*)-2-((tert-butoxycarbonyl)amino)-3-(4-(1-hydroxy-6-((4-methoxyphenyl)(methyl)amino)hexa-3,4-dien-3-yl)phenyl)propanoate (4aas)

^{1}H NMR (400 MHz, $CDCl_3$)

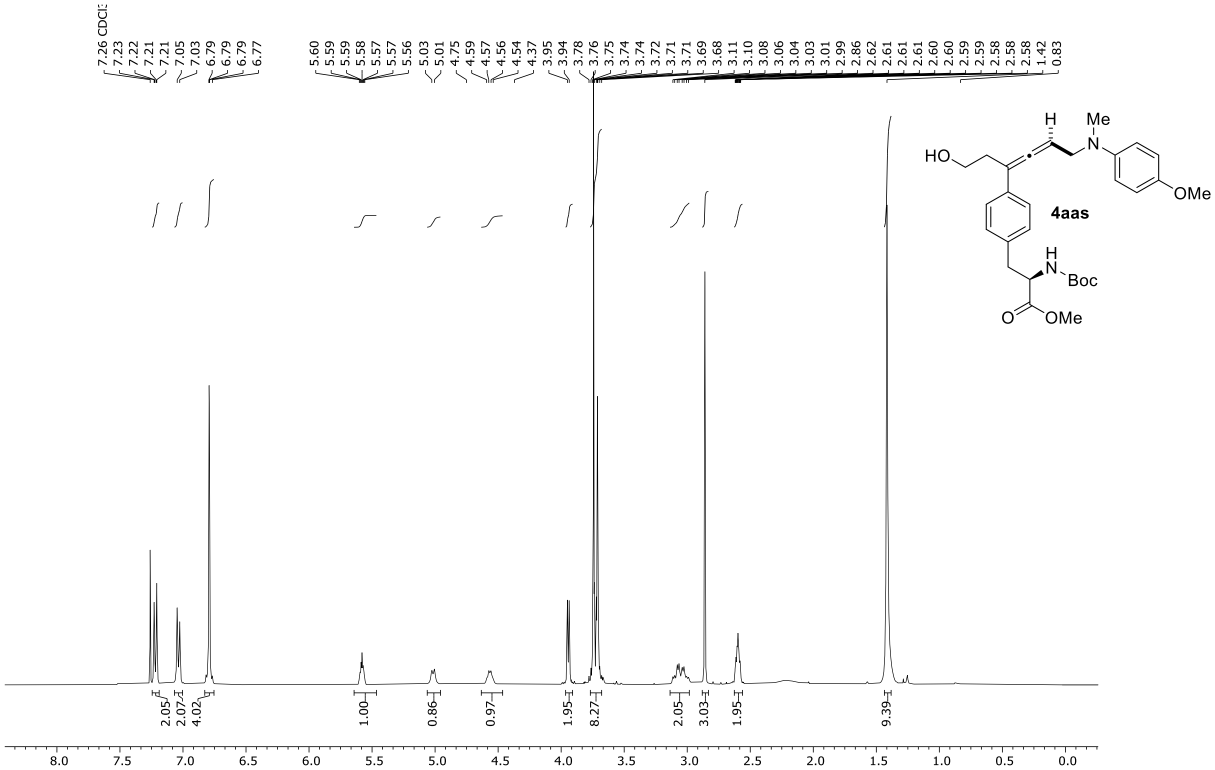

^{13}C NMR (101 MHz, $CDCl_3$)

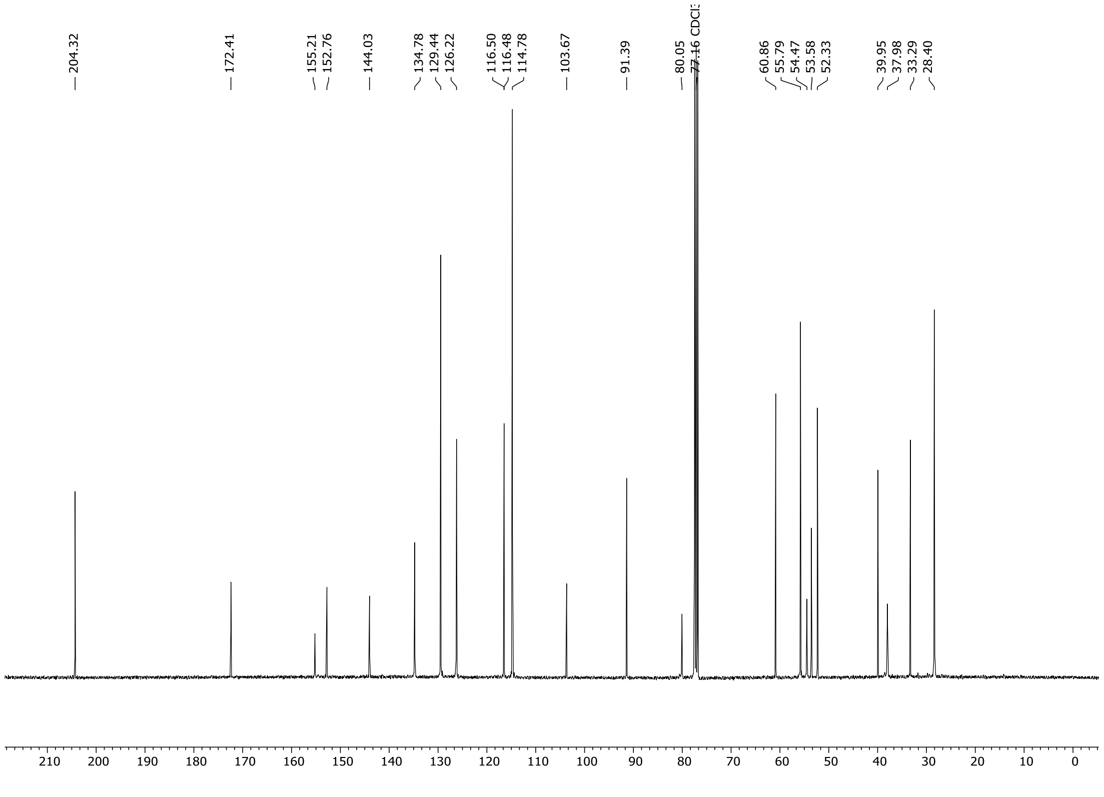

(8*R*,9*S*,13*S*,14*S*)-3-(1-Hydroxy-6-((4-methoxyphenyl)(methyl)amino)hexa-3,4-dien-3-yl)-13-methyl-6,7,8,9,11,12,13,14,15,16-decahydro-17*H*-cyclopenta[a]phenanthren-17-one (4aat)

^{1}H NMR (400 MHz, CDCl$_3$)

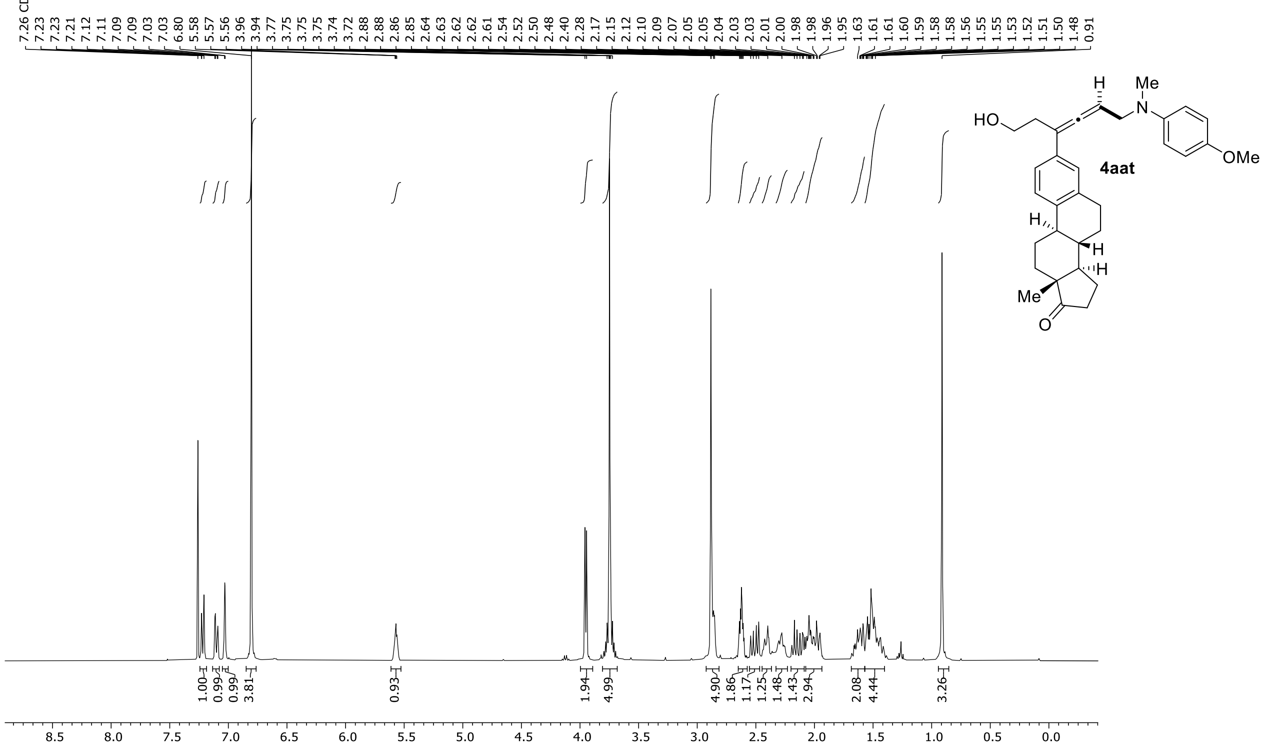

^{13}C NMR (101 MHz, CDCl$_3$)

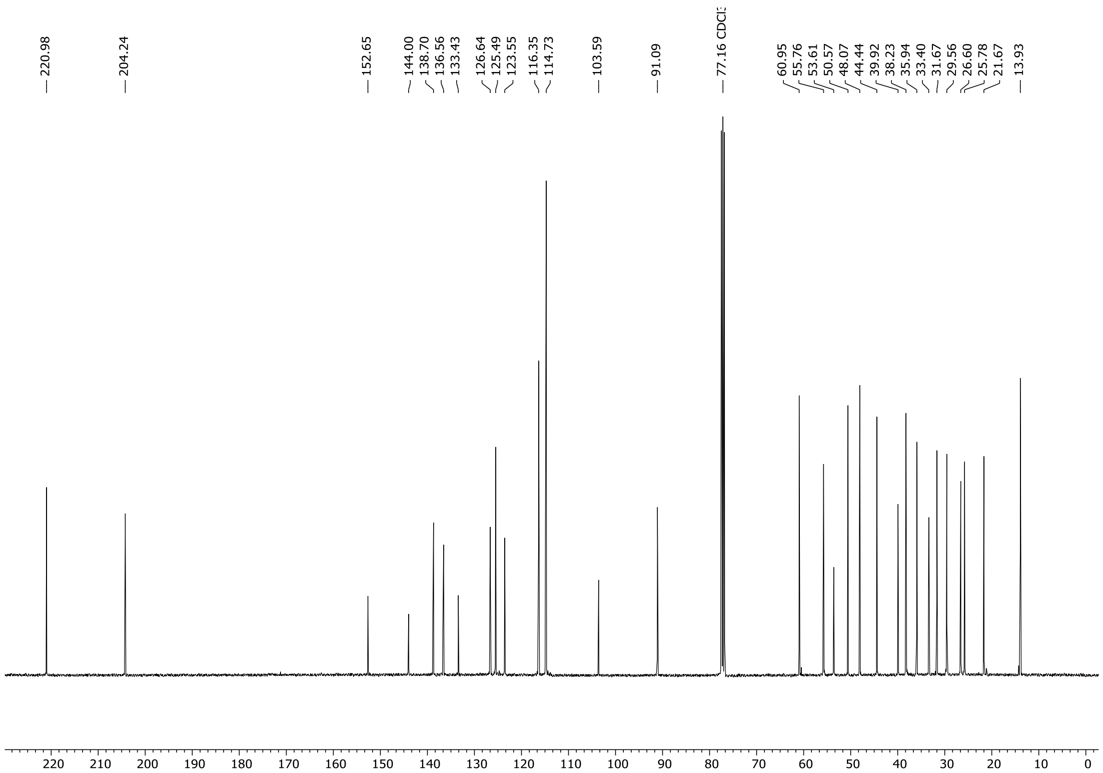

Methyl 4-(6-hydroxy-1-((4-methoxyphenyl)(methyl)amino)hepta-2,3-dien-4-yl)benzoate (4jab)

^1H NMR (400 MHz, $CDCl_3$)

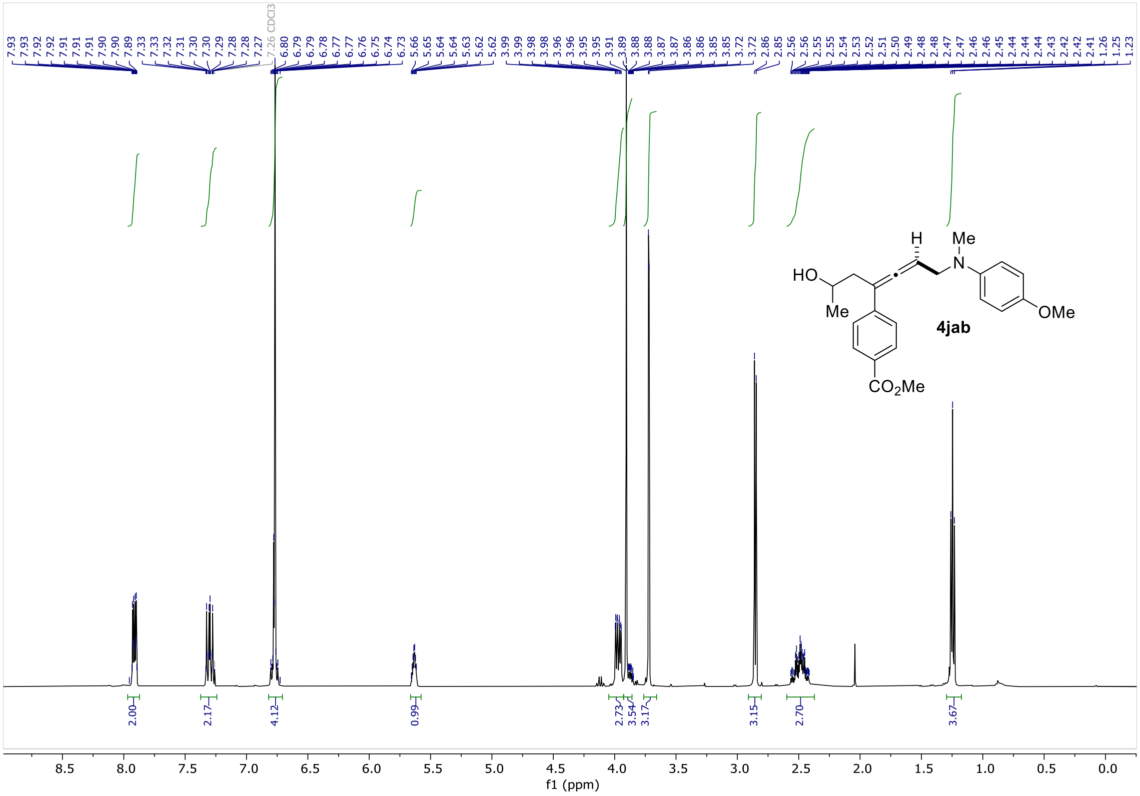

^{13}C NMR (101 MHz, $CDCl_3$)

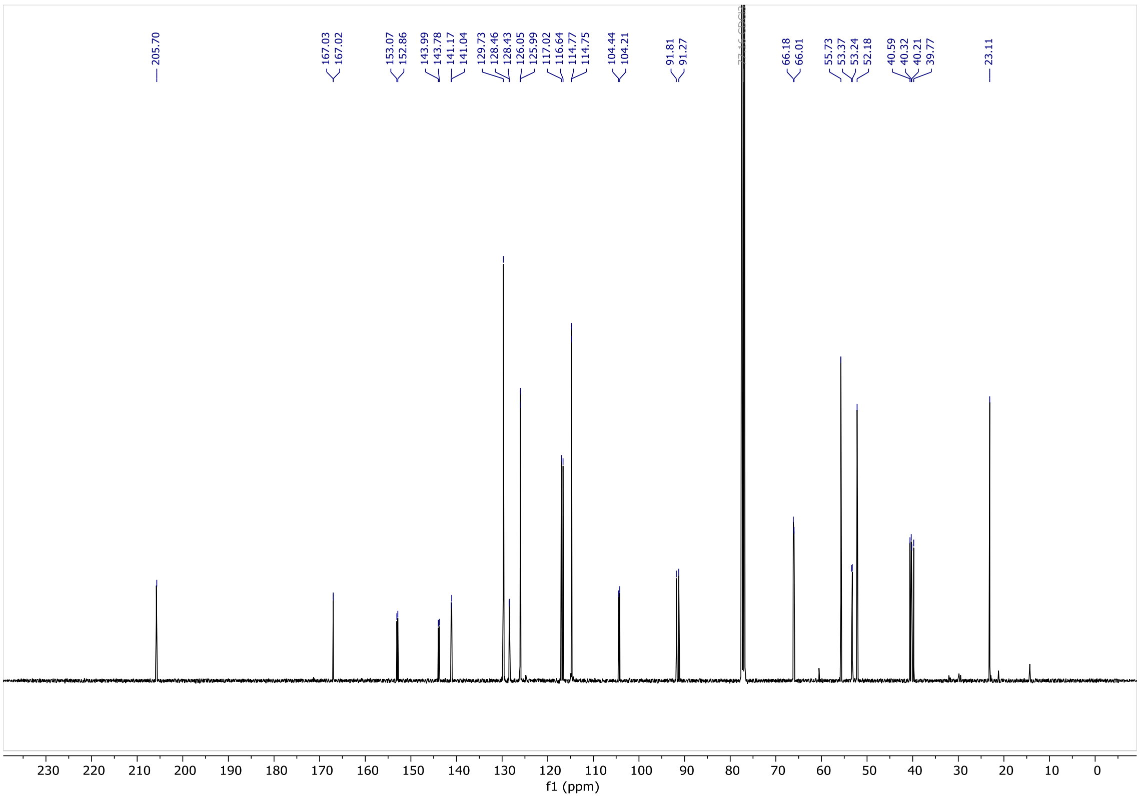

Methyl 4-(1-hydroxy-6-((4-methoxyphenyl)(methyl)amino)-5-methylhexa-3,4-dien-3-yl)benzoate (4kab)

^{1}H NMR (400 MHz, CDCl$_3$)

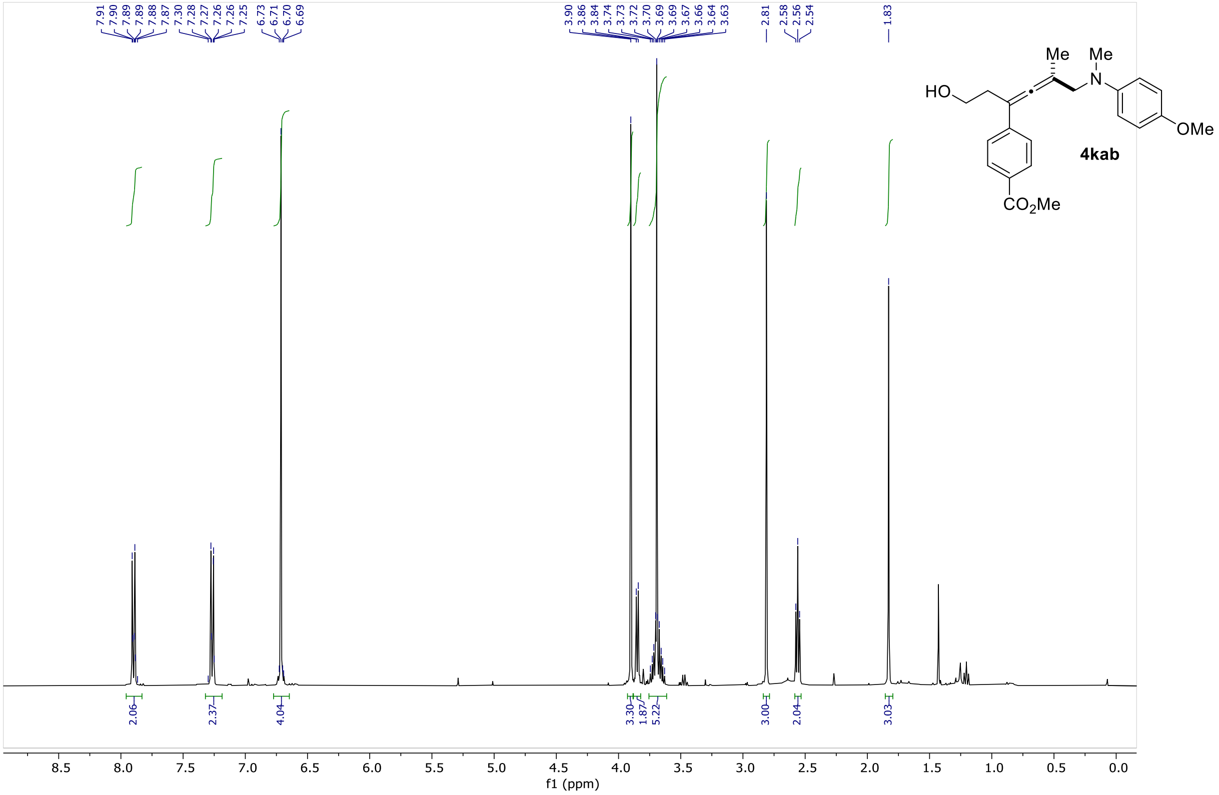

^{13}C NMR (101 MHz, CDCl$_3$)

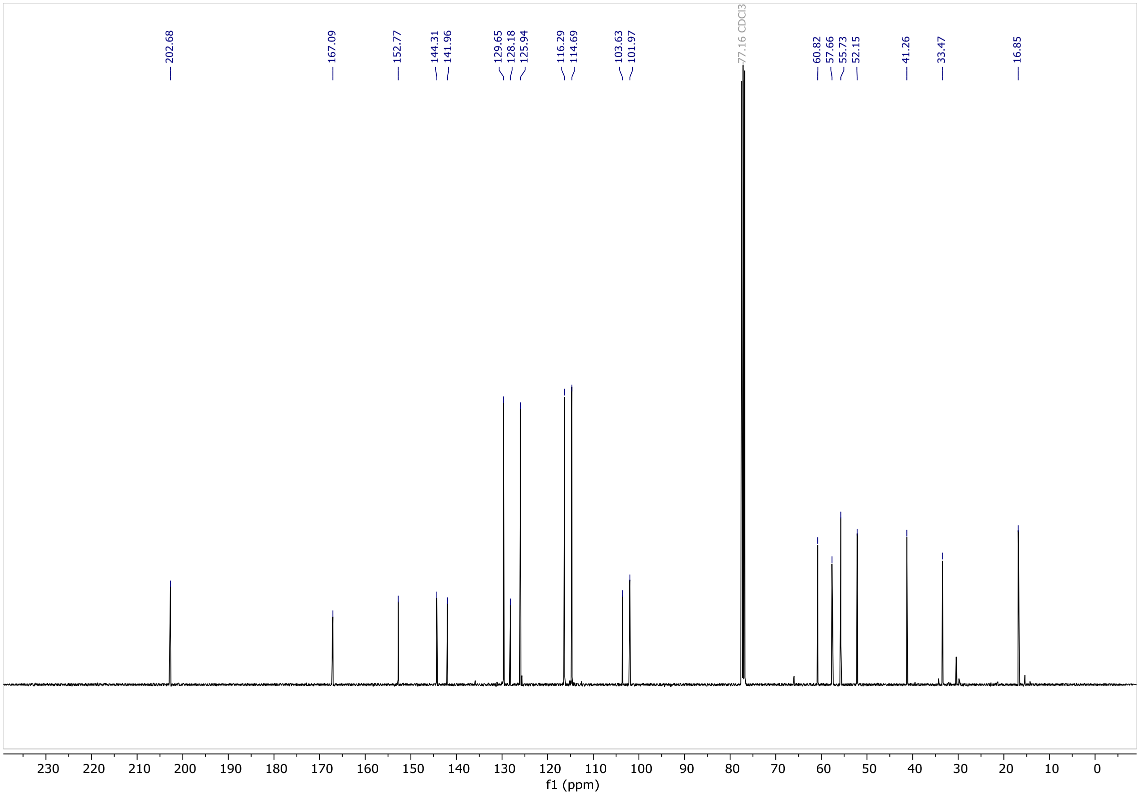

7-((4-Methoxyphenyl)(methyl)amino)-4-(*p*-tolyl)hepta-4,5-dien-1-ol (4baa)

^{1}H NMR (400 MHz, $CDCl_3$)

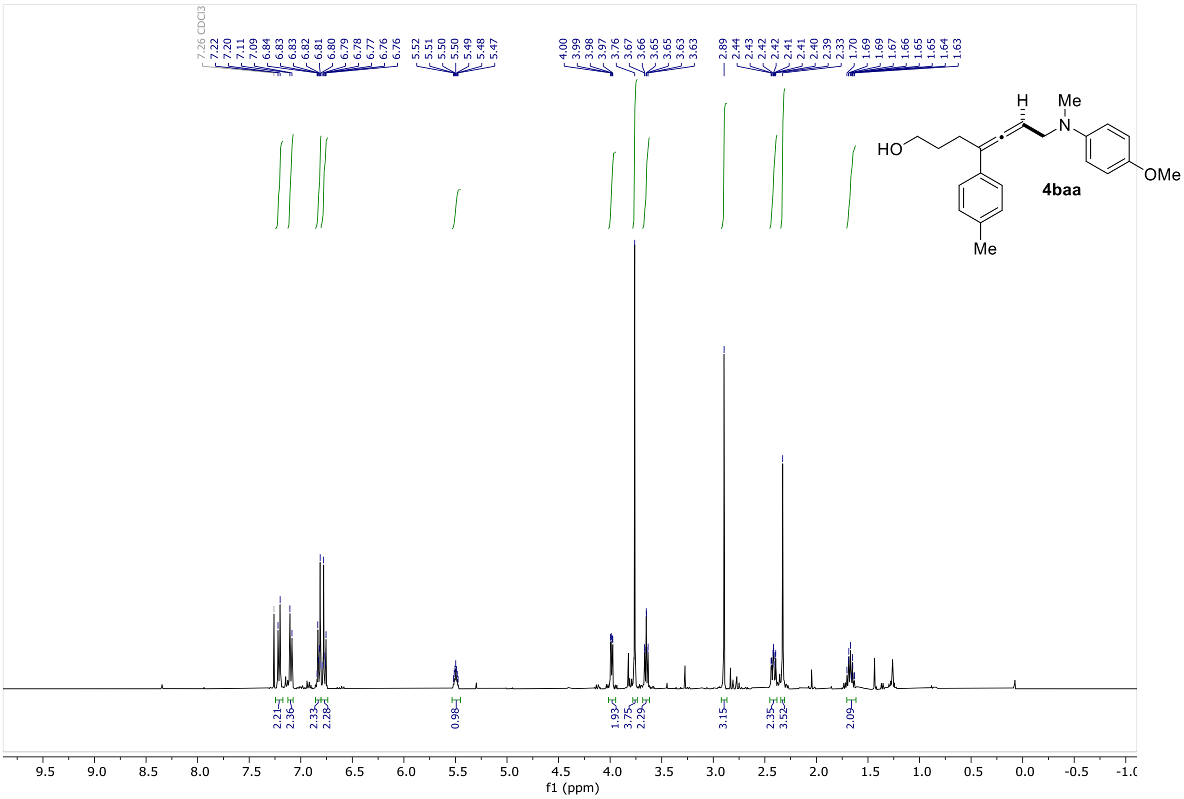

^{13}C NMR (101 MHz, $CDCl_3$)

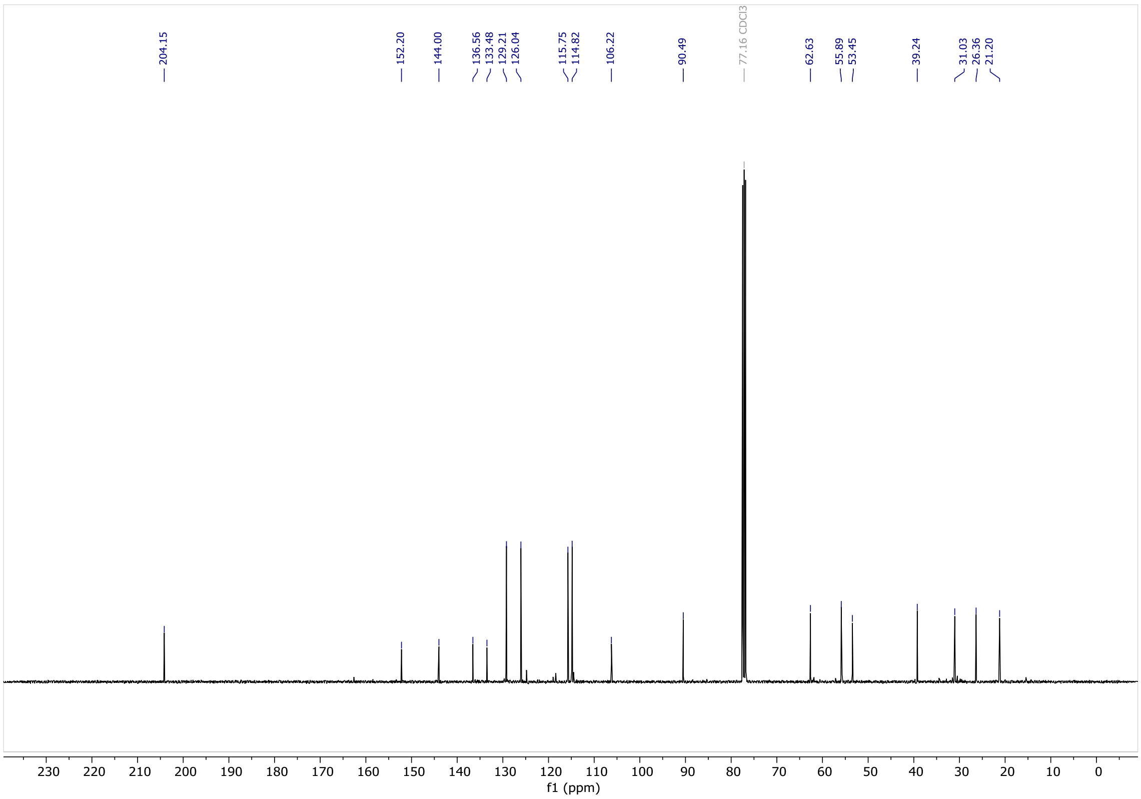

***N*-(6-(Benzyloxy)-4-(*p*-tolyl)hexa-2,3-dien-1-yl)-4-methoxy-*N*-methylaniline (4daa)**

^{1}H NMR (400 MHz, $CDCl_3$)

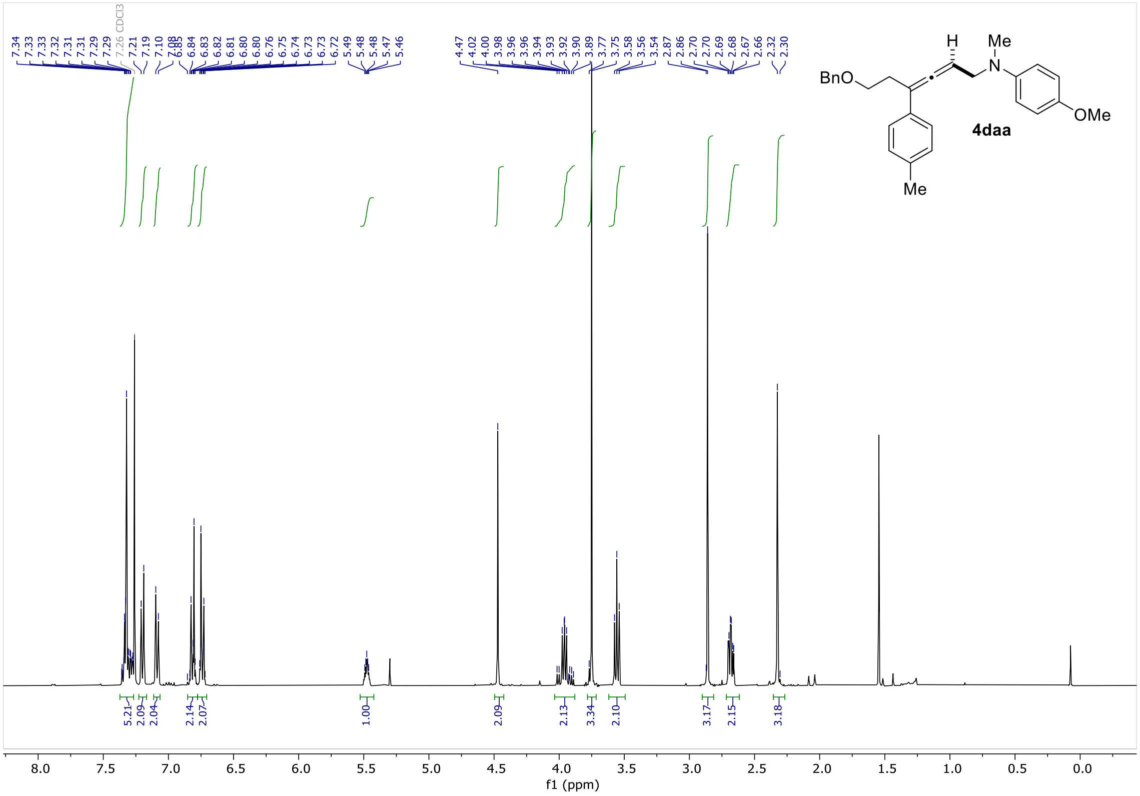

^{13}C NMR (101 MHz, $CDCl_3$)

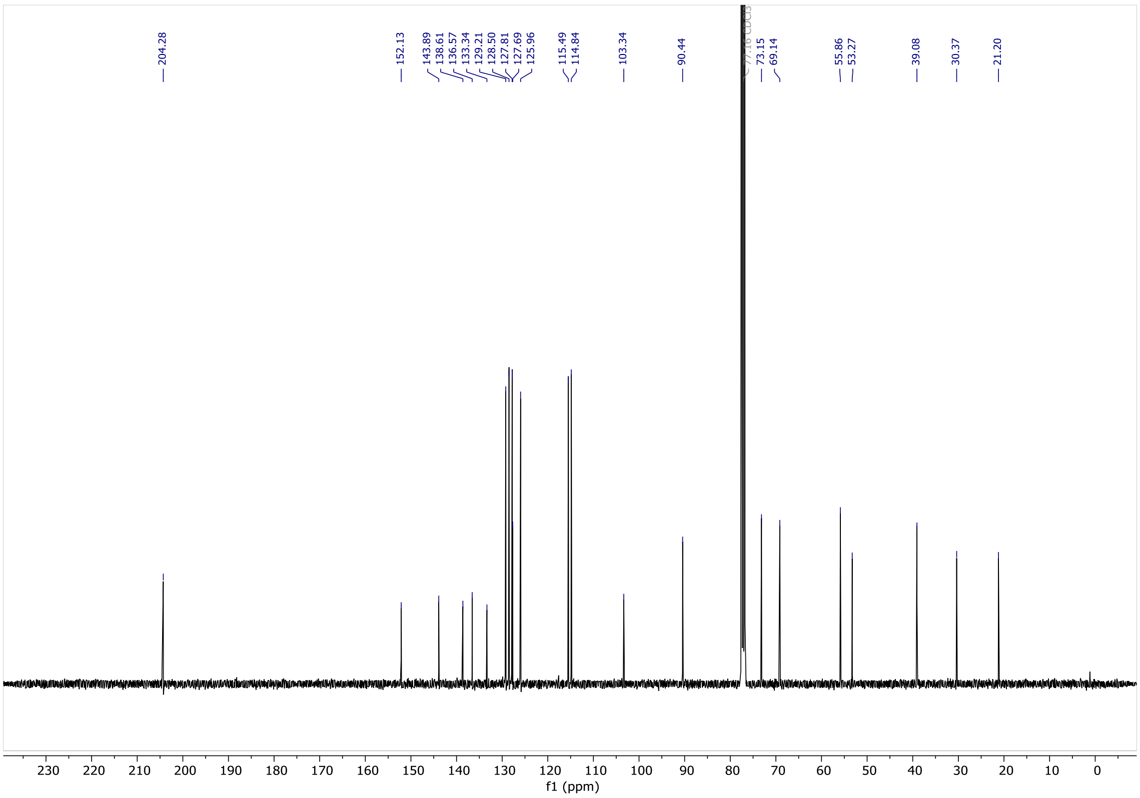

_N_-(6-((tert-butyldimethylsilyl)oxy)-4-(_p_-tolyl)hexa-2,3-dien-1-yl)-4-(trifluoromethyl)aniline (S34)

^{1}H NMR (400 MHz, $CDCl_3$)

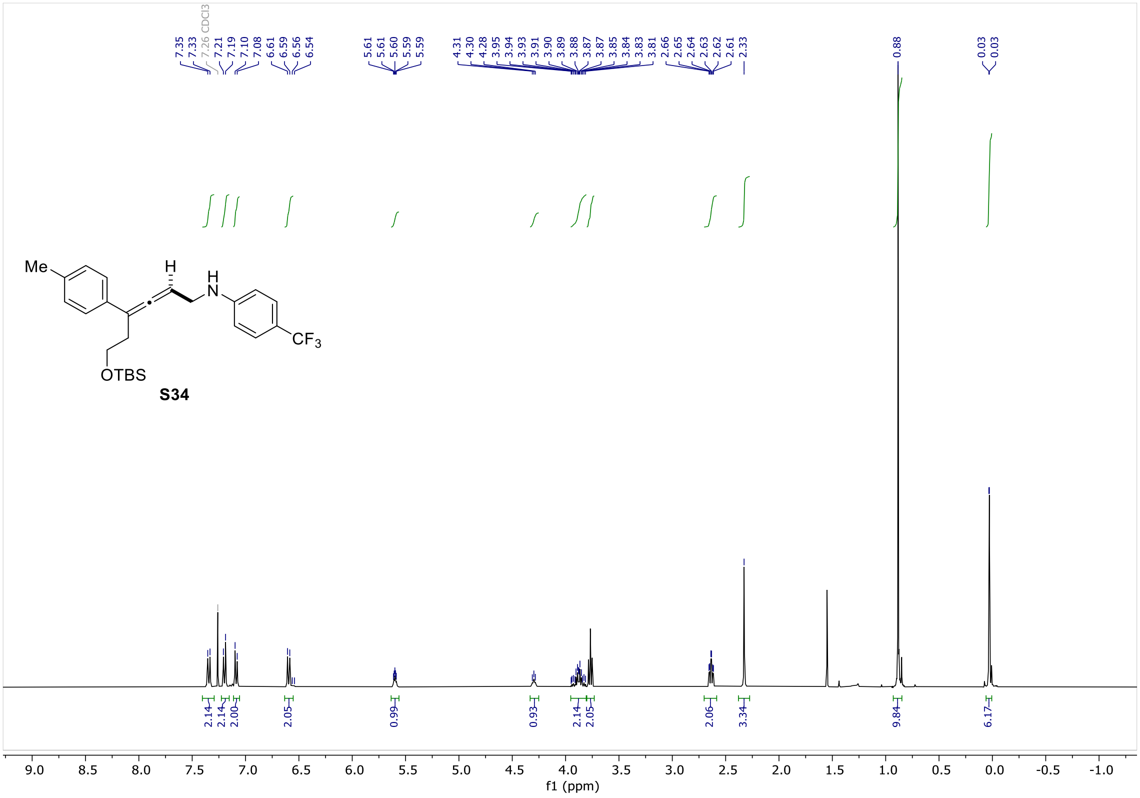

^{13}C NMR (101 MHz, $CDCl_3$)

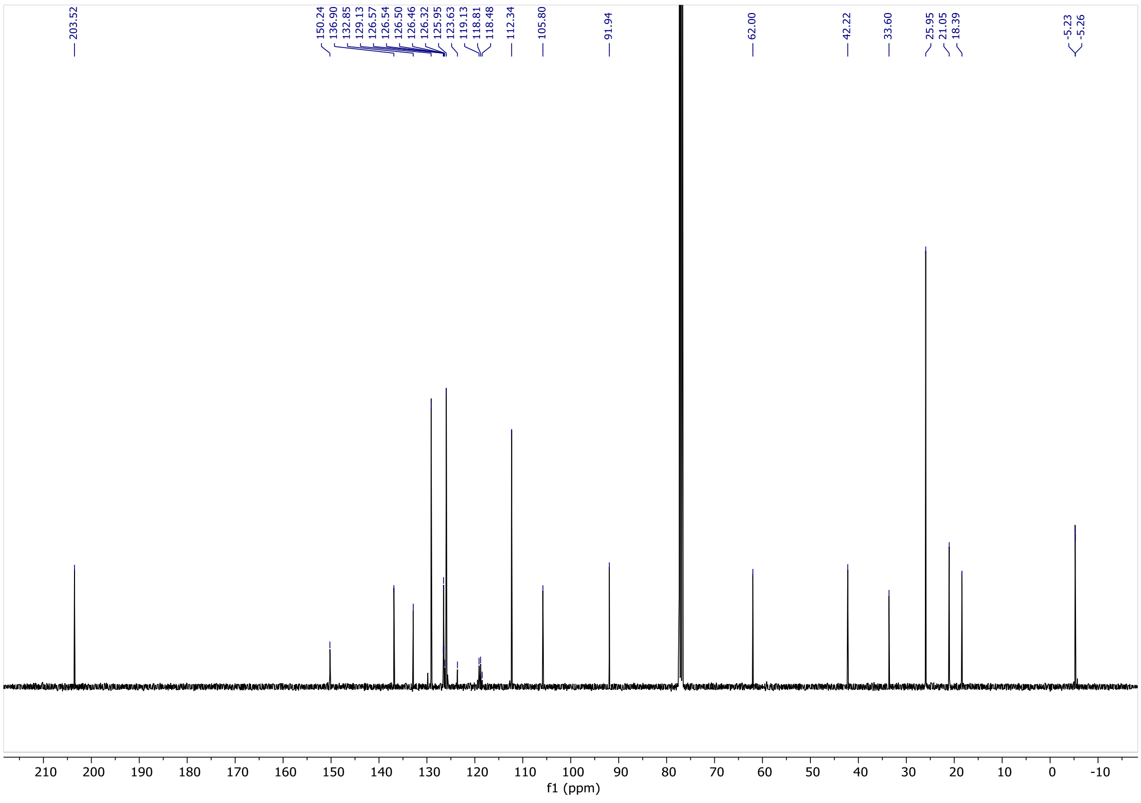

I.3. Spectra of 3-pyrrolines

Methyl 4-(2-(2-hydroxyethyl)-2-(*p*-tolyl)-1-(4-(trifluoromethyl)phenyl)-2,5-dihydro-1*H*-pyrrol-3-yl)benzoate (7a)

^{1}H NMR (400 MHz, CDCl$_3$)

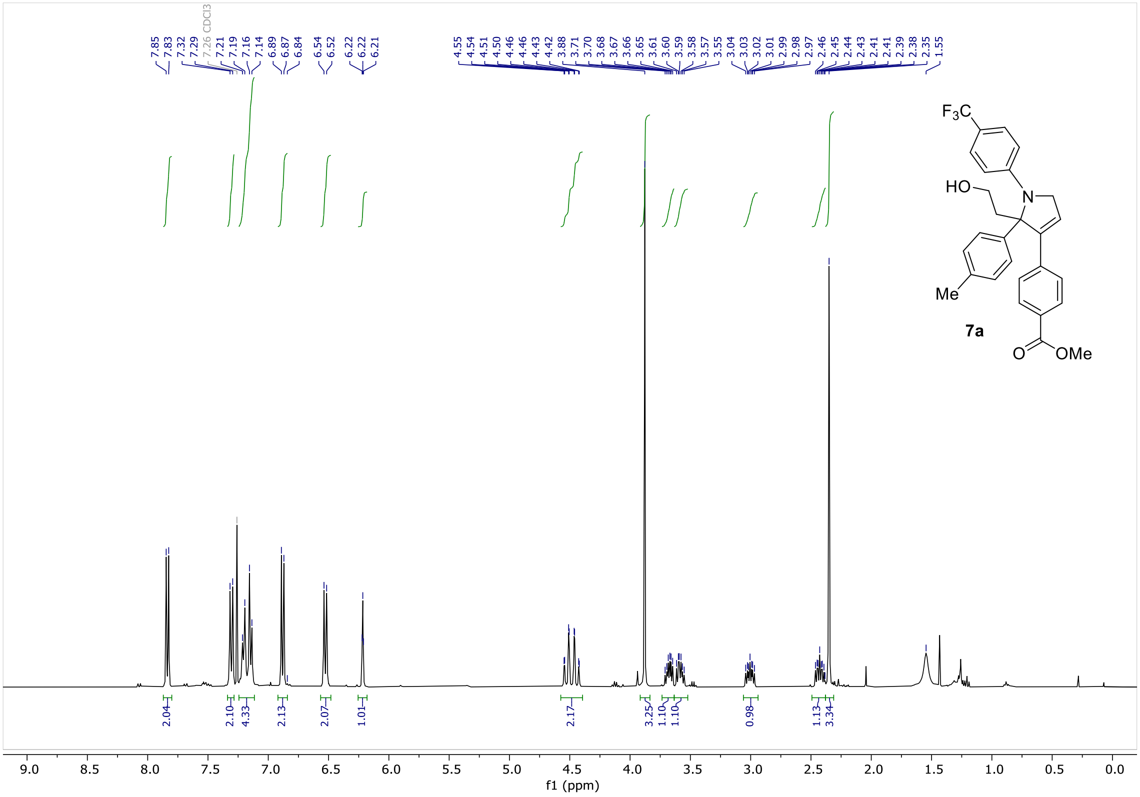

^{13}C NMR (101 MHz, CDCl$_3$)

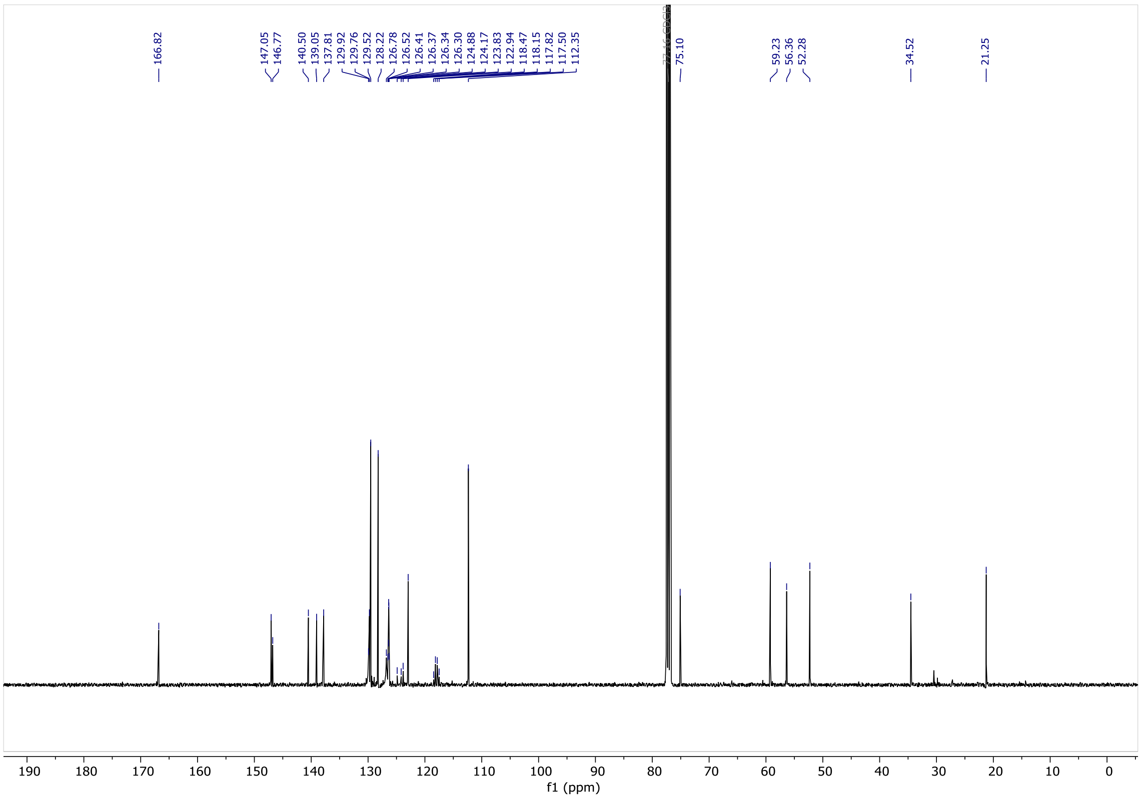

2-(3-(4-(*tert*-Butyl)phenyl)-2-(*p*-tolyl)-1-(4-(trifluoromethyl)phenyl)-2,5-dihydro-1H-pyrrol-2-yl)ethan-1-ol (7c)

^{1}H NMR (400 MHz, $CDCl_3$)

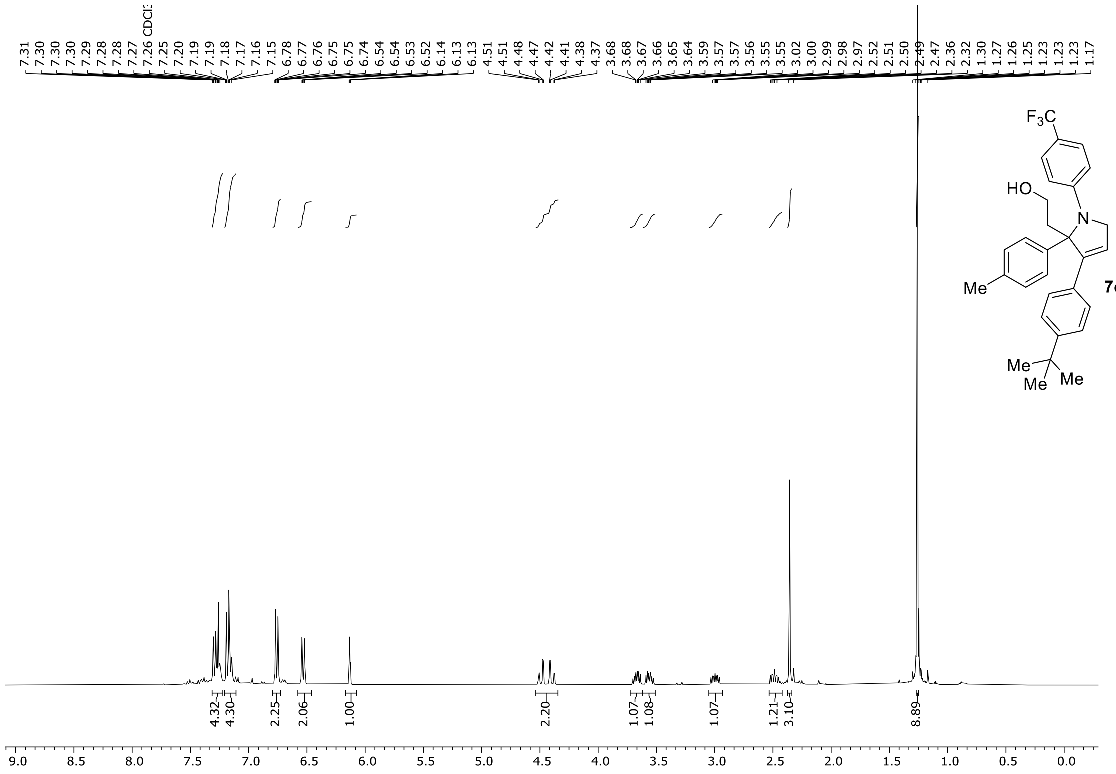

^{13}C NMR (101 MHz, $CDCl_3$)

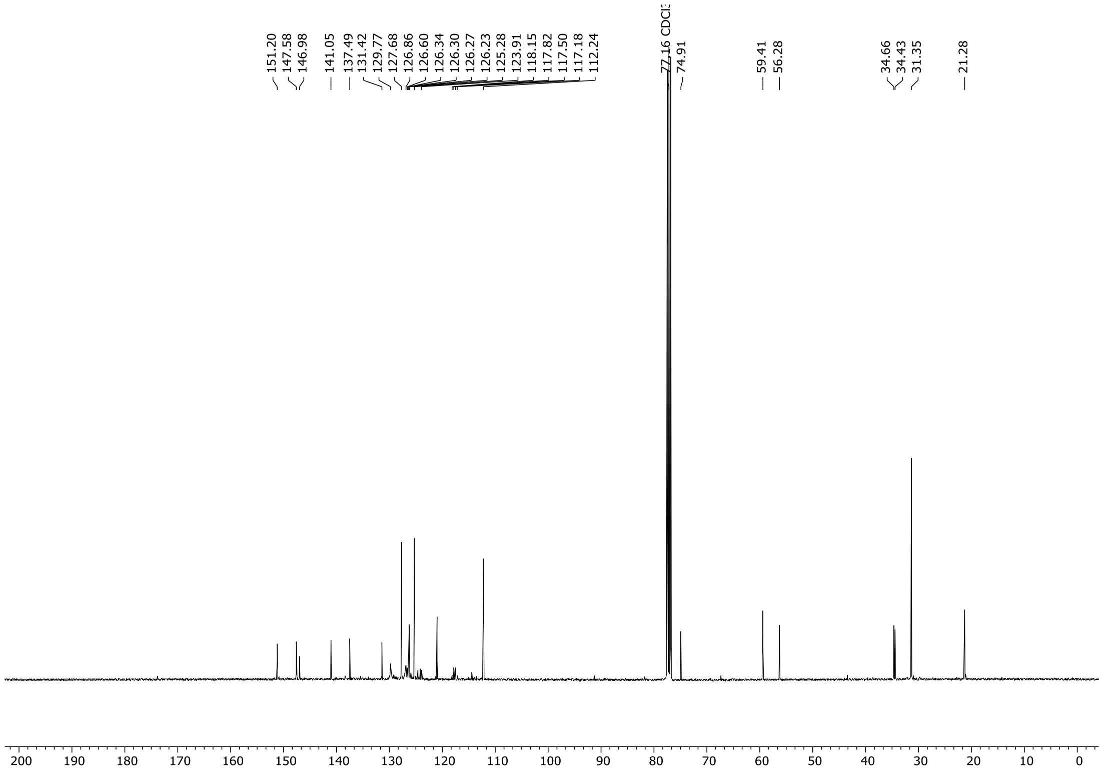

^{19}F NMR (376 MHz, $CDCl_3$)

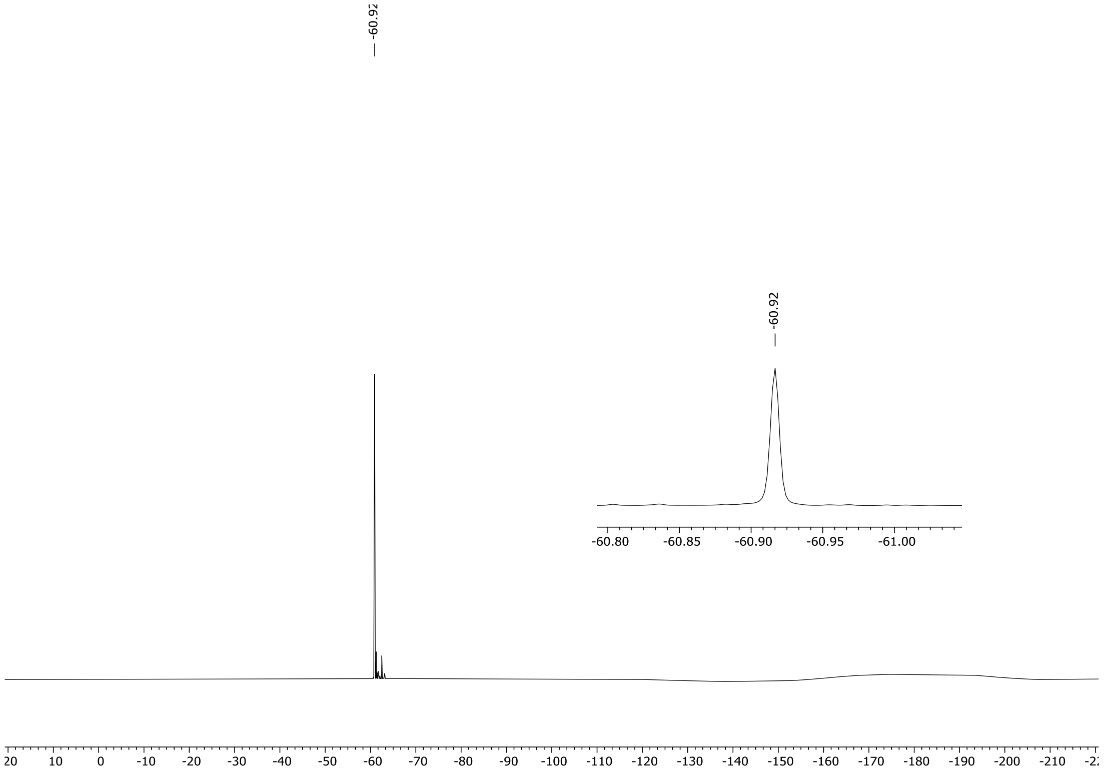

2-(3-(3-Methoxyphenyl)-2-(*p*-tolyl)-1-(4-(trifluoromethyl)phenyl)-2,5-dihydro-1H-pyrrol-2-yl)ethan-1-ol (7d)

^{1}H NMR (400 MHz, $CDCl_3$)

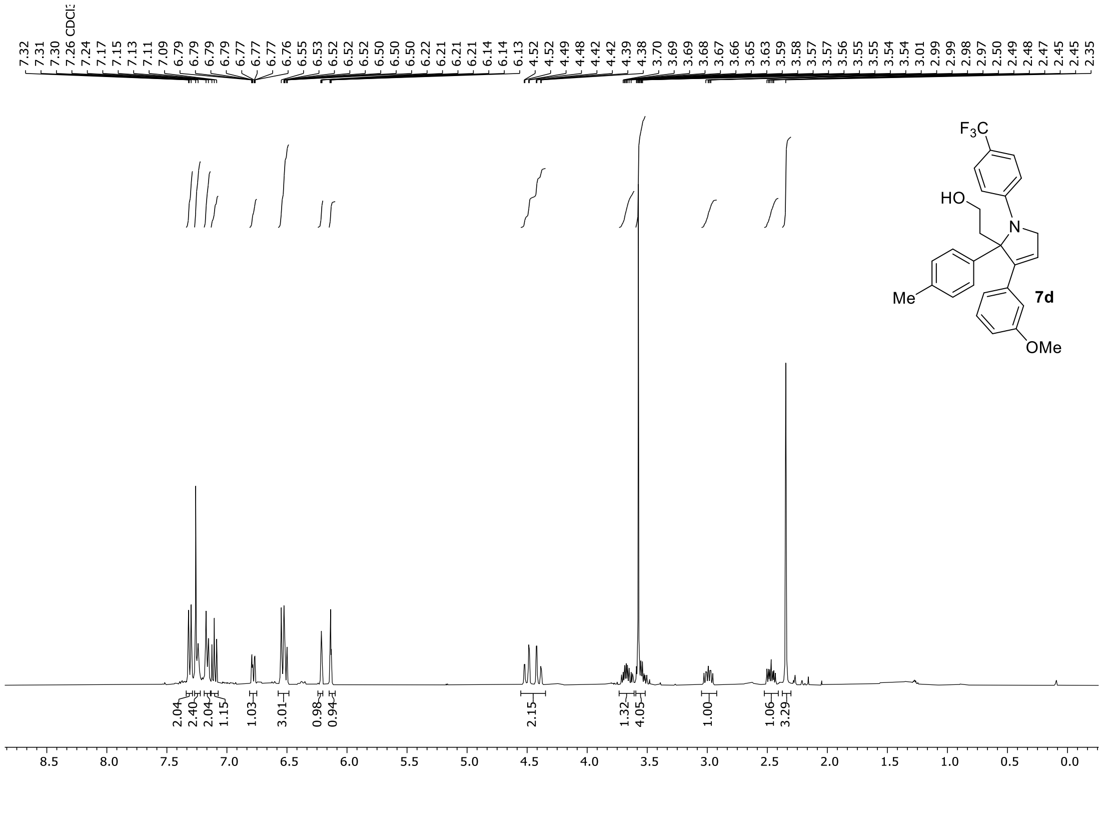

^{13}C NMR (101 MHz, $CDCl_3$)

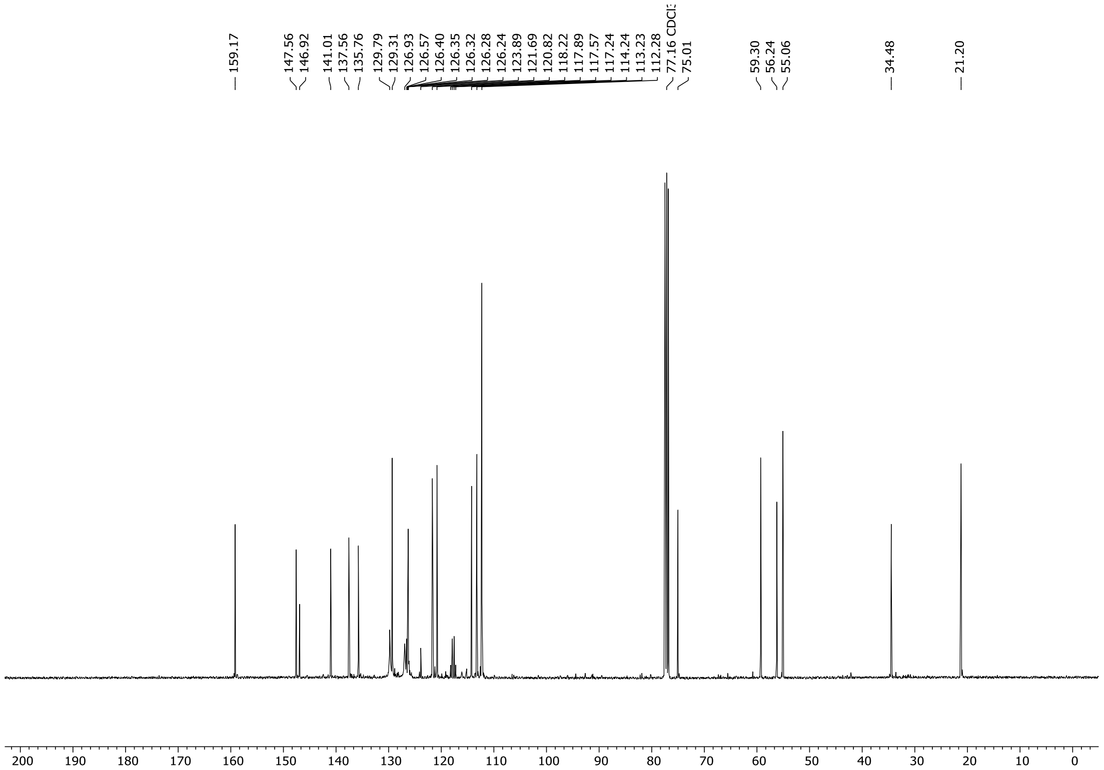

^{19}F NMR (376 MHz, $CDCl_3$)

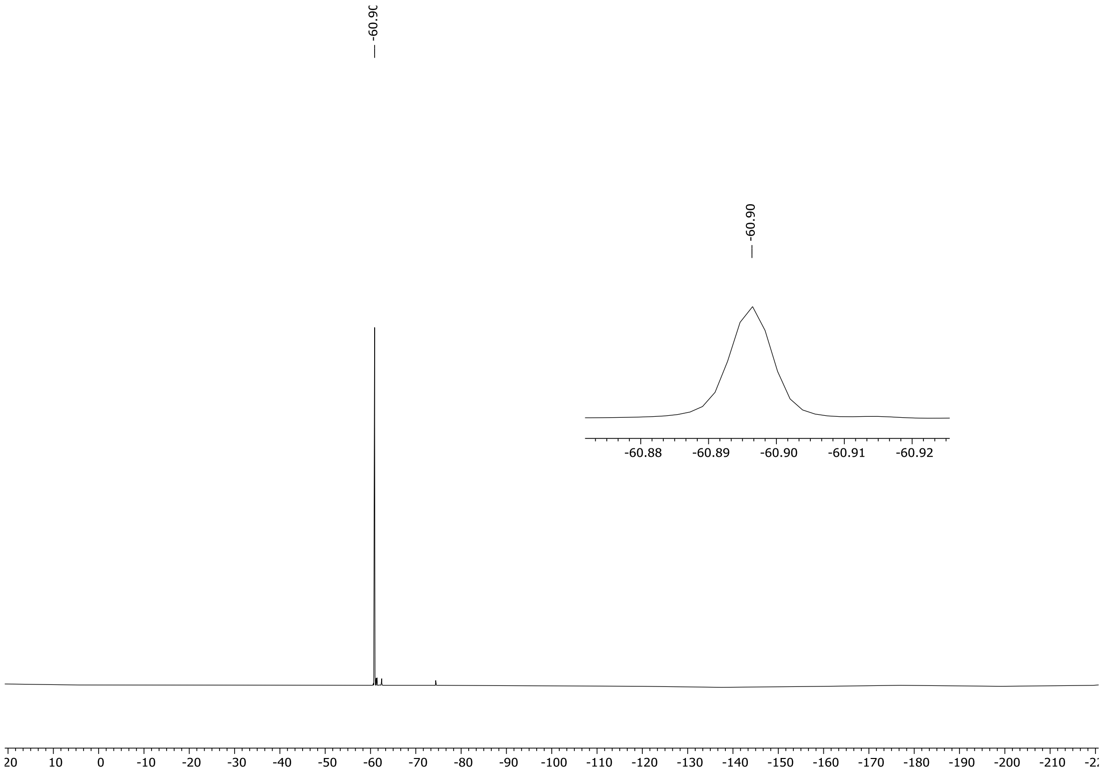

2-(3-(4-Chlorophenyl)-2-(*p*-tolyl)-1-(4-(trifluoromethyl)phenyl)-2,5-dihydro-1H-pyrrol-2-yl)ethan-1-ol (7e)

^{1}H NMR (400 MHz, $CDCl_3$)

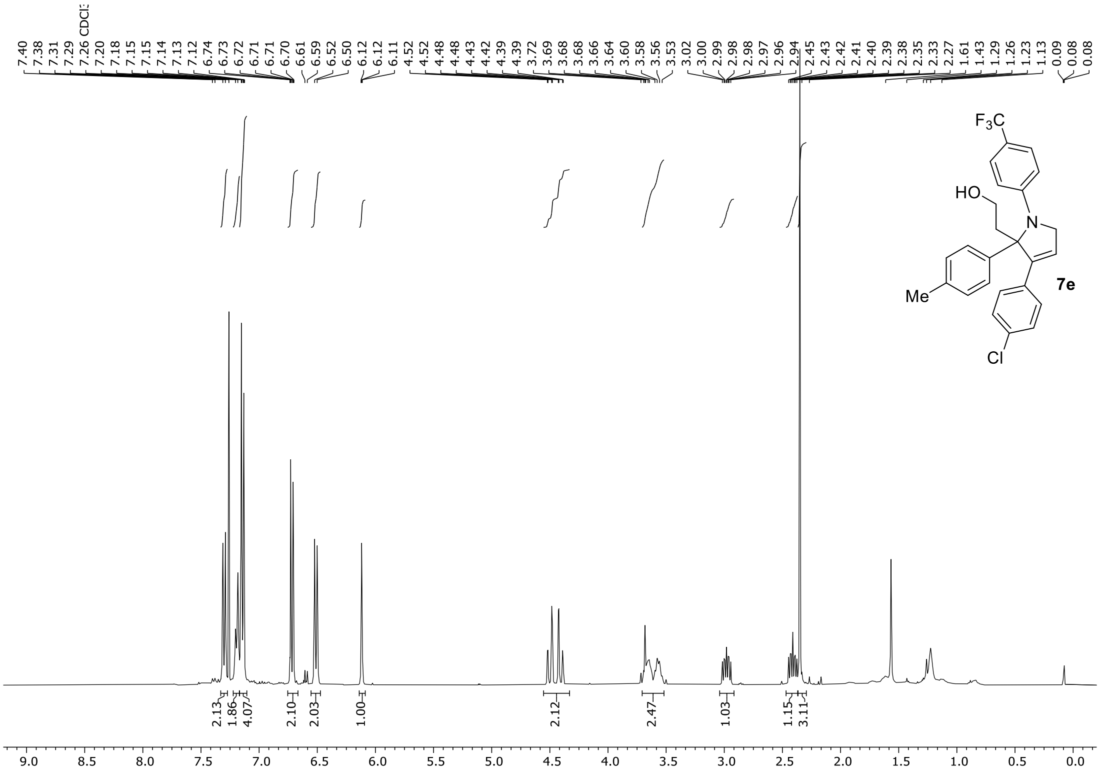

^{13}C NMR (101 MHz, $CDCl_3$)

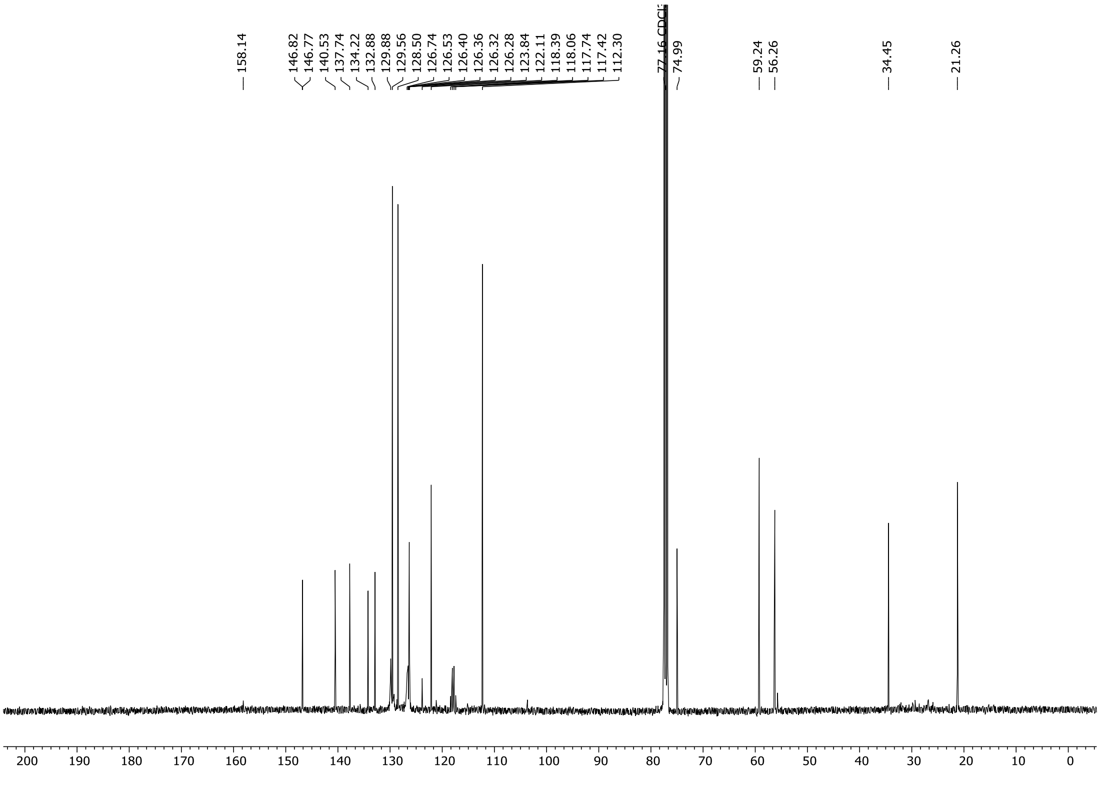

^{19}F NMR (376 MHz, $CDCl_3$)

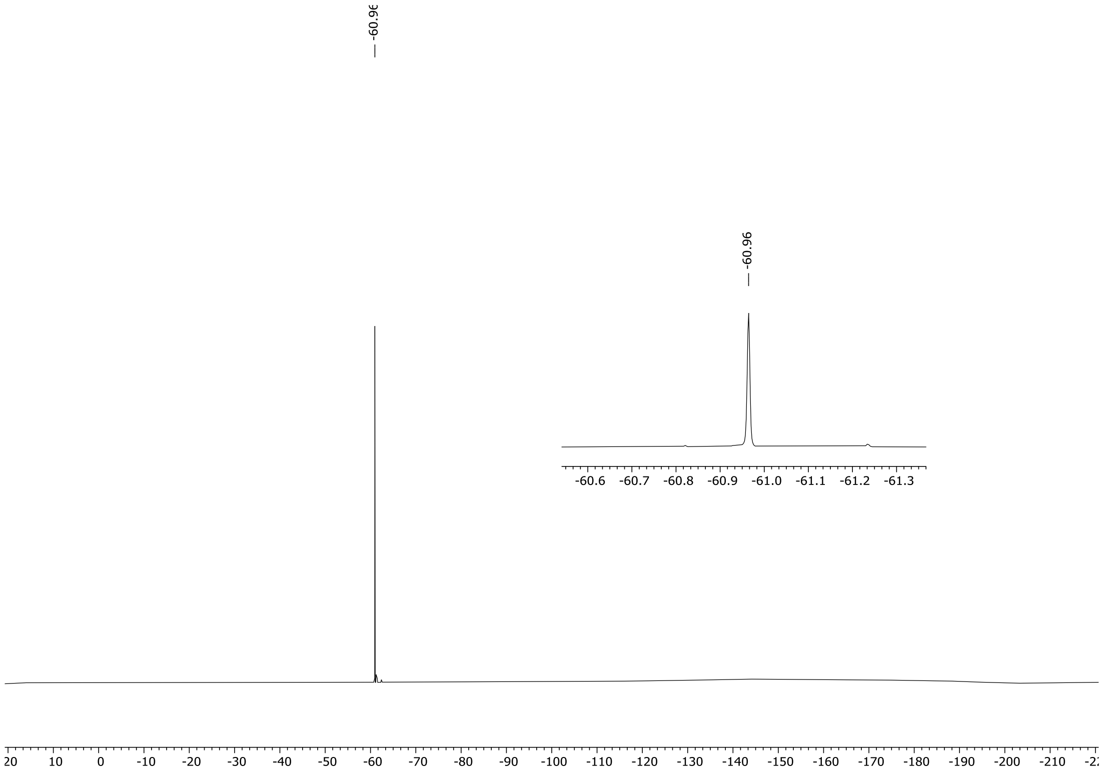

2-(3-(4-Fluorophenyl)-2-(*p*-tolyl)-1-(4-(trifluoromethyl)phenyl)-2,5-dihydro-1H-pyrrol-2-yl)ethan-1-ol (7f)

^{1}H NMR (400 MHz, $CDCl_3$)

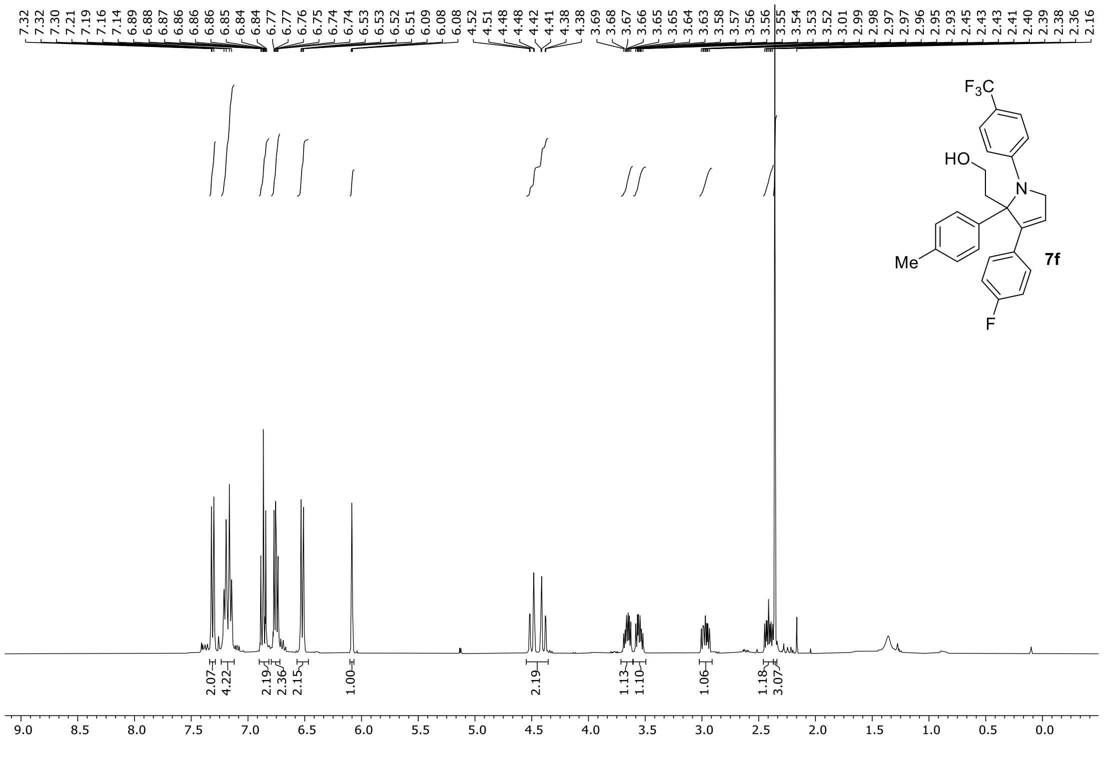

^{13}C NMR (101 MHz, $CDCl_3$)

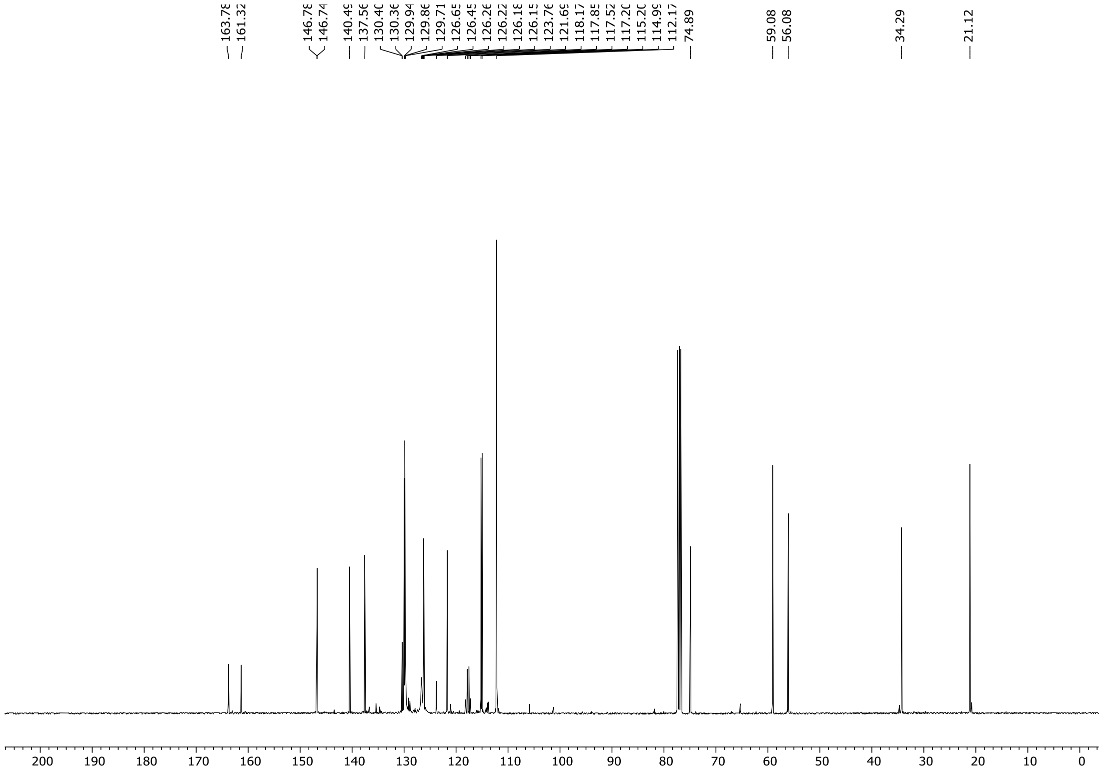

^{19}F NMR (376 MHz, $CDCl_3$)

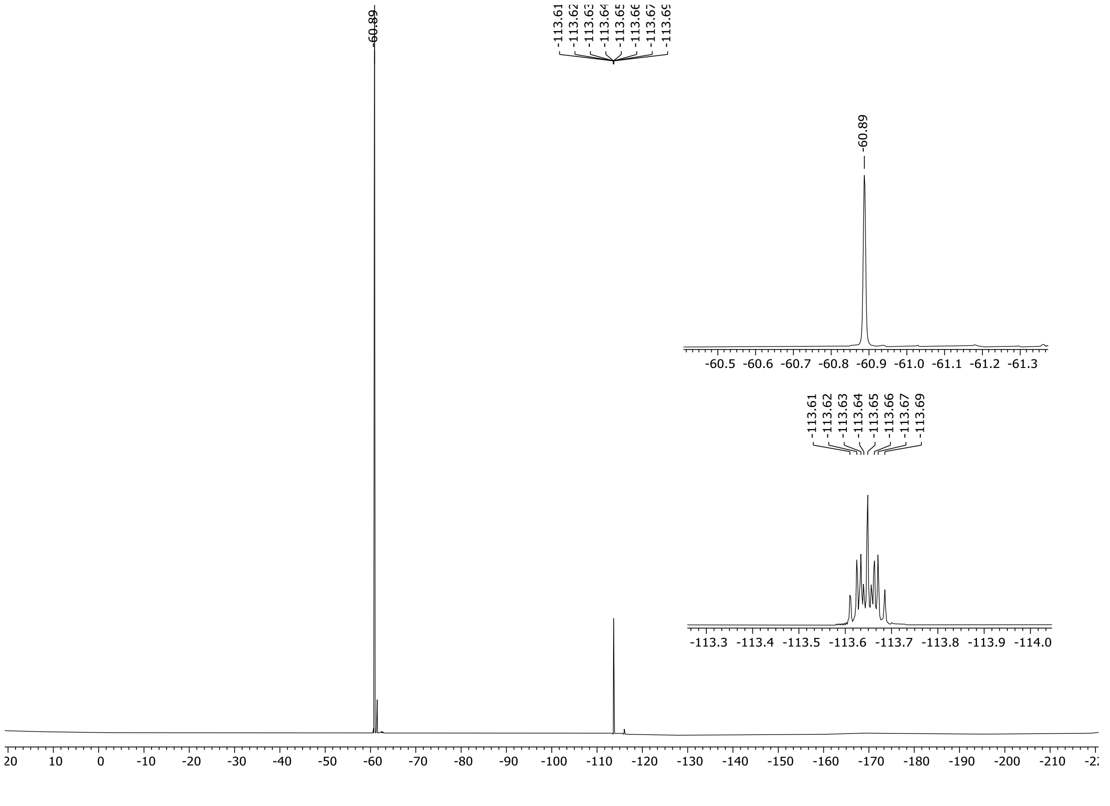

2-(3-(4-Nitrophenyl)-2-(*p*-tolyl)-1-(4-(trifluoromethyl)phenyl)-2,5-dihydro-1H-pyrrol-2-yl)ethan-1-ol (7g)

^{1}H NMR (400 MHz, $CDCl_3$)

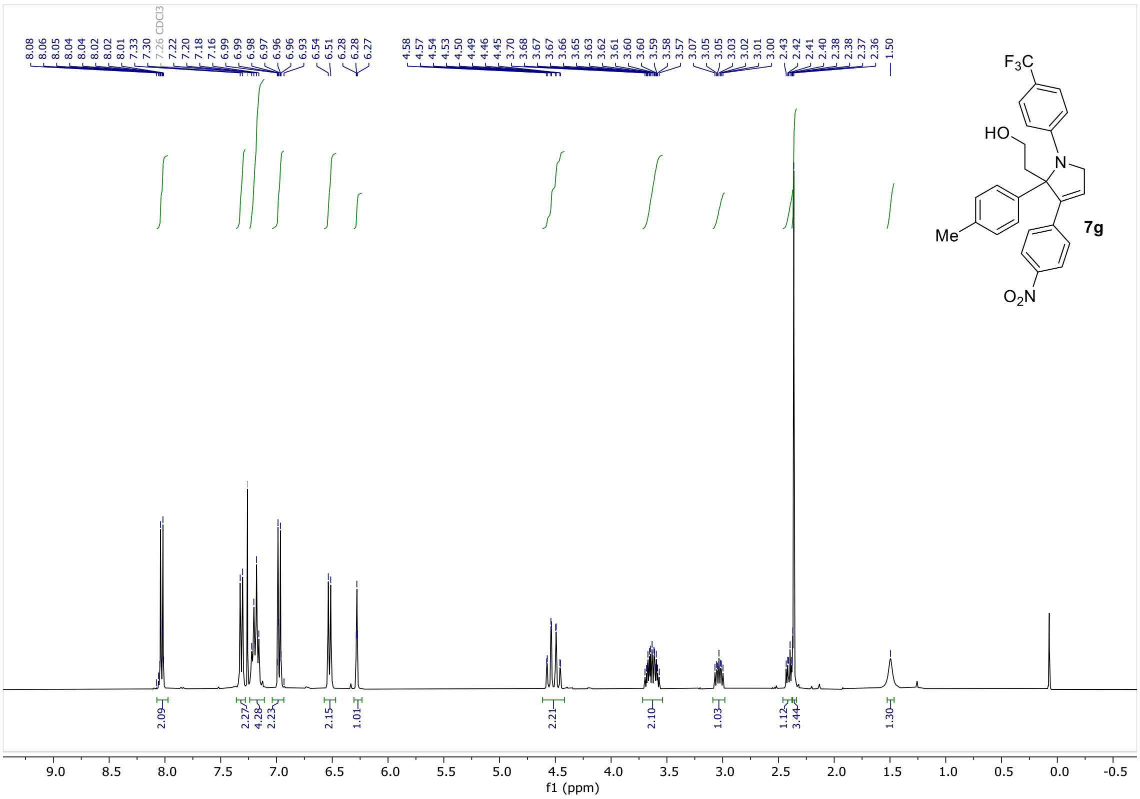

^{13}C NMR (101 MHz, $CDCl_3$)

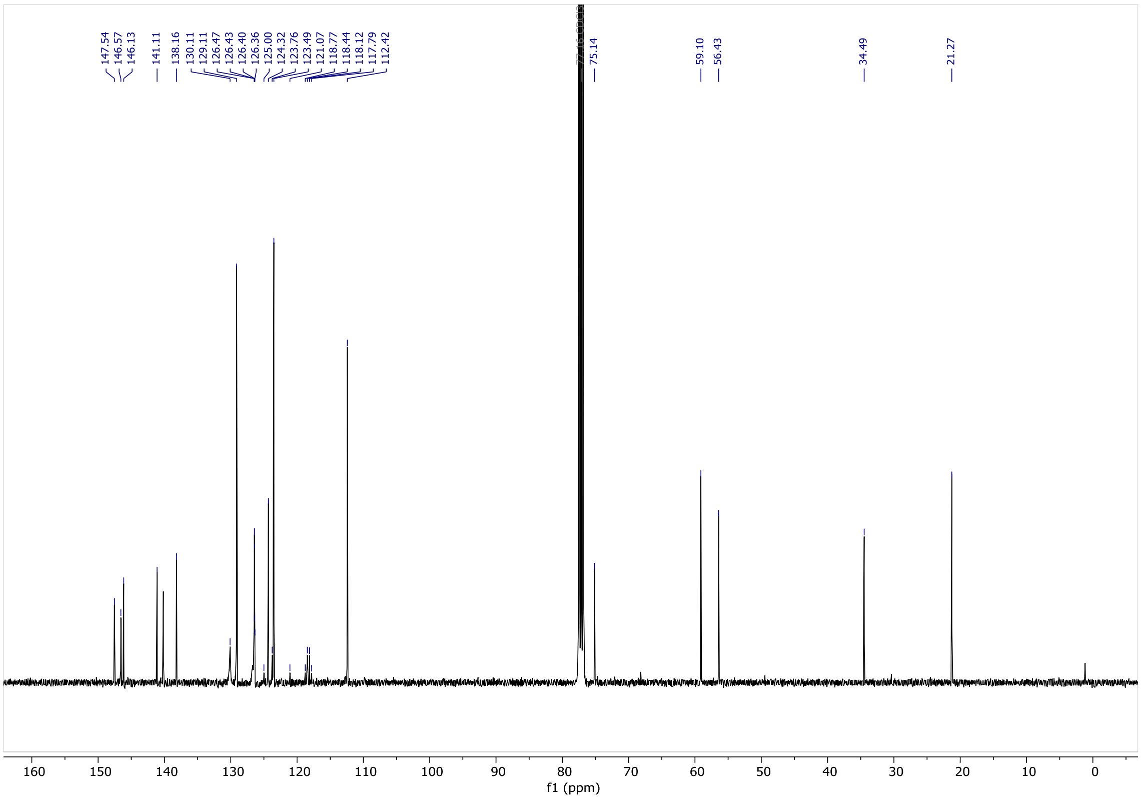

I.4. Spectra of product modifications

4-Methoxy-*N*-methyl-*N*-((4-(*p*-tolyl)-5,6-dihydro-2*H*-pyran-2-yl)methyl)aniline (8)

^{1}H NMR (400 MHz, $CDCl_3$)

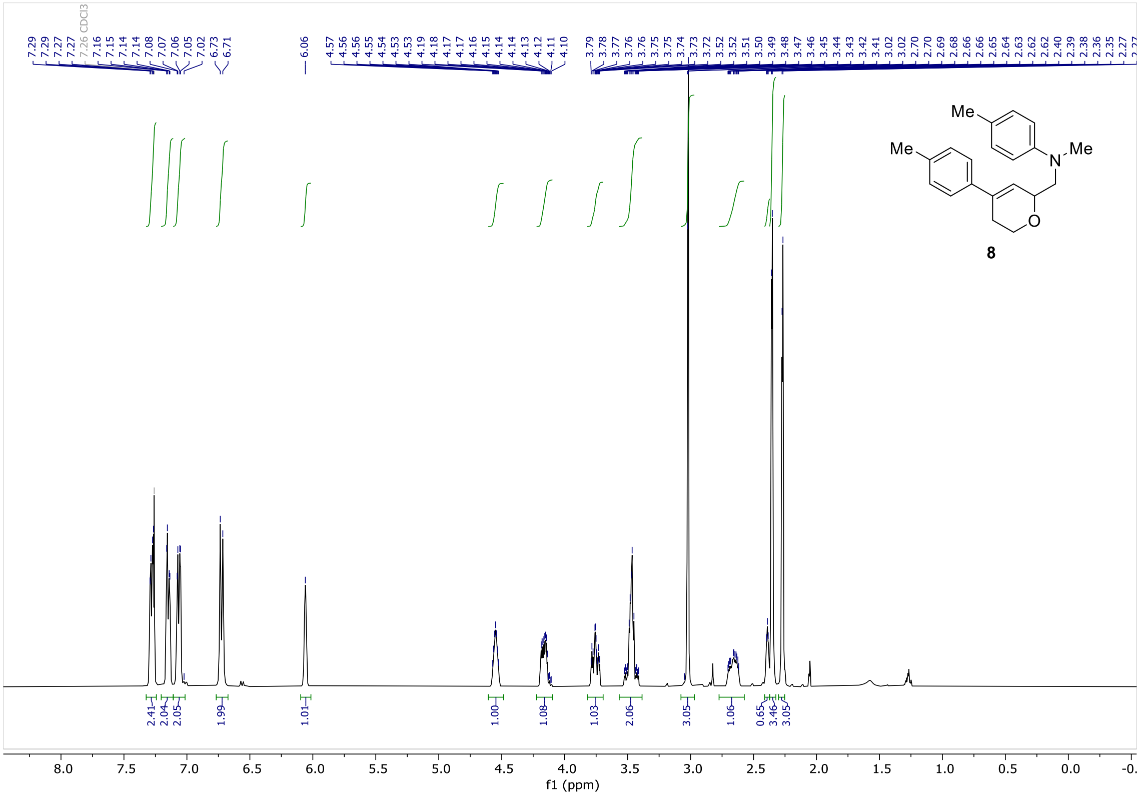

^{13}C NMR (101 MHz, $CDCl_3$)

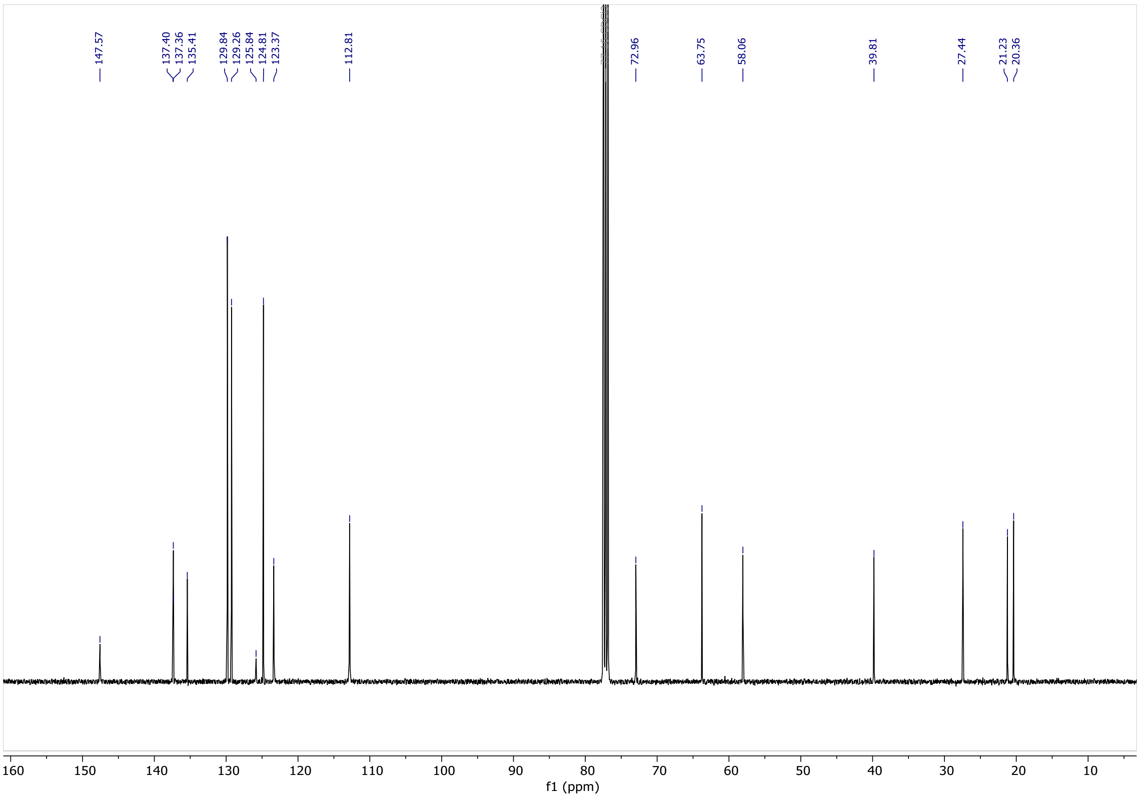

***Cis-N*,4-dimethyl-*N*-((-4-(p-tolyl)tetrahydro-2*H*-pyran-2-yl)methyl)aniline (9a)**

^{1}H NMR (400 MHz, $CDCl_3$)

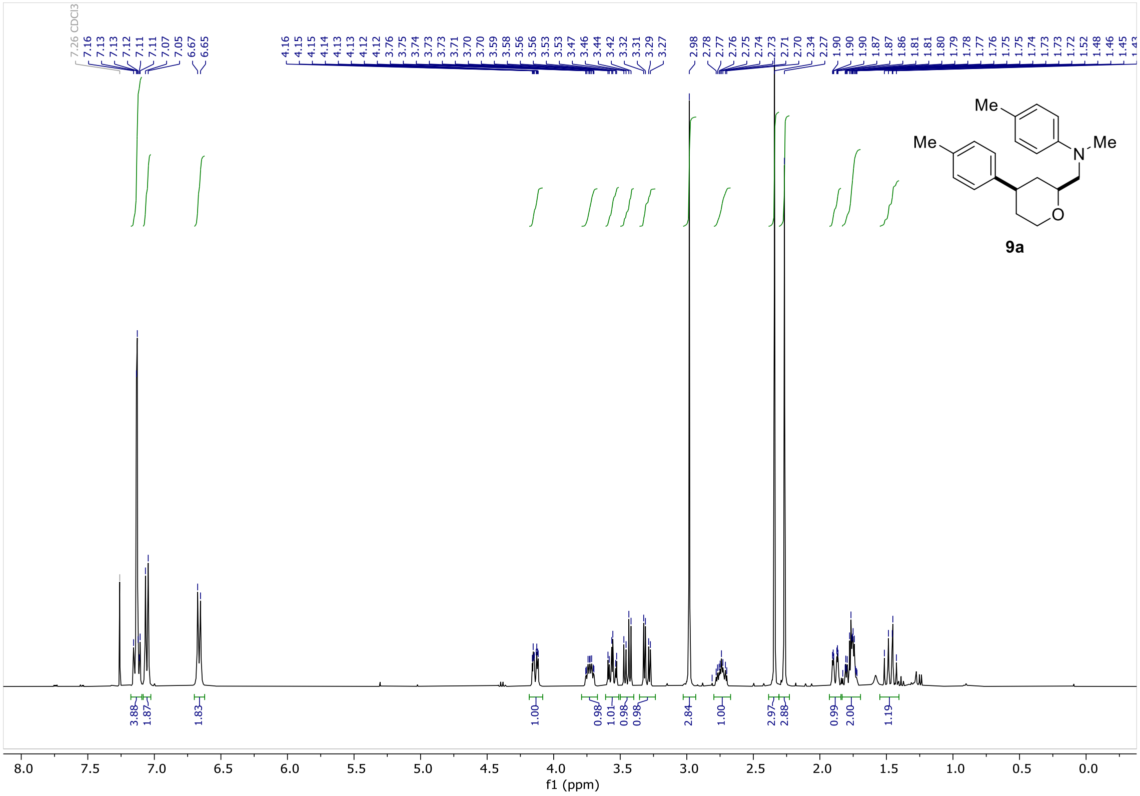

^{13}C NMR (101 MHz, $CDCl_3$)

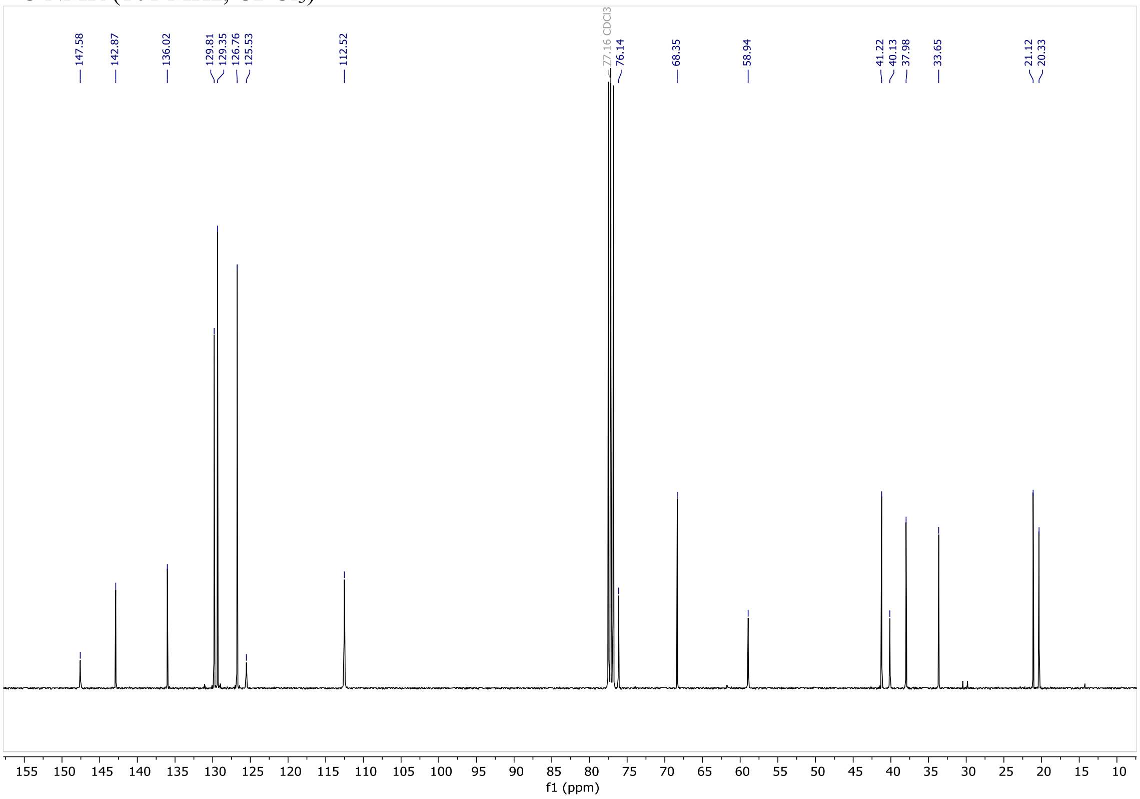

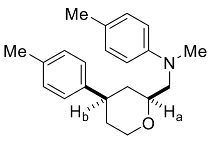
Me
Me
N
Me
H_b
H_a
O

9a

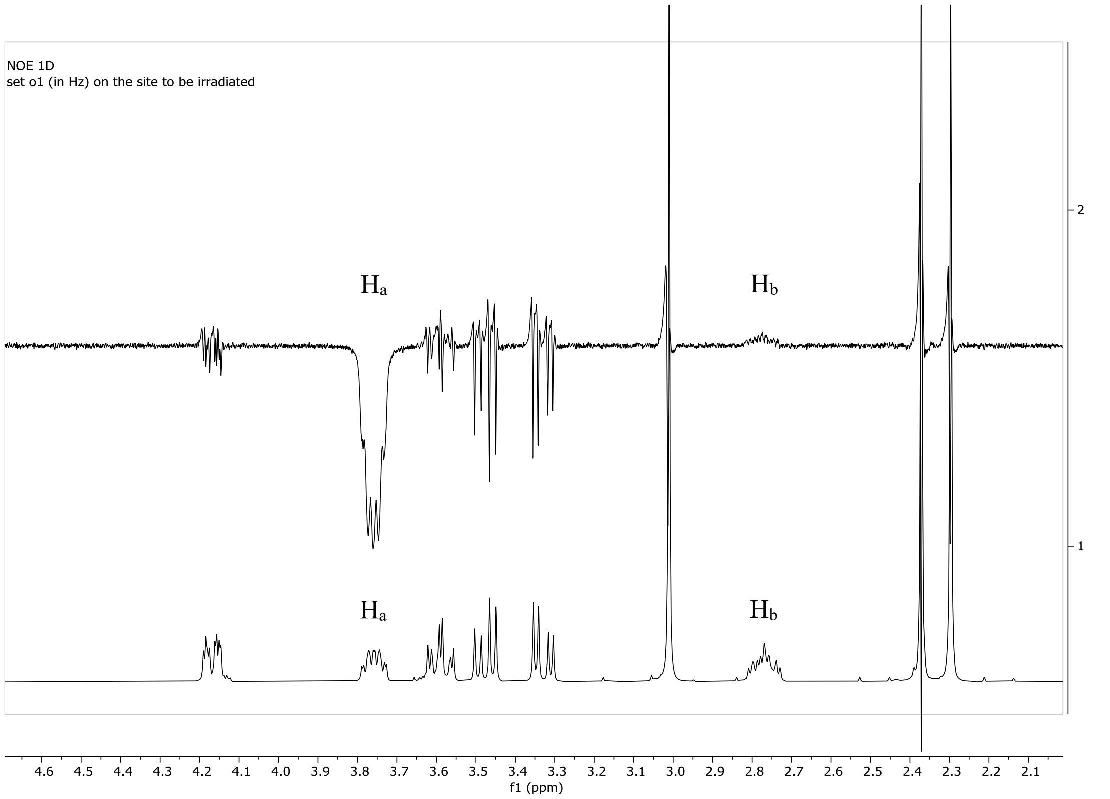
NOE 1D
set o1 (in Hz) on the site to be irradiated
H_a
H_b
H_a
H_b
f1 (ppm)

***Trans*-*N*,4-dimethyl-*N*-((-4-(p-tolyl)tetrahydro-2*H*-pyran-2-yl)methyl)aniline (9b)**

^{1}H NMR (400 MHz, $CDCl_3$)

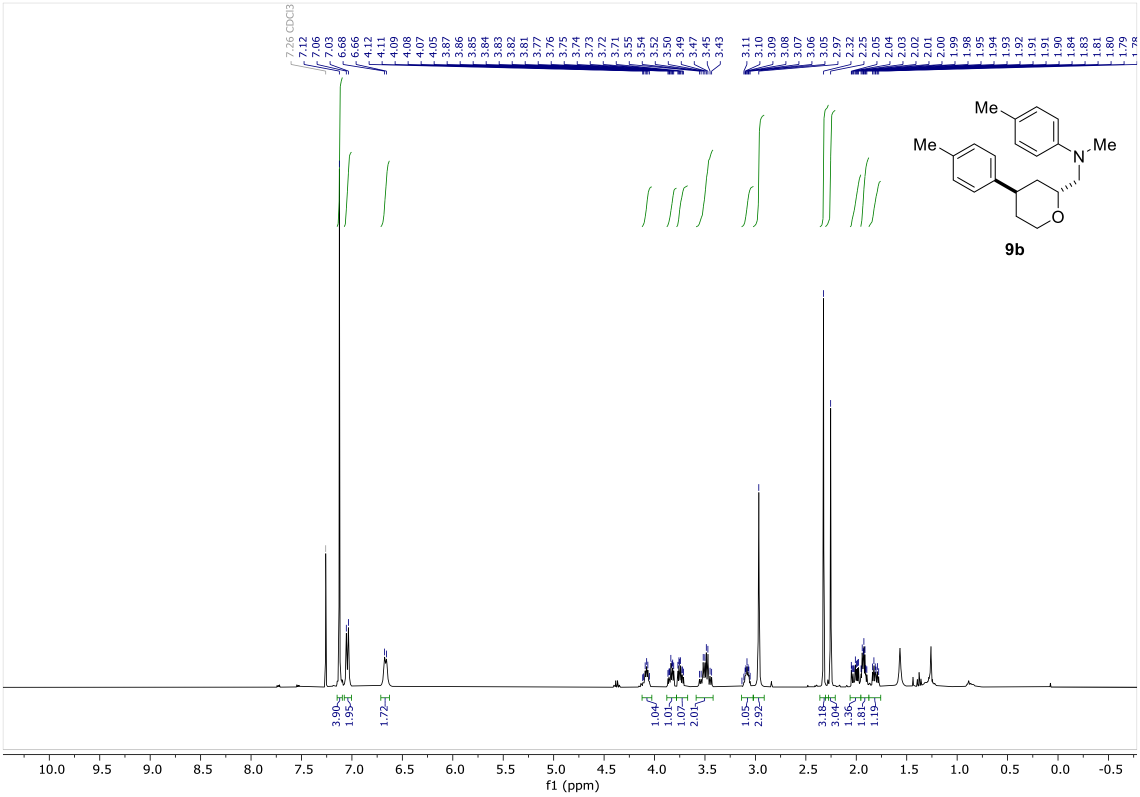

^{13}C NMR (101 MHz, $CDCl_3$)

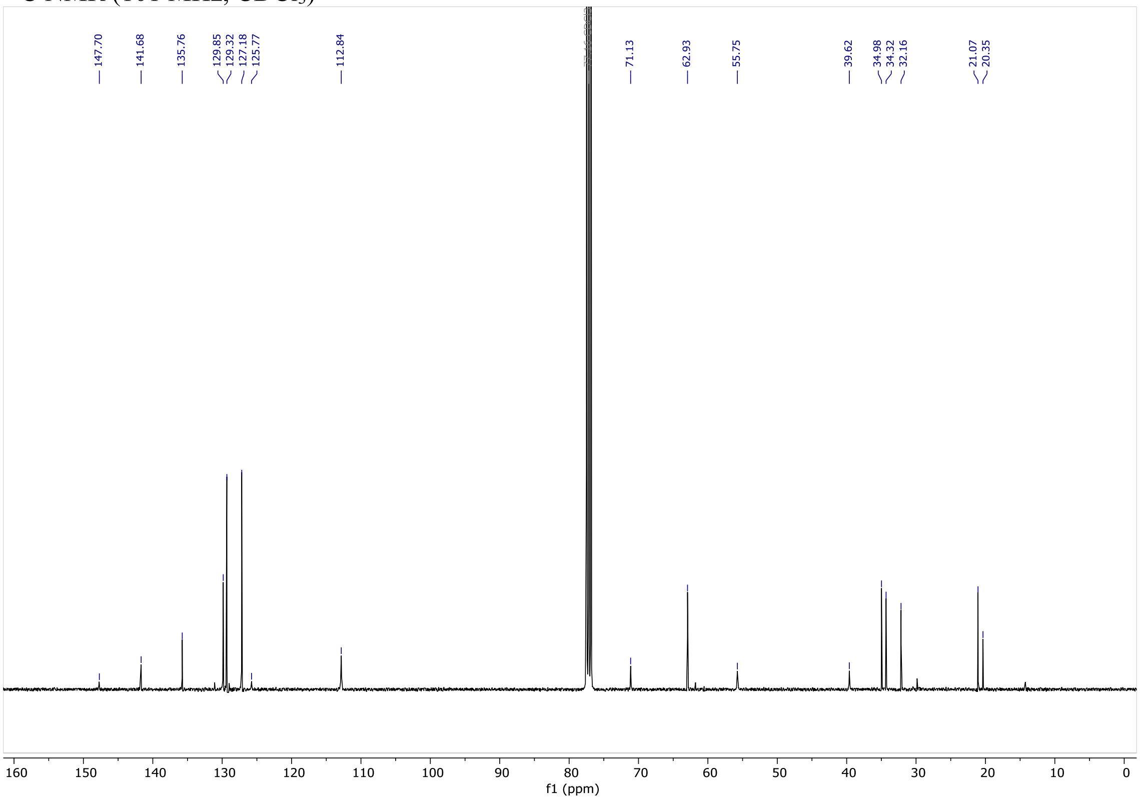

Methyl 4-(3-hydroxy-2-(2-hydroxyethyl)-2-(*p*-tolyl)-1-(4-(trifluoromethyl)phenyl)pyrrolidin-3-yl)benzoate (10)

^{1}H NMR (400 MHz, $CDCl_3$)

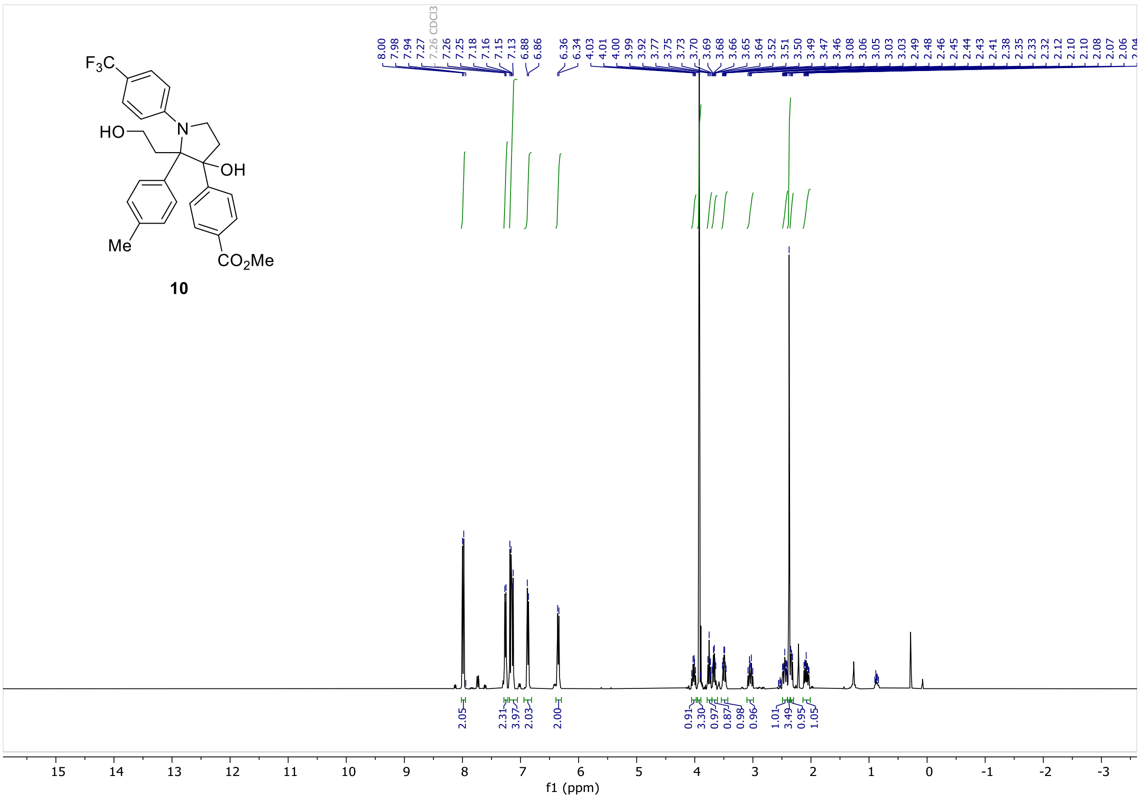

^{13}C NMR (101 MHz, $CDCl_3$)

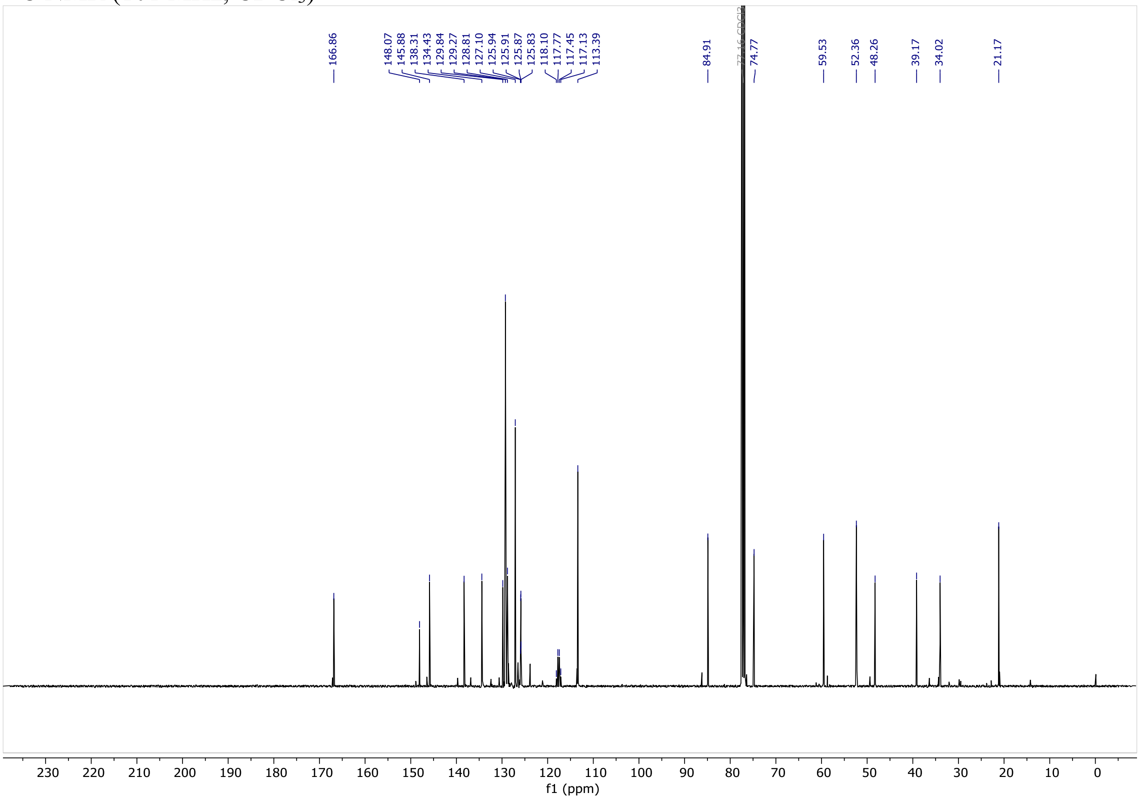

2-(2-(*p*-Tolyl)-1-(4-(trifluoromethyl)phenyl)-2,5-dihydro-1*H*-pyrrol-2-yl)ethan-1-ol (11)

^{1}H NMR (400 MHz, $CDCl_3$)

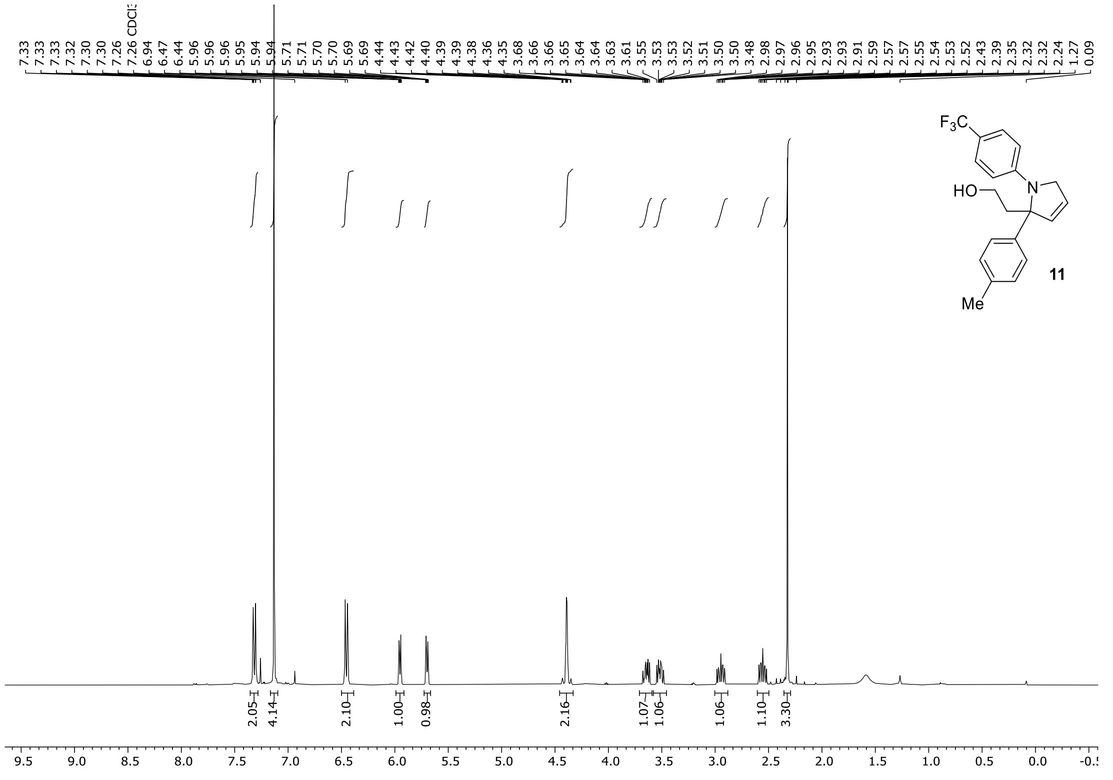

^{13}C NMR (101 MHz, $CDCl_3$)

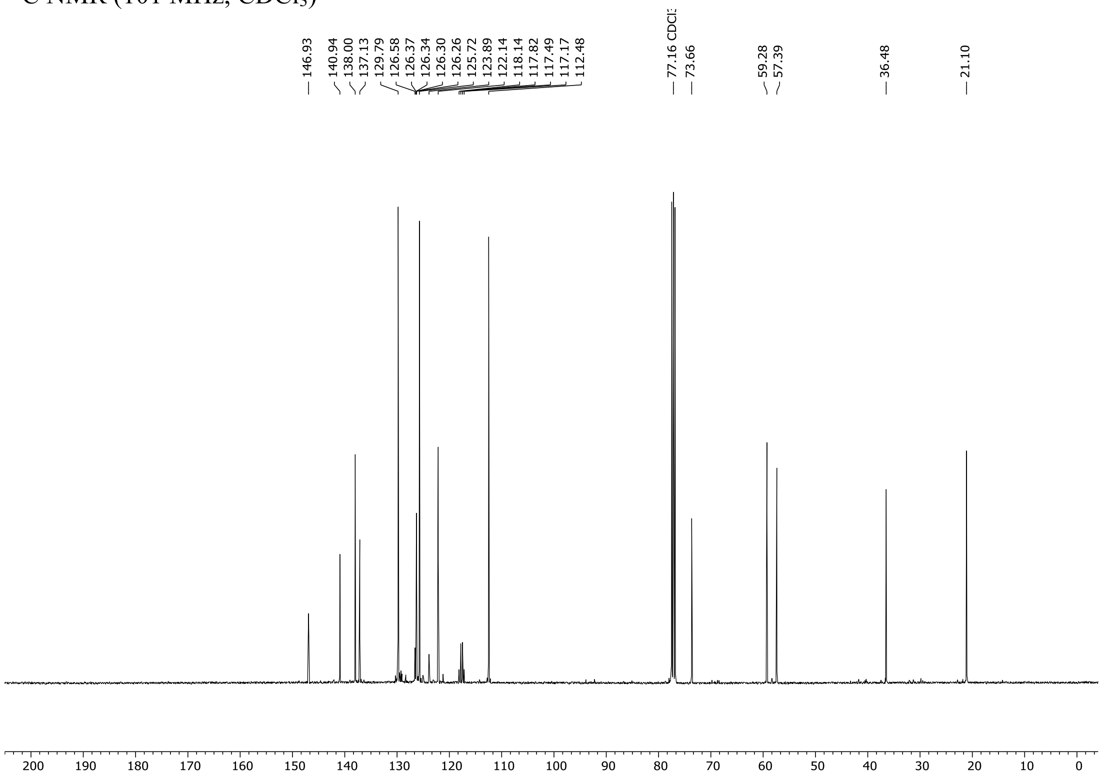

^{19}F NMR (376 MHz, $CDCl_3$)

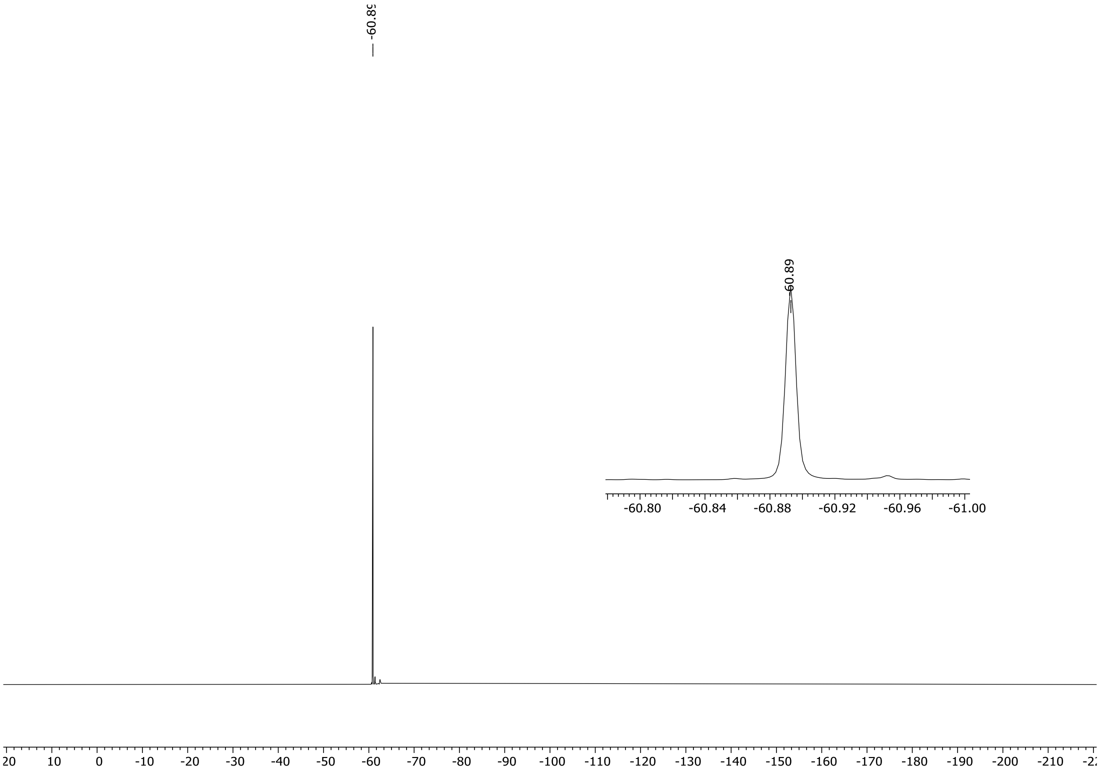

2-(2-(*p*-Tolyl)-1-(4-(trifluoromethyl)phenyl)pyrrolidin-2-yl)ethan-1-ol (12)

^{1}H NMR (400 MHz, $CDCl_3$)

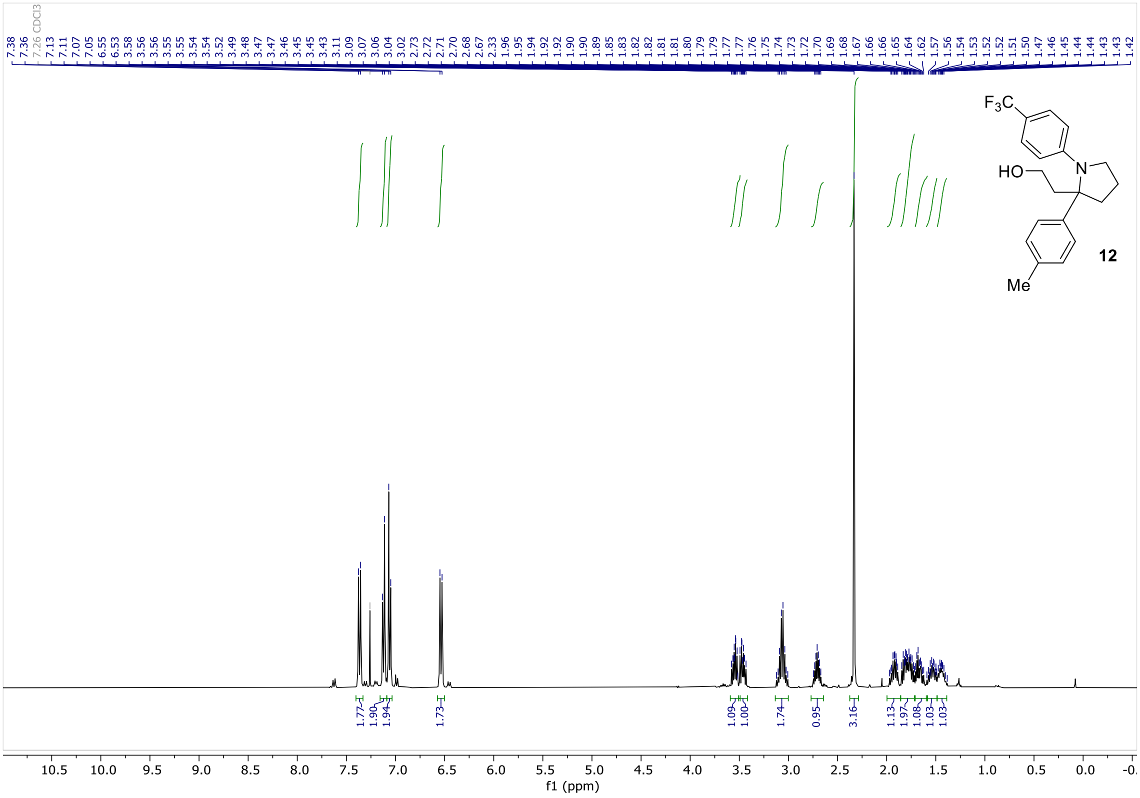

^{13}C NMR (101 MHz, $CDCl_3$)

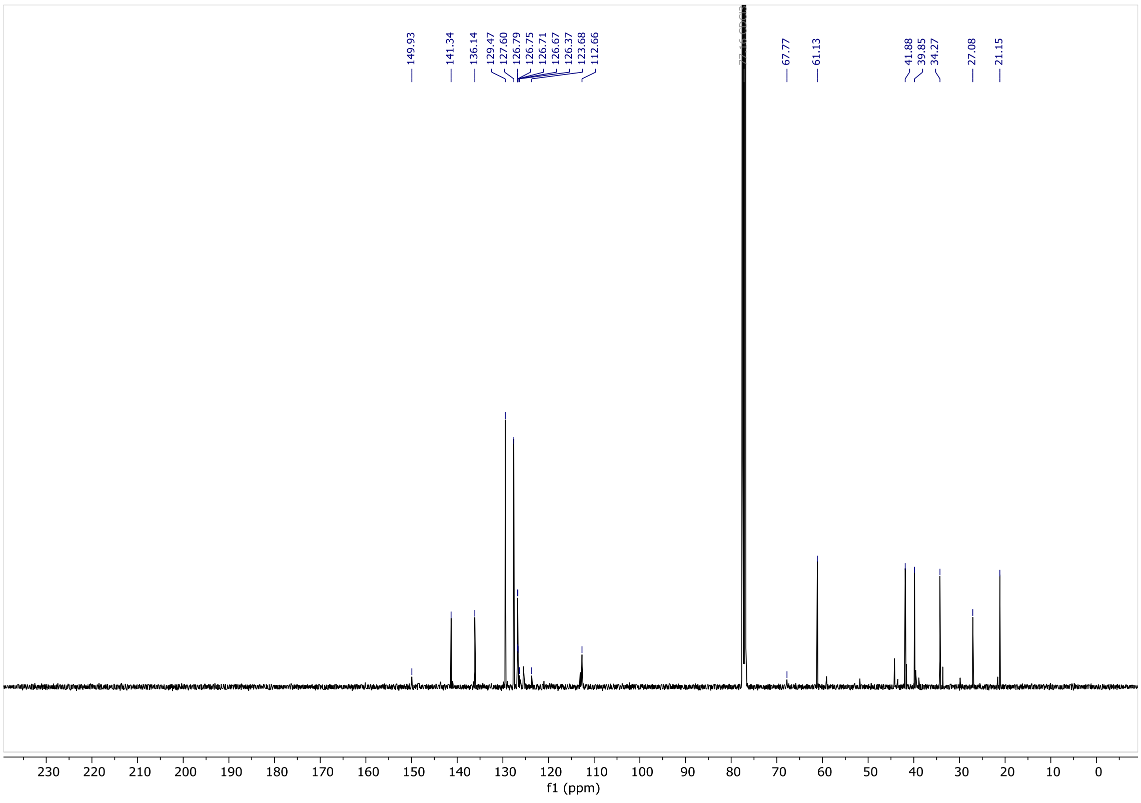

6-(Methyl(*p*-tolyl)amino)-3-(*p*-tolyl)hexa-3,4-dien-1-yl sulfamate (13)

^{1}H NMR (400 MHz, $CDCl_3$)

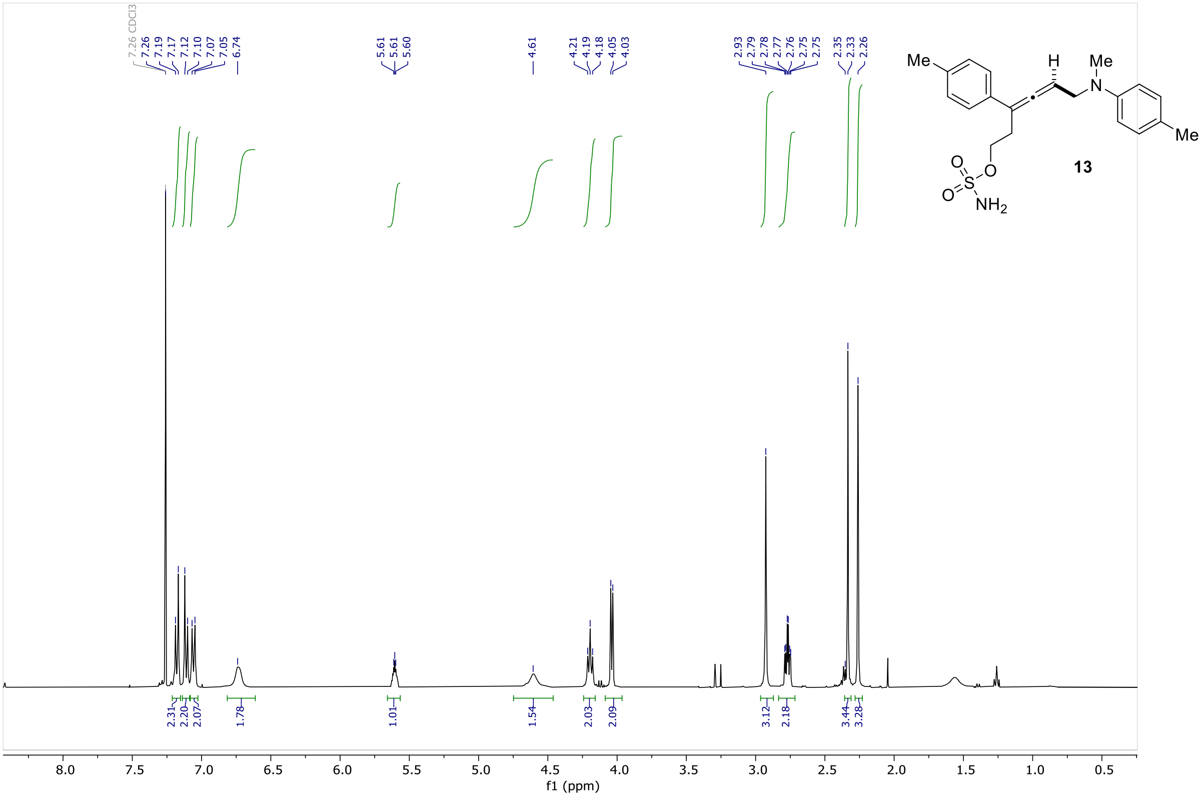

^{13}C NMR (101 MHz, $CDCl_3$)

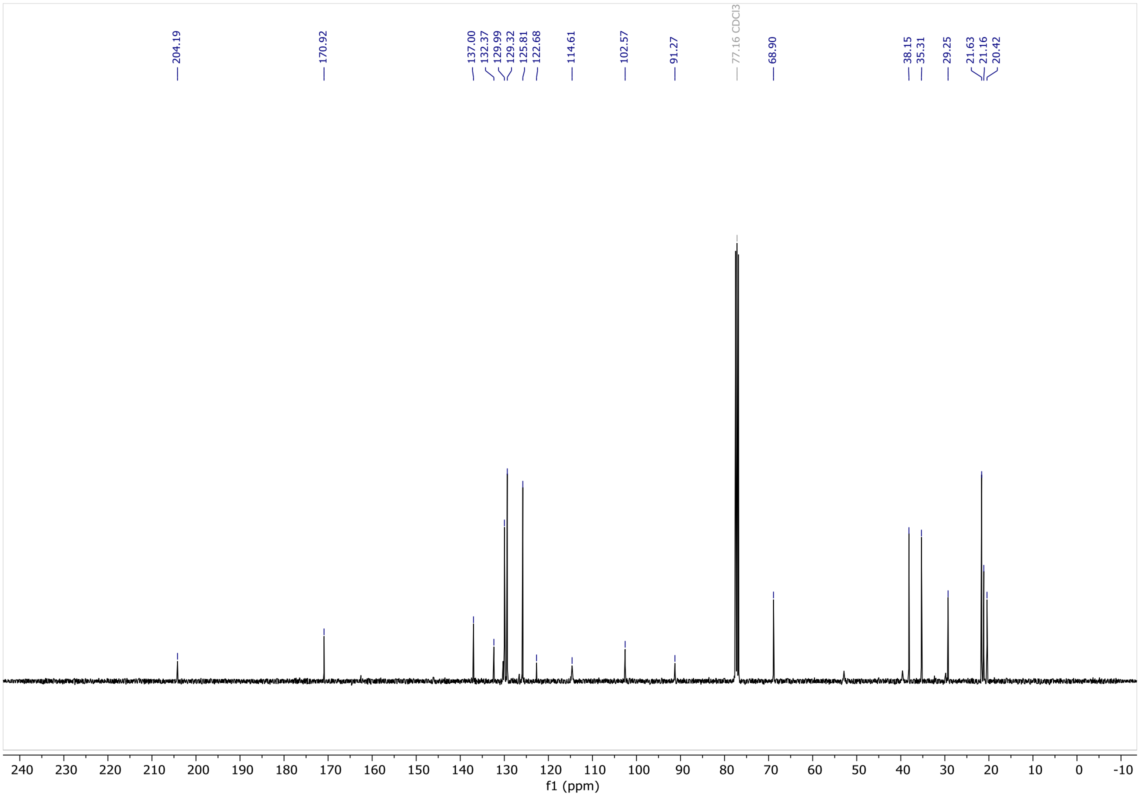

5,9-Bis(4-methylphenyl)-1-oxa-3,5-diaza-2,2-dioxo-2-thiacycloundeca-3,7,8-triene (14)

^{1}H NMR (400 MHz, $CDCl_3$)

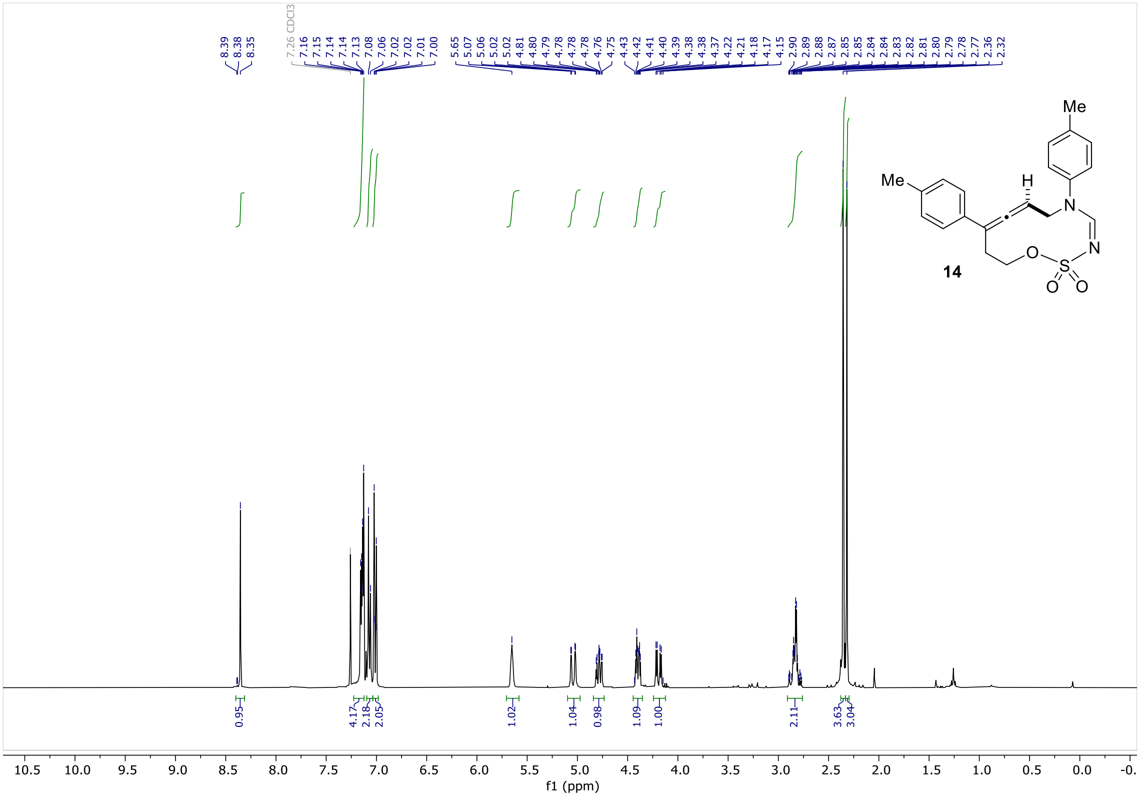

^{13}C NMR (101 MHz, $CDCl_3$)

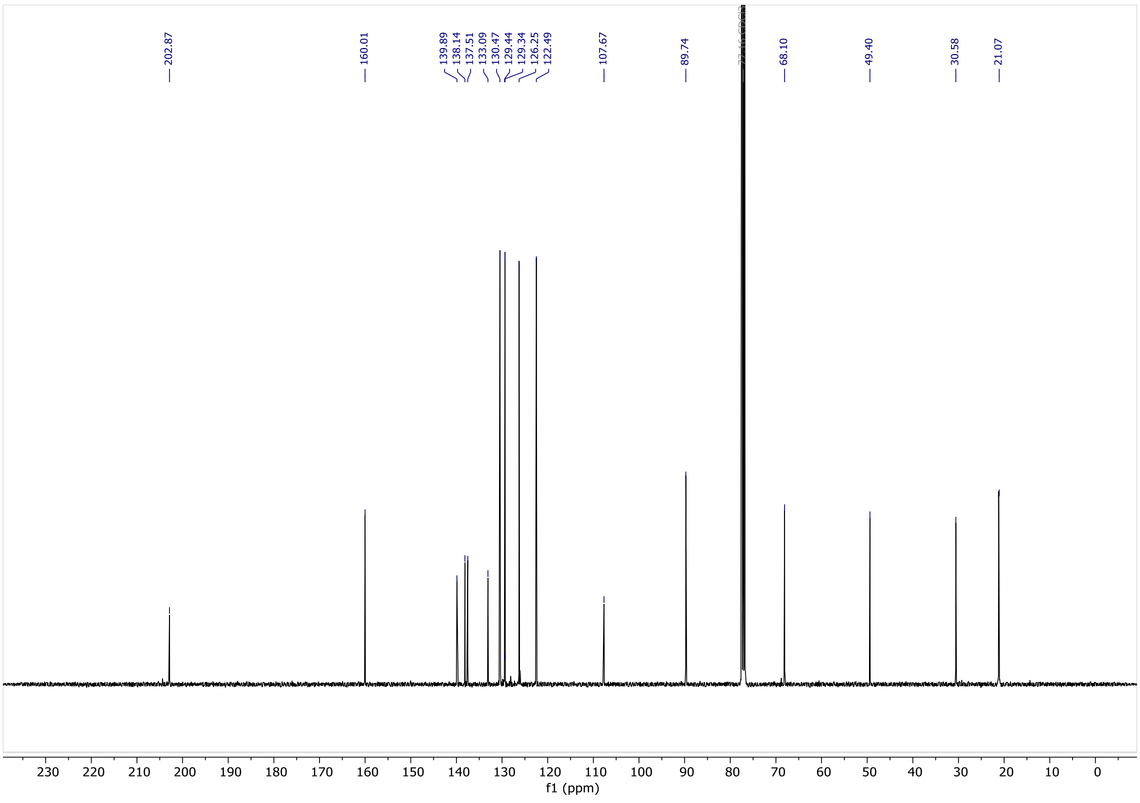

Supplement: Supplementary file 1 — General methods, experimental procedures, characterization data, and NMR spectra for new compounds. Raw NMR, MS, and IR is available at zenodo.org: https://doi.org/105281/zenodo.18851416. Supporting File: anie72348‐sup‐0001‐SuppMat.pdf. [file ANIE-65-e6963888-s001.pdf]
